# Supplementary material for: Severely Ill COVID-19 Patients May Exhibit Hypercoagulability Despite Escalated Anticoagulation
Source: J Clin Med. 2025 Mar 14;14(6):1966. doi: 10.3390/jcm14061966 (PMC11943368; doi:10.3390/jcm14061966)
Supplement: Supplementary file 1 [file jcm-14-01966-s001.zip › File S1. NationalGuidance.pdf]

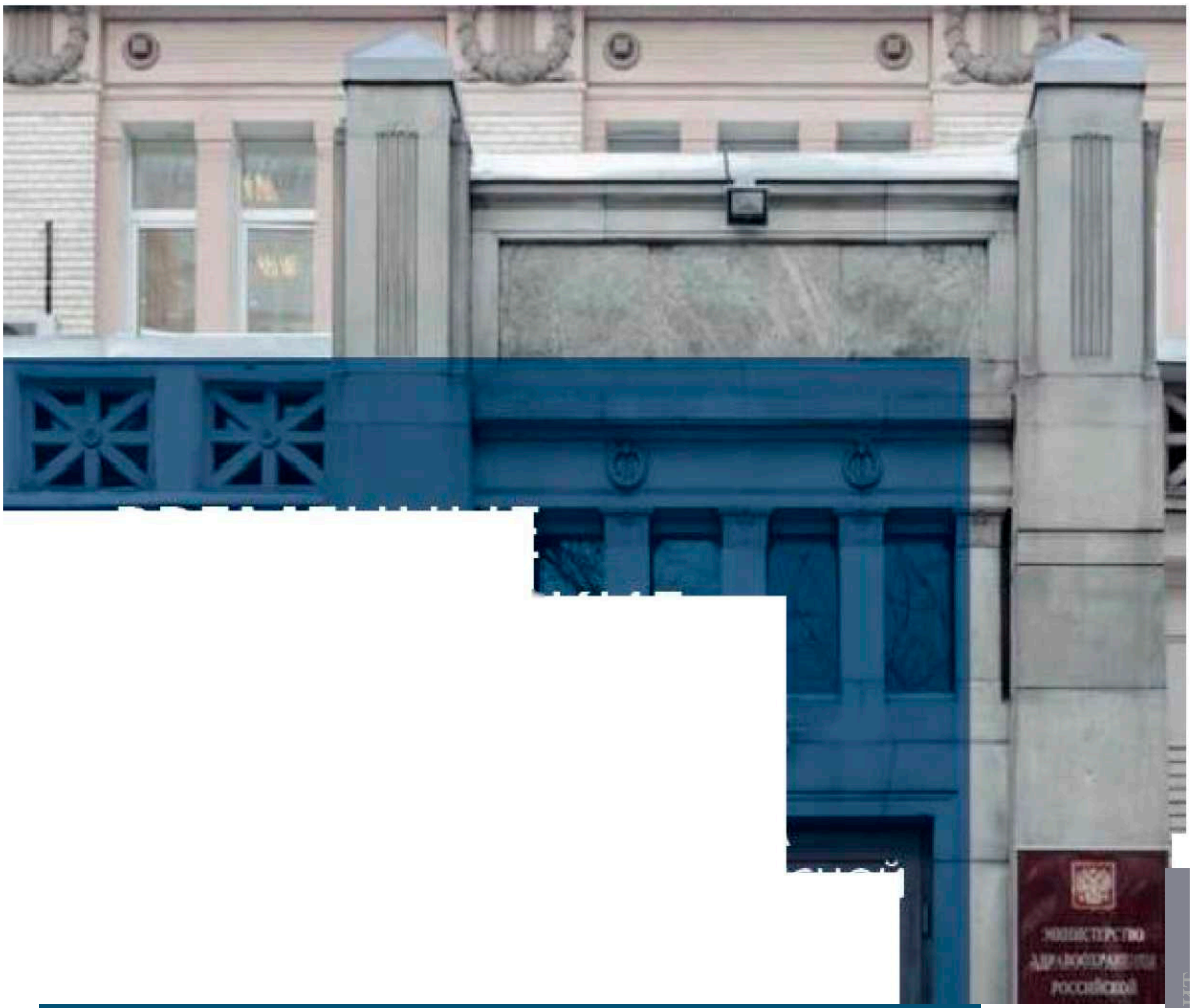

TEMPORARY GUIDELINES

PREVENTION, DIAGNOSIS AND TREATMENT OF THE NEW CORONAVIRUS INFECTION (COVID-19)

МИНИСТЕРСТВО
ЗДРАВООХРАНЕНИЯ
РОССИЙСКОЙ ФЕДЕРАЦИИ

I APPROVE
Deputy Minister of Health
Russian Federation
E.G. Kamkin

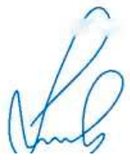

TEMPORARY METHODOLOGICAL RECOMMENDATIONS

PREVENTION, DIAGNOSTICS AND TREATMENT OF THE NEW CORONAVIRUS INFECTIONS (COVID-19)

TABLE OF CONTENTS

TABLE OF CONTENTS 3
INTRODUCTION 5
1. ETIOLOGY, PATHOGENESIS AND PATHOMORPHOLOGY 6
2. EPIDEMIOLOGICAL CHARACTERISTICS 13
3. CLINICAL FEATURES 16
4. DIAGNOSTICS OF CORONAVIRUS INFECTION 21
4.1. ALGORITHM FOR EXAMINING A PATIENT WITH SUSPECTED COVID-19 21
4.2. LABORATORY DIAGNOSTICS OF COVID-19 22
4.3. RADIATION DIAGNOSTICS OF COVID-19 31
4.4. DIFFERENTIAL DIAGNOSIS OF COVID-19 37
5. TREATMENT OF CORONAVIRUS INFECTION 38
5.1. ETIOTROPIC TREATMENT 38
5.2. PATHOGENETIC TREATMENT 45
5.3. SYMPTOMATIC TREATMENT 58
5.4. ANTIBACTERIAL THERAPY FOR COMPLICATED FORMS OF INFECTION 61
5.5. OBSTETRIC TACTICS IN COVID-19 67
5.6. BASIC PRINCIPLES OF EMERGENCY TREATMENT 71
5.7. SPECIAL PATIENT GROUPS 87
5.8. MONITORING CLINICAL AND LABORATORY INDICATORS 96
5.9. MEDICAL REHABILITATION IN PROVIDING SPECIALISED MEDICAL CARE TO PATIENTS WITH COVID-19 97
5.10. PROCEDURE FOR DISCHARGE (TRANSFER) OF PATIENTS FROM A MEDICAL ORGANIZATION 98
5.11. FEATURES OF DISPENSARY OBSERVATION AND IN-DEPTH TREATMENT DISPENSARY EXAMINATION OF CITIZENS WHO HAVE SUFFERED THE NEW CORONAVIRUS COVID-19 ....INFECTION 100
6. PECULIARITIES OF CARE FOR CHILDREN WITH COVID-19 104
7. PREVENTION OF CORONAVIRUS INFECTION 108
7.1. SPECIFIC PREVENTION OF COVID-19 IN ADULTS 108
7.2. NON-SPECIFIC PREVENTION OF COVID-19 112
7.3. MEDICATION PREVENTION OF COVID-19 IN ADULTS 113
7.4. MEASURES TO PREVENT THE SPREAD OF COVID-19 IN MEDICAL ORGANIZATION 113
7.5. RATIONAL USE OF PERSONAL PROTECTIVE EQUIPMENT IN MEDICAL ORGANIZATIONS 118
8. PROCEDURE FOR CONDUCTING PATHOLOGICAL ANATOMICAL AUTOPSIES 123
9. ROUTING OF PATIENTS AND FEATURES OF EVACUATION MEASURES FOR PATIENTS OR PERSONS SUSPECTED TO HAVE COVID-19 125
9.1. ROUTING OF PATIENTS AND PERSONS WITH SUSPECTED COVID-19 125
9.2. ORGANIZATION OF MEDICAL CARE 126

9.3. FEATURES OF EVACUATION MEASURES AND GENERAL PRINCIPLES HOSPITALIZATION OF PATIENTS OR PERSONS WITH SUSPECTED COVID-19 131
9.4. PROCEDURE FOR HOSPITALIZATION OF PATIENTS IN MEDICAL ORGANIZATIONS DEPENDING ON THE SEVERITY OF THE DISEASE 135
9.5. BASIC PRINCIPLES OF PROVIDING MEDICAL CARE IN OUTPATIENT SETTINGS (AT HOME) 139
9.6. ROUTING OF PATIENTS REQUIRING EMERGENCY SURGICAL CARE 142
10. RULES FOR FORMULATION OF DIAGNOSIS, CODING ACCORDING TO ICD-10 AND ACCOUNTING PATIENTS WITH COVID-19 IN THE INFORMATION RESOURCE 146
References 151
Appendix 1 160
Appendix 2 179
Appendix 3-1 180
Appendix 3-2 186
Appendix 3-3 190
Appendix 3-4 187
Appendix 4 194
Appendix 5 195
Appendix 6 197
Appendix 7-1 202
Appendix 7-2 204
Appendix 7-3 205
Appendix 8-1 206
Appendix 8-2 208
Appendix 9 214
Appendix 10 215
Appendix 11 216
Appendix 12 218
LIST OF ABBREVIATIONS USED 219
AUTHOR'S TEAM 221

INTRODUCTION

In late 2019, an outbreak of a new coronavirus infection occurred in the People's Republic of China (PRC), with the epicenter in Wuhan (Hubei Province). On February 11, 2020, the World Health Organization (WHO) defined the official name of the infection caused by the new coronavirus as COVID-19 ("Coronavirus disease 2019"). On February 11, 2020, the International Committee on Taxonomy of Viruses assigned the official name to the causative agent of the infection - SARS-CoV-2.

The emergence of COVID-19 has posed challenges for healthcare professionals related to rapid diagnosis and provision of medical care to patients. Currently, intensive study of the clinical and epidemiological features of the disease, development of new means of its prevention and treatment are ongoing. The most common clinical manifestation of the new variant of coronavirus infection is bilateral pneumonia (viral diffuse alveolar damage with microangiopathy), acute

respiratory distress syndrome (ARDS) is registered in 3-4% of patients. Some patients develop hypercoagulation syndrome with thrombosis and thromboembolism, other organs and systems are also affected (central nervous system, myocardium, kidneys, liver, gastrointestinal tract, endocrine and immune systems), sepsis and septic shock may develop.

The recommendations presented in the document are largely based on materials on the diagnosis, prevention and treatment of COVID-19 published by specialists from the WHO, the Chinese, American and European Centers for Disease Control, an analysis of domestic and foreign scientific publications, regulatory documents of the Government of the Russian Federation, the Ministry of Health of Russia and Rospotrebnadzor.

Measures to prevent the import and reduce the risks of the spread of COVID-19 in the territory of the Russian Federation are regulated by the Orders of the Government of the Russian Federation of 30.01.2020 No. 140-r, of 31.01.2020 No. 154-r, of 03.02.2020 No. 194-r, of 18.02.2020 No. 338-r, of 27.02.2020 No. 447-r, of 27.02.2020 No. 446-r, of 27.02.2020 No. 448-r of 16.03.2020 No. 635-r, of 06.03.2020 No. 550-r, of 12.03.2020 No. 597-r, dated 14.03.2020 No. 622-r, dated 16 March 2020 No. 730-r, dated 27 March 2020 No. 763-r, dated 1 August 2020 No. 1996-r, dated 1 August 2020 No. 1997-r, dated 2 September 2020 No. 2236-r, dated 12 September 2020 No. 2338-r, dated 20 September 2020 No. 2406-r, dated 14 October 2020 No. 2649-r and resolutions of the Chief State Sanitary Doctor of the Russian Federation dated 24.01.2020 No. 2, dated 01/31/2020 No. 3, from 03/02/2020 No. 5, from 03/13/2020 No. 6, from 03/18/2020 No. 7, from 03/30/2020 No. 9, from 04/03/2020 No. 10, from 04/13/2020 No. 11, from 05.22.2020 No. 15, dated 07.07.2020 No. 18, dated 07.13.2020 No. 20, dated 07.15.2020 No. 21, dated 07.27.2020 No. 22, dated 09.18.2020 No. 27, from 10.16.2020 No. 31,
from 11/13/2020 No. 34, from 11/13/2020 No. 35, from 12/21/2020 No. 43, from 04/09/2021 No. 12, from 04/16/2021 No. 13, from 07/02/2021 No. 17.

Measures to prevent the spread of COVID-19 in medical organizations are carried out in accordance with the order of the Ministry of Health of Russia dated 19.03.2020 No. 198n "On the temporary procedure for organizing the work of medical organizations in order to implement measures to prevent and reduce the risks of the spread of the new coronavirus infection COVID-19" (as amended by orders of the Ministry of Health of Russia dated 27.03.2020 No. 246n, dated 02.04.2020 No. 264n, dated 29.04.2020 No. 385n, dated 18.05.2020 No. 459n, dated 29.05.2020 No. 513n, dated 07.07.2020 No. 685n, dated 27.08.2020 No. 905n, dated 09/15/2020 No. 982n, dated 10/01/2020 No. 1062n, dated 10/23/2020 No. 1140n, dated 10/30/2020 No. 1184n, dated 12/04/2020 No. 1288n, dated 03/23/2021 No. 232n, dated July 22, 2021 No. 792n).

The guidelines are intended for heads of medical organizations and their structural divisions, general practitioners, general practitioners, infectious disease specialists, pediatricians, obstetricians and gynecologists, intensive care physicians of infectious disease hospital intensive care units, emergency medical care physicians, as well as other specialists working in the field of laboratory and instrumental diagnostics and organization of medical care for patients with COVID-19.

1. ETIOLOGY, PATHOGENESIS AND PATHOMORPHOLOGY

Coronaviruses (Coronaviridae) are a large family of RNA-containing viruses that can infect both animals (their natural hosts) and humans. Based on serological and phylogenetic analysis, coronaviruses are divided into four genera: *Alphacoronavirus* , *Betacoronavirus* , *Gammacoronavirus* and *Deltacoronavirus* . In humans, coronaviruses can cause a range of diseases, from mild acute respiratory infections (ARVI) to severe acute respiratory syndrome (SARS). Currently, four seasonal coronaviruses (HCoV-229E, -OC43, -NL63, and -HKU1) are circulating in the population, which are present in the structure of ARVI all year round and, as a rule, cause mild to moderate damage to the upper respiratory tract, as well as two highly pathogenic coronaviruses - the Middle East respiratory syndrome virus (MERS) and the new coronavirus infection COVID-19.

Until 2002, coronaviruses were considered agents causing mild upper respiratory tract infections (with extremely rare fatal outcomes). Between 2002 and 2004, the SARS-CoV coronavirus from the *Betacoronavirus genus* (reservoir - bats, intermediate reservoir - civets) first caused the development of an epidemic of the so-called atypical pneumonia (SARS) and the confirmed cause of death of 774 people in 37 countries. Since 2004, no new cases of atypical pneumonia caused by SARS-CoV have been registered. Another

An epidemic caused by the MERS-CoV coronavirus (reservoir – dromedary camels), also from the genus Betacoronavirus – Middle East coronavirus syndrome, began in 2012 in the Arabian Peninsula (82% of cases in Saudi Arabia). By 2020, 866 deaths from MERS were registered. At the moment, MERS-CoV continues to circulate and cause new cases of the disease.

SARS-CoV-2 is an enveloped, positive-sense, single-stranded RNA virus belonging to the Coronaviridae family, *Betacoronavirus* genus , *Sarbecovirus* subgenus . For members of the *Coronaviridae family,* Characterized by club-shaped spikes (peplomeres) that look like a crown on the surface of the virus particle when examined using electron microscopy. The genetic sequence of SARS-CoV-2 is at least 79% similar to that of SARS-CoV .

The original strain isolated from samples from patients hospitalized in Wuhan in December 2019 was assigned to the L genetic clade and serves as the reference genome for all subsequent sequences obtained by sequencing. In early January 2020, a slightly altered SARS-CoV-2 strain was assigned to the S clade, and then clade O was also isolated.

SARS-CoV-2 viruses into clades was based on minor mutations in the genome structure that were of no significance for practical healthcare, since they did not change the biological properties of the virus (virulence, contagiousness, sensitivity to antiviral drugs). Subsequently, circulating SARS-CoV-2 strains also continued to accumulate individual point mutations in the genome, which led to the allocation of the following two clades: clade V in mid-January 2020 (with mutations in the NSP6 and ORF3 proteins) and clade G with a characteristic mutation D614G. Clade G also subsequently split (approximately since February 2020) into two subclades GR and GH, the most common in the world.

SARS-CoV-2 variants currently exceeds 1000 different genetic lineages. Most of the

reported SARS-CoV-2 mutations have no functional significance. Only individual lineages have significant epidemic significance. Sequencing data is collected in different databases, which has led to the emergence of several nomenclature systems used worldwide to classify virus variants: PANGO (Phylogenetic Assignment of Named Global Outbreak), GISAID and Nextstrain. Each of the nomenclature systems uses its own designations for groups of virus variants united by a set of mutations contained in their genomes. To facilitate an expanded discussion of the epidemiological and clinical significance of virus variants and to facilitate the exchange of data on the emergence and spread of virus variants, WHO created a Working Group that proposed to unify the designation of groups of virus variants and designate them with letters of the Greek alphabet.

Based on the prevalence of different variants of the virus among the population and data on their biological properties (contagiousness, pathogenicity, relation to the neutralizing activity of antibodies), WHO proposed to distinguish variants of concern (VOC – variant of concern) and variants of interest (VOI – variant of interest).

VOIs are widespread in many countries around the world and have mutations that could potentially alter their biological properties, but there is currently no evidence for this.

VOCs, along with mutations, have biological properties that increase contagiousness, pathogenicity, or reduce the neutralizing activity of antibodies.

To date, the alpha (PANGO B.1.1.7 lineage, first detected in the UK in September 2020), beta (PANGO B.1.351 lineage, first detected in South Africa in May 2020), gamma (PANGO P.1 lineage, first detected in Brazil in November 2020) and delta (PANGO B.1.617.2 lineage, first detected in India in October 2020) variants have been classified as VOC variants. The delta variant was initially classified as a VOI but was reclassified as a VOC in May 2021. The eta, iota, kappa, lambda and mu variants are classified as VOI. Currently, the mu variant has become widespread in a number of countries (Colombia, Ecuador, Chile), but in general it has been detected in only 0.1% of the total number of SARS-CoV-2 strains studied. The designations of virus variants adopted in nomenclature systems are retained in scientific publications.

The delta variant, which has become widespread in many countries around the world, carries mutations in its genome that increase the contagiousness of the virus, mutations that increase the affinity of the S-protein of the virus for ACE-2 and reduce the recognition of viral antigens by post-infection and post-vaccination antibodies.

Considering their high pathogenicity, the SARS-CoV, SARS-CoV-2 and MERS-CoV viruses are classified as pathogenicity group II .

At room temperature (20-25 °C), SARS-CoV-2 can remain viable on various environmental objects in dried form for up to 3 days, in a liquid medium - up to 7 days. The virus remains stable in a wide range of pH values (up to 6 days at a pH of 5 to 9 and up to 2 days at pH 4.0 and pH 11.0). At a temperature of +4 °C, the virus remains stable for more than 14 days. When heated to 37 °C, complete inactivation of the virus occurs within 1 day, at 56 °C - within 45 minutes, at 70 °C - within 5 minutes. The virus is sensitive to ultraviolet irradiation with a dose of at least 25 mJ/cm2 [and] the action of various disinfectants in working concentrations.

The entry point for the pathogen is the epithelium of the upper respiratory tract and the

epithelial cells of the stomach and intestines. The initial stage of infection is penetration of SARS-CoV-2 into target cells that have angiotensin-converting enzyme type II (ACE2) receptors. Cellular transmembrane serine protease type 2 (TSP2) promotes the binding of the virus to ACE2, activating its S protein, which is necessary for SARS-CoV-2 to enter the cell. ACE2 is located in the cytoplasmic membrane of many types of human cells, including alveolar cells type II in the lungs and enterocytes of the small intestine, endothelial cells of the arteries and veins, smooth muscle cells of the arteries, and macrophages. ACE2 and TSP2 are found in the cells of the tissues of the respiratory organs, esophagus, intestines, heart, adrenal glands, bladder, brain, and others.

The nucleocapsid protein of the virus was found in the cytoplasm of epithelial cells of the salivary glands, stomach, duodenum and rectum, urinary tract, lacrimal fluid, sperm, and vaginal secretions. However, the main and rapidly accessible target of SARS-CoV-2 is alveolar cells type II (AT2) of the lungs, which determines the development of diffuse alveolar damage. It is believed that gastroenterocolitis can develop with COVID-19 , since the virus affects the epithelial cells of the stomach, small intestine and colon that have ACE2 receptors. With the development of the infectious process, blood vessels (endothelium), as well as the myocardium, kidneys and other organs, can be affected. Changes in immunocompetent organs have not been sufficiently studied; the possibility of specific damage to lymphocytes with their apoptosis and pyroptosis (which underlies the characteristic and prognostically unfavorable lymphopenia), macrophage hyperactivity syndrome and hemophagocytic syndrome, and neutrophil leukocyte deficiency (as one of the causes of disseminated intravascular coagulation (DIC) syndrome) is discussed.

Based on the ability of SARS-CoV-2 to infect various organs and tissues, the idea of the existence of additional receptors and co-receptors of the virus in addition to ACE2 is put forward. In particular, the role of CD147 and other receptors in SARS-CoV-2 cell invasion is discussed.

It has been established that dissemination of SARS-CoV-2 from the systemic bloodstream or through the ethmoid plate leads to brain damage. Changes in the sense of smell (anosmia) in patients at an early stage of the disease may indicate both damage to the central nervous system by the virus, penetrating primarily through the olfactory nerve, as well as morphologically demonstrated viral damage to the cells of the nasal mucosa.

Pathological examination of lung tissue has not revealed any specific macroscopic signs of COVID-19 , although the morphological picture can be considered characteristic. In observations in which signs of severe respiratory failure sharply predominate, a picture of ARDS ("shock lung" or diffuse alveolar damage) is noted :

severe plethora and diffuse compaction of the lungs, practically indistinguishable from that observed in the "swine" flu A/H1N1pdm (in 2009 and subsequent years), except for the typical SARS-CoV-2 damage to the pulmonary vascular system and severe alveolar-hemorrhagic syndrome. A feature of diffuse alveolar damage in COVID-19 is dyschrony and prolongation with a frequent combination of its two phases - exudative and proliferative. The lungs are enlarged in volume and mass, doughy or dense consistency, slightly airy or airless; varnish-like on the surface, dark red (cherry) color, when pressed, dark red liquid flows from the cut surfaces, with difficulty

squeezed out of the tissue. In addition to hemorrhages of varying sizes, hemorrhagic infarctions, occluding thrombi, mainly in the branches of the pulmonary veins, are encountered. No significant tracheal lesions are observed, the detected serous-purulent exudate and hyperemia of the mucous membrane in intubated patients are associated with nosocomial infection. In cases where COVID-19 was added to another severe pathology, a combination of changes characteristic of different diseases is naturally observed.

The nature of morphological changes in mild COVID-19 is unknown. Based on the analysis of clinical symptoms, it can be assumed that the virus is tropic to the laryngeal epithelium, the ciliated epithelium of the respiratory tract along its entire length, and alveolocytes of types I and II . Apparently, viral lesions in such patients do not lead to the development of severe exudative inflammation and, accordingly, catarrhal phenomena.

The critical form of COVID-19 is a type of cytokine storm, and its manifestations are similar to the course of primary and secondary hemophagocytic lymphohistiocytosis (HLH) or macrophage activation syndrome (MAS). In critical COVID-19, pathological activation of innate and acquired (Th1 and Th17 types) immunity, "dysregulation" of the synthesis of "pro-inflammatory", immunoregulatory, "anti-inflammatory" cytokines and chemokines develops: IL1, IL2, IL6, IL7, IL8, IL9, IL10, IL12, IL17, IL18, granulocyte colony-stimulating factor (G-CSF), granulocyte macrophage colony-stimulating factor (GM-CSF), tumor necrosis factor α (TNFα), IFNγ-inducible protein 10, IFN-α and IFN-β, monocyte chemoattractant protein 1 (MCP1), macrophage inflammatory protein 1α (MIC1α), as well as inflammation markers (CRP, ferritin).

The difference between COVID-19-induced secondary HLH and other forms of virus-induced cytokine storm is that the target organ in this variant of cytokine storm is the lungs, which is associated with the tropism of the coronavirus to lung tissue, as well as a more moderate increase in serum ferritin levels. Hyperactivation of the immune response in COVID-19 is often
is limited to the lung parenchyma, adjacent bronchial and alveolar lymphoid tissue, and is associated with the development of ARDS.

In the early period of COVID-19 pneumonia, normal blood fibrinogen levels, regional fibrinolysis, and high D-dimer levels are observed, which is not a sign of the development of acute macrophage activation syndrome. This process can be regarded as a CAM-like intrapulmonary inflammation that increases the severity of local vascular dysfunction, including microthrombosis and hemorrhage, which leads to the development of pulmonary intravascular coagulopathy to a greater extent than disseminated intravascular coagulation.

Patients with critical COVID-19 develop vascular endothelial dysfunction, coagulopathy, thrombosis with the presence of antibodies to phospholipids, with a clinical picture resembling catastrophic antiphospholipid syndrome. Clinical and pathological changes are difficult to differentiate from multiorgan thrombosis developing with DIC and thrombotic microangiopathy (TMA).

The cytokine storm in COVID-19 usually leads to the development of ARDS, multiple organ failure and can be fatal.

Microscopic examination reveals intraalveolar edema with admixture of erythrocytes,

macrophages, desquamated alveocytes, single neutrophils, lymphocytes and plasma cells in the edema fluid; intraalveolar hyaline membranes, sometimes extending to the inner surface of the bronchioles; desquamation of the alveolar (in the form of individual cells and their layers) and bronchiolar epithelium; the appearance of large, irregularly shaped type II alveocytes with enlarged nuclei with coarse-grained chromatin and distinct nucleoli (in some of them, a halo is visible around the nucleus, and in the cytoplasm - rounded basophilic and eosinophilic inclusions, characteristic of viral cell damage); proliferation of type II alveolocytes, formation of their symplasts. Weakly expressed perivascular and peribronchial lymphoid and macrophage infiltration is characteristic, as well as infiltration of the interalveolar septa and walls of small vessels, represented by various populations of lymphocytes and macrophages. CD3+ T lymphocytes predominate, among them are CD2+, CD5+, CD8+ T cells. B lymphocytes (CD20+, C80+) are relatively few in number, natural killers (CD16+/CD56+) are practically absent. The SARS-CoV-2 virus is detected in ciliated cells of the bronchi, bronchiole epithelium, alveolocytes and macrophages, as well as in the vascular endothelium using nucleic acid amplification methods (NAAT), immunohistochemical, ultrastructural studies.

Specific viral and cytokine storm-induced (and, at a later stage, possibly autoimmune) damage to the endothelium, called SARS-CoV-2-associated endothelial dysfunction and even endotheliitis, and hypercoagulation syndrome are the basis of thrombotic microangiopathy characteristic of COVID-19 , mainly in the lungs, less often in other organs (myocardium, brain, kidneys, etc.) and thrombosis of large arteries and veins (often with thromboembolism). The possibility of platelet activation by antibodies to SARS-CoV2 is not excluded as an important cause of the development of hypercoagulation syndrome. In isolated observations, local pulmonary or systemic productively destructive thrombovasculitis develops, possibly as a result of superinfection. There is evidence of a link between some observations of post-COVID syndrome, along with other pathological processes, with prolonged thrombotic microangiopathy and persistent hypercoagulability syndrome.

In the pathogenesis of COVID-19, damage to the microcirculatory bed plays a critical role. Lung damage in COVID-19 is characterized by pronounced plethora of the capillaries of the interalveolar septa, as well as branches of the pulmonary arteries and veins, with a slowdown in blood flow, with erythrocyte sludge, fresh fibrin and organizing thrombi; intrabronchial, intrabronchiolar and intra-alveolar hemorrhages, which are a substrate for hemoptysis, as well as perivascular hemorrhages. Damage to the vascular bed of the lungs is an important factor in the pathogenesis of hypoxia and ARDS. Severe alveolar-hemorrhagic syndrome is characteristic of most observations, up to the formation of, in fact, hemorrhagic infarctions (although true hemorrhagic infarctions are not uncommon). It is important to distinguish pulmonary vascular thrombi from thromboembolism, since pulmonary embolism (PE) is also characteristic of COVID-19. Pulmonary artery thrombosis sometimes progresses to the right heart; thrombosis of arteries of various organs with the development of their infarctions (myocardium, brain, intestines, kidneys, spleen) has been described; gangrene of the extremities has also been described. This distinguishes the changes in the lungs in COVID-19 from those previously observed in influenza A/H1N1 and

other coronavirus infections. Despite the pronounced alveolar hemorrhagic syndrome, significant hemosiderin deposits are not observed in their outcome. The described lesions of the lungs and other organs are the cause of death without the addition of bacterial or mycotic superinfection. Its frequency does not exceed 30-35%, mainly in patients with prolonged artificial ventilation (ALV).

Some observations also revealed changes in other organs that could presumably be associated with the generalization of coronavirus infection or immune disorders: the intestines (catarrhal and hemorrhagic gastroenterocolitis, ischemic lesions), the brain and soft tissue of the brain.

membrane (encephalitis, meningitis, hypoxic and ischemic lesions), heart (myocarditis, acute coronary syndrome), pancreas, kidneys, spleen, testicles. Direct viral lesions of the placenta are also very likely; isolated observations have shown the possibility of intrauterine infection, the clinical significance of which requires further study. Severe lesions of the microcirculatory bed have been noted, similar to those developing in the lungs in the form of widespread thrombotic microangiopathy, thrombosis of large arteries and veins. Skin manifestations typical of COVID-19 have been described - from hemorrhagic syndrome to rashes of various types, the pathogenesis of which is unclear. There is evidence that SARS-CoV2 is capable of activating previous chronic infectious processes.

Based on studies of autopsy material, taking into account the clinical picture of the disease and the characteristics of thanatogenesis, it is possible to identify at least the following clinical and morphological masks of COVID-19, but with mandatory lung damage: cardiac, cerebral, intestinal, renal, hepatic, diabetic, thromboembolic (with pulmonary embolism), septic (in the absence of bacterial or mycotic sepsis), cutaneous.

Thus, as with other coronavirus infections, as well as influenza A/H1N1, in most cases the main morphological substrate of COVID-19 is diffuse alveolar damage, but, unlike them, with simultaneous severe damage to the vascular bed and in a number of patients of various organs and systems. The term viral (interstitial) pneumonia, widely used in the clinic, essentially reflects the development of diffuse alveolar damage. In turn, severe diffuse alveolar damage is synonymous with the clinical concept of "acute respiratory distress syndrome" (ARDS).

Many aspects of the pathogenesis and pathomorphology of coronavirus infection require further comprehensive study using modern methods.

2. EPIDEMIOLOGICAL CHARACTERISTICS

From December 2019 to March 2020, SARS-CoV-2 was most widely distributed in the PRC, where confirmed cases of the disease were registered in all administrative entities. The largest number of cases was detected in the southeastern part of the PRC with the epicenter in Hubei Province (84% of the total number of cases in the PRC).

Since the end of January 2020, many countries around the world have started to register cases of COVID-19, mainly related to trips to China. At the end of February 2020, the epidemiological situation with COVID-19 in South Korea became much more complicated,
COVID-19 pandemic on 11 March 2020 .

Almost all countries in the world have been seriously affected by the COVID-19 pandemic, but the epidemic situation in different countries is extremely heterogeneous. High levels of morbidity and mortality are observed in those countries where isolation and restrictive measures were introduced late or incompletely (Italy, Spain, USA, Great Britain). On the contrary, in countries where anti-epidemic measures were introduced in a timely manner and in full (Singapore, South Korea, Taiwan, Japan), there is a low level of morbidity and mortality from COVID-19.

The source of infection is a sick person, including one in the incubation period of the disease, and an asymptomatic carrier of SARS-CoV-2. A sick person poses the greatest danger to others in the last two days of the incubation period and the first days of the disease.

The infection is transmitted by airborne droplets, airborne dust and contact. The leading route of transmission of SARS-CoV-2 is airborne droplets, which is realized when coughing, sneezing and talking at a close distance (less than 2 meters). Contact transmission is possible, which is realized during handshakes and other types of direct contact with an infected person, as well as through surfaces and objects contaminated with the virus.

According to available scientific data, the feco-oral mechanism of transmission of the virus is possible. SARS-CoV-2 RNA was detected in fecal samples of patients, as in other infections caused by highly pathogenic coronaviruses.

The role of COVID-19 as an infection associated with the provision of medical care has been established. Healthcare workers are at the highest risk of infection, since they have prolonged aerosol contact in the course of performing their professional duties. The risk of airborne, dust and contact transmission of the pathogen increases in conditions of non-compliance with the requirements of the sanitary and anti-epidemic regime, epidemiological safety rules, including the use of personal protective equipment.

There is a high risk of the formation of epidemic foci of COVID-19 in organized groups (military groups, educational institutions, nursing homes, mental health centers, dormitories, medical organizations) when the infection is brought in, especially in the event of a violation of the sanitary and anti-epidemic regime. There is also a risk of multiple diseases in closed-type organizations due to failure to comply with infection prevention measures.

The new coronavirus infection caused by SARS-CoV-2 has been included in the list of diseases that pose a danger to others (Resolution of the Government of the Russian Federation of January 31, 2020 No. 66).

Standard Case Definition for COVID-19

Suspected COVID-19 case

Clinical manifestations of acute respiratory infection (ARI) (body temperature (t) above 37.5 °C and one or more of the following signs: cough - dry or with scanty sputum, shortness of breath, a feeling of congestion in the chest, blood oxygen saturation according to pulse oximetry (SpO_2) $\leq$ 95%, sore throat, nasal congestion or mild rhinorrhea, impaired or lost sense of smell (hyposmia or anosmia), loss

of taste (dysgeusia), conjunctivitis, weakness, muscle pain, headache, vomiting, diarrhea, skin rash) in the absence of other known causes that explain the clinical picture regardless of the epidemiological history.

Probable (clinically confirmed) case of COVID-19

1. Clinical manifestations of ARI (body temperature > 37.5 °C and one or more of the following: cough, dry or with scanty sputum, shortness of breath, feeling of chest congestion, SpO2 ≤ 95%, sore throat, nasal congestion or moderate rhinorrhea, impaired or loss of smell (hyposmia or anosmia), loss of taste (dysgeusia), conjunctivitis, weakness, muscle pain, headache, vomiting, diarrhea, skin rash) in the presence of at least one of the epidemiological signs:

- Return from a trip abroad within 14 days before symptoms appeared;
- Having had close contact in the last 14 days with a person under surveillance for COVID-19 who subsequently became ill;
- Having had close contact in the last 14 days with a person who has been laboratory-confirmed to have COVID-19;
- Having professional contacts with individuals who have been diagnosed with a suspected or confirmed case of COVID-19.

2. The presence of clinical manifestations specified in paragraph 1, in combination with characteristic changes in the lungs according to computed tomography (CT) data (see Appendix 1 of these recommendations) regardless of the results of a single laboratory test for the presence of SARS-CoV-2 RNA and the epidemiological history.

3. The presence of clinical manifestations (indicated in paragraph 1), in combination with characteristic changes in the lungs according to radiological studies (specified in paragraph 2) if it is impossible to conduct a laboratory study for the presence of SARS-CoV-2 RNA.

Confirmed case of COVID-19

1. A positive laboratory test result for the presence of SARS-CoV-2 RNA using nucleic acid amplification methods (NAAT) or SARS-CoV-2 antigen using immunochromatographic analysis, regardless of clinical manifestations.

4. CLINICAL FEATURES

The incubation period ranges from 2 to 14 days, on average 5-7 days.

COVID-19 is characterized by the presence of clinical symptoms of acute respiratory viral infections:

- Increase in body temperature (> 90%);
- Cough (dry or with a small amount of sputum) in 80% of cases;
- Shortness of breath (30%);

- Fatigue (40%);
- Feeling of tightness in the chest (> 20%).

Sore throat, runny nose, decreased sense of smell and taste, and signs of conjunctivitis may also be noted.

The most severe dyspnea develops by the 6th-8th day from the moment of illness. It has also been established that among the first symptoms may be myalgia (11%), confusion (9%), headaches (8%), hemoptysis (2-3%), diarrhea (3%), nausea, vomiting, palpitations. These symptoms at the beginning of the disease may be observed even in the absence of an increase in body temperature.

Clinical variants and manifestations of COVID-19:

- ARVI (affecting only the upper respiratory tract);
- Pneumonia without respiratory failure;
- ARDS (pneumonia with acute respiratory failure);
- Sepsis, septic (infectious-toxic) shock;
- DIC syndrome, thrombosis and thromboembolism.

Hypoxemia ($SpO2 < 88\%$) develops in more than 30% of patients.

Classification of COVID-19 by severity

Mild flow

- Body temperature < 38 °C, cough, weakness, sore throat
- Lack of criteria for moderate and severe course

Moderately severe course

- Body T > 38 °C
- Respiratory rate > 22/min
- Shortness of breath during physical exertion
- CT (X-ray) changes typical of viral infection
- $SpO2 < 95\%$
- Serum CRP >10 mg/L

Severe course

- Respiratory rate > 30/min
- $SpO2 \leq 93\%$
- $PaO_2/FiO_2 \leq 300$ mm Hg.
- Decreased level of consciousness, agitation
- Unstable hemodynamics (systolic blood pressure less than 90 mmHg or diastolic blood pressure less than 60 mmHg, diuresis less than 20 ml/hour)
- Changes in the lungs on CT (X-ray) typical of viral infection

- Arterial blood lactate > 2 mmol/l
- qSOFA > 2 points

Extremely severe course

- Persistent febrile fever
- ARDS
- ARF requiring respiratory support (invasive ventilation)
- Septic shock
- Multiple organ failure
- Changes in the lungs on CT (X-ray) typical of a critical degree viral infection or a picture of ARDS.

On average, 50% of those infected are asymptomatic.

In 80% of patients with clinical symptoms, the disease occurs in a mild form of acute respiratory viral infection.

Skin rashes in COVID-19

Currently, there are a number of clinical observations describing skin rashes in COVID-19, in connection with which the main task of clinicians is the differential diagnosis of skin lesions in COVID-19 from other infectious exanthemas, as well as a number of dermatoses.

An analysis of the descriptions of clinical observations of skin rashes in patients with COVID-19 accumulating in the literature, as well as our own experience of ongoing dynamic observation of our compatriots suffering from this viral disease, allows us to conclude that skin lesions can be the first signs of the onset of coronavirus infection. In addition, the variety of observed dermatoses and skin rashes can be divided into seven groups depending on their etiology and mechanisms of development:

1 group - Skin angiitis.

As a rule, skin angiitis has an infectious-allergic genesis and occurs against the background of infectious processes of various, including viral, etiologies. A classic example is acute erythema nodosum against the background of a common acute respiratory viral infection. In coronavirus infection, the walls of small vessels of the dermis are affected by circulating immune complexes in the form of deposits with viral antigens. Acrovasculitis can be attributed to special forms associated with COVID-19 . The acral confinement of the rash is possibly due to hypoxia associated with the disease.

2 group - Papulosquamous rashes and pink lichen.

They are characteristic infectious-allergic skin lesions, also often associated with COVID-19. A clinical feature of pink lichen in coronavirus infection is the absence of a "mother plaque" (the largest element that appears first in the classic course of dermatosis).

3 group - Morbilliform rashes and infectious erythema.

In COVID-19, these rashes resemble those typical of measles or other viral infections in their clinical characteristics and, thus, indicate a pathogenetic closeness to classic viral exanthems.

4 group - Papulovesicular eruptions

(like miliaria or eccrine miliaria).

They occur against the background of subfebrile temperature with increased sweating for many days in patients. Unlike classic miliaria, rashes in COVID-19 are characterized by extensive skin lesions.

5 group – Toxicodermia.

They are not directly related to coronavirus infection and are a consequence of individual intolerance of patients to certain medications.

6 group - Nettle.

Depending on its origin, the disease can have a dual nature. On the one hand, urticarial rashes can be a harbinger of the onset of COVID-19 or occur along with its first symptoms. On the other hand, urticaria often develops as a result of drug intolerance and in this case is a clinical form of toxicoderma. The acral location of blisters against the background of COVID-19 can also be attributed to the specific features of urticarial skin lesions in this viral disease.

7 group - Artificial lesions

(trophic changes in facial tissues).

They are a consequence of patients being forced to remain in a prone position for a long time in order to improve respiratory function.

Features of clinical manifestations in elderly and senile patients

Elderly patients may present with an atypical presentation without fever and cough due to decreased reactivity. COVID-19 symptoms may be subtle and out of proportion to the severity of the disease and prognosis. Atypical symptoms of COVID-19 in elderly and senile patients include delirium and delirium. The Brief Confusion Scale is recommended for screening for delirium (Table 1).

Table 1. Brief Confusion Rating Scale

**STAGE 1**	
**Severity and wave-like nature of changes in mental status** :<br>Are there any changes in mental status from baseline?<br>OR<br>Have there been any fluctuating changes in mental status within the past 24 hours?	If the answers to both question "NO" → **NO DELIRIUM**<br><br>To 1 question "YES" → **stage 2**
**STAGE 2**	
**Attention deficit disorder** :<br>"Squeeze my hand every time I say the letter A"<br>"Read the following sequence of letters:<br>L A M P A A L A D I N A<br>ERRORS: does not compress on the letter A and compresses on other letters	0-2 errors →<br>**NO DELIRIUM**<br><br>≥ 2 errors → **stage 3**
**STAGE 3**	
**Changes in the level of consciousness**<br>Current level of consciousness	RASS* not 0 →<br>**DELIRIUM IS THERE**<br>RASS* = 0 → **stage 4**
**STAGE 4**	
**Disorganized thinking** :<br>1. Will the stone float on water?<br>2. Do fish live in the sea?<br>3. Does one kilogram weigh more than two?<br>4. Can you hammer a nail?<br>Team:<br>"Show me as many fingers" (show 2 fingers)<br>"Now do the same with the other hand."<br>(do not demonstrate)<br>OR<br>"Add another finger" (if the patient cannot move both hands)	≥ 2 errors → **DELIRIUM IS PRESENT**<br><br>0-1 error →<br>**NO DELIRIUM**
**Conclusion** : **DELIRIUM / no delirium**	

*** The Richmond Agitation-Sedation Scale (RAS)**

+4 WARRIOR: warlike, aggressive, dangerous to others (immediately inform the doctor about these phenomena)
+3 VERY ARROGATED: aggressive, tries to pull out tubes, IVs or catheters (report to doctor)
+2 AROUSED: frequent aimless movements, resistance to procedures
+1 RESTLESS: anxious, non-aggressive movements
0 CALM AND ATTENTIVE
- 1 SLEEPY: inattentive, sleepy, but always reacts to the voice
- 2 LIGHT SEDATION: wakes up briefly to voice
- 3 MEDIUM SEDATION: movement or opening

4. DIAGNOSTICS OF CORONAVIRUS INFECTION

4.1. PATIENT EXAMINATION ALGORITHM SUSPECTED OF COVID-19

If there are factors indicating a case suspected of COVID-19, patients, regardless of the type of medical care, undergo a comprehensive clinical examination to determine the severity of the condition, including anamnesis, physical examination, examination of diagnostic material

using NAAT, and pulse oximetry.

Based on the results of the clinical examination, the type of medical care and the scope of additional examination are decided. The diagnosis is established based on the clinical examination, epidemiological history data and laboratory test results.

4.1.1. Detailed assessment of all complaints, medical history, epidemiological history.

When collecting an epidemiological history, the presence of foreign trips 14 days before the first symptoms is established, as well as the presence of close contacts over the past 14 days with persons suspected of being infected with SARS-CoV-2, or persons whose diagnosis of COVID-19 has been confirmed by laboratory testing.

4.1.2. Physical examination to determine the severity of the patient's condition, which must include:

- Evaluation of the visible mucous membranes of the upper respiratory tract;
- Auscultation and percussion of the lungs;
- Palpation of the lymph nodes;
- Examination of abdominal organs with determination of the size of the liver and spleen;
- Thermometry;
- Assessment of the level of consciousness;
- Measurement of heart rate, blood pressure, respiratory rate;
- Pulse oximetry with SpO2 measurement to detect respiratory insufficiency and assessment of the severity of hypoxemia.

4.1.3. Instrumental diagnostics

Pulse oximetry with SpO2 measurement for detection of respiratory failure and assessment of hypoxemia severity is a simple and reliable screening method that allows identifying patients with hypoxemia requiring respiratory support and assessing its effectiveness. Dynamic saturation monitoring can be performed using devices
for daily pulse oximetry. For patients with signs of acute respiratory failure (ARF) (SpO2 less than 90%), it is recommended to study arterial blood gases with determination of PaO2 , PaCO2 , pH, bicarbonates, lactate .

Electrocardiography (ECG) in standard leads is recommended for all patients. This study does not contain any specific information, but viral infection and pneumonia increase the risk of developing rhythm disturbances and acute coronary syndrome, the timely detection of which significantly affects the prognosis. In addition, certain changes in the ECG (for example, prolongation of the QT interval) require attention when assessing the cardiotoxicity ~~of a number~~ of antibacterial drugs (respiratory fluoroquinolones, macrolides).

The decision on the need to hospitalize a patient is made by a doctor based on a set of clinical and epidemiological data, taking into account the severity of the patient's condition (moderate/severe course of the disease) and the requirements provided for by the order of the Ministry of Health of Russia dated March 19, 2020 No. 198n "On the temporary procedure for

organizing the work of medical organizations in order to implement measures to prevent and reduce the risks of the spread of the new coronavirus infection COVID-19."

4.2. LABORATORY DIAGNOSTICS OF COVID-19

4.2.1. Laboratory diagnostics etiological:

Direct methods of etiological diagnostics

- Detection of SARS-CoV-2 RNA using NAAT. Instructions for diagnosing COVID-19 using NAAT are presented in Appendix 3-1 .
- Detection of SARS-CoV-2 antigens using immunochromatographic methods. Instructions for detection of SARS-CoV-2 antigens using immunochemical methods are presented in Appendix 3-2.

Indirect methods of etiological diagnostics

- Detection of immunoglobulins of classes A, M, G (IgA, IgM and IgG) to SARS-CoV-2 (including the receptor-binding domain of the surface glycoprotein S). Instructions for diagnosing COVID-19 using immunochemical methods are presented in Appendix 3-2 .

The detection of SARS-CoV-2 RNA using NAAT or coronavirus antigens using immunochemical methods is of primary importance for the etiological laboratory diagnosis of COVID-19 . Diagnostic reagent kits for the detection of SARS-CoV-2 RNA and coronavirus antigens registered in the Russian Federation are presented in State Register of Medical Devices (Appendix 3-4) .

All individuals with signs of ARI are recommended to undergo laboratory testing for SARS-CoV-2 RNA. It is also possible to conduct a laboratory examination using a test to detect SARS-CoV-2 antigens in nasopharyngeal and oropharyngeal swabs using immunochemical methods (Appendix 3-2) . The algorithm for examination using direct etiological methods is given in Appendix 3.3 .

Mandatory laboratory testing to detect SARS-CoV-2 or coronavirus antigens is carried out for the following categories of persons [1]:

- those who arrived in the territory of the Russian Federation with symptoms of an infectious disease (or if symptoms appeared during the period of medical observation);
- those who have been in contact with a patient with COVID-19, if symptoms appear that do not exclude COVID-19;
- Patients diagnosed with community-acquired pneumonia;
- Employees of medical organizations who have a risk of infection during their professional activities,

[1]Resolution of the Chief State Sanitary Doctor of the Russian Federation of March 30, 2020 No. 9 "On additional measures to prevent the spread of COVID-2019"; Resolution of the Chief State Sanitary Doctor of the Russian Federation of May 22, 2020 No. 15 "On approval of sanitary and epidemiological rules SP 3.1.3597-20 "Prevention of a new coronavirus infection (COVID-19)" (as amended on November 13, 2020)

- until IgG appears – once a week [II];
- if symptoms appear that do not exclude COVID-19 – immediately;

- Persons in boarding schools, orphanages, children's camps, boarding houses for the elderly and other stationary social service organizations, penal institutions with the appearance of respiratory symptoms;
- Persons over 65 years of age who seek medical attention with respiratory symptoms;
- Employees of stationary social service organizations, penal institutions and shift workers before starting work in the organization in order to prevent the introduction of COVID-19;
- Children from organized groups when 3 or more cases occur

diseases that do not exclude COVID-19 (they are examined as in the case of an outbreak of diseases).

When contacting medical organizations, patients without signs of ARI are subject to laboratory testing for SARS-CoV-2 RNA if they have the following epidemiological history data:

- The presence of professional contacts with biomaterial from patients with COVID-19 and persons suspected of having this disease (doctors, specialists with higher professional (non-medical) education, middle and junior medical personnel);
- of COVID-19 during labor (within 14 days before delivery) .

Laboratory diagnostics of COVID-19 in the constituent entities of the Russian Federation is carried out in the laboratories of the Centers for Hygiene and Epidemiology of Rospotrebnadzor, in the laboratories of medical organizations (clinical diagnostic, bacteriological, molecular genetic (PCR laboratories) and laboratories of other organizations that have a sanitary and epidemiological conclusion for working with pathogens of pathogenicity groups III-IV using diagnostic methods that do not involve the accumulation of the pathogen, appropriate working conditions and trained personnel. The period for performing the study to detect SARS-CoV-2 RNA by NAAT should not exceed 48 hours from the moment the biological material is received until the person in respect of whom the corresponding study was conducted receives its result [III].

A positive or questionable result obtained in the laboratory of a medical organization is transmitted to the attending physician and to the nearest territorial body of Rospotrebnadzor for immediate implementation of treatment and anti-epidemic measures.

The same material can be sent for re-testing to laboratories of the Centers of Hygiene and Epidemiology, research organizations of Rospotrebnadzor or laboratories of medical organizations

[II]If IgG to SARS-CoV-2 appears as a result of a previous infection or vaccination, further testing for antibodies to the virus is not performed.
[III]Resolution of the Chief State Sanitary Doctor of the Russian Federation of May 22, 2020 No. 15 "On approval of sanitary and epidemiological rules SP 3.1.3597-20 "Prevention of a new coronavirus infection (COVID-19)" (as amended on November 13, 2020)

determined by a joint decision of the territorial body of Rospotrebnadzor and the healthcare department of the constituent entity (Regional of the Federation interdepartmental headquarters) as reference. Confirmation of results

laboratory tests conducted by any organizations, regardless of their organizational and legal form, in Rospotrebnadzor laboratories are carried out selectively [IV]. The term for performing confirmatory tests in a reference laboratory is no more than 48 hours from the moment of sample delivery.

The results obtained in the laboratories of medical organizations that demonstrate stable agreement with the test results in the reference laboratory do not require retesting. Stable agreement is defined as obtaining dynamic convergence of the results of positive (doubtful) samples (85% or more within 10 days), as well as fulfilling the requirements for ensuring biological safety in laboratories in accordance with the existing sanitary and epidemiological conclusion (in accordance with Methodological Recommendations MP 3.1.0169-20 as amended by MP 3.1.0174-20 "Amendments No. 1 to MP 3.1.0170-20 "Laboratory Diagnostics of COVID-19", approved by Rospotrebnadzor on 30.04.2020).

Medical organizations, in accordance with the current sanitary legislation, send an emergency notification (a list certified by a medical organization) to the territorial bodies of Rospotrebnadzor. Registration of COVID-19 patients and inclusion in the reporting forms of Rospotrebnadzor is carried out by the territorial bodies of Rospotrebnadzor only based on the received emergency notifications (lists certified by a medical organization).

Medical organizations that have identified a case of COVID-19 (including a suspicious one) enter information about it into the information resource in accordance with the Decree of the Government of the Russian Federation of March 31, 2020 No. 373 (as amended on June 5, 2020) "On approval of the Temporary rules for recording information in order to prevent the spread of a new coronavirus infection (COVID-19)".

Detection of antibodies to SARS-CoV-2 is of auxiliary importance for the diagnosis of the current infection and is essential for assessing the immune response to the current or past infection. Detection of antibodies to SARS-CoV-2 is carried out using immunochemical methods (Appendix 3-2). The decision to test for antibodies to SARS-CoV-2 is made by the attending physician individually, based on clinical feasibility. Class A antibodies (IgA) begin to form and are available for detection from about the 2nd day from the onset of the disease, reach a peak after 2 weeks and

[IV] Letter of Rospotrebnadzor dated September 18, 2020 No. 02/19400-2020-32 "On the cancellation of confirmation of laboratory test results"

persist for a long time. Class M antibodies (IgM) begin to be detected approximately on the 7th day from the onset of infection, reach a peak after a week and can persist for 2 months or more. Class G antibodies (IgG) to SARS-CoV-2 are detected from about the 3rd week or earlier. A feature of the humoral response to infection is a short time interval between the appearance of IgM and IgG antibodies, and sometimes their simultaneous formation.

To determine the levels of immunoglobulins to SARS-CoV-2, it is necessary to use reagent kits for the quantitative determination of antibodies, and the results of the studies should be presented using the conventional units of measurement BAU/ml (binding antibody units). The units of measurement BAU/ml have been adopted by WHO as an international standard (First WHO International Standard for anti-SARS-CoV-2 Immunoglobulin (Human) (NIBSC code: 20/136)[V]

In accordance with Part 4 of Article 38 of the Federal Law of 21.11.2011 No. 323-FZ "On the Fundamentals of Health Protection of Citizens in the Russian Federation", the circulation of medical products registered in the manner established by the Government of the Russian Federation or the federal executive body authorized by it is permitted on the territory of the Russian Federation (Appendix 3-4) .

4.2.2. General laboratory diagnostics

The volume, timing and frequency of laboratory tests depend on the severity of the disease. Mild disease with outpatient observation does not require additional laboratory tests. In case of hospitalization for moderate, severe and extremely severe disease, the following tests must be performed:

General (clinical) blood test with determination of the level of red blood cells, hemoglobin, hematocrit, leukocytes, platelets, and leukocyte formula.

Blood biochemistry (urea, creatinine, electrolytes, glucose, alanine aminotransferase, aspartate aminotransferase, bilirubin, albumin, lactate, lactate dehydrogenase. Additionally, troponin can be examined as a marker of myocardial damage, and ferritin as an acute phase protein of inflammation, providing information on the severity of the disease and prognosis). Blood biochemistry does not provide any specific information, but the detected deviations may indicate the presence of organ dysfunction, decompensation of concomitant diseases and the development of complications, have a certain prognostic value, affect the choice of drugs and / or their dosage regimen.

C-reactive protein (CRP) is the main laboratory marker of the activity of the process in the lungs. Its increase correlates with the volume of lung tissue damage and is the basis for starting anti-inflammatory therapy.

[V]Letter of Roszdravnadzor dated 05.07.2021 No. 02i-840/21 "On the international format for assessing the level of immunoglobulins, including IgG, to SARS-CoV-2"

Hormonal study: procalcitonin, brain natriuretic peptide - NT-proBNP/BNP. **Procalcitonin** in coronavirus infection with damage to the respiratory parts of the lungs is within the reference values. An increase in procalcitonin indicates the addition of a bacterial infection and correlates with the severity of the course, the prevalence of inflammatory infiltration and the prognosis for bacterial complications.

Coagulogram in volume: activated partial thromboplastin time (APTT), prothrombin time, prothrombin ratio and/or % prothrombin according to Quick, fibrinogen, D-dimer (quantitative method).

4.2.3. Prognostic laboratory markers

Most patients with COVID-19 have normal white blood cell counts, one third have leukopenia, and lymphopenia is present in 83.2% of patients. Thrombocytopenia is moderate, but is more pronounced in severe cases and in people who died from COVID-19.

An increase in D-dimer by 3-4 times the age norm and an increase in prothrombin time, especially in severe cases (decrease in % prothrombin), an increase in fibrinogen have clinical significance. It is necessary to take into account age-related features: D-dimer increases after 50 years due to the accumulation of chronic diseases. The age-related level of the upper limit of the reference interval can be calculated using the formula: age x 0.01 µg / ml (when measured in FEU units). Also, caution should be exercised when studying D-dimer in pregnant women. Pregnancy, even physiologically occurring, is characterized by an increase in D-dimer with a significant spread of values in this group. Outside of SARS-CoV-2 infection , D-dimer is not decisive in tactics and when prescribing low molecular weight heparins. The clinical significance of its increase in COVID-19 in pregnant women has not been finally determined.

The presence of organ dysfunction, decompensation of concomitant diseases and development of complications revealed by biochemical blood analysis have prognostic significance and influence the choice of drugs and/or their dosage regimen. Increased activity of aminotransferases and creatine kinase, concentration of troponin, creatinine or urea are possible.

The level of CRP correlates with the severity of the course, the extent of inflammatory infiltration and the prognosis in pneumonia. The concentration of CRP increased in most patients, simultaneously with an increase in interleukin-6 (IL-6) and ESR to varying degrees. IL-6, IL-10 and TNF-a increase during the disease and decrease during recovery. Patients requiring hospitalization have significantly higher levels of IL-6, IL-10 and TNF-a and a reduced number of CD4 and CD8 T cells. The level of IL-6, IL-10 and tumor necrosis factor -a inversely correlates with the number of CD4 and CD8 associated with lymphopenia. An increase in the acute phase protein ferritin was noted with an unfavorable course of the disease.

In the development of ARDS, the most important for monitoring the condition and assessing the effect of therapy are: IL-6, D -dimer, ferritin, fibrinogen, C- reactive protein, triglycerides, LDH.

Laboratory indicators of progressive macrophage activation syndrome: two- to three-line cytopenia, increased levels of ferritin, CRP, ALT, AST, LDH, hyponatremia, hypofibrinogenemia, decreased levels of antithrombin III, prolongation of prothrombin time and activated partial thromboplastin time.

Hyperinflammation in COVID-19 can manifest as cytopenia (thrombocytopenia and lymphopenia), coagulopathy (thrombocytopenia, hypofibrinogenemia and increased blood D - dimer), tissue damage/hepatitis (increased serum LDH and aminotransferase activity) and macrophage/hepatocyte activation (increased serum ferritin levels).

The level of procalcitonin is important in the diagnosis and prognosis of sepsis: < 0.5 μg/L - low risk of bacterial coinfection and adverse outcome; > 0.5 μg/L - high-risk patients, bacterial coinfection is likely. The procalcitonin test is additional information for early risk assessment and exclusion of bacterial coinfection in patients with COVID-19.

The development of cardiovascular complications in COVID-19 is also accompanied by lymphopenia, thrombocytopenia, increased CRP, MB fraction of creatine kinase, high-sensitivity troponin and brain natriuretic peptide (NT pro-BNP). To identify patients at risk, it is advisable to consider monitoring troponin, monitoring NT-proBNP as a marker of myocardial stress. Patients with increasing dyspnea and NT-proBNP ≥2000 pg/ml were at the highest risk, while those with values of 400 ≤ NT-proBNP < 2000 pg/ml were in the intermediate risk group.

The required volume of laboratory and instrumental examination depending on the clinical manifestations of the disease is presented in Appendix 2-1 . Laboratory monitoring of patients with COVID-19 or suspected of having COVID-19, depending on the severity of the condition, is presented in Appendix 2-2 .

4.2.4. Diagnosis of invasive aspergillosis and invasive candidiasis in patients with COVID-19

The main life-threatening mycoses in patients with COVID-19 are invasive aspergillosis (COVID-IA) and invasive candidiasis (COVID-IC), others (mucormycosis, etc.) are much less common. COVID-IA and COVID-IC occur mainly in patients in intensive care units (ICUs).

The incidence of COVID-IA in patients in the intensive care unit is 2.5-10%, with the use of mechanical ventilation - 18-35%. With the development of COVID-IA, mortality increases by 16-25%, without treatment all patients die. The occurrence of COVID-IA leads to an increase in the duration of treatment in the intensive care unit by 10-37 days. The main pathogens of COVID-IA (*Aspergillus fumigatus* , *A. flavus* and *A.niger*) are susceptible *in vitro* to voriconazole, isavuconazole, caspofungin and amphotericin B (AmB), resistant to fluconazole.

COVID-IA usually occurs 4-15 days after admission to the intensive care unit or 3-8 days after the start of mechanical ventilation. Risk factors for the development of COVID-IA: mechanical ventilation, ARDS, use of steroids and immunosuppressants, lymphocytopenia $\leq 1.0 \times 10^{9}$/l, neutropenia $\leq 0.5 \times 10^{9}$/l, decompensated diabetes mellitus, COPD, malignant tumors. COVID-IA more often develops in older men. The main clinical variant of COVID-IA is pneumonia, less often - ulcerative tracheobronchitis, later hematogenous dissemination with damage to the central nervous system and other organs occurs. Clinical signs of COVID-IA are nonspecific.

In patients with risk factors, COVID-IA should be excluded in the presence of fever refractory to adequate antibacterial therapy for ≥3 days or a new episode of fever after normalization of body temperature for ≥2 days, worsening respiratory failure, as well as the appearance of hemoptysis, chest pain or pleural friction rub. In COVID-IA, bilateral or unilateral foci of consolidation or destruction are detected on CT or RG of the lungs. In the presence of the above

In the presence of clinical and radiological signs of COVID-IA, bronchoscopy is indicated with obtaining BAL from the affected segment; during intubation of the patient, repeated obtaining of tracheal aspirate (TA) or non-bronchoscopic lavage (NBL) is possible.

Microbiologic testing of BAL, NBL, or TA should include a galactomannan (GM) test (Platelia *Aspergillus* , Bio-Rad), microscopy with calcofluor white staining, and Sabouraud culture. In non-neutropenic patients, including patients with COVID-IA, the serum GM test has high specificity but low sensitivity. The diagnosis of COVID-IA is established in the presence of clinical and radiologic features, a positive GM test (serum - optical density index (ODI) > 0.5, BAL - ODI > 1.0, NBL or TA - ODI > 1.0 on repeat determination), microscopy and culture of BAL, NBL, or TA.

The incidence of COVID-IC in patients in the ICU is 0.7-5%. With the treatment of COVID-IC, the mortality rate is 50%; without treatment, all patients die. The occurrence of COVID-IC leads to an increase in the duration of treatment in the ICU by 5-14 days. The main pathogens of COVID-IC (*Candida albicans* , *C. parapsilosis* , *C. auris, C.glabrata* and *C. krusei*) are susceptible *in vitro* to echinocandins (anidulafungin, caspofungin and micafungin). Species *C. albicans* usually sensitive to fluconazole, and non *-albicans Candida species* often resistant. Species *C. auris* resistant to fluconazole and may be resistant to voriconazole, AmB and echinocandins.

Risk factors for the development of COVID-IC in patients in the intensive care unit: long-term use of a central venous catheter (CVC) and antibacterial drugs, severe condition of the patient, mechanical ventilation, use of corticosteroids and immunosuppressants, total parenteral nutrition, hemodialysis. COVID-IC more often develops in older men. The main clinical variants of COVID-IC are candidemia (circulation of *Candida* spp . in the bloodstream) and acute disseminated candidiasis (ADC - candidemia in combination with a focus/foci of dissemination or multiple foci of dissemination). Clinical signs of COVID-IC are nonspecific and do not differ from the symptoms of bacterial sepsis: refractory to the use of antibacterial drugs, an increase in body temperature > 38 ° C, multiple organ failure syndrome, DIC and septic shock. With COVID-IC, almost all organs and tissues can be affected, but most often the skin and subcutaneous tissue, central nervous system, kidneys, heart, lungs and organs of vision are involved in the pathological process.

The diagnosis of COVID-IC is based on the isolation of Candida spp. from blood and other normally sterile substrates (CSF, biopsy, etc.). The standard method for diagnosing COVID-IC is blood culture (it has insufficient diagnostic sensitivity). Increasing the blood volume (≥40 ml per day for an adult patient) during sowing increases the efficiency of COVID-IC diagnostics. The average time for detecting Candida spp. during blood sowing ranges from 18 hours to 3 days and can reach 8 days for C. glabrata. When isolating Candida spp. from normally sterile biosubstrates, it is necessary to determine the type of COVID-IC pathogen and its sensitivity in vitro using a standard method. Reliable PCR methods for diagnosing invasive aspergillosis and invasive candidiasis have not yet been developed.

4.3. RADIATION DIAGNOSTICS OF COVID-19

Radiation diagnostic methods are used to identify COVID-19 pneumonia, its complications, differential diagnostics with other lung diseases, as well as to determine the severity and dynamics of changes, and assess the effectiveness of therapy.

Radiographic methods are also necessary for the detection and assessment of the nature of pathological changes in other anatomical areas and as a means of control for invasive

(interventional) medical interventions.

Methods of radiological diagnostics of chest pathology in patients with suspected/established COVID-19 pneumonia include:

- General chest radiography (RG),
- Computed tomography of the lungs (CT),
- Ultrasound examination of the lungs and pleural cavities (ultrasound).

Standard RG has low sensitivity in detecting initial changes in the first days of the disease and cannot be used for early diagnostics. The information content of RG increases with the duration of pneumonia. Radiography using mobile (ward) devices is the main method of radiation diagnostics of chest pathology in the intensive care unit. The use of a mobile (ward) device is also justified for conducting conventional RG studies in the X-ray room. In hospital conditions, the relative advantage of RG compared to CT is its high throughput. The method allows for confident detection of severe forms of pneumonia and pulmonary edema of various origins, which require hospitalization, including referral to the intensive care unit.

CT has a high sensitivity in detecting lung changes characteristic of COVID-19. CT is advisable for the initial assessment of the chest condition in patients with severe progressive forms of the disease, as well as for differential diagnosis of the identified changes and assessment of the dynamics of the process. CT allows identifying characteristic changes in the lungs in patients with COVID-19 even before positive laboratory tests for infection using NAAT. At the same time, CT reveals lung changes in a significant number of patients with asymptomatic and mild forms of the disease who do not require hospitalization. CT results in these cases do not affect the treatment tactics and prognosis of the disease in the presence of laboratory confirmation of COVID-19. Therefore, the widespread use of CT for screening asymptomatic and mild forms of the disease is not recommended. When a patient with suspected COVID-19 initially seeks medical attention, it is recommended to prescribe CT only if there are clinical and instrumental signs of respiratory failure ($SpO_2 < 95\%$, RR> 22).

The limitations of CT compared to RG are the lower availability of the technology in certain medical organizations, cities and regions; the inaccessibility of the study for some patients on mechanical ventilation; and the high need for CT studies to diagnose other diseases.

At the same time, a comprehensive assessment of anamnestic, clinical and radiological data allows us to determine a clinically confirmed case of COVID-19, route the patient and initiate antiviral therapy.

In this regard, CT can be a “first-line” examination in those medical organizations/territories that have a sufficient number of devices and personnel to perform the required volume of examinations without compromising the timely diagnosis of other diseases (oncological, neurological, etc.) in patients who most need this examination.

ultrasound in patients with suspected/known COVID-19 pneumonia is an additional imaging method that does not replace or exclude X-ray and CT. When the correct technique is

followed, the correct indications are selected, and trained medical personnel are available, this study is highly sensitive in detecting interstitial changes and consolidations in the lung tissue, but only if they are located subpleurally. Ultrasound data do not allow one to clearly determine the cause and/or actual prevalence of changes in the lung tissue.

It should be taken into account that ultrasound is not a standard procedure in the diagnosis of pneumonia, it is not included in clinical guidelines and standards of medical care for the diagnosis and treatment of community-acquired pneumonia. In this regard, the effectiveness of the studies largely depends on the experience and qualifications of the doctor conducting the study.

Recommendations

1. It is recommended to select imaging methods for known/suspected COVID-19 infection differentially, in accordance with the available equipment and human resources of the medical organization, as well as the structure and number of patients examined.

2. The use of radiological diagnostic methods is not recommended in the absence of symptoms of ARI in patients with positive results for SARS-CoV-2 RNA, as well as in the presence of epidemiological data indicating the possibility of infection.

Comments: The currently available clinical guidelines indicate that the use of X-ray, CT and ultrasound for screening (detection of pathology in the absence of clinical symptoms) of community-acquired pneumonia in general and COVID-19 in particular is inappropriate.

3. The use of radiation methods in patients with mild acute respiratory viral infection symptoms and a stable patient's condition is possible only for specific clinical indications, including the presence of risk factors, provided that there are sufficient technical and organizational capabilities. The method of choice in this case is CT of the lungs according to a standard protocol without intravenous contrast or RG with limited availability of CT. The use of ultrasound in these cases is inappropriate. The use of CT examination earlier than 3-5 days from the onset of symptoms of the disease, as well as in the absence of clinical manifestations of damage to the bronchopulmonary system is inappropriate. CT is advisable in the presence of clinical and instrumental signs of respiratory failure (SpO_2 <95%, RR> 22), or in differential diagnosis with another disease.

4. All signs revealed by radiological examinations, including CT symptoms, are not specific to any type of infection and do not allow establishing an etiological diagnosis. Outside of a clinical (epidemic) situation, they do not allow attributing the identified changes to COVID-19 pneumonia and differentiating them from other pneumonias and non-inflammatory diseases. Radiological examination data do not replace the results of the SARS-CoV-2 RNA examination . The absence of changes in CT does not exclude the presence of COVID-19 and the possibility of pneumonia developing after the examination.

5. It is recommended to conduct a radiological examination for patients with moderate, severe and extremely severe ARI for the purpose of medical triage, assessment of the nature of changes in the chest cavity and determination of the prognosis of the disease:

- performing CT scan of the lungs without intravenous contrast in a hospital setting or on

an outpatient basis - if hospitalization is indicated;

- performing lung X-ray in two projections if CT scanning is not possible in a given medical organization/clinical situation.

6. Intravenous contrast in CT in patients with known/suspected viral (COVID-19) pneumonia is used when there is a suspicion of diseases and pathological conditions, the diagnosis of which is impossible without the use of contrast agents (PE, cancer, etc.). A sudden increase in the concentration of D-dimer in blood tests and clinical suspicion of PE are important criteria for performing CT pulmonary angiograpgy, provided that its positive result can affect the treatment and management of the patient.

7. The decision on intravenous contrast is made by the radiologist together with the doctor referring the patient for CT. The contrast agent is administered in accordance with the general rules for conducting X-ray contrast studies.

8. It is recommended to conduct a radiological examination for patients with severe and extremely severe ARI requiring treatment in an intensive care unit:

- optimal: performing an emergency CT scan of the lungs according to the standard protocol without intravenous contrast, if it is possible to transport the patient to the CT room before the intensive care unit;

- optimal: performing emergency lung RG in the intensive care unit using a mobile (ward) apparatus;

- possible: performing ultrasound of the lungs and pleural cavities according to clinical indications in addition to X-ray or CT.

Comments.

1. The use of ultrasound of the lungs as an additional study is possible only if there are technical capabilities and trained personnel with experience in conducting lung studies. Ultrasound data complement, but do not replace RG and CT of the lungs. Detailed information on the use of ultrasound of the lungs for COVID-19 pneumonia is posted on the website http://www.rasudm.org and in Appendix 1 .

2. The use of MRI of the lungs for the diagnosis of pneumonia caused by COVID-19 is not recommended for practical healthcare. This method can be used in exceptional cases to assess the condition of the lungs when CT is unavailable (breakdown of the only device) and the results of X-ray are uncertain, as well as in the presence of doctors with experience in performing and interpreting such studies. The use of MRI for studies of other organs and systems (brain, spine, etc.) in patients with COVID-19 is carried out according to vital indications, in rooms specially allocated for this purpose, observing all methods of protecting the personnel of the radiation diagnostics departments. It is also possible to split the MRI operating time, when shifts (time intervals) are allocated for separate examination of infected and uninfected patients. This operating mode requires thorough disinfection of the room before the start of the examination of uninfected patients in accordance with the established rules.

9. COVID -19 pneumonia is assessed according to clinical indications using the following visualization methods:

- optimal: performing a CT scan of the lungs according to a standard protocol without intravenous contrast;
- possible: X-ray in two projections in the X-ray room;
- possible: performing an ultrasound of the lungs (as an additional study) if it is impossible to assess the dynamics using CT and RG, provided that there is initial information about the true volume and cause of lung damage and trained medical

personnel.

Comments. The frequency of CT, RG or ultrasound examinations depends on the clinical indications dictating the need to assess the dynamics. The recommended frequency of repetition for CT and RG is no more than once every 7 days .

An objective assessment of the dynamics is possible only when comparing data from one type of study, for example, CT or RG. Comparison of the visual picture of pneumonia using different visualization methods is difficult and, as a rule, subjective. It is possible to use ultrasound of the lungs, but only if there is (1) initial information about the true volume and cause of lung damage and (2) trained medical personnel.

10. Assessment of the dynamics of COVID-19 pneumonia in the intensive care unit is carried out according to clinical indications:

- optimal: performing CT scan of the lungs when transportation is possible patients in the CT room or using a mobile CT machine;
- optimal: performing lung RG with the possibility of transportation patients in the X-ray room;
- possible: performing RG with the help of a mobile (ward) X-ray machine;
- possible: performing an ultrasound of the lungs (as an additional study) when it is impossible to assess the dynamics using CT and RG, provided that there is initial information about the true volume and cause of lung damage and trained medical personnel.

Comments: Limited information content should be taken into account X-ray in the intensive care unit using a mobile (ward) X-ray machine, including due to the usual practice of examination in one direct projection. Ultrasound of the lungs and pleural cavities may be an additional examination if CT cannot be performed, which is performed according to clinical indications and if there is personnel with experience in conducting and interpreting such examinations.

11. It is recommended to use special measures to limit radiation doses when examining pregnant women, newborns and young children if they may develop COVID-19 pneumonia.

Comments. Examination of pregnant women with known/suspected COVID-19 pneumonia is carried out using standard X-ray and CT techniques. It is necessary to use preset programs to limit radiation doses, protection of radiosensitive organs and the fetus (abdominal and pelvic areas) is required using standard protective equipment (aprons, collars), available in the offices. If CT and RG are not possible or are refused, ultrasound of the lungs is used if trained medical personnel are available.

The examination of newborns and young children with known/suspected COVID-19 pneumonia , if possible, begins with the use of ultrasound of the lungs, pleural cavities and mediastinum, and if there are clinical indications, continues with the use of X-ray and/or CT of the chest.

12. If there are clinical indications, all of the above methods of radiation diagnostics can be used to assess the condition of other anatomical areas and individual organs in order to identify pathological changes and assess their dynamics.

13. All radiation studies must ensure epidemiological safety and protection of personnel and patients in accordance with the temporary recommendations of the Russian Ministry of Health for the operation of medical institutions during the COVID-19 epidemic.

Comments. The main components of personnel protection are zoning of radiation diagnostic rooms, limiting contacts between flows of potentially infected and uninfected patients, limiting contacts of department/radiology diagnostic room personnel, shift work of personnel, and mandatory use of personal protective equipment.

Cleaning and disinfection of the premises of the RG, CT and ultrasound rooms, sterilization of equipment and furniture of the rooms are carried out in accordance with these temporary methodological recommendations.

In the X-ray, CT and ultrasound rooms, the rooms are periodically treated with disinfectants to disinfect surfaces. In order to quickly and effectively disinfect air and surfaces in the absence of people, the rooms are periodically treated using open-type ultraviolet bactericidal irradiators with a UV dose of at least 25 mJ/cm2 [and] *a total bactericidal radiation flux of at least 100 W. Bactericidal installations with open irradiators can be used in a repeated short-term mode when people are removed from the room for the duration of irradiation. In this case, repeated irradiation sessions should be carried out every 2 hours during the working day* [VI].

14. The work of offices/departments of radiation diagnostics is organized around the clock (shifts of 6, 12 or 24 hours per day depending on the staffing schedule and workload of a specific medical institution). It is recommended to create a system of remote description of images by on-duty radiologists in medical organizations .

15. Recommendations for the formation of descriptions and assessment of changes in the lungs and chest X-ray in existing/suspected COVID-19 pneumonia are presented in Appendix 1 .

16. To diagnose deep vein thrombosis, it is recommended to perform ultrasound examination of the vessels of the lower extremities with Dopplerography.

17. . DIFFERENTIAL DIAGNOSIS OF COVID-19

It is necessary to differentiate the new coronavirus infection from influenza, acute respiratory viral infections caused by rhinoviruses, adenoviruses, RS virus, human metapneumoviruses, MERS-CoV, parainfluenza virus, viral gastroenteritis, bacterial pathogens of respiratory infections, tuberculosis.

The incubation period of COVID-19 can range from 2 to 14 days, but on average it is 5-7 days, while the incubation period of influenza and ARVI usually does not exceed 3 days. With influenza, the disease begins abruptly, with COVID-19 and ARVI, as a rule, gradually. Both COVID-19 and influenza can be accompanied by high fever, cough, and weakness. High fever and weakness are rare with ARVI. At the same time, with influenza and ARVI, shortness of breath and difficulty breathing are observed much less often than with COVID-19.

Symptoms of COVID-19 and tuberculosis can be similar (cough, fever, weakness). In tuberculosis, symptoms in most cases develop gradually, but there may also be an acute course of the disease. Both diseases can occur simultaneously, aggravating the course of each other. To exclude tuberculosis, it is advisable to conduct an in vitro test to detect effector T cells that respond to stimulation with the *Mycobacterium tuberculosis* antigen ELISPOT method , 3-fold sputum examination for the presence of acid-fast bacteria (after receiving a negative result for the presence

[VI]Guideline R3.5.1904-04 "Use of ultraviolet bactericidal radiation for indoor air disinfection"

of SARS-CoV-2 RNA). If a negative result is received from the microscopic examination of sputum, DNA markers *of Mycobacterium tuberculosis* are detected PCR method, radiological examination.

In viral gastroenteritis, the leading cause is damage to the gastrointestinal tract (GIT); symptoms of damage to the respiratory tract are usually minimal.

When conducting differential diagnostics, it is necessary to take into account the epidemiological anamnesis data, clinical symptoms and their dynamics. In all suspicious cases, testing for SARS-COV-2 and pathogens of other respiratory infections using NAAT is indicated: influenza viruses type A and B, parainfluenza, respiratory syncytial virus, rhinoviruses, adenoviruses,
human metapneumoviruses. It is also recommended to conduct microbiological diagnostics (culture study) and/or PCR diagnostics for Streptococcus pneumoniae, Haemophilus influenzae type B, Legionella pneumophila, as well as other pathogens of bacterial respiratory infections of the lower respiratory tract. For express diagnostics, express tests can be used to detect pneumococcal and legionella antigenuria.

5. TREATMENT OF CORONAVIRUS INFECTION

COVID-19 therapy should be preemptive treatment before the development of a full symptom complex of life-threatening conditions, namely pneumonia, ARDS, sepsis.

Treatment of COVID-19 in accordance with the protocols of these guidelines is carried out in confirmed and probable cases of the disease.

As part of the provision of medical care, it is necessary to monitor the patient's condition to identify signs of deterioration in his or her clinical condition. Patients infected with SARS-CoV-2 should receive supportive pathogenetic and symptomatic therapy.

Treatment of concomitant diseases and complications is carried out in accordance with clinical recommendations and standards of medical care for these diseases.

5.1. ETIOTROPIC TREATMENT

Currently, several drugs that can be used in the treatment of COVID-19 should be highlighted: favipiravir, remdesivir, umifenovir and interferon-alpha.

Favipiravir is a synthetic antiviral drug, a selective RNA polymerase inhibitor, active against RNA-containing viruses. A number of studies have demonstrated that favipiravir effectively inhibits SARS-CoV-2 in cell culture. In January 2020, an open, non-randomized study of favipiravir in the treatment of COVID-19 was conducted in China in comparison with a combination of lopinavir + ritonavir. In the favipiravir group, the median time to virus elimination was 4 days and was significantly less than in the comparison group (11 days). Moreover, by the tenth day, virus elimination was recorded in 80% of patients receiving favipiravir. Improvement according to chest CT data 14 days after the end of the study therapy was recorded in 91.4% of patients in the favipiravir group and only in 62.2% of patients in the control group. Adverse reactions in the favipiravir group were observed 5 times less frequently than in the comparison

group.

A clinical trial to evaluate the efficacy and safety of favipiravir was conducted in the Russian Federation involving 168 patients with mild to moderate COVID-19 (outpatient to inpatient ratio 4:1). The frequency of achieving clinical improvement on day 7 of therapy in the group of patients receiving favipiravir was almost 1.5 times higher than in the comparison group. The proportion of patients with virus elimination on days 3 and 5 of therapy was higher in the favipiravir treatment group, indicating an earlier onset of elimination when using the drug. In the cohort of inpatients receiving favipiravir, by day 28 of therapy, a clinically significant difference was found in the number of resolved cases according to chest CT.

The data obtained allow the use of the drug not only in hospitals, but also in outpatient settings.

Favipiravir dosing regimens are provided in Appendix 5 .

Another drug with antiviral activity against SARS-CoV-2 is remdesivir. Its active metabolite acts as an adenosine triphosphate (ATP) analogue and competes with the natural ATP substrate for incorporation into nascent RNA chains by the RNA-dependent RNA polymerase of the SARS-CoV-2 virus, resulting in delayed chain termination during viral RNA replication. One large study involving 1,063 patients showed that patients receiving a 10-day course of remdesivir had a 4-day shorter recovery time compared to the placebo group (11 and 15 days, respectively). No significant difference in mortality was found in the described groups. According to another phase 3 clinical trial (NCT04292899) and an analysis of a retrospective cohort of patients, treatment with remdesivir was associated with a higher rate of recovery (74.4% vs. 59%) and a 62% reduction in the risk of death (7.6% vs. 12.5%) at day 14 compared with standard therapy.

The drug may only be used in a medical organization providing medical care in a hospital setting. Given the favorable safety profile of the drug (the frequency of adverse events in patients receiving remdesivir in clinical trials did not differ from that in the standard therapy group), as well as data on its effectiveness, remdesivir can be considered as the drug of choice for patients with increased transaminase activity at the start of treatment and for patients at high risk of severe disease: patients over 65 years of age, patients with concomitant diseases (diabetes mellitus, obesity, chronic cardiovascular diseases).

Recombinant interferon alpha (IFN-α) for intranasal administration has immunomodulatory, anti-inflammatory and antiviral effects. The mechanism of action is based on preventing the replication of viruses that enter the body through the respiratory tract. Pregnant women are prescribed only recombinant IFN-α2b.

Etiotropic treatment of COVID-19, especially in patients with concomitant pathology, requires attention to possible drug interactions. Medicines that are prohibited or undesirable to take with etiotropic therapy of COVID-19 can be found on the website https://www.covid19-druginteractions.org/ . Drug interactions between antithrombotics and drugs used to treat patients with COVID-19 are listed in Appendix 4 .

Taking into account the similarity of the clinical picture of mild forms of COVID-19 with the clinical picture of seasonal ARVI, until the etiological diagnosis is confirmed, the treatment

regimens should include drugs recommended for the treatment of seasonal ARVI, such as intranasal forms of IFN-α, IFN inducer drugs, as well as broad-spectrum antiviral drugs such as umifenovir.

According to modern concepts of the pathogenesis of COVID-19, it is advisable to start using drugs recommended for etiotropic therapy early, no later than 7-8 days from the onset of the disease (the appearance of the first symptoms).

The list of possible drugs for the etiotropic therapy of COVID-19 in adults is given in Appendix 5. Recommended treatment regimens depending on the severity of the disease are given in Appendix 8-1 And 8-2 . The prescription of treatment must necessarily be accompanied by obtaining the voluntary informed consent of the patient (or his legal representative).

According to WHO recommendations, it is possible to prescribe drugs with the supposed etiotropic efficacy "off-label" (i.e. use for medical purposes does not comply with the instructions for medical use), while their prescription must comply with the ethical standards recommended by WHO and be carried out on the basis of the Federal Law of November 21, 2011 No. 323-FZ "On the Fundamentals of Health Protection of Citizens in the Russian Federation", the Federal Law of April 12, 2010 No. 61-FZ "On the Circulation of Medicines", the National Standard of the Russian Federation GOST R ISO 14155-2014 "Good Clinical Practice", the order of the Ministry of Health of the Russian Federation dated April 1, 2016 No. 200n "On Approval of the Rules of Good Clinical Practice" (registered by the Ministry of Justice of the Russian Federation on August 23, 2016, registration No. 43357), the Helsinki Declaration of the World Medical Association (WMA) on the ethical principles for conducting research involving human subjects, declared at the 64th General Assembly of the WMA, Fortaleza, Brazil, 2013.

The above-mentioned practice of assessing the appropriateness of using drugs outside the indications specified in the instructions for medical use is generally recognized in the world. In the current conditions of the spread of the new coronavirus infection and the limited evidence base for the treatment of COVID-19, the use of drugs in the "off-label" mode to provide medical care to patients with COVID-19 is based on international recommendations, as well as agreed expert opinions based on an assessment of the degree of benefit and risk when using therapy in the "off-label" mode.

Information on cases of side effects not specified in the instructions for use of medicinal products, on serious adverse reactions, unexpected adverse reactions when using medicinal products, on the specifics of interactions of medicinal products with other medicinal products should be collected and transmitted in the prescribed manner to Roszdravnadzor.

Clinical use of anti-covid plasma

According to WHO recommendations, the use of plasma from convalescent donors (individuals with a confirmed case of COVID-19 in the recovery stage) for the treatment of diseases characterized by epidemic outbreaks and the absence of specific treatment is based on the concept of passive immunization. According to published data, plasma obtained from COVID-19 convalescent donors (hereinafter referred to as anti-COVID plasma) was used in China and other countries, which was reflected in national guidelines for the treatment of COVID-19.

The procurement , storage, transportation and clinical use of anti-COVID plasma is carried out in accordance with the requirements established by the Decree of the Government of the Russian Federation of June 22, 2019 No. 797 "On approval of the Rules for the procurement, storage, transportation and clinical use of donor blood and its components and on recognizing as invalid certain acts of the Government of the Russian Federation", by the order of the Ministry of Health of the Russian Federation of October 28, 2020 No. 183n 1166n "On approval of the procedure for donors to undergo a medical examination and a list of medical contraindications (temporary and permanent) for donating blood and (or) its components and the terms of exemption to which a person is subject in the presence of temporary medical indications from donating blood and (or) its components."

In addition to the established mandatory requirements, donors are selected based on the results of a preliminary study for the presence of IgG to SARS-CoV-2, including rapid blood testing. Additional requirements for anti-COVID plasma donors:

- Age 18-55 years;
- Body weight over 50 kg;
- Total blood protein concentration is not less than 65 g/l;
- Not earlier than 14 days after the disappearance of clinical symptoms
  - negative result of testing for SARS-CoV-2 RNA in an oropharyngeal swab;
- Virus-neutralizing activity of plasma at a dilution of 1:160; in the absence of donors with optimal virus-neutralizing activity of plasma, it is permissible to procure it from donors with virus-neutralizing activity of plasma of 1:80 [VII].

In the absence of a history of an established diagnosis of a new coronavirus infection, donors are selected based on the detection of IgG to SARS-CoV-2. The study to detect IgG to SARS-CoV-2 is carried out using an enzyme-linked immunosorbent assay (immunochemiluminescent immunoassay) test system registered in accordance with the established procedure and approved for use in the territory of the Russian Federation, for which a correlation has been experimentally established between the results of determining the content of antibodies in plasma with virus-neutralizing activity determined in a test using a permissive cell culture.

For this purpose , it is possible to use enzyme immunoassay. (immunochemiluminescent) analysis of IgG to the S1 domain of the Spike glycoprotein of the SARS-CoV-2 virus. According to M. Joyner, 2020, a prospective study (n = 35,322 patients) showed that the effect of anti-COVID plasma transfusion increased as the titer of IgG to the S1 domain of the Spike glycoprotein increased.

It is possible to store a control sample of donor plasma for studies of its virus-neutralizing activity at a later date.

If minor changes are detected during the laboratory examination of the donor, the decision to allow donation is made by the transfusion physician in agreement with the head of the

[VII] Recommendations for Investigational COVID-19 Convalescent Plasma. FDA.2020.

department.

Data on indications for the clinical use of anti-COVID plasma, information on the content of IgG to SARS-CoV-2 in the blood component, the results of the transfusion performed, and the results of the transfusion are recorded in the information resource for recording patients with COVID-19.

Anti-Covid plasma preparation:

- It is carried out by the plasmapheresis method and (or) by the centrifugation method from a unit of blood;
- The donation volume is determined by the transfusion specialist and is 200-600 ml.
- Pathogen reduction of anti-COVID plasma that has not undergone quarantine is carried out using additional processing methods with methylene blue and visible light irradiation or using amotosalen with ultraviolet light irradiation; if the above-mentioned additional processing methods are unavailable, the pathogen reduction method using riboflavin with ultraviolet light irradiation is allowed;
- The interval between donations must be at least 14 days.

Anti-Covid plasma labeling:

- The component containing IgG to SARS-CoV-2 is labeled in accordance with the RF Government Resolution of 22.06.2019 No. 797 “On approval of the Rules for the procurement, storage, transportation and clinical use of donor blood and its components and on the recognition of certain acts of the Government of the Russian Federation as invalid” as “Plasma pathogen-reduced" or "Fresh frozen plasma" with the additional feature "Anticoronavirus".
- When labeling, information is provided on the value of the titer of virus-neutralizing antibodies (if determined) or on the content of IgG to the S1 domain of the Spike glycoprotein of the SARS CoV-2 virus.

Indications for clinical use of anti-COVID plasma:

1. The optimal period for administration is up to 10 days from the onset of clinical symptoms of the disease in patients:

- in serious condition, with a positive laboratory test result for SARS-CoV-2 RNA;
- with moderate severity with manifestations of ARDS.

2. In case of illness duration more than 21 days, if the treatment is ineffective and the result is positive for SARS-CoV-2 RNA.

The total transfusion volume is 5-10 ml of anti-COVID plasma/kg of patient weight, on average 400-600 ml. To increase clinical effectiveness, it is recommended to use 2 transfusions of anti-COVID plasma at intervals of 12-24 hours in a volume of 200-325 ml, prepared from different donors.

Anti-COVID plasma can also be used for plasma substitution when performing plasmapheresis.

Contraindications to the clinical use of anti-COVID plasma:

- History of allergic reactions to plasma proteins or sodium citrate;
- Patients with a history of autoimmune diseases or selective IgA deficiency require careful evaluation for possible side effects;
- General contraindications for fresh frozen plasma transfusion.

Use of human immunoglobulin against COVID-19

The use of a highly purified immunoglobulin preparation is based on the concept of passive immunization. The active ingredient of the preparation is immunoglobulins of class G (at least 95%), which have the activity of antibodies to SARS-CoV-2, obtained from a pool of donor plasma. The preparation is manufactured using a technology that includes procedures for inactivation and/or removal of viruses. IgG molecules are not subject to change during the manufacture of the preparation due to chemical or enzymatic action. The preparation does not contain preservatives and antibiotics.

Following intravenous infusion, the duration of IgG to SARS-CoV-2 at maximum concentration varies from 25 minutes to 6 hours. The half-life of IgG to SARS-CoV-2 is on average 11 days.

Indications for the use of human immunoglobulin against COVID-19:

Treatment of infection caused by the SARS-CoV-2 virus as part of complex therapy. Recommended for patients with a high risk of severe disease (age over 65, presence of concomitant diseases (diabetes mellitus, obesity, chronic cardiovascular diseases).

Contraindications for the use of human immunoglobulin against COVID-19:

- hypersensitivity to human immunoglobulin, especially in rare cases of deficiency of immunoglobulin class A (IgA) in the blood and the presence of antibodies against IgA;
- hypersensitivity to the components of the drug;
- history of allergic reactions to human blood products;
- pregnancy and breastfeeding period.

Method of use of human immunoglobulin against COVID-19:

The drug is administered once without dilution intravenously by drip at a dose of 4 ml/kg of body weight. The initial rate of administration is from 0.01 to 0.02 ml/kg of body weight per minute for 30 minutes. If the drug is well tolerated, the rate of administration can be gradually increased to a maximum of 0.12 ml/kg of body weight per minute.

The drug should be administered only in a hospital setting, observing all aseptic rules. Before administration, the solution temperature should be brought to room temperature or the patient's body temperature. The solution should be transparent or slightly opalescent. The drug is not suitable for use in vials with damaged integrity and/or markings, in case of turbidity of the solution, color change, presence of sediment in the solution, if the expiration date has expired, if storage conditions are not observed. Any amount of the drug remaining after infusion should be destroyed.

The drug can be used in combination with other drugs, in particular with antibiotics. However, mixing the drug with other drugs in one bottle is not allowed.

Human immunoglobulin may be used as a component of any of the inpatient regimens (Appendix 8-2) in patients with a confirmed diagnosis of COVID-19.

Etiotropic treatment of pregnant women, women in labor and women in childbirth

Detailed information on diagnostics, prevention and treatment of pregnant women, women in labor and women in childbirth and newborns is presented in the methodological recommendations of the Ministry of Health of Russia "Organization of medical care pregnant women, women in labor, women in labor and newborns with the new coronavirus COVID-19 infections " .

Etiotropic treatment of COVID-19 in women during pregnancy and breastfeeding has not yet been developed, however, antiviral drugs may be prescribed as etiotropic therapy, taking into account their effectiveness against COVID-19 for vital indications. In other cases, their safety during pregnancy and breastfeeding should be taken into account.

Treatment should be started as early as possible, which ensures recovery to a greater extent. Antiviral drugs for pregnant women with severe or progressive disease should be prescribed at a later stage from the onset of the disease.

When prescribing antiviral drugs to nursing women, the decision on whether to continue breastfeeding depends on the severity of the mother's condition.

5.2. PATHOGENETIC TREATMENT

Clinical trials of the efficacy and safety of targeted drugs in patients with severe/critical coronavirus infection are currently ongoing.

Considering that secondary macrophage activation syndrome (SAM)/hemophagocytic lymphohistocytosis (HLH) in COVID-19 is a consequence of massive uncontrolled activation of the immune system (hyperimmune reaction) provoked by an acute viral infection, patients, along with symptomatic and etiotropic treatment, in most cases should undergo immunosuppressive therapy.

Taking into account the peculiarities of the course of the disease in case of infection with the Delta strain of SARS-CoV-2 (early development of a hyperimmune reaction and massive lung damage), pathogenetic therapy, including therapeutic regimens,

used in outpatient settings, it is advisable to initiate at the early stages of the disease.

Algorithms for pathogenetic treatment of COVID-19 are presented in the table (Appendix 8-2) in the form of alternative therapeutic regimens that include genetically engineered biological drugs and/or GCS.

For outpatient treatment of patients with mild COVID-19 , inhalation budesonide (dosed inhalation powder) may be prescribed as an additional therapy at a dose of 800 mcg 2 times a day

until recovery, but not more than 14 days. This therapy can reduce the frequency of seeking emergency medical care, the risk of hospitalization, and reduce the time to recovery.

In case of hospitalization of a patient with a mild/moderate course of COVID-19 and risk factors for a severe course in the presence of pathological changes in the lungs corresponding to CT1-2, or moderate pneumonia according to X-ray examination data (heterogeneous darkening of a round shape and varying extent, involvement of the lung parenchyma ≤ 50%) in combination with two or more signs:

- SpO2 - 97 and above, without signs of shortness of breath
- 3N ≤ CRP level ≤ 6N
- Body temperature 37.5-37.9 °C for 3-5 days
- Leukocyte count - 3.5-4.0×10 ^{9} /l
- Absolute lymphocyte count - 1.5-2.0×10 ^{9} /l

It is recommended to prescribe a Janus kinase 1,2 inhibitor (tofacitinib or baricitinib) or an interleukin 17A inhibitor (netakimab) or the IL-6 receptor antagonist levilimab (subcutaneous/intravenous), or the IL-6 blocker olokizumab (subcutaneous/intravenous) (see Appendix 8-2).

In the presence of pathological changes in the lungs corresponding to CT1-2, or moderate pneumonia according to X-ray examination data (heterogeneous darkening of a round shape and varying length, involvement of the lung parenchyma ≤ 50%) in combination with two or more of the following signs: • SpO_2 94-97%, shortness of breath during physical exertion

- 6N ≤ CRP level < 9N
- Body temperature ≥ 38°C for 3-5 days
- Leukocyte count - 3.0-3.5×10 ^{9} /l
- Absolute lymphocyte count - 1.0-1.5×10 ^{9} /l

Intravenous administration of IL-6 receptor antagonists – tocilizumab or sarilumab, or levilimab, or the IL-6 blocker olokizumab is recommended (see Appendix 8-2) .

During the first 7 days of illness and/or in case of positive SARS-CoV-2 RNA , therapy with IL-6 receptor antagonists or IL-6 blockers is combined with etiotropic drugs (favipiravir, or remdesivir, or human immunoglobulin against COVID-19) as well as with anticoagulants (see Appendix 8-2) .

Table 2. "Empirical" visual scale for assessing the severity of changes in lung tissue based on chest X-ray data

Degree of change	Main manifestations of viral pneumonia
Medium-heavy	Heterogeneous darkening of round shape and varying length<br>Lung parenchyma involvement ≤ 50%

Heavy	Confluent opacities of the infiltration type (symptom of "white lungs")<br>Lung parenchyma involvement ≥ 50%

Glucocorticosteroids (GCS) are the first-line drugs for the treatment of patients with primary HLH and secondary SAM/HLH; they suppress all phases of inflammation, the synthesis of a wide range of proinflammatory mediators, an increase in the concentration of which within the cytokine storm is associated with an unfavorable prognosis for COVID-19 and the risk of developing ARDS and sepsis.

It takes several hours for the full effect of GCS to manifest. The maximum pharmacological activity of GCS occurs during the period of time when their peak concentrations in the blood are already behind them.

It is not recommended to use GCS for oral and intravenous administration for the treatment of mild forms of COVID-19, including in outpatient and inpatient settings.

For the treatment of severe COVID-19 (pneumonia with respiratory failure/ARDS, signs of "cytokine storm"), various regimens for the administration of GCS can be used: dexamethasone at a dose of 16-24 mg/day intravenously, depending on the severity of the patient's condition, in 1-2 administrations; methylprednisolone at a dose of 125 mg/administration/intravenously every 6-12 hours or 250 mg/administration/intravenously at one time.

With progression of macrophage activation syndrome (increase in ferritin level, serum CRP, development of two- or three-line cytopenia), methylprednisolone is used according to the regimen 125 mg/administration/intravenously every 6-12 hours or dexamethasone 20 mg/intravenously.

The maximum dose of GCS is used for 3-4 days and then reduced when the condition stabilizes (relief of fever, stable decrease in the level of CRP, ferritin (by at least 15%), activity of ALT, AST, LDH in the blood serum).

The dose of GCS is reduced by 20-25% every 1-2 days, then by 50% every 1-2 days until complete discontinuation.

The use of GCS should be in combination with anticoagulant therapy.

If there are signs of adrenal insufficiency, hydrocortisone is administered intravenously at a dose of 50-100 mg, followed by slow intravenous administration of the drug over 1 hour at a dose of 200 mg per day.

Patients receiving GCS should be monitored for adverse effects. In severely ill patients, these include hyperglycemia, hypertension, erosive and ulcerative lesions of the upper gastrointestinal tract, and an increased risk of infections (including bacterial, fungal); the frequency of these infections in patients with COVID-19 is unknown.

GCS should be used with caution in:

- Diabetes mellitus
- Hypertension
- Gastric ulcer and duodenal ulcer
- Obesity
- Signs of an active bacterial infection
- Thrombotic disorders

In the absence of contraindications for the treatment of severe forms of COVID-19 and cytokine storm, GCS are used in combination with IL-6 receptor antagonists, an IL-6 blocker, or an IL-1 blocker (Appendix 8-2).

In the presence of pathological changes in the lungs corresponding to CT1-4, or pneumonia according to X-ray examination data of moderate severity (heterogeneous opacities of round shape and varying extent; involvement of the lung parenchyma ≤ 50%) or severe degree (confluent opacities of the infiltration type (symptom of "white lungs") involvement of the lung parenchyma ≥ 50%)) in combination with two or more of the following signs:

- SpO2 < 93%, dyspnea at rest/progressive dyspnea during the first 2-3 days
- Body temperature > 38 °C for 2-3 days
- CRP level > 9N or a 3-fold increase in CRP level on days 3-5 of illness
- Leukocyte count < 3.0×10^9/l
- Absolute blood lymphocyte count < 1.0×10^9/l
- Serum ferritin* level ≥ 2 normal values on days 3-5 of illness
- Serum LDH level ≥ 1.5 normal values on days 3-5 of illness

- IL-6 level* > 40 pc/ml.

It is recommended to prescribe IL-6 receptor antagonists (tocilizumab, sarilumab, levilimab), IL-1 blockers (canakinumab, anakinra), or an IL-6 blocker (olokizumab) intravenously (Appendix 8-2) .

During the first 7 days of illness and/or in case of positive SARS-CoV-2 RNA , therapy with IL-6 receptor antagonists or IL-1β blocker is combined with etiotropic treatment drugs (remdesivir or human immunoglobulin against COVID-19) and with anticoagulants (Appendix 8-2) .

*** Serum ferritin and IL-6 levels are determined when studies are available.**

If monotherapy with IL-6 receptor antagonists (tocilizumab, sarilumab, levilimab) or an IL-6 blocker (olokizumab) is ineffective, combination therapy may be used by prescribing an IL-6 receptor antagonist and, if therapy is ineffective after 12-24 hours, additional use of an IL-6 blocker (olokizumab) in the recommended doses (see Appendix 6).

IL-6 receptor antagonists (tocilizumab, sarilumab, levilimab), IL-6 (olokizumab) and IL-1 (canakinumab, anakinra) blockers are prescribed in combination with GCS (see above).

Tocilizumab is administered at a dose of 4-8 mg/kg intravenously. (the dose of the drug is calculated based on body weight) in combination with GCS.

Re-administration of tocilizumab is possible if febrile fever persists for more than 12 hours after the first infusion.

Sarilumab is administered at a dose of 200 mg or 400 mg subcutaneously or intravenously.

Canakinumab is administered at a dose of 4 mg/kg (no more than 300 mg) intravenously when tocilizumab or sarilumab cannot be used or is ineffective.

Anakinra is prescribed at a dose of 200–400 mg/day, intravenously by drip for 10 days.

It is important to remember that when prescribing the above-mentioned genetically engineered biological drugs, the risk of developing a secondary bacterial infection increases.

Contraindications for the administration of genetically engineered biological drugs:

- sepsis confirmed by pathogens other than COVID-19;
- hypersensitivity to any component of the drug;
- viral hepatitis B;
- concomitant diseases associated, according to clinical judgment, with an unfavorable prognosis;
- immunosuppressive therapy in organ transplantation;
- neutropenia < 0.5×10^{9}/l;
- increase in AST or ALT activity by more than 5 times the norm;
- thrombocytopenia <50×10^{9}/l;
- The use of GIBP during pregnancy is possible if the potential benefit outweighs the risk.

When using IL-6 blockers, one should be aware of the occurrence of adverse events:

- Infectious diseases:
  bacterial pneumonia, phlegmon, infections caused by Herpes zoster, etc.;
- rash, hives;
- increase in lipid metabolism indicators
  (total cholesterol, triglycerides, HDL, LDL).

Elderly and senile age are not contraindications for the use of monoclonal antibodies to cytokines and their receptors. For patients receiving immunosuppressant treatment during organ transplantation, the prescription of these drugs is possible by decision of the medical commission with the correction of the initial basic immunosuppressive therapy - a decrease in the dose of calcineurin inhibitors, the concentration of cyclosporine 40-50 ng / ml and tacrolimus to 1.5-3 ng / ml, cancellation of the cytostatic, a 2-fold increase in the dose of GCS. Control of opportunistic infections is necessary.

With increasing signs of respiratory failure, the appearance of subfebrile/febrile fever with normal/moderate

If there are elevated/significantly elevated inflammation markers (ESR, CRP, procalcitonin and blood leukocyte levels), it is necessary to suspect the development of a fungal and/or opportunistic infection and carry out:

- microbiological (cultural) examination of bronchoalveolar lavage fluid, sputum or endotracheal aspirate for bacteria, yeast fungi and DNA *of Mycobacterium tuberculosis* , *Pneumocystis jirovecii* by PCR method;
- blood test for antibodies of class A and M (IgA, IgM) to fungi of the genus Aspergillus (*Aspergillus spp* .), *Pneumocystis jirovecii.*
- control CT scan of the chest organs to confirm/exclude the development of interstitial pneumonia.

Drugs for preemptive anti-inflammatory therapy are presented in Appendix 6 .

Characteristics of coagulopathy in COVID-19

Severe life-threatening cytokine release syndrome may result in the development of blood coagulation disorders. In the initial stages of the disease, hypercoagulation without signs of consumption and DIC syndrome is typical.

Coagulopathy in COVID-19 is characterized by activation of the blood coagulation system in the form of a significant increase in the concentration of D-dimer in the blood. The platelet count is moderately reduced, prothrombin time is slightly prolonged, and fibrinogen is significantly increased. Isolated studies indicate that the concentration of antithrombin in the blood rarely

decreases below 80%. The concentration of protein C also does not change significantly. Thus, coagulopathy in COVID-19, along with the signs characteristic of the advanced phase of DIC syndrome in the form of a high level of D-dimer, does not have typical signs of fibrinogen and platelet consumption. Also, there is no consumption of components of the anticoagulant system antithrombin and protein C, characteristic of DIC syndrome observed in sepsis. Interest in coagulopathy in COVID-19 is due to the fact that its presence is associated with the risk of death. In addition, arterial and venous thrombosis is often found in patients with COVID-19 .

Analysis of autopsy data of patients who died from COVID-19 indicates the presence of, in addition to diffuse damage to the alveoli, multiple thromboses of small vessels of the lungs and associated multiple hemorrhages in the alveoli. Megakaryocytes and platelets are involved in the thrombotic process in the lungs; the resulting thrombi are rich not only in fibrin, but also in platelets. Signs of thrombotic microangiopathy in the lungs are noted.

SARS-CoV-2 infiltration , widespread small vessel thrombosis, microangiopathy, alveolar capillary occlusion, and signs of neoangiogenesis.

DIC syndrome usually develops in the late stages of the disease. It occurs in only 0.6% of surviving patients and in 71.4% of deceased patients. The development of hypercoagulation is associated with the risk of thrombotic complications. To verify the diagnosis of PE, it is necessary to perform CT with intravenous contrast, and to diagnose deep vein thrombosis (DVT) of the lower extremities, an ultrasound of the vessels of the lower extremities is required.

Antithrombotic therapy

Antithrombotic therapy in patients managed at home Prevention of lower extremity DVT/PE using prophylactic doses of low molecular weight heparin (LMWH)/fondaparinux sodium should be considered for patients with moderate COVID-19 who are managed at home under quarantine and have a high risk of venous thromboembolic complications, a low risk of bleeding, and are not receiving anticoagulant treatment for other indications. This applies primarily to patients with severely limited mobility, a history of DVT/PE, active malignancy, major surgery or trauma in the previous month, carriers of a number of thrombophilias (antithrombin deficiency, protein C or S, antithrombin lipid syndrome, factor V Leiden, prothrombin gene mutation G-20210A), as well as in the presence of a combination of additional risk factors for DVT/PE (age over 70 years, cardiac/respiratory failure, obesity, systemic connective tissue disease, hormone replacement therapy/oral contraceptive use).

data on the use of direct oral anticoagulants in COVID-19 , however, in the absence of LMWH/UFH/fondaparinux sodium, the use of direct oral anticoagulants (rivaroxaban at a dose of 10 mg once a day or apixaban at a dose of 2.5 mg twice a day) can be considered. Dabigatran etexilate can be considered as a second-line drug. However, it should be taken into account that it

has not been studied as a means of preventing DVT/PE in non-surgical patients and its dose for this indication is unknown. In addition, the elimination of dabigatran etexilate is most dependent on renal function. Given the experience of using dabigatran etexilate for the prevention of DVT/PE in orthopedic surgery, as well as the results of its study for the prevention of vascular complications after non-cardiac surgery in patients with sinus rhythm and elevated cardiac troponin levels, it is possible that its dose may be 110 mg 2 times a day (for patients with creatinine clearance of 30-49 ml/min - 75 mg 2 times a day).

When prescribing anticoagulants on an outpatient basis, special attention should be paid to the presence of contraindications (primarily severe renal failure); during their use, it is necessary to re-assess the risk and actively look for signs of bleeding. The duration of anticoagulant use in outpatient treatment of COVID-19 is unclear, and, apparently, can last up to 30 days, depending on the dynamics of the patient's clinical condition and the timing of restoration of motor activity.

Antithrombotic therapy in patients admitted to hospital . LMWH/ fondaparinux sodium, at least in prophylactic doses, is indicated for ALL hospitalized patients and should be continued at least until discharge. There is no proven advantage of any one LMWH over others. If LMWH/fondaparinux sodium is unavailable or contraindicated, UFH may be used.

Increasing the heparin dose to intermediate or therapeutic levels may be considered in patients with high and very high D-dimer levels, in the presence of additional risk factors for venous thromboembolic complications, as well as in severe manifestations of COVID-19. According to published results of randomized controlled trials in patients in the ICU, routine escalation of the LMWH/UFH dose to intermediate and therapeutic levels does not improve clinical outcomes. Data on the appropriateness of routine use of high-dose anticoagulants in hospitalized patients outside the intensive care unit are conflicting. In obese patients (body mass index >30 kg/m ^{2}), an increase in the prophylactic dose by 50% should be considered.

Extended DVT/PE prophylaxis . *Extended prophylaxis in* COVID-19 patients after discharge may be considered if there is a persistent elevated risk of venous thromboembolic complications (see p. 58) and a low risk of bleeding [VIII]when therapeutic doses of anticoagulant are not required for other indications. As an anticoagulant for prolonged prophylaxis of DVT/PE in medical patients, there is an evidence base for prophylactic doses of enoxaparin (40 mg once daily) and rivaroxaban at a dose of 10 mg once daily. A trend towards a benefit in prolonged prophylaxis of DVT/PE was also noted with the use

[VIII]assessment of the risk of bleeding in patients with COVID-19 . According to the IMPROVE scale , the most significant risk factors for major and clinically significant minor bleeding in the first 2 weeks of hospitalization in medical patients are: active gastric or duodenal ulcer, bleeding in the previous 3 months, age over 85 years, blood platelet count below 50x10 ^{9} /l, severe liver failure, glomerular filtration rate below 30 ml/min/1.73 m ^{2} . Contraindications to anticoagulants, as set out in the instructions for the drugs, should also be taken into account.

of apixaban at a dose of 2.5 mg twice daily.

Extended DVT prophylaxis (up to 30-45 days after discharge) may be prescribed to patients with one of the following: age over 60 years, hospitalization in the intensive care unit, active malignancy, history of DVT/PE, persistent severe limitation of mobility, D-dimer blood concentration 2 or more times the upper limit of normal.

Treatment of DVT/PE.

In case of DVT/PE development in a patient with COVID-19, therapeutic doses of LMWH/UFH/fondaparinux sodium should be used. The use of therapeutic doses of LMWH/UFH/fondaparinux sodium may also be considered in patients with clinical suspicion of thrombotic complications when it is not possible to verify the diagnosis. Treatment of DVT/PE associated with COVID-19 with anticoagulants should be continued for at least 3 months; after discharge, it is preferable to use direct oral anticoagulants in generally accepted doses, unless there are contraindications to them.

Features of the use of anticoagulants .

The list of possible anticoagulants and their doses is presented in Appendix 7-1 . Algorithms for the use of anticoagulants for the treatment of COVID-19 in adult patients in the inpatient and outpatient settings are presented in Appendices 7-2 and 7-3, respectively. In patients with heparin-induced immune thrombocytopenia and with a low baseline platelet count, it is recommended to use anticoagulants for the prevention and treatment of venous thromboembolic complications.

use fondaparinux sodium. Fondaparinux sodium, unlike LMWH/UFH preparations, is devoid of potentially favorable pleiotropic effects, but, on the other hand, it does not cause heparin-induced thrombocytopenia.

LMWH/fondaparinux sodium is not recommended for use in patients with severe renal impairment or rapidly changing renal function.

Contraindications for the use of anticoagulants are ongoing bleeding, blood platelet levels below $25*10^{9}$/l, history of heparin-induced thrombocytopenia, severe renal failure (for LMWH and sodium fondaparinux). Increased prothrombin time and APTT are not contraindications for the use of anticoagulants.

If anticoagulants are contraindicated, mechanical methods of lower extremity DVT prophylaxis (preferably intermittent pneumatic compression) should be used. The use of mechanical methods of lower extremity DVT prophylaxis in addition to anticoagulants may also be considered in patients in intensive care units.

Prevention of DVT/PE in patients taking oral anticoagulants for other indications.

Patients receiving oral anticoagulants for other indications may continue taking them if

COVID-19 manifestations are not severe. In case of unacceptable drug interactions with drugs used to treat COVID-19 (Appendix 4), as well as in case of severe COVID-19, it is recommended to switch to therapeutic doses of heparin drugs (preferably LMWH/fondaparinux sodium).

Evaluation of hemostasis parameters during hospital stay.

Multiplicity of D-dimer determination (see also Appendix 2-2) , prothrombin time, fibrinogen and platelet count depend on the severity of COVID-19 infection, the dynamics of both an increase and a decrease in indicators are important. In hospitalized patients with moderate severity - once every two days, with severe - daily. An extraordinary analysis of the above indicators is taken when the severity of COVID - 19 worsens.

To diagnose DIC syndrome, it is recommended to use the criteria of the International Society of Thrombosis and Hemostasis (Table 3). The diagnosis of DIC syndrome is established if there are 5 points according to these criteria.

Table 3. Criteria for overt DIC of the International Society on Thrombosis and Haemostasis.

Indicator	Score	Values of the indicator
Platelet count (per 10^9/l)	2	< 50
	1	≥ 50, < 100
Fibrin degradation products/D-dimer	3	Greatly increased
	2	Moderately elevated
Prothrombin time	2	≥ 6 sec
	1	≥ 3 sec, < 6 sec
Fibrinogen (mg/ml)	1	< 100

In patients with consumption coagulopathy in the absence of bleeding, the platelet count should be maintained above $20*10^9$/l, plasma fibrinogen concentration above 2.0 g/l. In patients with bleeding, the platelet count should be maintained above $50*10^9$/l, plasma fibrinogen concentration above 2.0 g/l, and prothrombin ratio < 1.5.

In patients with very low or high body weight, severe renal impairment, high risk of bleeding, and during pregnancy, it is advisable to select the heparin dose under the control of anti-Xa activity in the blood (target ranges are presented in Appendix 7-1) . In patients in critical condition, as well as when using methods of extracorporeal circulatory support, in the absence of the possibility of monitoring the state of the hemostasis system by traditional methods, it is permissible to use global tests (thromboelastography/thromboelastometry) if there is experience in their use and interpretation.

Evaluation of the effectiveness of anti-inflammatory therapy

The main criteria for the effectiveness of preemptive anti-inflammatory therapy are the dynamics of the clinical response: a decrease in fever, improved well-being, appetite, decreased dyspnea, and an increase in SpO_2 . The effect of IL-6 blockers occurs within 12 hours (1-2 injections are most often used), GC treatment - from 12 to 72 hours (a course of 2-3 days), anticoagulant therapy - from 120 hours (a course of 5 days). If the effect of preemptive anti-

inflammatory therapy is not achieved, then it is necessary to assume other causes of deterioration of the condition (progression of pneumonia, sepsis, etc.).

Infusion therapy

When treating COVID-19, it is necessary to ensure adequate fluid intake. Daily fluid requirements should be replenished primarily through oral rehydration. Daily fluid requirements should be calculated taking into account fever, shortness of breath, and fluid loss.

fluids for diarrhea, vomiting (if the patient has such symptoms). In order to prevent pulmonary edema, patients should limit the volume of fluid administered, maintain zero or negative hydrobalance. In case of severe intoxication, as well as abdominal discomfort, nausea and/or vomiting, enterosorbents are indicated (colloidal silicon dioxide, polymethylsiloxane polyhydrate, etc.).

Patients in severe condition (in the intensive care unit) are given infusion therapy if indicated. Infusion therapy should be used with caution, since excessive fluid transfusions can worsen blood oxygen saturation, especially in conditions of limited mechanical ventilation, and can also provoke or worsen ARDS manifestations. The volume of infusion therapy should be 10-15 ml/kg/day.

When carrying out infusion therapy, the rate of fluid administration is of great importance: the lower the rate of fluid administration, the safer it is for the patient.

In the conditions of infusion therapy, the doctor evaluates daily diuresis, blood pressure dynamics, changes in the auscultatory picture in the lungs, hematocrit (not lower than 0.35/l). If the volume of diuresis decreases, blood pressure increases, the number of wheezing in the lungs increases, and hematocrit decreases, the volume of parenterally administered fluid should be reduced.

To reduce the volume of infusion therapy, the patient's nutritional status should be maintained, if necessary, by tube feeding using standard and semi-elementary mixtures for enteral nutrition. Feeding should be frequent and fractional to avoid overfilling the stomach and reduce lung excursion.

In order to improve expectoration in productive cough, mucoactive drugs (acetylcysteine, ambroxol, carbocysteine) are prescribed. Bronchodilator inhalation therapy using salbutamol, fenoterol, with the use of combined agents (ipratropium bromide + fenoterol) is advisable in the presence of broncho-obstructive syndrome.

One of the promising treatment methods for ARDS associated with COVID-19 is the use of exogenous surfactant preparations. The most important property of pulmonary surfactant is its ability to reduce surface tension in the alveoli; in addition, surfactant is involved in the barrier and protective functions of the lungs, affecting innate and adaptive local immunity. In ARDS, type II alveolocytes are damaged, which leads to a disruption in the synthesis of pulmonary surfactant de novo, and as a result of the release of leukocytes and blood plasma proteins into the alveolar space, surfactant is inhibited.

In Russia, the use of the natural drug Surfactant-BL is approved for the treatment of ARDS

in adult patients. According to the results of pilot studies, the use of Surfactant-BL inhalations for ARDS associated with COVID-19 was accompanied by an improvement in the oxygenation level and a decrease in the risk of using invasive respiratory support. The drug is recommended for use with SpO2 ≤ 92% in non-intubated patients using a nebulizer (mesh nebulizers are preferable) at a dose of 75-150 mg 2 times a day for 3-5 days.

5.3. SYMPTOMATIC TREATMENT

Symptomatic treatment includes:

- Relief of fever
  (antipyretic drugs, such as paracetamol or ibuprofen);
- Complex therapy of rhinitis and/or nasopharyngitis
  (moisturizers/eliminators, nasal decongestants);
- Complex therapy of bronchitis
  (mucoactive, bronchodilator and other agents).

Antipyretics are prescribed when the body temperature is > 38.0-38.5 °C. In case of poor

In cases of intolerance to fever syndrome, headaches, increased blood pressure and severe tachycardia (especially in the presence of ischemic changes or rhythm disturbances), antipyretic drugs are used even at lower numbers. The safest drug is paracetamol.

For local treatment of rhinitis, pharyngitis, nasal congestion and/or discharge, start with topical saline solutions based on sea water (isotonic, and hypertonic for congestion). If they are ineffective, nasal decongestants are indicated. If ineffective or symptoms are severe, various solutions with antiseptic action can be used. To stop coughing in COVID-19, antitussive drugs are used: butamirate, levodroprisin, rengalin.

Symptomatic treatment in pregnant women, women in labor and women in childbirth

The antipyretic drug of first choice is paracetamol, which is prescribed at 500-1000 mg up to 4 times a day (no more than 4 g per day).

During pregnancy (II and III trimesters), in the postpartum and post-abortion period, it is possible to use mucolytic agents using a mesh nebulizer (ambroxol 2-3 ml with isotonic solution 2 ml 3 times a day) and bronchodilators (ipratropium bromide + fenoterol 20 drops in 2-4 ml of isotonic solution 2 times a day). During pregnancy (I, II and III trimesters), in the postpartum and post-abortion period, salbutamol can also be used as a bronchodilator using a mesh nebulizer (2.5-5 mg 2 times a day).

Adequate respiratory support is a necessary component of complex therapy. Oxygen saturation indicators should be determined in all pregnant women with clinical features of acute respiratory disease and/or pneumonia.

unit for coronavirus infection are rapidly progressing acute respiratory failure (RR> 25 in 1 min, SpO2 < 92% when breathing atmospheric air, as well as other organ failure (2 or more points on the SOFA scale).

Peculiarities of care for elderly and senile patients

In elderly and senile patients, delirium may be the first manifestation of the disease or develop as it progresses. Hospitalization of the patient, placement in the intensive care unit and on artificial ventilation increase the risk of developing delirium. The development of delirium, especially the hyperactive form, may present additional difficulties in treating patients.

Recommendations:

1. Routine screening for delirium using recommended tools in all elderly and geriatric patients hospitalized with COVID-19.

2. It is recommended to prevent delirium in all elderly and senile patients hospitalized due to COVID-19 by correcting its triggers. Preventive measures include: regular patient orientation in their personality, time and space, prevention of constipation, pain treatment, treatment of bacterial pneumonia (there is a high probability of its occurrence in patients with COVID-19), adequate oxygen therapy, prevention of urinary retention, as well as review of concomitant therapy to minimize the use of drugs that increase the risk of delirium.

3. When behavioral disturbances occur, it is necessary first of all to assess and correct the triggers for the development of delirium (see above). If these measures are ineffective and/or there is a need for more rapid control of the situation to reduce the risk to the patient or others, an earlier than usual transition to pharmacological treatment may be required.

4. When prescribing fast-acting tranquilizers, it is necessary to monitor the development of side effects, the state of vital signs (respiratory rate, SpO2 , blood pressure, heart rate), hydration level and consciousness, at least every hour until there is confidence in their stability. It should be remembered that benzodiazepines depress respiration, and also not to exceed the maximum doses of haloperidol (2 mg / day regardless of the route of administration), risperidone (1 mg / day in several doses), lorazepam (2 mg / day regardless of the route of administration).

In elderly and senile patients, COVID-19 increases the risk of malnutrition. The increased risk is associated with both clinical symptoms of the disease (decreased sense of smell and taste, decreased appetite, diarrhea, fever, weakness) and social factors (self-isolation), which in some cases potentiate pre-existing risks. Elderly patients with polymorbidity are more susceptible to the risk of malnutrition and associated sarcopenia; it is this group that has the most unfavorable prognosis for COVID-19 . Mechanical ventilation, especially prolonged, is a well-documented cause of malnutrition with loss of muscle mass and function, which, in turn, is associated with a further decrease in quality of life and loss of autonomy after discharge from the hospital. Adequate assessment of nutritional status, prevention and correction of malnutrition effectively reduce the risk of complications and improve clinical outcomes.

Recommended:

1. All elderly and geriatric patients hospitalized with COVID-19 should be screened for risk of malnutrition using validated tools.

2. For patients who, based on screening results, fall into the categories of “risk of malnutrition” or “malnutrition”, correction should be carried out using oral enteral nutrition (OEN) products.

3. It is recommended to calculate the energy value of the diet of an elderly and senile patient, focusing on the level of 30 kcal per kg of body weight per day, the daily level of protein consumption should be at least 1.0-1.2 g per kg of body weight per day (in severe cases of the disease and diagnosed malnutrition - up to 2.0 g / kg of body weight per day).

4. It is recommended to prescribe PEP preparations that provide the body with at least 400 kcal/day of energy and at least 30 g/day of protein. In the absence of contraindications, PEP products containing dietary fiber are preferable for ensuring optimal functioning of the gastrointestinal tract.

5. In cases where oral feeding is not possible, it is recommended to start enteral tube feeding (ETT) without delay.

6. At discharge from hospital, patients with or at risk of malnutrition are recommended to use AEDs to optimize food intake and body weight and to reduce the risk of functional decline. It is recommended to prescribe AEDs for at least one month and to evaluate the effectiveness and expected benefit of AEDs once a month.

5.4. ANTIBACTERIAL AND ANTIMYCOTIC THERAPY FOR COMPLICATED FORMS OF INFECTION

5.4.1. Antibacterial therapy for complicated forms of infection

COVID-19, like any other viral infection, is not an indication for the use of antibiotics. The pathogenesis of lung damage in COVID-19 is based on immune mechanisms - macrophage activation syndrome with the development of a "cytokine storm", which is not affected by antibacterial drugs.

Antibacterial therapy is prescribed only in the presence of convincing signs of a bacterial infection (an increase in PCT of more than 0.5 ng/ml, the appearance of purulent sputum, leukocytosis > 12×10^{9}/l (in the absence of previous use of glucocorticoids), an increase in the number of band neutrophils of more than 10%).

Bacterial infections rarely complicate the course of COVID-19. Thus, in a meta-analysis of 24 studies including 3,338 patients, the frequency of bacterial coinfection at the time of seeking medical care was 3.5% (95% CI 0.4-6.7%); secondary bacterial infections complicated the course of COVID-19 in 14.3% of patients (95% CI 9.6-18.9%); in general, the proportion of patients with bacterial infections was 6.9% (95% CI 4.3-9.5%); bacterial infections were more often recorded in patients with severe COVID-19 (8.1%, 95% CI 2.3-13.8%). Therefore, the vast majority of patients with COVID-19, especially with mild and moderate course, **DO NOT NEED** antibacterial therapy.

If a bacterial infection develops outside a hospital or in the first 48 hours of hospital stay, antibacterial therapy is the same as for community-acquired pneumonia (Tables 4-6).

In patients who do not require hospitalization, it is advisable to prescribe oral dosage forms.

Table 4. Antibacterial therapy for community-acquired pneumonia (CAP)

Group	Drugs of choice	Alternative

Non-severe CAP in patients without comorbidities [1] , who have not taken antibiotics for ≥ 2 days in the last 3 months and have no other risk factors[IX]	Amoxicillin orally	Macrolide orally[X]
Non-severe CAP in patients with comorbidities [1] and/or who have taken antibiotics for ≥2 days in the last 3 months and/or have other risk factors [2]	Amoxicillin/clavulanic acid and other oral IDUs	RX (levofloxacin, moxifloxacin) orally OR CS III[XI] inside

Note : IPA – inhibitor-protected aminopenicillin (amoxicillin/clavulanic acid, amoxicillin/sulbactam, ampicillin/sulbactam), RQ – respiratory quinolone, CS – cephalosporin [1] COPD, diabetes, CHF, CKD, liver cirrhosis, alcoholism, drug addiction, exhaustion

[3] In areas with high (>25%) levels of *S. pneumoniae resistance* to macrolides (determined by erythromycin), the possibility of using RC should be considered. Preference should be given to the most studied macrolides in CAP with improved pharmacokinetic properties (azithromycin, clarithromycin); with known or suspected mycoplasma etiology in areas with a high (>25%) level of *M. pneumoniae resistance* In addition to macrolides, the use of RX or doxycycline should be considered.

[4] Cefditoren

Table 5. Antibacterial therapy for non-severe community-acquired pneumonia in hospital

Group	Drugs of choice	Alternative
Non-severe CAP in patients without concomitant diseases [1] , who have not taken AMP for ≥2 days in the last 3 months and do not have other risk factors [2]	Amoxicillin/clavulanic acid, etc. WPI* IV, IM OR Ampicillin IV, IM	RX (levofloxacin, moxifloxacin) i.v.
Mild CAP in patients with comorbidities [1] and/or recent history of 3 months AMP ≥2 days and/or having other risk factors [2]	Amoxicillin/clavulanic acid and other IDP* IV, IM<br>OR<br>III generation CS (cefotaxime, ceftriaxone, ceftriaxone/sulbactam) IV, IM<br>OR<br>RX (levofloxacin, moxifloxacin) i.v.<br>OR<br>Ceftaroline [3] IV<br>OR<br>Ertapenem [4] IV, IM	

Note : * IPA – inhibitor-protected aminopenicillin (amoxicillin/clavulanic acid, amoxicillin/sulbactam, ampicillin/sulbactam), CS – cephalosporin, RQ – respiratory quinolone

[1] COPD, diabetes, CHF, CKD, liver cirrhosis, alcoholism, drug addiction, exhaustion

[2] Risk factors for infection with rare and/or multidrug-resistant pathogens include residence in a nursing home or other long-term care facility, hospitalization for any reason for ≥ 2 days in the previous 90 days, IV therapy, dialysis, or home wound care in the previous 30 days

[3] Preferred in cases of high prevalence of penicillin-resistant S. pneumoniae (PRP) in the region or the presence of individual risk factors for PRP infection

[4] Use for limited indications - patients from long-term care facilities, presence of risk factors for aspiration, elderly and senile age with multiple comorbidities

[IX]Risk factors for infection with rare and/or multidrug-resistant pathogens include:
stay in a nursing home or other long-term care facility, hospitalizations
for any reason for ≥ 2 days in the previous 90 days, IV therapy, dialysis sessions or home wound treatment in the previous 30 days.

Table 6. Antibacterial therapy for severe (patient hospitalized in intensive care unit) community-acquired pneumonia

1. *Patients without additional risk factors*

Recommended mode:
Amoxicillin/clavulanic acid or ampicillin/sulbactam or cefotaxime or ceftriaxone or ceftaroline
+ azithromycin or clarithromycin
Alternative mode:
Amoxicillin/clavulanic acid or ampicillin/sulbactam or cefotaxime or ceftriaxone or ceftaroline or ceftriaxone/sulbactam + moxifloxacin or levofloxacin

2. *Patients with risk factors for PRP infection*

Recommended mode:
Ceftaroline or cefotaxime [2] or ceftriaxone [2]
+ azithromycin or clarithromycin
Alternative mode:
Ceftaroline or cefotaxime [2] or ceftriaxone [2] or ceftriaxone/sulbactam
+ moxifloxacin or levofloxacin

3. *Patients with risk factors for P. aeruginosa infection*

Recommended mode:
Piperacillin/tazobactam or cefepime or meropenem or imipenem
+ ciprofloxacin or levofloxacin
Alternative mode:
Piperacillin/tazobactam or cefepime or meropenem or imipenem
+ azithromycin or clarithromycin +/- amikacin

4. *Patients with risk factors for MRSA infection*

Recommended mode:
1. *Amoxicillin/clavulanic acid or ampicillin/sulbactam or cefotaxime or ceftriaxone*
+ azithromycin or clarithromycin
+ linezolid or vancomycin
2. Ceftaroline
+ azithromycin or clarithromycin
Alternative mode:
1. *Amoxicillin/clavulanic acid or ampicillin/sulbactam or cefotaxime or ceftriaxone or ceftriaxone/sulbactam*
+ moxifloxacin or levofloxacin
+ linezolid or vancomycin
2. Ceftaroline
+ moxifloxacin or levofloxacin

5. *Patients with risk factors for infection with enterobacteria, ESBL (+)*

Recommended mode:
Imipenem or meropenem or ertapenem
+ azithromycin or clarithromycin
Alternative mode:
Imipenem or meropenem or ertapenem
+ moxifloxacin or levofloxacin

6. *Patients with confirmed/suspected aspiration*

Recommended mode:
Ampicillin/sulbactam, amoxicillin/clavulanic acid, piperacillin/tazobactam, ertapenem
+ azithromycin or clarithromycin
Alternative mode:

Ampicillin/sulbactam, amoxicillin/clavulanic acid, piperacillin/tazobactam, ertapenem
+ moxifloxacin or levofloxacin

Note: PRP – penicillin-resistant *S. pneumoniae; MRSA* - methicillin-resistant *S.aureus;* ESBL - extended spectrum beta-lactamases

[1] In the presence of immediate-type allergic reactions to any beta-lactam, preference should be given to an ABP with a different chemical structure (for example, a respiratory quinolone can be prescribed in combination with linezolid or vancomycin).

[2] *cefotaxime should be prescribed in a dose of at least 6 g/day, ceftriaxone – 4 g/day*

In a hospital, in order to reduce the burden on medical personnel, it is advisable to use oral forms of antimicrobial drugs, step therapy (for example, amoxicillin/clavulanic acid intravenously - transition to amoxicillin/clavulanic acid orally, ceftriaxone, cefotaxime, ceftriaxone/sulbactam intramuscularly, intravenously with subsequent transition to cefditoren orally).

In case of clinical inefficiency or development of nosocomial (hospital-acquired) complications, the choice of antimicrobial therapy regimen should be based on the identification of risk factors for resistant pathogens, the results of monitoring antibiotic resistance in the hospital, analysis of previous therapy, and the results of microbiological diagnostics. For the treatment of nosocomial (hospital-acquired) bacterial infections in the hospital, depending on the results of monitoring the sensitivity of pathogens of nosocomial infections and the results of microbiological diagnostics in a particular patient, the following antibacterial drugs can be used: aztreonam (in combination with ceftazidime/avibactam), imipenem/cilastatin, linezolid, meropenem, piperacillin/tazobactam, polymyxin B (only in combination), telavancin, tigecycline, fosfomycin (only in combination), ceftazidime/avibactam, ceftolozane/tazobactam, cefepime/sulbactam, etc. The choice of antibacterial therapy for nosocomial infections is recommended based on consultation with a clinical pharmacologist.

5.4.2. Antifungal therapy for invasive aspergillosis and invasive candidiasis in patients with COVID-19

The main life-threatening mycoses in patients with COVID-19 are invasive aspergillosis (COVID-IA) and invasive candidiasis (COVID-IC). Other mycoses (mucormycosis, etc.) are much less common. COVID-IA and COVID-IC occur mainly in patients in the intensive care unit. Diagnosis of mycoses is presented in section 4.2.4.

The drugs of choice for the treatment of COVID-IA are voriconazole (IV 2x6 mg/kg on day 1, then 2x4 mg/kg/sec) and isavuconazole (IV 3x200 mg on days 1-2, then 200 mg/sec). After the patient's condition has stabilized, these drugs can be administered orally. Alternative drugs (liposomal AmB IV 3 mg/kg/sec, lipid complex AmB IV 5 mg/kg/sec, and caspofungin IV 70 mg on day 1, then 50 mg/sec) are prescribed when voriconazole or isavuconazole cannot be used. The duration of antifungal therapy is 4-6 weeks.

The risk of developing COVID-IC in ICU patients without specific risk factors (eg, severe neutropenia) is low, so routine primary antifungal prophylaxis is not recommended.

Early empirical administration of echinocandins improves survival in patients with septic shock. The indication for empirical therapy for COVID-IC in patients in the ICU is fever resistant

to adequate antibacterial therapy for more than 4 days, in combination with ≥2 risk factors (long-term use of a central venous line, total parenteral nutrition, use of corticosteroids or other immunosuppressants). In the presence of risk factors for COVID-IC and clinical signs of septic shock, empirical antifungal therapy should be initiated immediately. The drugs of choice for empirical therapy for COVID-IC are anidulafungin (IV 200 mg on day 1, then 100 mg/d), caspofungin (IV 70 mg on day 1, then 50 mg/d), and micafungin (IV 100 mg/d). When prescribing empirical therapy for COVID-19, the CVC should be replaced (not via a guidewire), and blood (≥40 ml per day for an adult patient), material from possible sites of dissemination, and the distal fragment of the CVC should be cultured.

If *Candida* spp. is isolated from normally sterile substrates (blood, CSF, etc.) within 24 hours, antifungal drugs should be prescribed and the CVC should be replaced (not via a guidewire). Anidulafungin, caspofungin, and micafungin are the drugs of choice for targeted therapy of all types of COVID-IC, except for meningitis and endophthalmitis. Triazole drugs (voriconazole IV or po 6 mg/kg 2 times on the first day, then 4 mg/kg 2 times a day, fluconazole IV or po 12 mg/kg on the first day, then 6 mg/kg/s) can be prescribed only if a drug-sensitive COVID-IC pathogen is isolated in a stable patient's condition, as well as for the treatment of candidal meningitis and endophthalmitis. In addition, voriconazole and fluconazole are used for de-escalation therapy after the patient has been stabilized on an echinocandin. Liposomal AmB (IV 3 mg/kg/sec) and lipid complex
AmB (IV 5 mg/kg/s) is used when echinocandins are ineffective, toxic or unavailable. Amphotericin B, posaconazole and itraconazole are not recommended for the treatment of IR. The duration of treatment is at least 14 days after the disappearance of clinical signs of COVID-IR and negative blood culture.

Features of antibacterial therapy in pregnant women, women in labor and women in childbirth

Initiate treatment with empirical antibiotics within 4 hours of diagnosis of pneumonia, or within 1 hour for severe pneumonia.

For patients with severe cases of the disease, antibacterial drugs are administered intravenously.

In secondary viral-bacterial pneumonia (the most likely pathogens are *Streptococcus pneumoniae* , *Staphylococcus aureus* and *Haemophilus influenzae*) it is preferable to use the following antibiotic therapy regimens:

- Third generation cephalosporin ± macrolide;
- Protected aminopenicillin ± macrolide;

In tertiary bacterial pneumonia (the most likely pathogens are methicillin-resistant strains

of *Staphylococcus aureus* , *Haemophilus influenza*), the following drugs (in various combinations) are prescribed:

- IV generation cephalosporin ± macrolide;
- Carbapenems;
- Vancomycin;
- Linezolid.

Antibacterial drugs that are contraindicated during pregnancy include tetracyclines, fluoroquinolones, and sulfonamides.

Features of the use of probiotics

The use of probiotic preparations as an additional therapy for various conditions is practiced quite widely, including their possible beneficial effect in the treatment of patients with COVID-19 is discussed. However, it should be noted that of the possible areas of use of probiotics for COVID-19, only their administration during and/or after antibacterial therapy for the prevention and treatment of various side effects has an evidence base. As a rule, preparations containing various types and strains of bifidobacteria and lactobacilli (including INN: Bifidobacterium bifidum and Bifidobacterium bifidum + Lactobacillus plantarum) are used as probiotics. It is important to emphasize that the use of probiotics for the prevention antibiotic-associated diarrhea are significantly more effective if they are prescribed as early as possible after the first dose of antibiotic.

5.5. OBSTETRIC TACTICS IN COVID-19

Obstetric tactics are determined by several aspects: the severity of the patient's condition, the condition of the fetus, and the gestational age. COVID-19, transferred before 12 weeks of pregnancy, due to the unproven negative effect on the fetus, is not a medical indication for termination of pregnancy. In severe and extremely severe cases of the disease, the issue of prolonging the pregnancy is decided by a council of doctors. In case of illness and the impossibility of performing 1st trimester screening, the risk of chromosomal aneuploidies in the fetus is assessed based on 2nd trimester screening. In case of COVID-19, it is recommended to postpone invasive diagnostics for at least 14 days.

The presence of COVID-19 is not an indication for delivery, except in cases requiring improvement of the pregnant woman's blood oxygenation level. Delivery at the height of the disease is associated with an increase in the maternal mortality rate and a large number of complications: worsening of the underlying disease and complications caused by it, development and progression of respiratory failure, occurrence of obstetric bleeding, intrapartum fetal death, postpartum purulent-septic complications. However, if it is impossible to eliminate hypoxia against the background of mechanical ventilation or with progression of respiratory failure, development of alveolar pulmonary edema, as well as with refractory septic shock, for vital indications in the interests of the mother and fetus, emergency abdominal delivery (cesarean section) is indicated with all necessary measures to prevent coagulopathic and hypotonic obstetric bleeding.

In the pregnancy period up to 20 weeks, an emergency caesarean section may not be performed, since the pregnant uterus at this stage does not affect cardiac output. In the pregnancy period of 20-23 weeks, an emergency caesarean section is performed to save the life of the mother, but not the fetus, and in the period of more than 24 weeks - to save the life of the mother and fetus.

In the event of spontaneous labor developing at the height of the disease (pneumonia), it is preferable to deliver the baby through the natural birth canal under monitoring of the condition of the mother and fetus.

The preferred method of pain relief is regional analgesia in the absence of contraindications. Antiviral, antibacterial, detoxification therapy, respiratory support are carried out according to indications.

In order to speed up the process of delivery in case of fetal distress, weak labor activity and/or deterioration of the woman's condition, vacuum extraction or obstetric forceps may be used.

Caesarean section is performed for standard obstetric indications. However, if hypoxia cannot be eliminated against the background of mechanical ventilation or if respiratory failure progresses, alveolar pulmonary edema develops, as well as in case of refractory septic shock, emergency abdominal delivery (cesarean section) is indicated for vital indications in the interests of the mother and fetus, with all necessary measures to prevent coagulopathic and hypotonic obstetric bleeding. In severe cases of COVID-19, the preferred approach is lower midline laparotomy.

Anesthetic support for cesarean section in severe cases of the disease: in the absence of signs of severe multiorgan dysfunction
insufficiency and changes in the hemostasis system due to the use of anticoagulant therapy (up to 2 points on the SOFA scale), it is possible to use regional methods of pain relief against the background of respiratory support; in case of severe multiple organ failure, total intravenous anesthesia with mechanical ventilation is used.

All patients, regardless of the gestational age, are recommended to take preventive measures against bleeding.

In all cases, the question of the time and method of delivery is decided individually.

The criteria for discharge from hospital for pregnant women and women in labor are:

- Normal body temperature for 3 days;
- Absence of symptoms of respiratory tract damage;
- Restoration of impaired laboratory parameters;
- Absence of obstetric complications;
- A single negative result of a laboratory test of nasopharyngeal and oropharyngeal swabs for the presence of SARS-CoV-2 RNA.

Tactics of newborn care in the context of the COVID-19 pandemic

Routing of high-risk newborns for developing COVID-19 is based on the identification of risk groups depending on the mother's infection.

A newborn is considered infected if the result of the biomaterial test for SARS-CoV-2 RNA by NAAT is positive, regardless of the presence or absence of a clinical picture.

Management of potentially COVID-19 infected newborns in the delivery room

Detailed information on the diagnosis, prevention and treatment of newborns is presented in the methodological recommendations of the Ministry of Health of Russia "Organization of medical care for pregnant women, women in labor, women in labor and newborns with a new coronavirus infection COVID-19" .

To be present at the birth and to move the child, there must be a pre-assigned medical and nursing team for the newborn, which is invited to the delivery room no earlier than the beginning of the pushing period or the beginning of anesthesia for a cesarean section, and waits for the birth of the child at a distance of at least 2 meters from the woman in labor. The use of PPE is mandatory.

The number of people providing care in the room should be minimized to reduce contact with the patient.

Delayed clamping of the umbilical cord is not recommended; mother-child contact is not recommended; the baby is not put to the breast to prevent postnatal infection, and is taken out of the delivery room as quickly as possible.

Depending on the results of laboratory testing for SARS-CoV-2, the following rules are recommended:

- if the mother or child has symptoms of an acute infectious disease (clinical suspicion of COVID-19), it is preferable to temporarily separate the mother and baby until laboratory test results are obtained and the clinical condition of the mother and/or child is stabilized;
- if the PCR test result for SARS-CoV-2 is positive for the mother but negative for the baby, it is preferable to temporarily separate the mother and baby, regardless of their clinical condition;
- If the PCR test results are positive for both the mother and the newborn, and their clinical condition does not require intensive care, it is preferable to organize their joint stay in the Mother and Child ward, subject to the conditions described below;
- If the mother's test result is negative and the mother and child are in stable condition, the newborn, including one infected with SARS-CoV-2, can be kept with the mother in the Mother and Child ward, subject to the conditions described below.

Clinical guidelines developed in various countries around the world during the first months of the COVID-19 pandemic included temporary physical separation of mother and child after birth to reduce the risk of postnatal SARS-CoV-2 infection of the child from a mother with COVID-19. However, subsequent observations of the health of such children, as well as a summary of international experience accumulated during the second and third waves of the pandemic, showed that, despite the effectiveness of this infection control measure, its costs associated with abandoning the practice of rooming-in, recommended by the World Health Organization to support effective breastfeeding, call into question its benefits.

Despite the lack of multicenter epidemiological studies in large cohorts and evidence based on them, there is currently evidence of fairly high effectiveness of such measures as wearing a medical mask, hand hygiene and disinfection of surrounding surfaces, as well as self-distancing between contacts with the baby, in preventing postnatal infection with SARS-CoV-2 in the child.

a SARS-CoV-2- infected mother from her newborn are currently not mandatory, and their routine use as an infection control measure for the spread of COVID-19 requires a differentiated approach and should depend on the mother's condition and her preferences.

At the same time, the joint stay of a woman infected with SARS-CoV-2 and her newborn in a

medical facility (hospital) requires a number of conditions (see below) that ensure the infectious safety of not only the mother and child, but also the staff of the medical facility, as well as compliance with the requirements for the provision of specialized medical care to both the woman and the newborn.

Depending on the clinical condition of the woman, it is possible to maintain lactation for subsequent breastfeeding of the child after the mother's recovery.

Primary and resuscitation care for a newborn is provided in a free delivery room or in a specially designated room, taking into account the minimization of the use of technologies that contribute to the formation of external infected aerosol (tracheal sanitation, ventilation with an Ambu bag, non-invasive administration of surfactant, etc.).

Diagnostic and treatment items (stethoscope, thermometer, etc.) and care products must be for individual use for each child and must be processed in accordance with the rules after transfer.

Doctors, nurses and other personnel who come into contact with the child must wear PPE.

After birth, the child must be taken out of the premises intended for pregnant women, women in labor, and women in labor with COVID-19 and isolated in a specially designated department (usually a department of a children's hospital). Transportation is carried out in a transport incubator, the staff uses PPE. Specially designated medical vehicles are subject to disinfection according to the rules for working with especially dangerous infections.

Nasal and oropharyngeal swabs are taken from the newborn baby for COVID-19 immediately after being moved from the delivery room or immediately after establishing postnatal contact with COVID-19- positive people from his environment. More

SARS-CoV-2 RNA test is repeated after 2-3 days. If both test results are negative, the child is considered not infected with SARS-CoV-2. If the child must be quarantined for epidemiological reasons, control tests of biological material from the nose, oropharynx, and stool are performed on the 10th-12th day of quarantine in order to decide whether it can be stopped by the 14th day. If one of the SARS-CoV-2 RNA test results is positive, the child is considered infected with this virus and further control tests are performed in accordance with the recommendations for the management of patients with COVID-19.

If necessary, medical care for the newborn is provided in accordance with clinical recommendations.

5.6. BASIC PRINCIPLES OF EMERGENCY TREATMENT

5.6.1. Indications for transfer to intensive care unit

According to Order No. 459n of the Ministry of Health of the Russian Federation dated May 18, 2020 , patients,

those in extremely serious condition are hospitalized in a structural unit of a medical organization for the treatment of COVID-19 in beds for patients in extremely serious condition requiring mechanical ventilation, based on the presence of two of the following criteria:

а) disturbance of consciousness;

б) SpO2 < 92% (with oxygen therapy);

в) Respiratory rate >35 min $^{-1}$.

5.6.2. Monitoring the patient's condition in the intensive care unit

In patients with COVID-19 in the ICU, it is recommended to routinely monitor SpO_2, respiratory rate, ECG with heart rate, non-invasive blood pressure measurement and body temperature. During mechanical ventilation, it is additionally recommended to monitor the gas composition and acid-base balance of arterial and venous blood, the oxygen content of the inspired mixture (FiO_2), the carbon dioxide content at the end of expiration ($EtCO_2$) and airway pressure. If PaO_2 determination is not available, it is recommended to use the SpO_2/FiO_2 indicator : if its maximum achievable value is less than or equal to 315, this indicates ARDS (including in patients without mechanical ventilation).

COVID-19- related shock, where technically feasible, comprehensive hemodynamic monitoring should be performed in accordance with the European Society of Critical Care Medicine (ESICM) guidelines. Whenever possible, it is recommended to monitor the response to volume loading.

use not static preload indicators (CVP, PAWP, ICGDO, etc.), but dynamic parameters - stroke volume variability (SVV) and pulse pressure variability (PPV), skin temperature, capillary refill time and/or lactate level. It is important to remember that SVV and PPV are only applicable to patients on mechanical ventilation without attempts at spontaneous breathing.

In case of multiple organ dysfunction against the background of COVID-19, it is recommended to monitor the daily and cumulative fluid balance, avoiding hyperhydration, and to quantitatively assess the severity of multiple organ failure using the SOFA scale (Table 6).

SOFA Organ Dysfunction Assessment Scale

Function	Indicator	Points				
		0	1	2	3	4
**CNS**	GCS score, points	15	13-14	10-12	6-9	< 6
**Oxygenation**	PaO_2/FiO_2, mm Hg.	> 400	< 400	< 300	< 200*	< 100*
**Hemodynamics**	Mean BP, mmHg or catecholamine doses, mcg/kg per min	> 70	< 70	Dop < 5 or Dob (any dose)	Dop 5.1-15 or Epi (Nor) < 0.1	Dof >15 or Epi (Nor) >0.1
**Hemostasis**	Platelets, x 10^9/µl	> 150	< 150	< 100	< 50	< 20
**Liver**	Bilirubin, µmol/l	< 20	20-32	33-101	102-204	> 204
**Kidneys**	Creatinine, mol/l	< 100	110-170	171-299	300-440	> 440
	Diuresis, ml/day				< 500	< 200

* against the background of respiratory support;
designations: Dop - dopamine, Dob - dobutamine, Epi - epinephrine (adrenaline), Nor - norepinephrine (norepinephrine).

5.6.3. Intensive care of acute respiratory failure

ARF is one of the most common complications of COVID-19. In patients with severe and extremely severe course (10-15%), fever persists after the 5th day of illness, symptoms of respiratory failure appear, infiltrative changes in the lungs (viral pneumonia), ARDS progress. Even with a mild course of COVID-19, infiltrative changes are visible in the CT scan of the lungs in most patients. Thus, SARS-CoV-2 was detected in nasopharyngeal washes in 59% of patients, and infiltrative changes on CT scan of the lungs were detected in 88% of patients with probable COVID-19.

Features of viral pneumonia and ARDS in COVID-19

Lung lesions in COVID-19 are characterized by severe arterial hypoxemia, often out of proportion to the volume of damage and the degree of decrease in lung tissue compliance, which is associated with the phenomenon of excessive perfusion

affected alveoli in COVID-19 (vasodilation) and ventilation-perfusion mismatch. When ARDS develops, these patients, as a rule, have no other reasons for tracheal intubation - impaired consciousness, muscle weakness (diaphragm dysfunction in critical illness polyneuromyopathy), unstable hemodynamics, intra-abdominal hypertension, low chest wall compliance, impaired respiratory biomechanics leading to increased work of breathing. As a result, in a significant proportion of such patients, compensation for hypoxemia and ARF is achieved by non-invasive methods - oxygen therapy and non-invasive mechanical ventilation (NIVL) even with a decrease in the PaO_2/FiO_2 index to 100 mm Hg, and in some - even lower.

In COVID-19, two types of lung damage have been described, which are essentially stages of the same process that lead to ARF:

- Focal lung lesion (viral pneumonia, earlier stage): normal or slightly reduced compliance of lung tissue, CT shows only areas of ground glass located subpleurally and along the interlobar fissures, low lung recruitability. Such patients are more likely to require oxygen therapy, and if unsuccessful, noninvasive mechanical ventilation (steps 1-2).
- Diffuse alveolar damage (corresponds to CT stages 3-4) (ARDS proper). The alveoli are filled with exudate, diffuse ventilation-perfusion ratio disorder, some alveoli may be collapsed, atelectasis of lung areas is possible, more pronounced in the dorsal parts. High-flow oxygen therapy, non-invasive and invasive mechanical ventilation in the prone position are indicated. With diffuse alveolar damage in patients with COVID-19, the probability of volutrauma (alveolar injury due to increased volume) is very high, therefore, the tidal volume should not be set at more than 6 ml/kg BMI (or the tidal volume should be monitored during non-invasive mechanical ventilation) and a moderate PEEP should be used (usually 8-10 cm H2O, less as the disease progresses).

Diffuse alveolar damage (ARDS) in COVID-19 is diagnosed on average on the 8th day from the onset of the disease; upon admission to the intensive care unit, the incidence of ARDS is about 60%, and the PaO_2/FiO_2 index is 136 (103-234) mm Hg.

In ARDS, patients with COVID-19 have a high incidence of hypercapnia, which persists

even with invasive mechanical ventilation due to an increase in alveolar dead space (pulmonary capillary microthrombosis, pulmonary embolism) and/or an increase in shunt (venous admixture), which leads to a large (above 5 mmHg) difference in CO_2 tension in arterial blood and at the end of expiration.

Gas exchange targets associated with improved outcome in ARDS:

- PaO_2 90-105 mm Hg or SpO_2 95-98%;
- $PaCO_2$ 35-50 mmHg, hypercapnia up to 70 mmHg may be used if normocapnia cannot be achieved with a tidal volume of 6 ml/kg BMI and a respiratory rate of 30 per minute

Algorithm for providing assistance in case of ODN

A step-by-step approach to respiratory therapy is recommended (diagram 1):

1 step - at SpO_2 <92% , start conventional O_2 therapy (through a face mask or nasal cannulas, preferably a mask with a dispensing bag) with a flow of up to 15 l/min to SpO_2 96-98 %; in patients with concomitant diseases (COPD, chronic heart failure), instead of step 1, go straight to step 2.

2 step (if step 1 is ineffective) – prone position for at least 12-16 hours per day with high-flow oxygenation (HFO), it is recommended to put on the patient a protective mask with a flow of 30-60 l/min or NIVL in the CPAP mode of 7-10 cm H_2O, and if visible work of breathing and participation of accessory respiratory muscles are maintained - NIVL in modes with a given level of inspiratory pressure (S, S/T, Pressure Support, BIPAP) of 14-24 cm H_2O (minimum level while maintaining patient comfort) and a minimum inspiratory fraction of oxygen to maintain the target SpO_2 value (usually 60-100%) (see the clinical guidelines of the Federation of Anesthesiologists and Resuscitators "Use of non-invasive ventilation of the lungs");

3 step - if hypoxemia persists (SpO_2 < 92%), there are signs of increased work of breathing (involvement of accessory muscles, frequent deep breathing), patient fatigue, impaired consciousness, unstable dynamics, the appearance of pressure "dips" of 2 or more cm H_2O below the CPAP level against the background of step 2, tracheal intubation and invasive mechanical ventilation in combination with the prone position are indicated (scheme 1). It is important that an isolated increase in respiratory rate to 30-35 per minute in the absence of the above signs is not an indication for tracheal intubation. If step 2 is ineffective in patients with COVID-19 , it is not recommended to delay tracheal intubation and the start of mechanical ventilation, since delaying tracheal intubation worsens the prognosis. It is important to remember that respiratory failure can progress extremely quickly.

Oxygen therapy

All systems for delivering oxygen to the patient's respiratory tract are divided into low-flow (oxygen flow up to 15 l/min) (nasal cannulas, simple oronasal masks, Venturi masks, oronasal masks with a reservoir bag) and high-flow (oxygen flow 30-60 l/min).

FiO_2 value depends not only on the oxygen flow, but also on the patient's condition (factors such as minute ventilation and the patient's inspiratory flow should be taken into account - the larger they are, the lower the resulting FiO_2.

Low-flow oxygen therapy is effective in mild to moderate hypoxemic ARF - with a

PaO2/FiO2 index of 300-150 mmHg (saturation in air without oxygen 75-93%) (step 1).

Low flow systems can be arranged in the following order of increasing resulting inspiratory oxygen fraction: nasal cannula -> simple oronasal masks -> Venturi masks -> oronasal masks with reservoir bag.

High-flow oxygen therapy is an oxygen therapy method that delivers a heated and humidified oxygen-air mixture through special nasal cannulas at high flow rates (up to 60 l / min), while it is possible to provide FiO_2 up to 100%. High-flow oxygen therapy is effective in severe hypoxemic ARF - with a PaO_2 / FiO_2 index of less than 150 mm Hg (saturation in air without oxygen below 75%) (step 2). One of the important physiological properties of high flow for patients with COVID-19 is the effect of washing the anatomical dead space, which leads to improved elimination of carbon dioxide and a decrease in the patient's work of breathing.

In order to accurately dose and save the oxygen-air mixture, patients breathing independently must use nasal-oral masks with a storage bag and a switching valve (the Ruben valve or modifications are available in a wide variety).

Non-invasive mechanical ventilation

Due to the low alveolar recruitability and the absence of significant extrapulmonary pathology in most patients with COVID-19, non-invasive mechanical ventilation is becoming one of the main methods of respiratory support, replacing invasive mechanical ventilation.

Non-invasive mechanical ventilation is indicated when low-flow and high-flow oxygen therapy (if used) is ineffective (step 2).

The use of NIVL is recommended only under the following conditions:

- Preservation of consciousness, stable hemodynamics;
- Ability to collaborate with staff;
- No claustrophobia (when using helmets);
- Preservation of the mechanism of expectoration of sputum.

NIVL is not recommended for:

- Absence of spontaneous breathing (apnea);
- Unstable hemodynamics (hypotension, myocardial ischemia or infarction, life-threatening arrhythmia, uncontrolled arterial hypertension);
- Inability to provide airway protection (impaired coughing and swallowing) and high risk of aspiration;
- Excessive bronchial secretion;
- Signs of impaired consciousness (excitement or depression of consciousness), inability of the patient to cooperate;
- Trauma or burns to the face, anatomical defects that prevent the installation of the mask;
- Inability of the patient to remove the mask from the face in case of vomiting;
- Active bleeding from the gastrointestinal tract;

- Upper airway obstruction;
- Discomfort from the mask.

Non-invasive mechanical ventilation can be performed using both special non-invasive mechanical ventilation devices (including devices for home NIVL) and universal mechanical ventilation devices with NIVL mode. It should be noted that the efficiency of using specialized NIVL devices is higher.

For non-invasive mechanical ventilation, special masks for NIVL (oronasal and full-face) as well as helmets (for CPAP and for NIVL, depending on the selected mode and type of circuit of the device) can be used.

When using oronasal and full-face NIVL masks, the following points should be taken into account:

- If a ventilator with a two-hose circuit and an exhalation valve is used (standard ventilator), then non-ventilated masks (without leakage at the “knee” of the mask) should be used;
- if a specialized NIVL apparatus with a single-hose circuit is used, then if there is an exhalation port in the circuit, masks with a non-ventilated “elbow” are used, and if there is no such port, masks with a ventilated “elbow” are used;
- the optimal leakage is about 30 l/min, if the leakage drops to less than 7 l/min, the mask should be loosened, if the leakage is 30-60 l/min, the mask should be tightly attached, if the leakage is more than 60 l/min, the mask should be changed;
- The optimal mask size should be selected according to the patient's face size; most adult patients will be suitable for size M masks;
  - If pressure sores develop from a mask, the mask should be changed to another type (mask rotation), for example, from an oronasal mask to a full-face mask; to prevent pressure sores, it is recommended to use mask rotation and special gel adhesive patches (protectors).

When using helmets for NIVL, attention should be paid to the correspondence between the helmet type, the circuit type, the selected ventilation mode and the type of ventilator.

The starting mode of NIVL is CPAP (EPAP) 8-10 cm H2O and an inspiratory oxygen fraction of 60%; if the respiratory muscles of the neck continue to work significantly against this background, the device should be switched to a pressure support mode (S, S/T, Pressure Support, BIPAP) with an IPAP pressure level of 14-22 cm H2O, selecting the minimum inspiratory pressure that maintains patient comfort and does not cause visible work of breathing for the patient. The FiO2 level should be selected based on the target oxygenation value.

When performing NIVL, it is necessary to monitor the value of the exhaled tidal volume, which should not exceed 9 ml/kg BMI with an oronasal and full-face mask, and with a helmet it can be 50-75% higher due to the high compliance and large volume of dead space of the helmet.

As the disease progresses, delayed tracheal intubation may lead to a worsening prognosis. As the degree of lung damage decreases and oxygen demand decreases, it is necessary to gradually reduce: first FiO_2, then the level of inspiratory pressure (IPAP, Pressure Support), then the level of CPAP (EPAP).

Prone and lateral decubitus positions in non-intubated patients

Patients with COVID-19 develop atelectasis in the dorsal parts of the lungs, which is why independent prone positioning (lying on the stomach) is highly effective even in non-intubated patients who receive oxygen therapy or NIVL. Prone positioning is performed at least twice a day (the optimal total time on the stomach is 12-16 hours per day). Early use of the prone position in combination with oxygen therapy or NIVL helps to avoid intubation in many patients.

The main mechanisms of action of the prone position:

- Straightening of gravity-dependent atelectasis;
- Improving ventilation-perfusion ratios;
- Improving drainage of respiratory secretions;
- With CPAP, ventilation is distributed more evenly.

Contraindications to independent prone position:

- Impaired consciousness (depression or agitation);
- Hypotension;
- Recent abdominal or chest surgery;
- Severe obesity;
- Massive bleeding;
- Spinal cord injuries;
- Rhythm disturbances that may require defibrillation and/or cardiac massage.

In patients with severe obesity and in pregnant women in the later stages of pregnancy, instead of the prone position, it is preferable to use the side-lying position, changing sides several times a day.

Indications for tracheal intubation (one criterion is sufficient):

- Hypoxemia (SpO_2 <92%) despite high-flow oxygen therapy or NIV in the prone position with FiO_2 100%;
- Patient fatigue due to VPO or NIVL in the prone position with FiO2 100 %;
- Increased visible chest wall excursions and/or involvement of accessory respiratory muscles despite VPO or NIV in the prone position with FiO2 100 %;
- Depression of consciousness or agitation;
- Respiratory arrest;
- Unstable hemodynamics.

Rational use of oxygen in respiratory support of patients

In order to reduce the deficit of medical oxygen, which is mostly consumed for inhalation of patients breathing independently, it is advisable to:

1. Instead of high-flow oxygen therapy, use low-flow oxygen therapy, including face masks with a disposable receiver bag, if they are sufficiently effective.
2. High-flow respiratory support should be performed only as prescribed and under the

supervision of an anesthesiologist-resuscitator using special equipment that allows for humidification, warming and strict dosing of oxygen in the inhaled mixture. Timely switch to non-invasive artificial ventilation technologies if high-flow is ineffective.

3. Limit the use of any high-oxygen systems (high-flow oxygen therapy, high-flow CPAP therapy using a Venturi valve, etc.) when oxygen is in short supply and consider using devices that consume less oxygen (low-flow oxygen therapy, noninvasive ventilation devices, including home CPAP devices).

4. Avoid oxygen consumption beyond the patient's control due to voluntary mask removal.

Invasive mechanical ventilation

Invasive mechanical ventilation in the treatment of COVID-19-associated ARF is used in case of ineffectiveness of non-invasive mechanical ventilation (step 3) or unavailability of the latter (step 2). Mechanical ventilation is aimed not only at ensuring adequate gas exchange, stabilization of collapsed alveoli, but also at minimizing potential patient-induced or iatrogenic lung injury. When using

invasive mechanical ventilation when non-invasive mechanical ventilation is ineffective, it should be borne in mind that in most cases, the use of auxiliary mechanical ventilation modes against the background of clear consciousness or moderate sedation after tracheal intubation can increase lung damage, therefore, in the first few hours after tracheal intubation, fully controlled mechanical ventilation modes should be used against the background of deep sedation and/or myoplegia. The strategy for the use of mechanical ventilation in COVID-19 is based on the temporary clinical guidelines of the Federation of Anesthesiologists and Resuscitators "Diagnosis and Intensive Care of Acute Respiratory Distress Syndrome" (2020) and the temporary guidelines of the Federation of Anesthesiologists and Resuscitators "Anesthesiological and Resuscitation Care of Patients with the New Coronavirus Infection COVID-19".

For mechanical ventilation in COVID-19 patients , a tidal volume of 6 ml/kg ideal body weight is recommended. Using a tidal volume greater than 6 ml/kg BMI leads to increased complications and mortality.

"Safe" ALV can be performed in both pressure-controlled (PC) and volume-controlled (VC) modes. In the latter case, it is desirable to use a descending form of inspiratory flow, which ensures better gas distribution in the lungs and lower pressure in the airways.

In patients with PaO_2/FiO_2 above 150 mm Hg, with reversal of myoplegia, it is recommended, if technically possible and in the absence of pathological respiratory rhythms, to switch to a fully assisted ventilation mode (in most devices - PSV) to improve gas distribution, prevent atelectasis and diaphragmatic atrophy.

In patients with COVID-19 , during mechanical ventilation, it is recommended to use PEEP depending on the alveolar recruitability and the risk of atelectasis. In patients with COVID-19, low alveolar recruitability is noted, the starting value of effective and safe PEEP is 8-10 cm H2O.

To assess recruitability, it is recommended to evaluate the difference between plateau pressure and PEEP (“driving pressure”) or the static compliance of the respiratory system: a decrease in the value of “driving pressure” in response to an increase in PEEP indicates recruitment of collapsed alveoli, and an increase in it indicates overinflation of already open alveoli.

The methodology for using PEEP is described in detail in the Clinical Guidelines of the Federation of Anesthesiologists and Resuscitators “Diagnostics and Intensive Care of Acute Respiratory Distress Syndrome”.

Routine use of alveolar recruitment is not recommended in COVID-19 due to low recruitability and high risk of acute cor pulmonale.

In patients with ARDS due to COVID-19, it is recommended to use a non-inverted inspiratory to expiratory ratio during mechanical ventilation for a more uniform
gas distribution in the lungs and reduction of the negative impact of mechanical ventilation on the right ventricular afterload; routine use of an inverse inspiratory to expiratory ratio (more than 1 to 1.2) is not recommended, while incomplete exhalation should be avoided (the expiratory flow before the start of inhalation of the device should reach zero). The respiratory rate should be adjusted to achieve normocapnia, but not more than 30 per minute. A time of 0.8-1.2 s is sufficient for inhalation.

Ventilation in the prone position (prone position) and in the lateral position

COVID-19 patients, the prone position is recommended for at least 16 hours per day to improve oxygenation and possibly reduce mortality. The patient should be placed on the stomach, with cushions under the chest and pelvis, as well as a face pillow (it is advisable to use special pillows for the prone position) so that the stomach does not exert excessive pressure on the diaphragm, and also does not create conditions for the development of facial pressure ulcers.

In cases of severe obesity, instead of the prone position during artificial ventilation, the position lying on the side with a change of side several times a day is preferable.

Complications with ventilation in the prone position:

- Kinks and dislocations of endotracheal tubes and venous catheters;
- Difficulty performing cardiopulmonary resuscitation;
- Development of neuritis of the peripheral nerves of the upper extremities;

Damage to the nose and eyes – facial and periorbital edema – develops in almost 100% of cases; keratoconjunctivitis requiring treatment develops in 20% of patients;

In the prone position, it is difficult to sanitize the oral cavity and trachea, and treat the eyes and face.

Criteria for termination of prone positioning: increase in PaO_2/FiO_2 >200 mmHg with PEEP <10 cm H_2O, maintained for ≥ 4 hours after the last session of prone positioning.

Drug-induced sedation and myoplegia during mechanical ventilation

When performing mechanical ventilation in patients with a PaO_2/FiO_2 index >200
mm Hg.
use a "light" level of sedation (-1...-2 points on the Richmond Agitation and Sedation Scale RASS). This strategy reduces the duration of respiratory support and improves outcome. It is also advisable to avoid the use of benzodiazepines for sedation.

In patients with PaO_2 / FiO_2 < 120 mmHg against a background of PEEP >5 cm H_2O, it is

recommended to use neuromuscular blockade, but only in the first 48 hours after intubation, which can lead to a decrease in ventilator-associated lung damage and reduced mortality. Muscle relaxants should not be used routinely for synchronization with the device.

Timing of tracheostomy

Early tracheostomy (within the first three days after intubation) is recommended due to the duration of respiratory support and the high probability of complications of orotracheal intubation (tube dislocation and unintentional extubation when turning into the prone position, obstruction of the tube, risk of nosocomial pneumonia).

Discontinuation of respiratory support

It is recommended to extend respiratory support up to 14 days or more even with positive dynamics of the oxygenating function of the lungs, since with COVID-19 , a repeated deterioration of the course of ARDS is possible; the average duration of mechanical ventilation in survivors is 14-21 days.

To improve outcomes and reduce the duration of respiratory support, it is recommended to use general and respiratory criteria for readiness to discontinue it.

Basic respiratory criteria:

- PaO_2/FiO_2 >300 mm Hg, i.e. SpO_2 during inhalation of air is 90% or more;
- Restoration of the cough reflex and cough impulse;
- Absence of bronchorrhea;
- Tobin's index (f/Vt) <105.

Additional respiratory criteria:

- Static compliance of the respiratory system >35 ml/cm H2O;
- Airway resistance <10 cm H2O/L/s;
- Negative pressure during inhalation is less than –20 cm H2O;
- Occlusion pressure of the circuit during inspiration for the first 100 ms (P0.1) 1-3 cm H2O;
- Decreased infiltration on chest radiograph (and/or CT);

General criteria for readiness to discontinue respiratory support:

- Absence of depression of consciousness and pathological breathing rhythms;
- Complete termination of the action of muscle relaxants and other drugs that depress respiration;
- Absence of signs of shock (marbling of the skin, white spot >3 s, cold extremities), life-threatening rhythm disturbances, hemodynamic stability.

To initiate weaning of respiratory support, all essential respiratory and general criteria for readiness to wean respiratory support must be present.

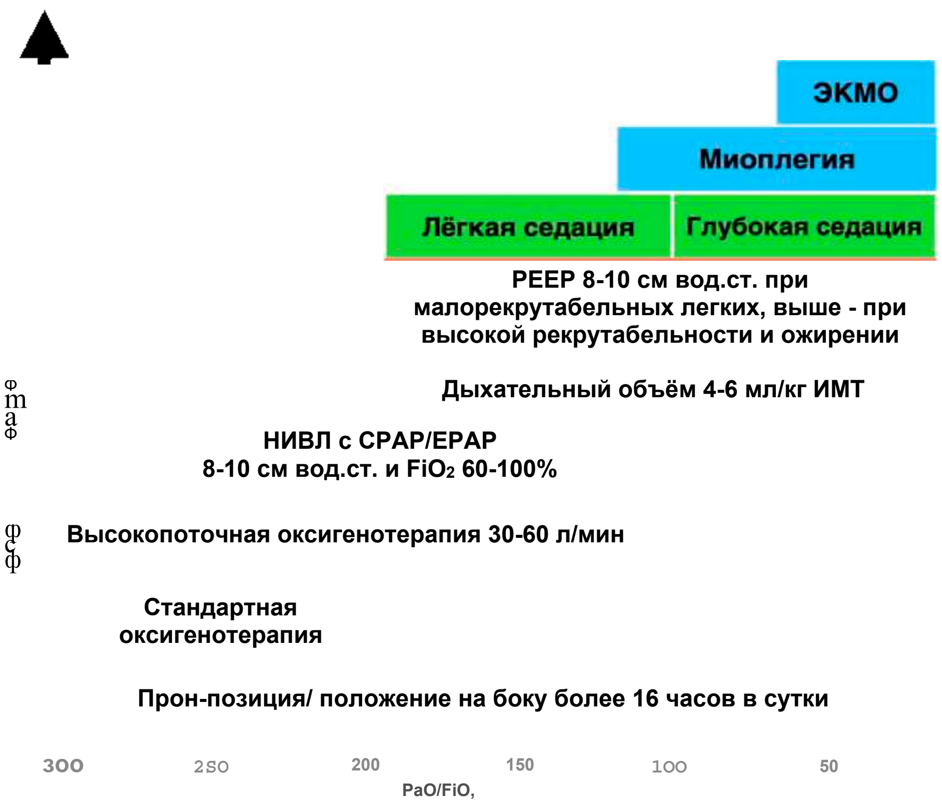

Figure 1: Step-by-step approach to choosing respiratory therapy for COVID-19

Helium-oxygen gas therapy

Currently, the effectiveness of the heated oxygen-helium mixture heliox (70% helium/30% oxygen) is being studied in the complex intensive care of patients in the initial stages of hypoxemia in COVID-19 to improve aeration of areas of the lungs with impaired bronchial patency.

Helium-oxygen gas mixture therapy is performed using special devices that provide effective and safe inhalation of thermal helium-oxygen mixture, allowing to change the percentage ratio of helium and oxygen, as well as the temperature at any time during one procedure. The device allows to create a homogeneous helium-oxygen mixture , repeatedly change and monitor the percentage of helium and oxygen, the temperature of the inhaled gas mixture during one procedure in order to determine the most effective mode for each patient, ensure that the actual composition of helium, oxygen and temperature correspond to the specified parameters during the procedure, monitor the necessary parameters (tidal volume, respiratory rate, saturation) during the procedure, form a uniform laminar flow of the gas mixture, and supply the necessary pharmaceuticals through a nebulizer built into the breathing circuit.

It should be noted that such therapy is limited by the inability to create FiO_2 above 30%, since helium therapy is only effective at concentrations exceeding 70%.

5.6.4. Extracorporeal membrane oxygenation (ECMO)

ECMO is performed in departments with experience in using this technology and by specialists who are proficient in the technique of cannulation of the main vessels and ECMO setup.

The decision to use ECMO should be made only after the ineffective use of the entire arsenal of standard therapy, including mechanical ventilation and prone positioning (prone position).

The success of the method is largely determined by effective pathogenetic therapy, prevention and treatment of bacterial and/or fungal complications, assessment of the prospects for restoration of lung function, and determination of contraindications. Most contraindications are not absolute and, undoubtedly, the use of ECMO in a patient even in a critical condition with some contraindications eliminates hypoxia and gives a chance for recovery.

An indication for venovenous ECMO is a result of 3 points or more when summing up the following indicators:

Polysegmental pneumonia CT 3-4, (1 point)

$PO_2/FiO_2 < 100$ for more than 12 hours (1 point)

$PO_2/FiO_2 < 80$ for more than 6 hours (2 points)

$PCO_2 > 60$ mmHg for more than 12 hours (2 points)

Provided that protective ventilation of the lungs is provided - PEEP>10 cm H_2O, DO 4-6 ml/kg, peak airway pressure <32 cm H_2O , the patient is in the prone position, and other causes of hypoxemia and/or hypercapnia are eliminated.

Contraindications to ECMO:

- Age ≥65 years
- Body mass index ≥40 kg/ m^2
- Artificial ventilation for more than 5 days
- SOFA score >12 points)
- Hemorrhagic stroke
- Active bleeding, inability to administer anticoagulant therapy
- Inability to achieve vascular access
- Severe damage to the central nervous system (Glasgow scale score <5 points)

5.6.5. Circulatory support and infusion therapy

In patients with COVID-19, it is recommended to adhere to conservative (restrictive) infusion therapy tactics with a dynamic assessment of its effectiveness based on the response of hemodynamic parameters, blood gas composition, lactate clearance and other parameters. As an initial infusion therapy, bolus administration of crystalloids of 500 ml should be used, depending on the response of hemodynamics and gas exchange. It is necessary to manage patients in zero or negative fluid balance with mandatory monitoring of diuresis and daily fluid balance. Diuretics and renal replacement therapy methods can be used to maintain a negative fluid balance.

In patients with hypotension (systolic BP <90 mmHg or mean BP <65 mmHg), screening

is recommended to identify possible additional foci of infection, including bacterial superinfection. When performing infusion loads to stabilize hemodynamics in such patients, preference should be given to balanced crystalloid preparations. Routine use of colloidal preparations is not recommended.

In patients with hypotension who do not require infusion load (with negative results of dynamic tests for infusion load) or infusion load is not accompanied by rapid stabilization of hemodynamics, it is recommended to start the administration of vasoactive drugs with the aim of initially maintaining mean BP within 65-75 mm Hg. In patients under 65 years of age, in the absence of cardiac pathology and signs of tissue hypoperfusion, it is permissible to maintain mean BP within 60-65 mm Hg. In elderly patients with concomitant arterial hypertension, to prevent acute kidney injury, it is advisable to maintain mean BP of 75-85 mm Hg.

Norepinephrine is recommended as the first-line vasoactive agent; if norepinephrine is not available, epinephrine. Dopamine can be used only in the absence of norepinephrine and epinephrine. In patients with COVID-19 and shock with evidence of myocardial dysfunction, manifested by increased cardiac filling pressures and decreased cardiac output, or with persistent signs of hypoperfusion despite achieving adequate preload and mean arterial pressure, dobutamine is recommended as an additional treatment. In patients with refractory shock (requirement for norepinephrine or epinephrine infusion at a dose of > 0.5 mcg/kg/min to maintain mean arterial pressure within

65-75 mmHg) is recommended in addition to vasopressor support
use low doses of glucocorticoids (hydrocortisone infusion 200 mg/day).

5.6.6. Treatment of patients with sepsis and septic shock

To screen patients with a high probability of developing sepsis, including consideration of early hospitalization in the intensive care unit, the Quick SOFA (qSOFA) criteria should be used , which allow one to assume the presence of sepsis based on clinical signs without laboratory tests:

- decreased level of consciousness to 13 or less points on the Glasgow Coma Scale;
- decrease in systolic blood pressure to less than 100 mm Hg;
- Respiratory rate 22 or more.

Each of the signs is given one point. In the case of two or three points, the probability of the presence of an infection in the sepsis variant is about 80%, exceeding the diagnostic value of the classic SOFA scale, with a higher risk of death by 3-14 times compared to the qSOFA index of less than two points.

An important differential criterion for the etiology of sepsis is a clinical blood test: in the case of bacterial genesis, leukocytosis is observed, and in the case of viral genesis, normo- or leukopenia and lymphopenia.

Tasks of intensive care for septic shock (except etiotropic treatment):

1. Hemodynamic support (optimization of $O_{2\ delivery}$)
2. Metabolic support (minimizing extraction deficits or increasing $O2_{consumption}$).

The key principle of intensive care in the early phase of septic shock is the immediate initiation of hemodynamic support in patients with hypotension or elevated serum lactate concentration (> 2 mmol/L). Infusion replacement therapy is recommended as the initial measure of hemodynamic stabilization. If initial infusion therapy is ineffective, vasopressors are administered according to the principles outlined in section 5.6.5.

Microcirculatory-mitochondrial distress in septic shock is largely responsible for tissue oxygen consumption disturbances. The target for intensive care is central venous oxygen saturation ($ScvO_2$) of 65-75%. In the absence of tissue hypoperfusion,
In cases of ischemic heart disease and bleeding, it is recommended to maintain hemoglobin (Hb) at a level of 70-90 g/l. Transfusion of red blood cell-containing components may be prescribed when the Hb concentration drops below 70 g/l.

5.6.7. Extracorporeal detoxification and hemocorrection

In patients with severe COVID-19 , the indication for extracorporeal treatment is progressive respiratory failure and/or multiple organ failure.

It is recommended to consider the possibility of using:

- Selective hemosorption of cytokines - in severe cases of the disease and progressive respiratory failure due to a cytokine storm that cannot be relieved by medication;
- Renal replacement therapy using membranes with increased adsorption capacity and a high cutoff point - to relieve cytokine storm and treat acute kidney injury;
- Plasma exchange with replacement of fresh frozen donor plasma (FFP) or selective plasma filtration in the absence of a sufficient volume of FFP – in the presence of signs of macrophage activation syndrome, DIC syndrome, thrombotic microangiopathy.

COVID-19 convalescent donors at a dose not exceeding 20 ml/kg per day.

In case of complications of the clinical course of COVID -19 with clinical manifestations of bacterial sepsis and septic shock, it is recommended to consider the use of selective hemosorption of lipopolysaccharides; renal replacement therapy is carried out according to generally accepted indications and methods.

5.6.8. Nutritional support

Early enteral nutrition (EN) is recommended for all patients with COVID-19 who are unable to ingest food on their own. It is necessary to aim to meet the daily requirements for energy (25-30 kcal/kg) and protein (1.2-1.5 g/kg). In cases of high risk of aspiration or intolerance to EN, parenteral nutrition should be administered.

Postpyloric access for EN is recommended in cases of gastric feeding intolerance or when there is a high risk of aspiration.

When performing EP in the prone position, it is necessary to raise the head end of the bed by 10-25° in order to reduce the risk of aspiration of gastric contents, facial edema and intra-abdominal hypertension.

Nutritional support should be delayed in refractory shock, uncontrolled life-threatening hypoxemia, hypercapnia or acidosis. Once shock is controlled, transition to full energy and protein support

It should be carried out gradually (over 3-5 days), especially in elderly patients who require mechanical ventilation and/or receive adrenergic drugs.

When providing nutritional support, it is recommended to correct hyperglycemia with insulin at blood glucose levels greater than 10 mmol/l.

In general, anesthesiology and resuscitation care for patients with the new coronavirus infection COVID-19 is recommended to be carried out in accordance with Methodological recommendations of the All-Russian public organization "Federation of Anesthesiologists and Resuscitators" (http://far.org.ru/newsfar/496- metreccovid19) .

5.7. SPECIAL PATIENT GROUPS

Patients with arterial hypertension

Since ACE2 is a functional receptor for SARS-CoV-2, it has been suggested that this may explain the high risk of death from COVID-19 in patients with arterial hypertension receiving drugs from the group of ACE inhibitors or angiotensin receptor blockers. These concerns were hypothetical and have not received practical confirmation. Moreover, there is indirect evidence of a possible protective effect of these drugs in COVID-19. Experts from the European Society of Cardiology published a statement that there is no data on the adverse effects of these drugs on the course of COVID-19 , and their use is strongly recommended to be continued.

Patients with hyperlipidemia

Statins have an immunomodulatory effect. If COVID-19 is confirmed , statin therapy is not stopped. If patients have not taken statins, then therapy is recommended for mild and moderate cases. During treatment of the infection, monitoring of liver enzymes and the risk of rhabdomyolysis is necessary.

Patients with acute coronary syndrome

Since non-specific increase in troponin levels is detected in COVID-19 , and cases of myocarditis have been described, a more thorough examination is required to confirm the diagnosis of acute coronary syndrome in the context of COVID-10 . In general, the tactics of managing patients with acute coronary syndrome should not differ from the standard one. Patients with acute coronary syndrome and suspected COVID-19 should be referred to hospitals that have the ability to perform percutaneous coronary intervention.

Patients with diabetes mellitus

Diabetes mellitus (DM) is a risk factor for severe pneumonia and septic course of viral infection associated with the development of multiple organ failure and an increased risk of complications and death. Patients with diabetes mellitus should carefully follow recommendations for the prevention of COVID-19. The use of alpha interferon drugs is recommended as a specific prevention. If COVID-19 is detected in patients with DM over 50 years of age, hospitalization is recommended.

In case of mild COVID-19 , a patient with diabetes is recommended to increase glycemic control every 4-6 hours with an expansion of the drinking regimen to 2-3 liters per day, taking into account concomitant diseases. It is necessary to continue current hypoglycemic therapy.

If glycemia increases, it is necessary to intensify diabetes therapy. If fasting glycemia is above 13 mmol/l, it is necessary to evaluate the level of ketones in the urine, start basal insulin therapy (for example, insulin isophane human genetically engineered insulin or a long-acting insulin analogue) or increase its dose (if the patient has previously received basal insulin therapy).

In mild cases of COVID-19, target fasting blood glucose levels are no more than 7 mmol/L.

In case of moderate COVID-19 and the appearance of respiratory symptoms:

- Glycemic monitoring is performed every 3-4 hours, urine ketones are monitored 1-2 times a day, and blood lactate levels are assessed;
- If glycemia is above 15.0 mmol/l or ketones appear in the urine or lactate levels in the blood increase, it is necessary to discontinue the use of non-insulin drugs (metformin, GLP-1 receptor agonists (GLP-1 ar), sodium-glucose cotransporter type 2 inhibitors (SGLT-2 inhibitors), drugs sulfonylureas) and start basal-bolus insulin therapy (with short-acting and long-acting insulin preparations).
- Target fasting blood glucose levels are no more than 7.5 mmol/L.

Severe COVID-19 is characterized by the progression of respiratory and multiple organ failure. Recommended:

- Target glycemia is determined by the severity of the patient's condition and the course of the disease;
- Stop taking all hypoglycemic drugs except insulin;
- Prescribe continuous intravenous administration of short-acting insulin using an infusion pump;
- Monitor glycemia hourly if glycemia is above 13.0 mmol/l or every 3 hours if glycemia is below 13.0 mmol/l to adjust the rate of insulin administration; monitor the content of ketones in urine and lactate in blood twice a day.

COVID-19 treatment in patients with diabetes:

1. Patients with diabetes are at high risk of developing a bacterial infection.
2. When prescribing GCS therapy, an increase in glycemia should be expected. It is necessary to conduct monitoring every 3 hours, increase the rate of insulin delivery according to glycemic control data (the insulin dose can be increased 2-3 times compared to the initial one).
3. Patients with diabetes are characterized by a more rapid development of ARDS. Earlier preemptive administration of monoclonal antibodies for the treatment of cytokine storm is justified. It is necessary to monitor clinical markers of interleukin inflammation for timely administration of therapy
4. There are high risks of developing hypercoagulation syndrome. Prescription of anticoagulants is mandatory when using the appropriate treatment regimens.

When discharging patients from hospital:

- Provide patients with insulin preparations during self-isolation;
- It is possible to resume taking metformin, GLP-1 arginine reuptake inhibitors, and NGLT2 inhibitors after 2 weeks in case of complete patient recovery.

- Continue anticoagulants until complete recovery.

Patients with chronic obstructive pulmonary disease

Chronic obstructive pulmonary disease (COPD) is a disease characterized by damage to the bronchial tree, mainly small bronchi, and the development of various forms of pulmonary emphysema, which leads to a decrease in the air flow rate in the respiratory tract and clinically - the occurrence of dyspnea. The main cause of the disease is smoking, in rare cases - other exogenous effects.

COVID-19 is detected in a patient with COPD, the development of respiratory failure may progress faster, which requires special monitoring of these patients and assessment of the level of gas exchange. During the treatment period, it is necessary to continue basic therapy with long-acting bronchodilators; if they have not been prescribed, they must be included in the general regimen. Inhaled GCS
should be used as metered-dose aerosols or powders. Nebulizer therapy should be avoided and used only for vital indications, with precautions taken to prevent the spread of infection through aerosols. Systemic GCS should be used according to the rules for the treatment of exacerbation of COPD.

Patients with bronchial asthma

If COVID-19 is detected in patients with bronchial asthma, basic therapy, including topical GCS, should be maintained in the same volume as before the disease. Treatment of exacerbations of bronchial asthma is carried out according to general rules. Restrictions on nebulizer therapy are the same as for COPD. If the patient has used biological therapy and needs to continue this treatment, since there are no contraindications for the administration of immunobiological therapy drugs.

Patients with tuberculosis

COVID-19 infection in patients with tuberculosis are not fully understood. There are scientific publications that the presence of tuberculosis infection, including latent, aggravates the course of COVID-19. In order to prevent the development of combined pathology and exclude tuberculosis infection at the initial stage of managing a patient with suspected COVID-19, it is necessary to conduct a tuberculosis examination simultaneously with testing for the SARS-CoV-2 virus. In patients with pneumonia caused by COVID-19, leukopenia and lymphopenia are noted in blood tests, which can be considered a risk factor for the transition of latent tuberculosis infection to active and classify the group of patients with COVID-19 as risk groups that require not only the exclusion of active tuberculosis, but also mandatory testing for latent tuberculosis infection during their hospital stay. After suffering COVID-19, many patients develop pronounced residual changes in the lungs in the form of fibrosis. It can be assumed that this category of patients has an increased risk of developing tuberculosis in the future. Thus, tuberculosis can occur before, simultaneously with, or after COVID-19. To detect tuberculosis infection, it is optimal to conduct laboratory tests for the release of gamma interferon, which require a single visit to the laboratory. Blood can be

taken simultaneously for testing for immunoglobulins to COVID-19 and for testing for tuberculosis. Given the high likelihood of lymphopenia in patients with COVID-19, it is advisable to test using an in vitro test to detect effector T cells that respond to stimulation with the Mycobacterium tuberculosis antigen, using the ELISPOT method . Studies have shown that under conditions of low lymphocyte counts

ELISPOT method is superior to others for detecting tuberculosis, regardless of age, gender and diet.

Patients with interstitial, rare and genetically determined lung diseases

Patients from these groups who become ill with COVID-19 should be monitored together with specialists in the specific pathology, taking into account possible complications, adverse drug interactions and the specifics of respiratory failure therapy.

Patients with immune-mediated inflammatory rheumatic diseases

Patients with immune-inflammatory rheumatic diseases (IIRD) are at risk for COVID-19 and an unfavorable course of the disease. Risk factors include old age, high doses of "antirheumatic" drugs, simultaneous use of several "antirheumatic" drugs, especially in combination with GCS, high disease activity, the presence of comorbid and multimorbid pathology. The high frequency of "cardiometabolic" and pulmonary comorbidity, characteristic of IIRD, in the case of COVID-19 may contribute to a decrease in the effectiveness of therapy for the underlying disease and complicate the diagnosis of the infection (lung damage).

It should also be borne in mind that SARS-CoV-2 infection can cause inflammation activation in IRR, and some clinical manifestations and laboratory abnormalities observed in COVID-19 can develop at the onset or during exacerbation of IRR: fever, arthralgia, fatigue, myalgia, cytopenia (primarily lymphopenia, less often anemia and thrombocytopenia), acute interstitial pneumonia ("ground glass"), myocarditis, venous thrombosis, livedo reticularis, increased concentrations of CRP, D-dimer, ferritin. Immune system pathology in IRR and concomitant comorbid diseases can aggravate the course of COVID-19 and increase the risk of developing cytokine storm syndrome.

During the COVID-19 pandemic , hospitalization of patients is possible only for urgent indications with the mandatory provision of laboratory data on the absence of SARS-CoV-2 infection.

The specific features of treating patients with IRR in the context of the COVID-19 pandemic are:

- In case of SARS-CoV-2 infection of patients with IRR, treatment with standard disease-modifying antirheumatic drugs (DMARDs) (methotrexate, leflunomide, azathioprine), genetically engineered biological drugs (inhibitors) should be temporarily (until complete recovery) interrupted.

  tumor necrosis factor-α, IL-6, IL-17, IL-12/23, IL-23, blockers

co-stimulation of T cells, anti-B cell drugs (rituximab, belimumab) and “targeted” DMARDs (Janus kinase and phosphodiesterase type 4 inhibitors) and consult a rheumatologist;

- It is recommended to continue taking 4-aminoquinoline drugs (or prescribe them in the absence of contraindications) and sulfasalazine;
- It is possible to use NSAIDs in low doses (ibuprofen, ketoprofen) and paracetamol as antipyretic drugs;
- It is not recommended to interrupt treatment with GCS, but the dose of the drug should be reduced as much as possible;
- During the COVID-19 pandemic , “planned” therapy with cyclophosphamide and anti-B-cell drugs (rituximab) should be interrupted and therapy with standard DMARDs (methotrexate, leflunomide), GIBAs and targeted DMARDs should not be initiated unless there is an absolute indication associated with the risk of developing urgent complications or irreversible damage to internal organs;
- In the absence of suspicion of SARS-CoV-2 infection and other contraindications, immunization with a vaccine against pneumococcal infection is recommended.

Patients with oncological diseases

Oncological diseases determine a more severe course and worse prognosis of COVID-19. Those at the highest risk are: patients currently receiving antitumor drug therapy or in the last 3 months; patients undergoing radiation therapy; patients with leukopenia and/or low immunoglobulin levels; patients after autologous and allogeneic bone marrow transplantation in the last 6 months; patients receiving immunosuppressive therapy; patients with certain types of hematopoietic and lymphoid tissue tumors, even if they are not currently undergoing treatment (chronic lymphocytic leukemia, lymphoma, myeloma, acute leukemia). In addition, factors of an unfavorable prognosis for COVID-19 in oncohematological patients include old age, poor somatic status (3-4 on the ECOG scale), tumor relapse or progression.

Patients with cancer should follow all recommendations for the prevention of COVID-19. It is advisable to minimize the length of the patient's stay in the hospital, consider the possibility of conducting antitumor drug treatment on an outpatient basis, using tablets, if this does not worsen the course of the oncological process. When choosing a chemotherapy regimen that involves the parenteral route of drug administration, it is recommended to give preference to regimens with long intervals between administrations. It is necessary to avoid prescribing antitumor drugs that have pulmonary
toxicity, and consider the possibility of temporarily interrupting antitumor drug treatment, transferring to observation or supportive therapy, provided that a reduction in the number of chemotherapy courses does not lead to a worsening of the oncological prognosis.

Patients with cancer and laboratory-confirmed COVID-19 are hospitalized for the same indications as patients without cancer. When treating COVID-19 on an outpatient basis, it is necessary to ensure careful monitoring of the patient's condition with the possibility of urgent hospitalization if the condition worsens or indications appear. Patients with cancer and laboratory-confirmed/suspected COVID-19 and the presence of signs of lung tissue damage need to undergo computed tomography of the chest organs (assessment of the lung tissue condition).

Patients with laboratory-confirmed COVID-19 and/or characteristic clinical and radiographic features of COVID-19 should discontinue antitumor drug therapy until significant clinical improvement, pneumonia regression, and a negative PCR result. It is acceptable to resume antitumor drug therapy under the above conditions and persistent residual changes in the lung tissue related to postpneumonic changes. In all cases, it is necessary to consider the possibility of transferring the patient to a less toxic antitumor drug regimen or temporarily interrupting treatment in cases where this is possible in accordance with the oncological prognosis.

In some cases (progression of an oncohematological disease requiring immediate initiation/continuation of antitumor drug therapy for vital indications), chemotherapy may be administered with the preferred use of the least toxic treatment programs.

Treatment of oncological/oncohematological patients with febrile neutropenia (as a consequence of chemotherapy) and COVID-19 is carried out in accordance with the accepted protocols for the management of patients with febrile neutropenia. It is necessary to approach the appointment of colony-stimulating factors with extreme caution, which have the potential to potentiate the "cytokine storm".

Despite the higher risk of developing secondary infectious complications in patients with cancer and a high risk of severe COVID-19, treatment of the new coronavirus infection is carried out in accordance with general recommendations for patients with COVID-19, but with more careful monitoring of the patient's condition, control of the level of CRP and other markers of systemic inflammation.

Patients with chronic kidney disease

According to WHO, every 9th person in the world has chronic kidney disease (CKD) and this problem is reaching the level of a non-infectious epidemic.

kidney disease is the most common variant of non-pulmonary SARS-CoV-2 damage due to the presence of ACE2 in the organ. Frequent involvement of the kidneys in the pathological process is characterized by a wide range of manifestations - from mild proteinuria and hematuria to progressive acute kidney injury (AKI), requiring the use of renal replacement therapy.

The incidence of AKI varies from 5.1 to 27% of cases and is an independent risk factor for death, increasing its probability by 1.9-4.4 times depending on the stage. This complication is observed in 68% of patients with ARDS. If mechanical ventilation is required, the probability of AKI increases by 10.7 times, and the existing underlying kidney disease, against the background of intoxication syndrome caused by COVID-19, can accelerate the progression of renal failure.

The use of drugs for the treatment of COVID-19, with a probable nephrotoxic effect in patients with CKD, requires constant monitoring of renal function (creatinine, urea, potassium and sodium concentration in the blood). The group of particularly high risk of infection and the course of SARS-CoV-2 includes patients receiving RRT in the form of programmed hemodialysis for the terminal stage of CKD, due to the impossibility of isolation and the need to be present in dialysis centers to receive a life-saving procedure at least 12 times a month. Moreover, hemodialysis patients, as the cause of the terminal stage of renal failure, have diabetes mellitus, arterial hypertension, systemic diseases with lung and kidney damage, oncohematological diseases, immunobacterial kidney damage, immunodeficiency of various origins, including those caused by uremia, nutritional status disorders that complicate the course and aggravate the prognosis of a new coronavirus infection. Also a special group includes organ transplant recipients receiving chemotherapy.

As a preventive measure against COVID-19 infection in patients receiving program hemodialysis treatment, strict routing is required both in case of suspected COVID-19 and in case of signs of COVID-19 disease. In the first case, it is necessary to transfer patients to observational dialysis in a dialysis center, excluding contacts with patients without suspicion and with signs of COVID-19. There is no specific prevention for patients with CKD, therefore, patients with CKD stages 4-5, especially those who need dialysis treatment, need to be hospitalized in a specialized hospital with a hemodialysis department.

Hospitalization of patients with CKD stages 4-5 is indicated if COVID-19 is detected. Patients with stage 3 CKD and those receiving dialysis treatment (peritoneal, hemodialysis) are subject to mandatory hospitalization.

COVID-19 treatment in patients with end-stage chronic renal failure:

1. Patients receiving hemodialysis treatment require constant and mandatory monitoring of volume status in order to minimize the "wet" lung syndrome.
2. An earlier start of renal replacement therapy is necessary (SCF less than 25 ml/min) taking into account possible hypervolemia and progression of uremic intoxication against the background of an active inflammatory process.
3. Patients on peritoneal dialysis with moderate COVID-19 who require prone positioning and precise and constant correction of volume status are temporarily transferred to hemodialysis.
4. Patients with terminal stage CRF are at high risk of developing bacterial infection, relapse and decompensation of the underlying disease, and therefore it is necessary to exclude activation and decompensation of the underlying disease.
5. When prescribing GCS therapy, one should expect an increase in glycemia, increased arterial hypertension, and hypervolemia. It is necessary to conduct monitoring every 3 hours, increase the rate of insulin delivery according to glycemic control data (the insulin dose can be increased 2-3 times compared to the initial dose) and adhere to a strict salt-free diet to correct arterial hypertension and hyperhydration against the background of GCS use.
6. Patients with CKD are characterized by a more rapid development of ARDS. Early preemptive administration of monoclonal antibodies for the treatment of cytokine storm at minimally advanced stages of lung damage is absolutely justified. It is necessary to monitor clinical markers of interleukin inflammation for timely administration of therapy.
7. There are high risks of developing hypercoagulation syndrome. Prescription of anticoagulants is mandatory when using the appropriate treatment regimens.
8. Patients who are recipients of transplanted organs require discontinuation of cytostatic therapy (mycophenolates and azathioprine) at the onset of the disease with a two-fold increase in the dose of GCS and correction in the form of a decrease to 2-2.5 times the initial dose of calcineurin inhibitors (tacrolimus, cyclosporine) with constant monitoring of their concentration in the blood.
9. Correction of hypocalcemia in patients with end-stage chronic renal failure receiving programmed dialysis treatment.

When discharging patients with CKD from the hospital:

1 When discharging patients receiving program hemodialysis treatment, provision of 2-week isolation shifts of hemodialysis until a negative laboratory test result for the presence of SARS-CoV-2 RNA is obtained. Continuation of anticoagulant administration until complete recovery under the control of fibrinogen levels, due to a significant decrease in the level of the latter after regression of disease activity.

5.8. MONITORING CLINICAL AND LABORATORY INDICATORS

An important circumstance when providing medical care to patients with COVID-19 or suspected COVID-19 is the assessment of the dynamics of clinical and laboratory parameters for the timely prescription of drugs and timely correction of therapy.

Clinical signs requiring monitoring:

- T of the body (monitoring daily at least twice a day in the morning and evening hours), the height of the increase in t of the body, the frequency of its rises during the day, the duration of the increase are assessed. Particular attention should be paid to episodes repeated increase in body temperature after normalization for 1 day or more.
- The respiratory rate is assessed daily; if the respiratory rate increases, it is necessary focus not only on the standard normal values of the indicator, but also on the increase in the indicator compared to the initial RR. With the development or increase of signs of respiratory failure, it is necessary to carefully monitor SpO_2 . If the RR increases to more than 22 per minute during treatment at home, it is necessary to decide on hospitalization of the patient.
- SpO2 is assessed daily (once in hospitalized patients) in three days, when the indicator decreases to a level of ≤93%, oxygen supplementation is required.
- For linear departments, SpO_2 during oxygen therapy should be ≥ 92%, if the indicator is lower, the prone position is indicated. If SpO_2 in the prone position is < 92%, a resuscitator consultation is indicated within an hour. When conducting oxygen therapy, it is indicated to briefly stop it at least once every 2 hours, if a decrease in SpO_2 < 85% is observed, a call to a resuscitator is indicated within an hour, if SpO_2 decreases to 80% or less, an emergency call to a resuscitator is indicated.

Laboratory parameters requiring monitoring:

- Levels of leukocytes, neutrophils, lymphocytes, platelets;
- Activity of ALT, AST, lactate dehydrogenase, CRP, glucose levels;
- Prothrombin time;
- Fibrinogen level;
- According to indications: levels of ferritin, D-dimer, IL-6, troponin; number of T- and B-lymphocytes; NT-proBNP, procalcitonin.

Instrumental signs requiring monitoring are the nature and area of lung damage on chest CT (as indicated).

The required volume of laboratory and instrumental examination depending on the clinical manifestations of the disease is presented in Appendix 2-1 . Laboratory monitoring of patients with COVID-19/suspected COVID-19 depending on the severity of the condition is presented in Appendix 2-2 .

5.9. MEDICAL REHABILITATION IN PROVIDING SPECIALISED MEDICAL CARE TO PATIENTS WITH COVID-19

Detailed information on medical rehabilitation of patients is presented in the methodological recommendations of the Ministry of Health of Russia " Medical rehabilitation in new coronavirus infection (COVID-19) » .

It is recommended to start medical rehabilitation of patients with coronavirus pneumonia in intensive care units when the patient's condition stabilizes and continue them at home after completion of inpatient treatment. It is recommended to organize medical rehabilitation activities in 4-level medical organizations, taking into account the specifics of providing assistance to patients with coronavirus pneumonia at 3 stages.

Medical rehabilitation activities at the 1st stage should include the provision of medical care for medical rehabilitation in intensive care units and infectious/therapeutic departments organized for patients with the new coronavirus infection COVID-19, in medical organizations by specialists of a multidisciplinary rehabilitation team who have undergone special training in weaning from mechanical ventilation, respiratory rehabilitation, nutritional support, restoration of tolerance to physical activity, maintenance and management of patients with the consequences of PIT syndrome, the formation of motivation to continue rehabilitation measures, compliance with the anti-epidemic regime and a healthy lifestyle.

The 2nd stage of medical rehabilitation is recommended to be organized in medical rehabilitation departments for patients with somatic diseases and conditions.

The 3rd stage of medical rehabilitation is recommended to be organized in the medical rehabilitation departments of the day hospital, outpatient medical rehabilitation departments for patients with somatic diseases and conditions of medical organizations in accordance with the Procedure for organizing medical rehabilitation, as well as at home using telemedicine technologies upon referral from the medical commission of the medical organization that provided assistance to the patient at the 1st or 2nd stage or the medical commission of the polyclinic implementing secondary prevention measures.

A repeated rehabilitation course should be carried out for patients who completed a rehabilitation course more than 1 year ago.

5.10. PROCEDURE FOR DISCHARGE (TRANSFER) OF PATIENTS FROM A MEDICAL ORGANIZATION

Patients diagnosed with the new coronavirus infection COVID-19 aged 18 years and older (hereinafter referred to as patients) admitted to a structural unit of a medical organization for the treatment of COVID-19 type I created for the hospitalization of patients in severe and extremely severe condition, as well as patients belonging to the risk group (age over 65 years, the presence of concomitant diseases and conditions: arterial hypertension, chronic heart failure, oncological diseases, hypercoagulability, DIC syndrome, acute coronary syndrome, diabetes mellitus, liver cirrhosis, long-term use of steroids and biological therapy for inflammatory bowel diseases, rheumatoid arthritis, patients receiving hemodialysis sessions or peritoneal dialysis, the presence of immunodeficiency states, including patients with HIV infection without antiretroviral therapy and patients receiving chemotherapy) are transferred to continue treatment in inpatient settings to

beds for patients on follow-up treatment (to a structural unit of a medical organization for the treatment of COVID-19 type II for the hospitalization of patients transferred from a structural unit of a medical organization for the treatment of COVID-19 type I to continue treatment, and patients in mild to moderate condition, living in a dormitory, communal apartment, social service institutions with round-the-clock stay and accommodation facilities providing hotel services, with persons over 65 years of age, with persons suffering from chronic diseases of the bronchopulmonary, cardiovascular and endocrine systems who are unable to stay in a separate room at the place of stay, out-of-town patients undergoing inpatient treatment in other medical organizations with a positive test result for COVID-19), based on the presence of the following criteria:

а) persistent improvement of the clinical picture;
б) blood oxygen saturation level in air ≥ 93%;
в) body temperature < 37.5 °C;
г) C-reactive protein level < 30 mg/l;
д) blood lymphocyte level > 1 x 10^9/l.

Patients admitted to a structural unit of a medical organization for the treatment of COVID-19 type I are discharged to continue treatment on an outpatient basis based on the presence of the following criteria:

а) persistent improvement of the clinical picture;
б) blood oxygen saturation level in air ≥ 95%;
в) body temperature < 37.5 °C;
г) C-reactive protein level < 10 mg/l;
д) blood lymphocyte level > 1.2 x 10^9/l.

If the above criteria are met, the patient is transferred to continue treatment in an inpatient setting in beds for patients undergoing follow-up treatment, or the patient is discharged, with the exception of patients specified in paragraph 8 of Appendix No. 12 to the Order of the Ministry of Health of Russia dated March 19, 2020 No. 198n, for continuation of treatment on an outpatient basis may be carried out until a negative result of laboratory tests of biological material for the presence of the new coronavirus infection COVID-19 is received.

When discharging or transferring a patient, in the absence of a negative laboratory test result for the biological material for the presence of the new coronavirus infection COVID-19, his transportation is carried out by specially designated or ambulance transport, provided that the patient, driver and accompanying person use personal protective equipment.

After the patient has been transported, the interior surfaces and body of the ambulance are disinfected at a specially designated site.

Information about the patient's discharge from a medical organization providing inpatient medical care is transferred to the medical organization in which the patient will receive outpatient

medical care and rehabilitation.

When providing medical care to a patient on an outpatient basis, he must be provided with:

daily medical monitoring, including remote monitoring;

conducting a laboratory test using the polymerase chain reaction method for the presence of the COVID-19 pathogen.

Radiography and/or CT before discharge to assess the dynamics of pneumonia are not mandatory procedures, but may be prescribed by the attending physician according to clinical indications.

Radiological criteria for regression of pathological changes:

reduction of “ground glass” zones; new “ground glass” zones of no more than 25% of the transverse size of the hemothorax are allowed;

reduction in the volume of previously visible consolidation zones;

residual parenchymal compactions variable in extent and location;

absence of pleural effusion associated with COVID-19.

A patient is considered recovered based on the presence of the following criteria:

а) $SpO2 > 96\%$;

б) $T < 37.2$ °C;

в) a single negative result of a laboratory test using the polymerase chain reaction method for the presence of the COVID-19 pathogen.

If a positive result is obtained from a laboratory test using the polymerase chain reaction method for the presence of the COVID-19 pathogen , the next laboratory test is carried out no earlier than 3 calendar days later.

In the event of a negative laboratory test result using the polymerase chain reaction method for the presence of the COVID-19 pathogen and the presence of the above criteria, patients are discharged from the medical organization for the treatment of COVID-19, and in cases of medical indications, they are discharged (transferred) to continue treatment and medical rehabilitation in outpatient and (or) inpatient settings in medical organizations of the corresponding profile.

5.11. FEATURES OF DISPENSARY OBSERVATION AND IN-DEPTH DISPENSARY OF CITIZENS WHO HAVE SUFFERED THE NEW CORONAVIRUS INFECTION COVID-19

Patients who have undergone long-term non-invasive and/or artificial ventilation and who had signs of significant functional/organic disorders upon discharge are recommended to have a remote consultation 4 weeks after discharge from the medical organization to assess the general condition, identify depression, symptoms suspicious for thromboembolism or other syndromes and diseases requiring attention.

8 weeks after discharge, it is recommended to visit a doctor and undergo instrumental examinations (as indicated):

1. chest radiography * ;
2. spirography * ;

3. measurement of blood oxygen saturation (saturation) at rest and under load (it is possible to conduct a 6-minute walk test with determination of saturation before and after the test) * ;

If pathology is detected, a CT scan of the lungs is performed.

4. echocardiography (EchoCG);
5. other research methods (diffusion test, arterial blood gases, etc.).

Further tactics of dispensary observation are determined by the doctor depending on the results of the examination and survey.

In the absence of complaints and pathological changes based on the research results, further dispensary observation is carried out in accordance with the Order of the Ministry of Health of Russia No. 173n dated March 29, 2019 with determination of saturation and radiography of the lungs.

If pathological changes are detected on CT of the lungs (signs of pulmonary fibrosis, interstitial lung disease, vasculitis), a referral to a specialist (pulmonologist) is recommended. If there are no pathological changes on CT of the lungs, but the patient has complaints or changes in the results of other studies, it is recommended to conduct a differential diagnosis with other diseases/conditions.

Patients with mild or moderate pneumonia who did not require treatment in the intensive care unit (including patients who were treated on an outpatient basis) are monitored in accordance with the order of the Russian Ministry of Health dated March 29, 2019 No. 173n "On approval of the procedure for conducting dispensary observation of adults" with determination of saturation and chest X-ray. If during inpatient treatment a suspicion of a malignant neoplasm in the lungs is detected, it is recommended to conduct a repeat chest X-ray 6 weeks after discharge, if necessary, refer the patient for a CT scan of the lungs and consult with an oncologist.

If the patient has persistent chest X-ray or CT abnormalities at hospital discharge, an imaging study (chest X-ray, CT) is recommended 8 weeks after the last chest CT and/or chest X-ray.

If pathological changes are detected on the radiograph:

- conducting spirography (recording and subsequent clinical consultation by a specialist can be performed remotely) * ;
- measurement of blood oxygen saturation (saturation) at rest and under load (it is possible to conduct a 6-minute walk test with determination of saturation before and after the test) * ;
- EchoCG * ;
- If PE is suspected, it is recommended to immediately perform CT angiography of the pulmonary arteries;
- if pulmonary fibrosis, interstitial lung diseases are suspected – high-resolution CT (and diffusion test).

If pathological changes are detected on CT of the lungs (signs of interstitial lung diseases, pulmonary vasculitis, pulmonary hypertension), a referral to a specialist (pulmonologist, cardiologist) is recommended.

If pathology is detected, perform a CT scan of the lungs if an X-ray of the lungs was previously performed.

If pathological changes are not detected, but the patient has complaints or changes in the results of

other studies, it is recommended to conduct a differential diagnosis with other diseases/conditions.

During dispensary observation, it is necessary to assess psychosocial risk factors, including identifying symptoms of anxiety and depression using validated questionnaires (for example, the Hospital Anxiety and Depression Scale), followed by correction of the identified deviations, if necessary, with the involvement of a psychologist, psychiatrist, and social workers.

Patients should be advised that if respiratory symptoms progress or develop new ones before the scheduled review date, they should seek medical attention.

In order to organize the in-depth medical examination of citizens who have recovered from the new coronavirus infection COVID-19, a medical organization, based on information from regional information systems of compulsory medical insurance integrated with the state information system of compulsory medical insurance, compiles a nominal list of citizens subject to in-depth medical examination (hereinafter referred to as the list of citizens), which indicates the following information:

а) last name, first name, patronymic (if any), age (date, month, year of birth);

б) citizen's compulsory medical insurance policy number;

в) the category to which the citizen belongs;

г) planned date and place of the in-depth medical examination.

The list of citizens is subject to monthly updating by the medical organization.

The planned date for conducting an in-depth medical examination is set no earlier than 60 calendar days after the recovery of a citizen who has had the new coronavirus infection COVID-19, when providing him with medical care on an outpatient basis or in a hospital setting.

The categories of citizens undergoing in-depth medical examination on a priority basis are defined in the Appendix to the Procedure for referring citizens for in-depth medical examination, including categories of citizens undergoing in-depth medical examination on a priority basis, approved by Order of the Ministry of Health of Russia dated 01.07.2021 No. 698n (hereinafter referred to as the Procedure).

Based on the list of citizens, the medical organization forms and maintains a calendar plan for conducting an in-depth medical examination, indicating examinations by specialist doctors, laboratory, instrumental and other studies, dates and places of their implementation, the number of citizens (hereinafter referred to as the calendar plan).

When planning the date of an in-depth medical examination of a citizen, drawing up a list of citizens subject to in-depth medical examination, as well as when updating the said list, the category to which the citizen belongs is taken into account.

If a citizen is suspected of having a disease (condition) the diagnosis of which cannot be established during the examinations and other medical interventions included in stage I , the in-depth medical examination is completed if the examinations and other medical interventions included in stage II of the in-depth medical examination are carried out in accordance with the list of examinations and other medical interventions carried out within the framework of

in-depth medical examination, given in Appendix No. 4 to the Program of State Guarantees for Free Provision of Medical Care to Citizens (introduced by Decree of the Government of the Russian Federation dated June 18, 2021 No. 927) (hereinafter referred to as the List of Studies).

In accordance with the list of studies, the first stage of in-depth medical examination is carried out in order to identify signs of the development of chronic non-communicable diseases, risk factors for their development in citizens who have had the new coronavirus infection COVID-19, as well as to determine medical indications for additional examinations and examinations by specialist doctors to clarify the diagnosis of the disease (condition) at the second stage of the medical examination and includes:

а) measurement of blood oxygen saturation (saturation) at rest;

б) a 6-minute walk test (with an initial blood oxygen saturation of 95 percent or more, combined with the citizen complaining of shortness of breath, swelling that has appeared for the first time or has increased in intensity);

в) conducting spirometry or spirography;

г) general (clinical) blood test, detailed;

д) biochemical blood test (including studies of cholesterol levels, low-density lipoprotein levels, C -reactive protein, determination of alanine aminotransferase activity in the blood, determination of aspartate aminotransferase activity in the blood, determination of lactate dehydrogenase activity in the blood, study of creatinine levels in the blood);

е) determination of the concentration of D-dimer in the blood of citizens who have suffered from moderate or severe cases of the new coronavirus infection COVID-19;

ж) performing chest X-ray (if not previously performed within a year);

з) appointment (examination) with a general practitioner (district therapist, general practitioner).

II of in-depth medical examination is carried out for the purpose of additional examination and clarification of the diagnosis of the disease (condition) and includes:

а) echocardiography (if the saturation level at rest is 94 percent or lower, as well as based on the results of the 6-minute walk test);

б) performing a computed tomography scan of the lungs (in the case of a saturation level at rest of 94 percent or less, as well as based on the results of a 6-minute walk test);

в) duplex scanning of the veins of the lower extremities (if indicated based on the results of determining the concentration of D-dimer in the blood).

If, during an in-depth medical examination, a citizen is identified with medical indications for examinations (consultations) by specialist doctors, studies and other medical interventions that are not included in the scope of in-depth medical examination in accordance with this Procedure, they are prescribed and performed in accordance with the provisions of the procedures for the provision of medical care in the profile of the identified or suspected disease (condition), taking into account the standards of medical care, as well as on the basis of clinical recommendations.

6. FEATURES OF CARE FOR CHILDREN WITH COVID-19

Detailed information on diagnostics, prevention and treatment of children is presented in the methodological recommendations of the Russian Ministry of Health “Features clinical manifestations and treatment of the disease caused by the new coronavirus infection (COVID-19) in children . "

According to available data, children get sick less often, with less pronounced clinical symptoms, require hospitalization less often, and their illness is milder, which, however, does not exclude cases of severe progression.

Available data to date indicate that children account for up to 10% of those infected with SARS-CoV-2 and up to 2% of patients diagnosed with clinical cases of COVID-19.

In the Russian Federation, children account for 7.6% of registered cases of COVID-19, the number and age structure of manifest diseases in children are currently unknown. The disease is extremely rare in newborns, and intrauterine transmission of the infection has not been proven.

In children, risk factors differ significantly, as only 9% of cases of SARS-CoV-2 infection are associated with travel to other countries and 91% of cases had local contacts, mainly in family outbreaks.

Children have a milder course of the disease compared to adults, the development of viral pneumonia is not typical, the symptoms are less pronounced, and fatal outcomes are extremely rare. In children, as in adults, fever and respiratory syndrome predominate, but lymphopenia and inflammatory markers are less pronounced.

The incubation period in children ranges from 2 to 10 days, most often 2 days.

The clinical symptoms of COVID-19 in children correspond to the clinical picture of acute respiratory viral infections caused by other viruses: fever, cough, sore throat, sneezing, weakness, myalgia. The severity of the febrile reaction may vary: fever up to 38 ° C is observed in half of sick children, in a third of children an increase in body temperature from 38.1 to 39.0 ° C is recorded.

The accumulated experience of observing manifest cases of COVID-19 in children shows that the characteristic combination of fever, cough and shortness of breath occurs in only 73% (in adults - 93%). Comparison of the frequency of individual symptoms also shows their predominance in adults. According to American researchers, only 56% of pediatric patients reported fever, 54% cough and 13% shortness of breath, compared with 71%, 80% and 43%, respectively, among patients aged 18-64 years. In children, not only are clinical manifestations of intoxication (headache 28% versus 58% in adults; myalgia - 23% versus 61% in adults) and respiratory failure less common, but also gastrointestinal manifestations
(nausea/vomiting – 11% vs. 16%; abdominal pain – 5.8% vs. 12%; diarrhea – 13% vs. 31%). Tachycardia is observed in half of hospitalized children, tachypnea – in a third. Children rarely experience a decrease in $SpO_2 < 92\%$.

According to various data, from 5.7% to 20% of children with COVID-19 were hospitalized, the rest were treated on an outpatient basis, including patients with mild, subclinical and asymptomatic forms, in whom the SARS-CoV-2 virus was isolated during contact testing. In the United States, only 1.6-2.5% of children with COVID-19 required hospitalization, and there was no need for intensive care.

The severity of clinical manifestations of coronavirus infection varies from no symptoms (asymptomatic course) or mild respiratory symptoms to severe SARS, which occurs with:

- High fever;
- A pronounced deterioration in well-being, even to the point of impaired consciousness;
- Chills, sweating;

- Headaches and muscle pain;
- Dry cough, shortness of breath, rapid and difficult breathing;
- Rapid heartbeat.

The most common manifestation of SARS is bilateral viral pneumonia complicated by ARDS or pulmonary edema. Respiratory arrest may occur, requiring mechanical ventilation and intensive care.

Adverse outcomes develop with progressive respiratory failure and the addition of a secondary infection, which occurs in the form of sepsis.

Possible complications:

- ARDS;
- Acute heart failure;
- Acute renal failure;
- Septic shock;
- Multiple organ failure (dysfunction of many organs and systems).

Not all children with suspected COVID-19 who had severe forms of the disease had the SARS-CoV-2 virus isolated in the laboratory, which does not allow us to exclude combined infections or the presence of other respiratory diseases in children with suspected cases of the disease based on clinical and epidemiological data.

Mild severity is characterized by an increase in body temperature no higher than 38.5°C, absence of shortness of breath at rest, but it may appear during physical exertion, SpO2 > 95%.

Moderate severity is characterized by an increase in body temperature above 38.5 ◦ C, absence of shortness of breath at rest, but its appearance during physical exertion (screaming/crying), SpO2 ≤ 95%.

Severe COVID-19 is characterized by dyspnea (feeling of shortness of breath, tightness in the chest, shortness of breath, or tachypnea), cyanosis/acrocyanosis, SpO2 ≤ 93%.

An extremely severe degree is recorded with the development of respiratory failure with the need for respiratory support, ARDS, shock, signs of multiple organ failure (encephalopathy, cardiovascular, renal, hepatic failure, DIC syndrome).

The incidence of severe and extremely severe cases of the disease does not exceed 1%. Cases of extremely severe COVID-19 in children are usually registered in the presence of risk factors: severe premorbid diseases (children with lung diseases, congenital heart defects, bronchopulmonary dysplasia, Kawasaki disease, hydronephrosis, leukemia, etc.), immunodeficiency states of various origins (children over 5 years of age are more often affected; pneumonia is registered 1.5 times more often), the influence of coinfection with other respiratory viruses (respiratory syncytial virus, rhinovirus, bocavirus, adenovirus) on the severity of the condition cannot be ruled out), which are characterized by damage to the lower respiratory tract (pneumonia, bronchiolitis).

If an asymptomatic form or mild severity of COVID-19 is detected , outpatient treatment, including at home, is permissible. If a decision is made on the possibility of outpatient treatment, the doctor must explain to the parents or other legal representatives of the child the need to observe the isolation regime. The legal representatives of the child providing care sign an informed consent of the established form. In the case of outpatient treatment, it is possible to use remote consultation of the patient (legal representative) using telemedicine technologies.

Indications for hospitalization of children with COVID-19 or suspected of it:

1. Severe or moderate respiratory disease or community-acquired pneumonia.
2. Fever above 38.5 °C, including according to anamnesis data, or below 36.0 °C, or with a fever duration above 38.0 °C for more than 5 days.
3. Shortness of breath at rest or when anxious.
4. Tachypnea not associated with fever, more than 20% of the age norm: up to 1 year - more than 50, from 1 to 5 years - more than 40, over 5 years - more than 30 per minute.
5. Tachycardia not associated with fever, more than 20% of the age norm: up to 1 year - more than 140, from 1 to 5 years - more than 130, over 5 years - more than 120 per minute.
6. $SpO2 \leq 95$ %.
7. Depression of consciousness (drowsiness) or increased arousal, sleep inversion, refusal to eat or drink.
8. Cramps.
9. Lack of positive dynamics or increase in clinical symptoms against the background of therapy 5 days after the onset of the disease.
10. Presence of severe underlying diseases:
    - Congenital and acquired heart diseases, including those in the anamnesis (heart defects, rhythm disturbances, myocardiopathy or myocarditis);
    - Chronic lung diseases (bronchopulmonary dysplasia, bronchial asthma, cystic fibrosis, bronchiectasis, congenital lung defects, etc.);
    - Primary or secondary immunodeficiency, including HIV infection, autoimmune diseases, immunosuppressive therapy;
    - Oncohematological diseases, chemotherapy;
    - Metabolic diseases (diabetes, obesity, etc.);
    - Liver and kidney diseases.
11. Impossibility of isolation when living with people from risk groups.
12. Lack of conditions for treatment at home or guarantees of compliance with recommendations (hostel, social welfare institutions, temporary accommodation center, socially disadvantaged family, poor social and living conditions).

Indications for transfer to the intensive care unit:

1. Impaired consciousness (14 points or less on the Glasgow Coma Scale for the corresponding age category) or unexplained severe agitation (crying, screaming) against the background of the course of ARI;

2. Increase in respiratory rate by more than 15% of physiological age indicators at rest;
3. Groaning or grunting breathing;
4. Increase in heart rate by more than 15% of physiological age indicators at rest;
5. Cyanosis and dyspnea determined by visual examination, flaring of the nasal wings in children in the first year of life;
6. $SpO2 \leq 93\%$;
7. Respiratory acidosis (pCO2 > 50 mmHg);
8. Decompensated disorders of the acid-base balance of the blood (pH < 7.25);
9. Severe tissue perfusion disorders, arterial hypotension;
10. Lactic acidosis (lactate concentration > 2.5 mmol/l);
11. Arterial hypotension with clinical manifestations of shock;
12. Decreased diuresis to the level of oliguria and below (below 50% of the age norm and less);
13. The appearance of a cough with blood in the sputum, pain or heaviness in the chest;
14. The appearance of signs of hemorrhagic syndrome.

Between 0.5% and 2% of hospitalized children require intensive care.

For antiviral therapy of children with COVID-19 in cases of moderate and mild disease, IFN-α drugs are recommended; in cases of severe disease, intravenous immunoglobulins are used.

Interferon-alpha can reduce the viral load in the early stages of the disease, relieve symptoms and reduce the duration of the disease. Studies in China in children have shown the possibility of a longer excretion of the virus with feces than from the upper respiratory tract (up to 28 versus 14 days). Therefore, it is justified to use IFN-α suppositories, especially with antioxidants, which provide a systemic effect of the drug, can help reduce the period of excretion of the SARS-CoV-2 virus with feces.

Umifenovir is used in patients with COVID-19, but there is no evidence of its effectiveness and safety. The drug can be used in children over 6 years of age.

Antibacterial therapy is indicated if there are signs of a bacterial infection joining the COVID-19 infection .

Recovery in children usually occurs within 1-2 weeks.

7. PREVENTION OF CORONAVIRUS INFECTION

7.1. SPECIFIC PREVENTION OF COVID-19 IN ADULTS

In the Russian Federation, the following vaccines are registered for the specific prevention of COVID-19 in adults:

- combined vector vaccine (Gam-COVID-Vac), date of registration 11.08.2020;
- combined vector vaccine (Gam-COVID-Vac-Lio), date of registration 25.08.2020,
- vaccine based on peptide antigens (EpiVacCorona), registration date 13.10.2020

- coronavirus vaccine inactivated whole-virion concentrated purified ("CoviVac"), date of registration 19.02.2021
- vaccine for the prevention of COVID-19 (Sputnik Light), registration date 06.05.2021
- vaccine based on peptide antigens (EpiVacCorona-N), registration date 08/26/2021.

The combined vector vaccine "Gam-COVID-Vac" is obtained by biotechnology, which does not use the SARS-CoV-2 virus. The drug consists of two components: a recombinant adenoviral vector based on human adenovirus serotype 26, carrying the SARS-CoV-2 S-protein gene (component I) and a recombinant adenoviral vector based on human adenovirus serotype 5, carrying the SARS-CoV-2 S-protein gene (component II). The vaccine "Gam-COVID-Vac" is used to prevent COVID-19 in adults over 18 years of age.

The Gam-COVID-Vac vaccine in the form of a frozen preparation should be stored in a place protected from light, at a temperature not exceeding minus 18 °C [XII]. When carrying out vaccination, it is allowed to store a defrosted single-dose ampoule for no more than 30 minutes. A vial containing 3.0 ml of the vaccine is intended for vaccination of five patients, contains 5 doses of 0.5 ml. It is allowed to store an opened vial of 3.0 ml for no more than 2 hours at room temperature.

The Gam-COVID-Vac vaccine in the form of a liquid preparation and the Gam-COVID-Vac-Lio vaccine in the form of a lyophilisate for the preparation of a solution must be stored in a place protected from light, at a temperature of +2 to +8 °C. Storage of the reconstituted preparation is not allowed!

Based on the known effects of COVID-19 vaccines on obstetric and perinatal outcomes, there is no evidence that these vaccines pose a risk to pregnant women or their fetuses. The CDC registry, which contains the most data on vaccinations during pregnancy, currently includes more than 114,000 women (as of May 17, 2021). Data on 827 patients vaccinated during pregnancy have been published. The incidence of pregnancy complications was no different from the incidence of complications in unvaccinated patients. It was concluded that to date, there is no evidence of a negative impact of vaccination on pregnancy and perinatal outcomes.

Therefore, some major foreign national associations have recommended vaccination of pregnant women who are not vaccinated before pregnancy and who have a high risk of developing severe COVID-19. Pregnant women at risk of severe COVID-19 include those with obesity, chronic lung disease, diabetes, cardiovascular disease, cancer, chronic kidney disease, and liver disease.

During the study of the reproductive toxicity of the domestic vaccine Gam-COVID-Vac on animals, no negative effects on the course of pregnancy, embryofetal development (in females) and prenatal development of the offspring were found. However, the experience of clinical use of the drug Gam-COVID-Vac during pregnancy is insufficient. In this regard, the Gam-COVID-Vac vaccine should be used during pregnancy in cases where the expected benefit to the mother outweighs the potential risk to the fetus. That is, it is advisable to vaccinate in the risk group for

[XII]The requirements for the conditions of transportation and storage of the Gam-Covid-Vac vaccine are approved by the Resolution of the Chief State Sanitary Doctor of the Russian Federation dated December 3, 2020 No. 41 "On approval of sanitary and epidemiological rules SP 3.1.3671-20 "Conditions for transportation and storage of the vaccine for the prevention of a new coronavirus infection (COVID-19) Gam-Covid-Vac"

severe COVID-19 from the 22nd week of pregnancy. There are no clinical data on the use of the Gam-COVID-Vac vaccine in breastfeeding women. Therefore, the risk for newborns/infants who are breastfed cannot be excluded. In this regard, the use of the Gam-COVID-Vac vaccine during breastfeeding is contraindicated.

The Sputnik Light vaccine is a recombinant adenoviral vector based on human adenovirus serotype 26, carrying the SARS-CoV-2 S-protein gene and is used to prevent COVID-19 in adults over 18 years of age.

The vaccine based on peptide antigens (EpiVacCorona and EpiVacCorona-N) is a chemically synthesized peptide antigen of the S protein of the SARS-CoV-2 virus , conjugated to a carrier protein and adsorbed on an aluminum-containing adjuvant (aluminum hydroxide). The vaccine is stored at a temperature of +2 to +8 °C. Do not freeze the vaccine! The vaccine is administered twice with an interval of 21 days at a dose of 0.5 ml intramuscularly into the upper third of the outer surface of the shoulder. The EpiVacCorona vaccine is used to prevent COVID-19 in adults over 18 years of age, the EpiVacCorona-N vaccine is used in adults aged 18-60 years.

The inactivated vaccine ("CoviVac") is a purified concentrated suspension of the SARS-CoV-2 coronavirus strain "AYDAR-1", obtained by reproduction in a continuous Vero cell culture , inactivated with beta-propiolactone. The vaccine is stored at a temperature of +2 to +8 °C. Do not freeze the vaccine! The vaccine is administered twice with an interval of 14 days at a dose of 0.5 ml intramuscularly into the upper third of the outer surface of the shoulder. The "CoviVac" vaccine is used to prevent COVID-19 in adults aged 18-60 years.

Vaccination of the adult population against COVID-19 is carried out in accordance with the temporary methodological recommendations of the Ministry of Health of the Russian Federation “Procedure for vaccinating the adult population against COVID-19” (Letter of the Ministry of Health of Russia dated 08.24.2021 No. 30-4/I/2-13501).

In the Calendar of Preventive Vaccinations for Epidemiological Indications[XIII] categories of citizens have been determined based on the priority of receiving the COVID-19 vaccine. It is also recommended to vaccinate people over 60 years of age who have no contraindications to vaccination as a priority.

Since 18.01.2021, mass vaccination of the population against COVID-19 has been carried out in the Russian Federation.

Numerous studies have shown that more than 80% of virus-neutralizing antibodies are directed to the receptor-binding domain (RBD) of the SARS-CoV-2 S protein . When assessing the intensity of post-vaccination protective immunity using enzyme-linked immunosorbent assay, it is recommended to determine antibodies to the receptor-binding domain (anti-RBD antibodies).

Vaccines are registered under a special registration procedure, which requires notifying the Federal Service for Surveillance in Healthcare of each fact of use of a medicinal product by entering information into the relevant section of the Unified State Health Information System

[XIII]Order of the Ministry of Health of the Russian Federation dated December 9, 2020 No. 1307n "On Amendments to the Calendar of Preventive Vaccinations for Epidemiological Indications, Approved by Order of the Ministry of Health of the Russian Federation dated March 21, 2014 No. 125n"

(EGISZ). The fact of use of the vaccine at stages I and II is confirmed by entering information in the form of the register of those vaccinated against COVID-19 into the Unified State Health Information System (EGISZ).

When working with adverse reactions after immunization with vaccines, the provisions of the "Methodological recommendations for the detection, investigation and prevention of adverse reactions after immunization" (approved by the Ministry of Health of Russia on 12.04.2019) should be taken into account. In the constituent entities of the Russian Federation, it is preferable to create and approve regional immunological commissions (hereinafter referred to as the Commission), which can be promptly involved in conducting a commission investigation of adverse reactions after immunization. The investigation report for each case of a serious adverse reaction after immunization is sent to the Central Office of Roszdravnadzor in electronic form as an appendix to the notification of an adverse reaction to a medicinal product of the information resource "Pharmacovigilance 2.0" of the Automated Information System of Roszdravnadzor.

7.2. NON-SPECIFIC PREVENTION OF COVID-19

Non-specific prevention is measures aimed at preventing the spread of infection:

- early diagnosis and active identification of infected people, including those with asymptomatic forms;
- compliance with the self-isolation regime;
- maintaining a distance of 1.5 to 2 meters;
- the use of social distancing measures (temporary closure of catering establishments, retail trade (except for trade in essential goods), transition to remote work, transfer of educational institutions to distance learning;
- compliance with personal hygiene rules (wash hands with soap, use disposable tissues when sneezing and coughing, touch your face only with clean tissues or washed hands);
- use of personal respiratory protective equipment depending on the degree of risk of infection (face shield, disposable medical mask, respirator, isolating half mask, full face mask);
- carrying out disinfection measures;
- irrigation of the nasal mucosa with an isotonic solution of sodium chloride;
- the use of topical medications that have barrier functions;
- timely referral of the patient to medical organizations in the event of the appearance of symptoms of ARI;
- other activities in accordance with regulatory and methodological documents on the fight against the new coronavirus infection.

Anti-epidemic measures for a new coronavirus infection are carried out in relation to the source of infection (a sick person and an asymptomatic carrier), the mechanism of transmission of the infectious agent, as well as the susceptible contingent (protection of persons who are and/or have been in contact with a sick person).

Measures against the source of infection:

- diagnostic;
- medicinal;
- isolation, including hospitalization for clinical reasons and epidemiological indications, using special transport and observing routing to medical organizations.

Measures aimed at the mechanism of transmission of the infectious agent:

- focal disinfection: current and final;
- compliance with personal hygiene rules (hand washing, use of antiseptics, medical masks, gloves);
- use of PPE for healthcare workers;
- disinfection and disposal of class B medical waste.

Activities aimed at susceptible groups:

- emergency prevention;
- restrictive measures, including clinical observation and quarantine;
- testing for SARS-CoV-2 when clinical signs of infection appear.

7.3. MEDICAL PREVENTION OF COVID-19 IN ADULTS

For drug prophylaxis of COVID-19 , it is possible to prescribe intranasal forms of IFN-α (in pregnant women, only intranasal administration of recombinant IFN-α2b is possible) and/or umifenovir (Appendix 9) .

7.4. PREVENTIVE MEASURES SPREAD OF COVID-19 IN MEDICAL ORGANIZATIONS

Upon admission to the emergency department of a medical organization providing medical care in inpatient settings of a patient with clinical manifestations of acute respiratory viral infection with symptoms characteristic of COVID-19 and epidemiological history data, the medical worker carries out a set of primary anti-epidemic measures using PPE [XIV].

A medical worker, without leaving the room in which the patient was identified, using the available means of communication, notifies the head of the medical organization about the identified patient and his condition in order to resolve the issue of his isolation at the place of his identification (the admission department box) until his transfer to the Meltzer box of the medical organization in which the patient was identified, or

[XIV] MR 3.1.0252-21. 3.1. Prevention of infectious diseases. Amendments No. 1 to MR 3.1.0229-21 "Recommendations for the organization of anti-epidemic measures in medical organizations providing medical care to patients with a new coronavirus infection (COVID-19) (suspected disease) in inpatient settings." Methodological recommendations (approved by the Chief State Sanitary Doctor of the Russian Federation on July 9, 2021)

hospitalization in a specialized infectious disease hospital if it is impossible to organize the patient's isolation in the medical organization where he/she was identified. In case of patient isolation at the place of identification, transfer to a specialized infectious disease hospital is carried out after confirmation of the COVID-19 diagnosis.

Healthcare workers who have identified a patient with clinical manifestations of acute respiratory viral infection with symptoms characteristic of COVID-19 must monitor the patient until he or she arrives and is transferred to an ambulance team.

After the medical evacuation of the patient, the health worker who identified the patient removes the PPE, places it in a tank with a disinfectant solution, treats shoes and hands with a disinfectant solution, and changes completely into a spare set of clothes. Open parts of the body are treated with a skin antiseptic. The mouth and throat are rinsed with 70% ethyl alcohol, and a 2% solution of boric acid is instilled into the nose and eyes.

The laboratory is a zone of increased biological danger. When conducting research on COVID-19, this danger increases many times over. The work of MAAT is carried out in accordance with the Methodological Guidelines MU 1.3.2569-09 "Organization of the work of laboratories using MAAT when working with material containing microorganisms of pathogenicity groups I-IV ".

Not only NAAT, but also any other laboratory tests on material from COVID-19 patients in other departments of the laboratory pose a high biological hazard. It is not recommended to conduct a sputum test on patients with community-acquired pneumonia in the general clinical department until a negative result for COVID-19 is obtained. In case of a positive answer, all work should be carried out in a biological safety cabinet of at least class II protection and as part of a bacteriological study. General sputum analysis can be performed according to strict indications with potential clinical information content and with full equipment of the laboratory employee, as for working with NAAT.

In order to disinfect surfaces in the admission department, disinfection is carried out, including using the aerosol method of disinfecting premises. For rapid and effective disinfection of the air and surfaces in the absence of people, open-type ultraviolet bactericidal irradiators with high doses of UV radiation (at least 25 mJ/cm2) and a total bactericidal radiation flux of at least 200 W are used ; for air disinfection in the presence of people, closed-type ultraviolet bactericidal irradiators (recirculators) with high doses of UV radiation (at least 25 mJ/cm2) and with sufficient productivity (ensuring recirculation of the entire air volume in the room at least 4 times per hour) are used. The number of required
The irradiators are calculated in accordance with the instructions for their use for the cubic capacity of the room in which they will be installed.

If the diagnosis of COVID-19 is confirmed in a hospital, it is necessary to identify persons who had contact with the patient, including:

- Those who were in this institution;
- Transferred or referred (for consultation, inpatient treatment) to other medical

organizations, and discharged;

- Medical and other workers
  (cloakroom, reception, diagnostic and examination rooms);
- Visitors to a medical organization, as well as visitors who have left the medical organization by the time the patient is identified;
- Persons at the patient's place of residence, work, study.

Persons in contact with the patient are placed under medical observation.

In conditions of high probability of admission of a patient with a new coronavirus infection (the presence of local transmission of infection in the territory), the following measures must be implemented:

- Prohibition of visits by relatives and other persons to patients in inpatient medical institutions;
- Prohibition of visits to inpatient medical institutions by persons who are not employees of the organization;
- Stopping and postponing planned hospitalization;
- Conducting medical examination and temperature measurements of all inpatients twice a day, with the results recorded in the observation sheet;
- Training and instruction of medical personnel on preventing the spread of COVID-19, conducting anti-epidemic measures, using PPE and personal preventive measures;
- Developing a procedure for action upon identification of a patient with suspected infection caused by the new coronavirus.

In inpatient medical institutions, it is necessary to organize isolation wards where a patient with a suspected infection caused by the new virus can be placed.

Hospitalization of patients (persons with suspected disease) is carried out in boxes, boxed wards or, in their absence, in wards with an airlock and a toilet, observing the principle of one-time (cyclical) filling

wards and taking into account the severity of the patient's condition. Persons with suspected cases are recommended to be placed in single wards. Patients with a confirmed diagnosis can be placed in wards for 2-4 people, subject to the hygienic requirements for the area of wards per 1 bed in infectious disease hospitals (at least 8 sq. m) and placement of beds at a distance of at least 1.5 - 2 meters from each other. Patients in the presence of staff or other patients must wear medical masks and observe hand hygiene. Patients are not allowed to leave the wards (boxes). The use of mobile communications is permitted (in intensive care units, taking into account the clinical condition) with their disinfection with alcohol-containing products.

To perform medical manipulations, care, cleaning, disinfection, it is necessary to assign personnel and equipment to work only with this patient. It is necessary to review the instructions for medical personnel in order to minimize visits to the patient's isolation area. For example, combining a medical examination and minor invasive interventions). All those entering the isolation ward must be recorded in the log.

Patient movement within a medical facility is strictly prohibited. However, if there are no necessary material and technical resources for providing medical care (e.g. portable ultrasound machines, mobile X-ray equipment stands), the patient is moved along strictly specified routes in a transport isolation box for infectious patients. In the absence of a transport isolation box, before leaving the ward, the patient must change into clean clothes, put a respirator on the mouth and nose, and the patient's hands must be treated with an alcohol antiseptic. When moving, the patient must not touch any surfaces (walls, door handles, etc.).

Patients with suspected infection and those with confirmed infection should be separated (stay in different rooms). If new patients with suspected infection are identified, they can be placed in a department (room) together with other patients with suspected infection.

If during hospitalization a patient with suspected or confirmed infection requires intensive care or oxygen support, if possible an intensive care post should be organized in the ward where the patient is isolated. If this is not possible, an isolation ward and a separate post in the intensive care unit should be organized. The following requirements should be met:

- Place a respirator or medical mask on the patient, if possible;
- When providing artificial ventilation, use closed breathing circuits;
- All medical equipment must be assigned to the patient;
- Access to the area where the patient is located must be restricted;
- The department must organize the collection and disposal of class B waste.

When performing aerosol-generating medical procedures (tracheal intubation, non-invasive ventilation, tracheotomy, cardiopulmonary resuscitation, artificial ventilation using manual devices before intubation and bronchoscopy), personnel must:

- Use a respirator (such as NIOSH-certified N95, FFP3, or similar). When using a disposable respirator, a leak test is mandatory. It should be taken into account that in the case of PPE users with a moustache and/or beard, ensuring a leak-proof seal may be difficult;
- Use eye protection

(eg safety glasses or face shield);

- Wear protective coveralls or, if unavailable, disposable surgical gowns and gloves. Gloves should be pulled over the sleeves. If gowns are not waterproof, waterproof aprons should be used when performing procedures that involve handling large volumes of liquids that may penetrate the gown;
- Minimize the number of contacts with the patient. Group manipulations for their sequential implementation: transition from "clean" to "dirty".

All personal protective equipment must be removed and discarded in a Class B waste container without leaving the intensive care unit.

Medical waste, including biological secretions of patients (sputum, urine, feces, etc.), are classified as extremely epidemiologically hazardous waste of class B and are subject to mandatory decontamination (disinfection)/neutralization by physical methods (thermal, microwave, radiation and others), for which purpose a medical organization must provide for the presence of a specialized installation operating using such methods. Removal of non-disinfected class B waste outside the territory of a medical organization is not permitted. After hardware methods of disinfection using physical methods and changing the appearance of waste, class B waste can accumulate, temporarily store, transport, destroy and be buried together with class A waste. The use of chemical disinfection methods is possible only for the disinfection of food waste and patient excreta, as well as when organizing anti-epidemic measures in outbreaks. Disposal of medical waste is carried out in accordance with the regulations adopted in the institution, which do not contradict sanitary and epidemiological rules and regulations.

To prevent the occurrence of healthcare-associated infections (HAI) in patients with COVID-19 , it is necessary to implement a full range of measures to ensure the epidemiological safety of medical care, including hand treatment (in two pairs of gloves) with alcohol antiseptics, the use of a third pair of sterile / non-sterile gloves in accordance with standard indications, performing all invasive manipulations in compliance with standard operating procedures, monitoring invasive procedures and timely examination of patients if HAI is suspected, adequate antimicrobial therapy taking into account the antibiotic resistance of the isolated pathogens, ongoing and final disinfection of surfaces and equipment, including ventilators, oxygen concentrators, Bobrov jars, etc., as well as air disinfection. It is necessary to enhance microbiological monitoring of circulating strains of relevant infectious agents, including *Klebsiella pneumoniae, Acinetobacter baumannii, Pseudomonas aeruginosa, Staphylococcus spp.* (MRSA, MRSE) and others, by examining patients and objects of the hospital environment with the determination of the resistance of microorganisms to antimicrobial drugs. It is necessary to strengthen control over ensuring the epidemiological safety of medical care and the prevention of

HAI using checklists and the use of various forms of observation, including video surveillance [XV].

7.5. RATIONAL USE OF PERSONAL PROTECTIVE EQUIPMENT IN MEDICAL ORGANIZATIONS

In order to limit the unjustified consumption of PPE, it is recommended to determine the need for them in medical organizations, as well as to ensure their correct use. To rationalize the need for PPE while simultaneously ensuring the safety of medical workers, it is recommended:

- Using technical and administrative measures, limit the number of people working in high-risk areas and requiring the use of PPE;
- Determine the required number of health care workers in contact with patients;
- Regulate the required number of entrances to the chamber;
- Provide remote consultations for patients and persons suspected of having COVID-19.
- Introduce into practice the extended use of respirators (wearing the same respirator when working with several patients without removing the respirator).

Respirators, or filtering half masks, are personal respiratory protective equipment (PPE) designed to reduce the risk of infection of healthcare workers working in high-risk conditions of airborne infections. There is reason to believe that the rapid spread of coronavirus infection is facilitated by the fact that the virus is transmitted not only through direct contact with the source of infection and by airborne droplets (through large droplets), as is typical for most ARVI, but also to a significant extent through infectious aerosols (infected air). Therefore, the use of respirators (with a protection rating of at least FFP2) in high-risk infection areas is mandatory for medical personnel involved in providing medical care to COVID-19 patients.

The principle of operation of the respirator is based on highly efficient filtration of inhaled air, due to which the risk of infectious aerosol (a persistent suspension in the air of tiny particles containing viable pathogenic microorganisms) penetrating into the respiratory tract, including the terminal bronchioles and alveoli, is sharply reduced.

To ensure maximum protection when using a respirator, a number of conditions must be met:

- the respirator models used must be certified for compliance with the requirements of at least one of the national or international standards: TR CU 019/2011 “On the safety of personal protective equipment”, or GOST 12.4.294-2015 or EN 149:2001+A1:2009 “Respiratory protective devices - Filtering half masks to protect against particles”;
- the respirators used must have a protection class of at least FFP2;
- The respirator must be used correctly (proper donning, safe removal, care, disposal and

[XV]Methodological recommendations MR 3.1.0229-21 "Recommendations for the organization of anti-epidemic measures in medical organizations providing medical care to patients with a new coronavirus infection (COVID-19) (suspected disease) in inpatient settings"

disposal as class B medical waste).

Proper donning is the most important condition for its effectiveness in protecting against infection. Proper donning is absolutely

is necessary to ensure the most hermetic fit of the edges of the respirator half mask to the face to eliminate the possibility of leakage of unfiltered infected air into the breathing zone bypassing the highly effective filter, which is the respirator half mask. It is extremely important to read and carefully follow the instructions for the correct donning of the respirator each time in text or graphic form in accordance with the above standards is always located on the respirator packaging or in the insert.

After each donning of the respirator before entering a high-risk infection area, it is necessary to check it for leaks: do 2–3 forced inhalations and exhalations, making sure that there is no air suction or air leakage along the edges of the respirator, and that when inhaling, the respirator is pressed tightly to the face without air leakage along the edges. If air leakage under the half mask is detected, you need to check the correctness of putting on the respirator, put it on again.

The duration of use of the respirator during the working day is limited only by hygienic considerations (the need to eat, the appearance of excess moisture under the half mask in hot weather, etc.), since the filtration efficiency only increases over time, provided that the respirator is not damaged and ensures a good fit to the face.

Safe removal of the respirator is necessary to eliminate the risk of infection from the outer surface of the respirator as a result of its contact with the skin, if it is contaminated with infected biological fluids. The respirator is removed with gloves by the elastic bands (first removing the lower elastic band, then the upper one), without touching the outer and inner surfaces of the respirator half mask.

Disinfection and/or rendering harmless of used respirators is carried out in accordance with the requirements for class B medical waste.

In the context of an infectious disease epidemic, it is important to apply organizational measures that not only reduce the risk of nosocomial spread of infection, but also significantly reduce the need for respirators:

- Training personnel in the principles of proper use of respirators, including avoiding wearing them on the neck or forehead during work breaks, and proper careful storage not only increases the efficiency of their use, but also extends their service life;
- Conducting a risk assessment based on patient flow analysis, visitors, laboratory samples and personnel;
- Maximum separation of flows to identify low-risk areas (where the use of RPE is not required) and high risk (where the use of RPE is required). High risk areas should be designated

special warning signs prohibiting access by unauthorized persons without protective equipment;

- Allocation of personnel rest areas and office work areas in maximally isolated areas, where the flow of infected air from high-risk areas is excluded. The use of RPE in these areas is not required;
- Based on risk assessment, selection of narrower groups of personnel who work in the highest risk conditions, where the use of RPE is required. Other personnel, in order to work in low or medium risk conditions, can effectively use the listed organizational measures to reduce it and measures to control the living environment (ventilation, ultraviolet emitters);
- Mandatory 24-hour use of medical masks by patients who pose a risk of spreading infection reduces the risk to others by half;
- The use of the maximum possible natural ventilation modes (constant maximum possible ventilation) allows for a sharp reduction in the concentration of infectious aerosol in the air of premises and, accordingly, a sharp reduction in the risk of spreading infections through the air. An effective method of disinfecting the air environment of premises is the use of ultraviolet bactericidal units with a dose of ultraviolet bactericidal radiation (UVBR) of at least 25 mJ/cm ^{2}, built into central supply and exhaust ventilation systems. Such supply and exhaust ventilation in the presence of people must operate continuously throughout the entire working time.
- In areas with a high risk of COVID-19 spread, the use of room air conditioners (split systems) should be excluded, since they actually increase the risk of infection, as they contribute to maintaining high concentrations of infectious aerosols when natural ventilation is blocked. The use of closed-type irradiators (recirculators) with a UVBI source inside is an effective measure to reduce the risk of spreading airborne infections, including COVID-19, only if sufficient UV irradiation doses are provided (at least 25 mJ/cm2 $^{)}$ and the required air exchange rate (ensuring recirculation of the entire air volume in the room at least 4 times per 1 hour). Closed irradiators in the presence of people must operate continuously throughout the entire working time.

COVID-19 epidemic rise, their availability may be temporarily limited due to a sharp increase in demand for them.

The proposed recommendations for longer and re-use of PPE are temporary in nature during periods of possible PPE shortages.

If there is a shortage of respirators in a medical organization, it is possible to introduce a regime of limited reuse (using the same respirator with a surgical mask over it during multiple contacts with patients, while after each contact it is necessary to change the upper surgical mask).

Reuse of a respirator by the same healthcare worker when providing care to patients with COVID-19 is possible if the following conditions are met:

- the respirator is not damaged;
- the respirator ensures a tight fit to the face, eliminating air leakage under the half mask;
- the respirator does not create excessive breathing resistance due to high humidity;

- The respirator has no visible traces of contamination with biological fluids.

The duration and frequency of reuse of the respirator are determined by the fulfillment of all the above conditions. If at least one of the above conditions is not fulfilled, safe reuse of such a respirator is impossible, and it is subject to decontamination and/or rendering harmless with subsequent burial or destruction in accordance with the requirements for class B medical waste.

If the respirator is to be reused, it is marked with the user's initials, disinfected with ultraviolet germicidal irradiation, allowed to dry completely if the respirator is wet, and stored in a paper bag or napkin until use.

It is permissible to use UVBI for disinfection of the outer surface of used respirators and their reuse. UVBI, without penetrating deeply into the filter material, effectively disinfects its irradiated surface, sharply reducing the risk of contact infection. To disinfect the surface of the respirator, it is left carefully straightened with the outer surface upward in the direction of the open-type UVBI irradiator with a total bactericidal radiation flux of at least 100 W at a distance of no more than 2 meters from it for at least 10 minutes. The total dose of UVBI is sufficient for reliable disinfection of the unshaded surface of the filtering half mask for its safe reuse, subject to the above standard measures to prevent contact infection.

Used respirators must not be washed, mechanically cleaned, treated with disinfectants, disinfected with high temperatures, steam, etc. Between periods of reuse, the respirator must be stored unfolded in a dry, clean place (in a napkin or paper bag with the user's initials). Transferring the respirator for use by another person is not allowed.

Using a medical (surgical) mask over a properly fitted respirator can dramatically reduce the likelihood of contamination of the outer surface of the respirator with biological fluids. At the same time, after each removal of the respirator, the mask should be discarded in a class B waste container, and the respirator can be reused.

In exceptional cases, when the respirator resource is limited, it is permissible to use disposable surgical masks when providing medical care to patients who do not require aerosol-generating procedures (oxygenation, intubation, bronchial tree sanitation, artificial ventilation, etc.). In this case, a surgical mask must also be put on the patient.

8. PROCEDURE FOR CONDUCTING PATHOLOGICAL ANATOMICAL AUTOPSIES

In case of death in hospital of a patient diagnosed with COVID-19 during life or classified as a "suspected and probable case of COVID-19" , a pathological autopsy in accordance with Federal Law No. 323-FZ of 21.11.2011 "On the Fundamentals of Health Protection of Citizens in the Russian Federation" and Order of the Ministry of Health of Russia No. 354n of 06.06.2013 "On the Procedure for Conducting Pathological Autopsies" is carried out in specially repurposed for such autopsies, including interhospital pathological departments, in compliance with biosafety rules. Cancellation of an autopsy is not allowed. Complexity category 5 (Appendix 1 of Order No.

354n).

The administration of pathological anatomy bureaus, hospitals that have pathological anatomy departments, and forensic medical examination bureaus ensure compliance with the requirements of SP 1.3.3118-13 “Safety of work with microorganisms of pathogenicity (hazard) groups I-II ” and other regulatory and methodological documents in a separate section.

The pathology department must have a complete set of instructions and the necessary means for their implementation:

- Methodological folder with operational plan of anti-epidemic measures
- measures in case of detection of a patient with COVID-19;
- Alert scheme;
- A reminder on the technique of opening and collecting material for bacteriological examination;
- Functional responsibilities for all employees of the department;
- Protective clothing (type 1 anti-plague suit, PPE type "Quartz" and similar, type II anti-plague suit is allowed with the additional wearing of double surgical gloves and cut-resistant synthetic gloves between them, protective glasses, an oilcloth or polyethylene (laminated) apron, armlets made of similar material; it is necessary to use FFP3 class respirators);
- Laying for collecting material;
- Sterile dissection set;
- A supply of disinfectants and containers for their preparation.

Medical waste generated as a result of the pathological examination of such corpses is subject to disinfection and/or disposal in accordance with the requirements for class B medical waste (SanPiN 2.1.7.2790-10 “Sanitary and Epidemiological Requirements for Handling Medical Waste”).

The autopsy is performed or supervised by the head of the department or the most experienced pathologist. Pathologists, medical technicians (laboratory assistants) and orderlies are allowed to perform pathological anatomical autopsies.
pathological anatomy department, who have undergone instructions, special training (in-person or remote). The autopsy should be performed as soon as possible. The time of the autopsy and the number of personnel involved should be reduced to a minimum. The autopsy is performed without the use of water with the drain turned off, the so-called "dry autopsy". During the autopsy, especially of the skull, it is necessary to exclude the formation of aerosols. Autopsy material (pieces of the lung, trachea, bronchi) is sent as soon as possible to the Federal Budgetary Institution of Health "Center for Hygiene and Epidemiology" in the constituent entity of the Russian Federation for the presence of COVID-19, as well as other viral and bacterial pathogens of ARI. The volume

and type of biological material is agreed with the territorial body of Rospotrebnadzor.

For histological examination, samples of each organ are taken. Fixation is performed in 10% neutral buffered formalin solution; after fixation in formalin solution for at least one day, the material is biologically safe. Fixation of pieces in duration should correspond to the size of the piece and can be increased to 48-72 hours if necessary. After fixation and histological processing, tissue pieces do not pose an epidemiological hazard, and further sample preparation is carried out in the usual way. Microscopic examination necessarily includes: trachea (proximal and distal sections); central part of the lung with segmental bronchi, right and left first-order bronchi; representative areas of the lung parenchyma from the right and left lung. Histological examination of all other vital internal organs is mandatory, depending on macroscopically detected changes: myocardium, liver, kidneys, spleen, pancreas, brain, and others. All diagnostically significant morphological changes should, if possible, be recorded using macro and microphotography (or video).

9. ROUTING OF PATIENTS AND FEATURES OF EVACUATION MEASURES FOR PATIENTS OR PERSONS SUSPECTED OF HAVING COVID-19

9.1 . ROUTING OF PATIENTS AND PERSONS SUSPECTED OF COVID-19

The routing procedure regulates the provision of medical care to patients with COVID-19 in medical organizations.

Medical care for patients with COVID-19 is provided in accordance with the order of the Ministry of Health of Russia dated March 19, 2020 No. 198n “On the temporary procedure organization of the work of medical organizations in order to implement measures to prevent and reduce the risks of the spread of the new coronavirus infection COVID-19" in the form of emergency, including emergency specialized, primary health care and specialized medical care in medical organizations and their structural divisions, as well as in outpatient settings (at home).

Depending on the severity of the condition, when a diagnosis of coronavirus infection is confirmed, treatment is carried out:

- Medical workers of outpatient medical organizations at home;
- In the department for the treatment of infectious patients of a medical organization;
- In the intensive care unit of a medical organization (if indicated).

Emergency medical care, including specialized emergency medical care, for patients with infectious diseases is provided by paramedic emergency medical care teams, medical emergency medical care teams, specialized emergency medical care teams, and emergency medical care teams of territorial disaster medicine centers.

The activities of these teams are aimed at carrying out measures to eliminate life-threatening conditions with subsequent medical evacuation to a medical organization providing inpatient

medical care to patients with infectious diseases. Medical care to patients with infectious diseases with life-threatening acute conditions, including infectious-toxic, hypovolemic shock, acute renal and hepatic failure, acute cardiovascular and respiratory failure, outside a medical organization is provided by teams (including resuscitation) of emergency medical care.

9.2 ORGANIZATION OF MEDICAL CARE

Anti-epidemic measures in medical organizations are carried out in accordance with the Plan of sanitary and anti-epidemic measures to prevent the importation and spread of a new coronavirus infection caused by SARS-CoV-2, approved by the authorized executive body of the constituent entity of the Russian Federation, as well as the current requirements of sanitary legislation.

a) The procedure for organizing emergency, including specialized emergency, medical care.

Heads of medical organizations providing emergency medical care must ensure:

- Availability of a stock of necessary consumables for collecting samples for laboratory testing for the presence of COVID-19, disinfectants and PPE;
- Informing healthcare workers on issues of prevention, diagnosis and treatment of COVID-19;
- Implementation of the Instructions for compliance with infection safety measures for specialized mobile ambulance teams in accordance with Appendix No. 10 to these guidelines;
- Transfer of biological material (nasopharyngeal and oropharyngeal swabs) from patients (if it is necessary to collect biological material at the emergency medical care stage,

  COVID-19 is suspected in the laboratory of medical organizations that have an epidemiological conclusion to work with pathogenicity groups III and IV ;
- Indication by healthcare workers in the referral form for laboratory testing of the diagnosis of "pneumonia" when referring biological material from patients with community-acquired pneumonia for the diagnosis of COVID-19;
- Systematic work to inform the population about the risks of COVID-19, individual preventive measures, the need to promptly seek medical help when the first symptoms of acute respiratory viral infections appear.

б) The procedure for organizing medical care in inpatient settings.

Heads of medical organizations providing medical care in inpatient settings must ensure:

- Availability of a stock of necessary consumables for the collection of biological material for laboratory testing, disinfectants and PPE (glasses, disposable gloves, a respirator of the appropriate protection class, a type 1 anti-plague suit or a disposable gown, shoe covers),

provision of medical products, including pulse oximeters, artificial lung ventilation devices;

- Informing healthcare workers on issues of prevention, diagnosis and treatment of COVID-19, as well as collecting epidemiological history;
- Hospitalization of patients with atypical course of acute respiratory viral infections, community-acquired pneumonia;
- Conducting anti-epidemic measures when suspected COVID-19 is detected in accordance with the requirements of Rospotrebnadzor;
- Reception of patients with signs of acute respiratory viral infections, community-acquired pneumonia through reception and examination boxes and (or) filter boxes and further routing of patients to a medical organization;
- Compliance with temperature conditions, ventilation conditions, ongoing disinfection in a medical organization (ICUs require special attention), use of PPE by employees of the medical organization;
- Carrying out disinfection of air and surfaces in rooms using ultraviolet bactericidal irradiators with a UV dose of at least 25 mJ/cm2 [and] /or other devices for disinfecting air and (or) surfaces;
- Control of the concentration of disinfectants in working solutions;
- Increasing the frequency of disinfection treatments of medical facilities;
- Transfer of biological material from patients (nasopharyngeal and oropharyngeal swabs) in case of suspected COVID-19 to laboratories of medical organizations that have an epidemiological conclusion to work with pathogenicity groups III and IV , with the execution of an Acceptance and Transfer Certificate;
- Indication by healthcare workers in the referral form for laboratory testing of the diagnosis of "pneumonia" when referring biological material from patients with community-acquired pneumonia for the diagnosis of COVID-19;
- Informing the population about the risks of the spread of COVID-19, individual preventive measures, paying particular attention to the need to promptly seek medical help when the first symptoms of acute respiratory viral infections appear;
- Consideration of the possibility of postponing the provision of planned medical care.

в) The procedure for organizing medical care in outpatient and day hospital settings.

Heads of medical organizations providing medical care in outpatient and day hospital settings must ensure:

- Availability of a stock of consumables for sampling for laboratory tests, disinfectants and medical products, including PPE, medical products, including pulse oximeters;
- Informing healthcare workers on issues of prevention, diagnosis and treatment of COVID-19, as well as collecting epidemiological history;
- Hospitalization of patients with atypical course of acute respiratory viral infections and community-acquired pneumonia;

- Conducting anti-epidemic measures when suspected COVID-19 is detected;
- Reception of patients with signs of acute respiratory viral infections through reception and examination boxes and/or filter boxes, as well as schemes for further routing of patients to medical organizations providing medical care in inpatient settings;
- Compliance with temperature conditions, ventilation conditions, ongoing disinfection in a medical organization, use of PPE by employees of a medical organization;
- Carrying out disinfection of air and surfaces in rooms using ultraviolet bactericidal irradiators with a UV dose of at least 25 mJ/cm2 and (or) other devices for disinfecting air and (or) surfaces;
- Control of the concentration of disinfectants in working solutions;
- Increasing the frequency of disinfection treatments of medical facilities;
- Transfer of biological material from patients (nasopharyngeal and oropharyngeal swabs) in case of suspected COVID-19 to laboratories of medical organizations that have an epidemiological conclusion to work with pathogenicity groups III and IV , with the execution of an Acceptance and Transfer Certificate;
- Indication by healthcare workers in the referral form for laboratory testing of the diagnosis of "pneumonia" when referring biological material from patients with community-acquired pneumonia for the diagnosis of COVID-19;
- Systematic work to inform the population about the risks of COVID-19, individual preventive measures, paying particular attention to the need to promptly seek medical help when the first symptoms of ARI appear;
- Indication of medical care for patients with acute respiratory viral infections in outpatient settings, primarily at home;
- Updating information about persons over 60 years of age, as well as persons suffering from chronic bronchopulmonary, cardiovascular diseases vascular and endocrine systems, persons with motor neuron disease, pregnant women living in the service area medical organization;
- Possibility of remote prescription of medications and their home delivery;
- Medical observation (daily thermometry, questioning of the citizen by a medical worker, including by telephone, for the presence of symptoms of acute respiratory viral infections) of citizens who have returned from countries where cases of COVID-19 have been registered, for a period of at least 14 calendar days from the date of their return, as well as persons living with them. Transfer of consolidated statistical information on the results of medical observation to the territorial office of Rospotrebnadzor;
- Immediate isolation and, if indicated, hospitalization of patients in medical organizations specially created for this contingent that provide medical care in inpatient settings, should be carried out upon suspicion or establishment of the fact of COVID-19 disease;
- The possibility of issuing sick leave certificates without visiting a medical organization for persons who arrived in the Russian Federation from countries where cases of COVID-19

have been registered, as well as for persons living with them.

г) Procedure for organizing laboratory testing for the diagnosis of COVID-19.

Heads of medical organizations that supervise diagnostic laboratories that perform research to diagnose COVID-19 must ensure:

- Availability of a stock of reagents, test systems, consumables for laboratory research, disinfectants and medical products, including PPE to ensure efficient and safe operation of the laboratory;
- Conducting anti-epidemic measures and observing the rules for working with pathological biological agents of hazard group II ;
- Compliance with temperature conditions, ongoing disinfection in the laboratory, use of PPE by laboratory workers;
- Carrying out disinfection of air and surfaces in rooms using ultraviolet bactericidal irradiators with a UV dose of at least 25 mJ/cm2 [and] (or) other devices for disinfecting air and (or) surfaces;
- Control of the concentration of disinfectants in working solutions;
- Increasing the frequency of disinfection treatments of laboratory premises;
- Obtaining biological material from patients (nasopharyngeal and oropharyngeal swabs) in case of suspected COVID-19 with the execution of an Acceptance and Transfer Certificate;
- Indication by healthcare workers in the referral form for laboratory testing of the diagnosis of "pneumonia" when referring biological material from patients with community-acquired pneumonia for the diagnosis of COVID-19;
- Medical supervision (daily temperature monitoring) and laboratory testing of laboratory staff as required.

9.3 FEATURES OF EVACUATION MEASURES AND GENERAL PRINCIPLES OF HOSPITALIZATION OF PATIENTS OR PERSONS SUSPECTED OF HAVING COVID-19

The patient is hospitalized in medical organizations that have Meltzer boxes in their structure, or in medical organizations that are being re-profiled as specialized institutions in the administrative territory where the patient was identified.

Requirements for work in infectious disease hospitals, isolation wards and observation centres are set out in current regulatory and methodological documents [XVI].

[XVI] SP 1.3.3118-13 “Safety of work with microorganisms of pathogenicity (hazard) groups I-II ”.
SP 3.1.3597-20 "Prevention of the new coronavirus infection COVID-19".
Order of the Ministry of Health of Russia dated March 19, 2020 No. 198n "On the temporary procedure for organizing work
medical organizations in order to implement measures to prevent and reduce the risks of the spread of the new coronavirus infection COVID-19."
MR 3.1.0229-21. Recommendations for organizing anti-epidemic measures in medical organizations providing medical care to patients with a new coronavirus infection (COVID-19) (suspected disease) in inpatient settings.

Provision of medical care to patients with infectious diseases during the preparation and implementation of medical evacuation is carried out in accordance with current procedures, clinical recommendations and standards.

In the presence of life-threatening syndrome complexes, resuscitation measures and intensive therapy are carried out according to schemes approved in the established manner.

Transportation of patients with an infectious disease is carried out without a transport isolation box (TIB) or in one.

a) Transporting a patient with an infectious disease without a transport isolation box

Activities of epidemiological teams and/or emergency medical teams before transportation [XVII].

Members of the epidemiological team and/or medical evacuation team, upon arrival at the location where the patient was identified, before entering the room where the patient is located, under the supervision of the senior medical worker of the team, put on protective suits in the established manner [1].

Team doctor:

- Clarifies the patient's epidemiological history data, the circle of people who communicated with him (indicating the date, degree and duration of contact);
- Determines the contingents of persons subject to isolation, medical observation, and emergency prevention;
- Ensures control over the evacuation of the patient and persons in contact with him;
- Reports immediately according to the approved scheme (senior doctor of the shift) updated information about the patient, those who had contact with the patient, and the primary measures taken to localize the outbreak.

The team performing the medical evacuation of an infectious patient must consist of medical workers trained in the requirements of compliance with the anti-epidemic regime and who have received additional training on disinfection issues.

The patient is transported wearing a mask with all precautions. The driver of the vehicle in which the medical evacuation is carried out, if there is an isolated cabin, must be dressed in overalls and a mask, if there is none, in protective clothing in accordance with the established requirements.

If there are several patients with signs of an infectious disease, the senior medical worker of the team determines the number and order of medical evacuation of patients, specifies the patient evacuation route and the medical organization. If there are several medical organizations that meet the minimum requirements for structural units of a medical organization for the treatment of COVID-19 for patients in serious condition, patients with severe and extremely severe forms of the disease should be sent to medical organizations that have all the technologies of respiratory support, efferent and preemptive anti-inflammatory therapy.

[XVII] measures are also taken when transporting patients with infectious diseases using a transport isolation box

Transportation of two or more infectious patients from different foci in one vehicle is not allowed. Joint transportation of several patients with a confirmed diagnosis of COVID-19 from one foci is allowed in the absence of contraindications due to clinical status. Transportation of persons who have been in contact with patients together with the patient in one vehicle is not allowed.

The ambulance is additionally equipped with a set of disinfectants for 1 day:

- Disinfection agent for secretions;
- Disinfection agent for interior surfaces;
- Hand sanitizer for personnel (1-2 packages);
- Bactericidal irradiator.

The consumption of disinfectants required for 1 shift is calculated depending on what product is available and the possible number of trips.

After the patient is delivered to the infectious diseases hospital, the team carries out a full sanitary treatment on the hospital grounds with disinfection of protective clothing.

The vehicle and patient care items undergo final disinfection on the hospital premises by the hospital itself or by teams from the institution’s disinfection profile (in accordance with the comprehensive plan).

The members of the team that carried out the medical evacuation are placed under observation for a period of 14 days.

б) Transportation of a patient with an infectious disease using a transport isolation box

Patients or persons suspected of having COVID-19 are transported using a transport isolation box (TIB) equipped with filtering and ventilation units, windows for visual monitoring of the patient's condition, and two pairs of built-in gloves for performing basic procedures during transportation.

For medical evacuation of the patient, a medical team is formed that has been instructed in matters of sanitary and anti-epidemic regime. Medical workers receive the patient, place him/her in the TIB and provide subsequent support.

All movements of newborns (out-of-hospital and intra-hospital) are carried out in a transport incubator, regardless of gestational age and birth weight. The incubator must be equipped with sealing ports for manipulation and passage through the walls of the incubator of devices necessary for the child's life support.

Medical workers and the driver must wear protective clothing.

The patient is prepared for transportation to the TIB: at the evacuation site, the senior medical worker of the team assesses the patient's condition at the time of transportation and decides on the issue of carrying out additional medical procedures.

The patient is placed inside the transport module chamber in a horizontal position on his back and secured with belts; the equipment and medicines necessary for transportation and medical care are placed in the TIB; after that, the zipper is closed. The reliability of the filter fastening is

checked, and the filter and ventilation unit is turned on to negative pressure mode.

After placing the patient in the TIB, the medical team staff:

- Wipes hands in rubber gloves and the surface of the oilcloth apron, sprays the outer surface of the transport module

  disinfectant solution with exposure in accordance with the instructions for use;
- Treats protective suits by spraying them with a disinfectant solution in accordance with the instructions for use, then removes the protective suits and places them in hazardous waste bags;
- Sprays the outer surface of the bags with used protective suits with a disinfectant and takes them to the vehicle. In the infectious diseases hospital box, the patient from the TIB is handed over to the hospital's medical workers.

After the patient is delivered to the hospital, the medical transport and the TIB, as well as the items in it used during transportation, are disinfected by a team of disinfectors on the territory of the infectious diseases hospital on a special site equipped with a drain and a pit for disinfection of transport used for transporting patients in accordance with the current methodological documents. The internal and external surfaces of the transport module and vehicles are treated by spraying from a water gun with disinfectants approved for work with dangerous viruses in a concentration in accordance with the instructions.

Filter elements of the TIB and other medical waste are disposed of in accordance with the established procedure.

At the end of the patient's transportation, protective and work clothing is subjected to special treatment by soaking in a disinfectant solution according to the viral regimen in accordance with the instructions for use.

All team members are required to undergo sanitary treatment in a specially designated room of the infectious diseases hospital.

Members of the teams that carried out the medical evacuation are placed under medical observation for a period of 14 days.

в) Disinfection team activities

Disinfection measures are carried out taking into account the letter of Rospotrebnadzor dated 23.01.2020 No. 02/770-2020-32 “On instructions for carrying out disinfection measures to prevent diseases caused by coronaviruses”.

Upon arrival at the disinfection site, team members put on protective clothing. Final disinfection in the vehicle is carried out immediately after the patient has been evacuated.

To carry out disinfection, two team members enter the outbreak, one disinfector remains outside the outbreak. The latter is responsible for receiving items from the outbreak for chamber disinfection, preparing disinfectant solutions, and carrying the necessary equipment.

Before disinfection, it is necessary to close the windows and doors in the rooms to be treated. The final disinfection begins from the entrance door of the building, sequentially treating

all rooms, including the room where the patient was. In each room, from the threshold, without entering the room, generously spray the floor and air with a disinfectant solution.

The head of a medical organization in which a patient suspected of having COVID-19 has been identified carries out primary anti-epidemic measures in accordance with the operational plan of the medical organization, as in the event of identification of a patient with a particularly dangerous infection, in order to ensure timely information, temporary isolation, consultation, evacuation, disinfection, and provision of the patient with the necessary medical care in accordance with current regulatory documents and sanitary legislation.

In a medical organization providing medical care to patients and persons suspected of having COVID-19, according to sanitary rules, it is necessary to have:

- A minimum stock of personal protective equipment (protective clothing, masks, respirators, glasses/screens, gloves, etc.);
- Sets for collecting biological material from a patient (suspect);
- Kits with emergency prevention equipment for medical workers;
- A month's supply of disinfectants and equipment;
- Test systems for laboratory diagnostics in case of detection persons suspected of having coronavirus infection;
- Medical personnel trained in actions upon identification of a patient (suspected of having) COVID-19.

When using PPE, it is necessary to follow the requirements of sanitary rules. Used materials are disinfected and/or rendered harmless and subsequently buried or destroyed in accordance with the requirements for class B medical waste. Final and ongoing disinfection is carried out, including air disinfection.

9.4. PROCEDURE FOR HOSPITALIZATION OF PATIENTS IN MEDICAL ORGANIZATIONS DEPENDING ON THE SEVERITY OF THE DISEASE

1. Hospitalizations in medical organizations and their structural divisions providing medical care to patients with an established diagnosis of COVID-19 or with suspected COVID-19 in inpatient settings (hereinafter, respectively, the structural division of a medical organization for treatment

COVID-19, patients), are subject to patients with an established diagnosis of COVID-19 or with suspected COVID-19, who are in moderate, severe and extremely severe condition. It is allowed to provide medical care at home to adult patients with a moderate course of the disease, if the conditions are available.

2. Patients are hospitalized in a structural unit of a medical organization for the treatment of COVID-19, which has beds for patients in serious condition who do not require artificial ventilation, beds for patients in serious condition who require NIVL, and beds for patients in

extremely serious condition who require artificial ventilation.

3. Patients undergoing outpatient treatment with a body temperature of ≥ 38.5 °C for 3 days or more are hospitalized in a structural
a unit of a medical organization for the treatment of COVID-19 for beds for patients in moderate condition.

4. Patients in moderate condition are hospitalized in a structural unit of a medical organization for the treatment of COVID-19 in beds for patients in severe condition who do not require mechanical ventilation, based on the presence of two of the following criteria:

- $SpO2 < 95\%$;
- $t \geq 38$ °C;
- Respiratory rate > 22;
- . Patients in serious condition are hospitalized in a structural unit of a medical organization for the treatment of COVID-19 in beds for patients in serious condition requiring NIVL, based on the presence of two of the following criteria:
- $SpO2 \leq 93\%$;
- $t \geq 39$ °C;
- Respiratory rate ≥ 30;

Additional signs of a patient's serious condition include decreased level of consciousness, agitation, unstable hemodynamic parameters (systolic blood pressure < 90 mm Hg, diastolic blood pressure < 60 mm Hg).

6. Patients in critical condition are hospitalized in a structural unit of a medical organization for the treatment of COVID-19 in beds for patients in critical condition requiring mechanical ventilation, based on the presence of two of the following criteria:

- Impaired consciousness;
- $SpO2 < 92\%$ (with oxygen therapy);
- Respiratory rate > 35.

7. Patients in moderate, severe and extremely severe condition, as well as patients in the risk group (see below, item 8) are recommended to undergo chest CT or, if this is not possible, chest X-ray on the day of hospitalization. Ultrasound of the pleural cavities and lungs can only be used as a supplement to CT or X-ray to assess the dynamics of changes in the chest cavity. The frequency of repetition of these examination methods is determined by clinical indications. In the absence of negative clinical dynamics, control X-ray is performed no more than once a week.

8. The following are subject to hospitalization in a structural unit of a medical organization for the treatment of COVID-19 , regardless of the severity of the disease:

- Patients belonging to the risk group (over 65 years old, with concomitant diseases and conditions: arterial hypertension; chronic heart failure; oncological diseases;

hypercoagulability; disseminated intravascular coagulation; acute coronary syndrome; diabetes mellitus; motor neuron disease; liver cirrhosis; long-term use of corticosteroids and biological therapy for inflammatory bowel diseases; rheumatoid arthritis; patients receiving hemodialysis or peritoneal dialysis; immunodeficiency states, including those with HIV infection without antiretroviral therapy; receiving chemotherapy);

- Patients living in a dormitory, a crowded apartment, with people over 65 years of age, with people suffering from chronic diseases of the bronchopulmonary, cardiovascular and endocrine systems.

9. Patients specified in paragraph 8 who are in a mild condition are hospitalized in a structural unit of a medical organization for the treatment of COVID-19 in beds for patients in a moderate condition, based on the presence of two of the following criteria:

- $SpO_2 \geq 95\%$ (mandatory criterion) ;
- t < 38 °C;
- Respiratory rate ≤ 22.

10. Patients under 18 years of age are hospitalized in a structural unit of a medical organization for the treatment of COVID-19 if one of the following criteria is met:

- t > 39.0 °C on the day of treatment or t > 38 °C for 5 days or more;
- Respiratory failure (presence of any sign of the following symptoms of respiratory distress):
    - tachypnea: respiratory rate in children under 1 year of age is more than 50, from 1 to 5 years - more than 40, over 5 years - more than 30 per minute;
    - shortness of breath at rest or when the child is restless;
    - participation of accessory muscles in the act of breathing;
    - retraction of the compliant areas of the chest during breathing;
    - flaring of the wings of the nose when breathing;
    - grunting or groaning breathing;
    - episodes of apnea;
    - nodding movements of the head, synchronous with inhalation;
    - distant wheezing;
    - inability to suck/drink due to breathing problems;
    - acrocyanosis or central cyanosis;
    - $SpO_2 < 95\%$;
- Tachycardia in children under 1 year of age - more than 140, from 1 to 5 years - more than 130, over 5 years - more than 120 beats per minute;
- Presence of hemorrhagic rash;
- The presence of any of the following urgent and emergency signs:
    - convulsions;
    - shock;
    - severe respiratory failure;
    - severe dehydration;
    - depression of consciousness (drowsiness) or agitation;
- The presence of one of the following severe underlying diseases, regardless
- depending on the level of increase in T and the severity of respiratory failure:
    - immunodeficiency state, including treatment with immunosuppressive drugs;
    - oncological and oncohematological diseases;
    - diseases with disorders of the blood clotting system;
    - congenital and acquired heart defects and diseases, including rhythm disturbances, cardiomyopathy;
    - congenital and acquired chronic lung diseases;
    - endocrine system diseases (diabetes, obesity);

- motor neuron diseases (amyotrophic lateral sclerosis, spinal muscular atrophy, and others);
- chronic severe diseases of the liver, kidneys, gastrointestinal tract.

- Impossibility of isolation when living with persons belonging to a risk group (over 65 years old, with concomitant diseases and conditions: arterial hypertension; chronic heart failure; oncological diseases; hypercoagulability; DIC syndrome; acute coronary syndrome; diabetes mellitus; motor neuron diseases; liver cirrhosis; long-term use of GCS and biological therapy for inflammatory bowel diseases; rheumatoid arthritis; patients receiving hemodialysis or peritoneal dialysis; immunodeficiency states, including HIV infection without antiretroviral therapy; receiving chemotherapy);
- Lack of conditions for treatment at home or guarantees of compliance with recommendations (hostel, social welfare institutions, temporary accommodation center, socially disadvantaged family, unfavorable social and living conditions).

9.5. BASIC PRINCIPLES OF PROVIDING MEDICAL CARE IN OUTPATIENT SETTINGS (AT HOME)

The algorithm of actions of medical workers providing medical care in outpatient settings, including at home, to patients with acute respiratory viral infections is presented in Appendix 11.

Upon receipt of a positive result of laboratory tests of the patient's biological material for the presence of COVID-19 (hereinafter referred to as the COVID-19 test result), an authorized person of the medical organization:

- COVID-19 test result ;
- the head of a medical organization of a positive COVID-19 test result;
- COVID-19 patient registration log scheduled dates for repeated collection of biological material (nasopharyngeal and oropharyngeal swabs);
- Organizes examination of employees of a medical organization who have been in contact with a sick patient and, if symptoms of acute respiratory viral infection are detected, collection of biomaterial from them (nasal and oropharyngeal swabs) for laboratory testing for the presence of COVID-19;
- Conducts a patient survey to clarify his condition;
- Informs a healthcare worker sent to provide medical care to a patient about a positive COVID-19 test result.

Healthcare workers providing home care to patients who test positive for COVID-19 are required to:

- Use PPE (glasses, disposable gloves, respirator of the appropriate protection class, type 1 plague suit or disposable gown, shoe covers);
- Have a supply of at least 20 medical masks and offer them to the patient before beginning the interview and examination;

- Recommend that the patient wear a medical mask during examination and questioning by a health care professional;
- Treat your gloved hands with a disinfectant;
- While in the patient's apartment, do not remove PPE;
- B medical waste bag and ensure its further transportation for disposal in accordance with
  with requirements;
- Upon completion of providing medical care to a patient, inform the authorized person of the medical organization about persons who have had contact with the patient.

COVID-19 test result can be provided at home in the absence of clinical manifestations of the disease or in a mild course of the disease. Medical care is allowed to be provided at home to adult patients with a moderate course of the disease if the conditions are available.

- A patient who tests positive for COVID-19 receives the necessary treatment in accordance with these guidelines.
- The patient should be informed by a healthcare professional about the need to call a doctor or an ambulance team if their health deteriorates (body temperature > 38.5 °C for 3 days or more, difficulty breathing, shortness of breath, SpO2 < 93%), as well as about possible ways to seek medical help.
- Persons living in the same room with the patient should be informed about the risks of COVID-19 and the need to temporarily live elsewhere.
- The patient and persons living with him must be informed that violation of sanitary and epidemiological rules, which through negligence resulted in mass illness, may result in their being held criminally liable under Article 236 of the Criminal Code of the Russian Federation (Collected Legislation of the Russian Federation, 1996, No. 25, Art. 2954; 2011, No. 50, Art. 7362).
- The patient and persons living with him/her should be provided with information materials on issues of caring for patients with COVID-19 and general recommendations for protection against airborne and contact infections.
- If a decision is made to continue providing medical care to the patient on an outpatient basis (at home), consent is issued for the provision of medical care on an outpatient basis (at home) and compliance with the isolation regime during the treatment of COVID-19.

A patient who tests positive for COVID-19 is subject to hospitalization if one of the following circumstances exists:

- Mild course of the disease, if the patient is over 65 years old or has symptoms of acute respiratory viral infection in combination with chronic heart failure, diabetes, respiratory disease (bronchial asthma, chronic obstructive pulmonary disease). This category of

patients can receive medical care on an outpatient basis if conditions are available.

- Cohabitation with persons belonging to risk groups (persons over 65 years of age, as well as persons suffering from chronic diseases of the bronchopulmonary, cardiovascular and endocrine systems, pregnant women) and the impossibility of their resettlement regardless of the severity of the patient's disease;
- Mild course of the disease in children with symptoms of acute respiratory viral infection in combination with chronic diseases: heart failure, diabetes, bronchial asthma, congenital heart and lung defects, those undergoing immunosuppressive therapy, and others;

A medical institution where a child with a positive COVID-19 test result and no clinical manifestations of the disease is being observed,
ensures daily questioning by the district nurse (by telephone) about the patient’s condition at least twice a day, as well as home care by the district pediatrician at least once every 5 days.

A medical organization where a child with a positive COVID-19 test result and a mild course of the disease is being observed ensures a daily survey by a district nurse (by telephone) about the patient's condition at least 2 times a day, as well as home care by a district pediatrician (paramedic), taking into account the child's condition, at least once every 2 days.

COVID-19 test result who is receiving medical care at home must be informed of the risks of COVID-19 and the need to comply with recommendations for protection against airborne and contact-transmitted infections , the specifics of caring for patients with this infection, and also have information that a violation of sanitary and epidemiological rules that has inadvertently resulted in a mass disease may result in criminal liability under Article 236 of the Criminal Code of the Russian Federation.

9.6. ROUTING OF PATIENTS IN NEED IN EMERGENCY SURGICAL CARE

Emergency surgical care for inpatients with COVID -19

For patients undergoing inpatient treatment, specialized emergency surgical care is provided in a multidisciplinary infectious diseases hospital or a repurposed multidisciplinary hospital for providing care to patients with COVID-19 (in an operating unit equipped to perform surgeries on patients with COVID-19). If necessary, a specialized surgical team from another institution can be called to the infectious diseases hospital if there are no specialized specialists on site.

In some cases, the decision to transfer a patient with COVID-19 for emergency surgical care to another specialized infectious diseases hospital or a repurposed multidisciplinary hospital may be made on an individual basis, taking into account the possibility of safe transportation in the absence of a threat to life due to transportation. Specialized surgical teams can be used in cases where there are no specialists of the appropriate profile or qualifications in the medical institution.

Emergency surgical care for patients with COVID-19 treated at home

The patient is hospitalized for emergency surgical care in a multidisciplinary infectious diseases hospital or a repurposed multidisciplinary hospital for providing care to patients with COVID-19. If necessary, a specialized surgical team can be called to the hospital. Specialized surgical teams can be used in cases where the medical institution does not have specialists of the appropriate profile or qualifications. In other cases, emergency surgical care is provided by specialists of the multidisciplinary infectious diseases hospital.

Emergency surgical care for non- COVID-19 patients quarantined at home

The patient is transported for emergency care to a specialized surgical hospital that has the ability to isolate suspected (conditionally infected) patients with subsequent transfer to the infectious diseases department for patients with community-acquired pneumonia. The patient undergoes an emergency chest CT scan using appropriate safety measures for both the patient and medical personnel (use of PPE is mandatory) and laboratory testing for COVID-19.

The decision on patient routing should be made based on epidemiological and clinical data. In addition, test results cannot influence the decision on patient routing. In the absence of clinical symptoms of acute respiratory viral infection or pneumonia, emergency surgical care is provided in a specialized surgical hospital (with Meltzer boxes or an infectious diseases department for patients with community-acquired pneumonia). Medical care is provided by medical personnel who do not have contact with COVID-19 patients if the patient has been in quarantine for more than 14 days or has 2 negative COVID-19 tests.

If community-acquired pneumonia is detected or a positive test for COVID-19 is given, emergency surgical care is provided in a multidisciplinary infectious diseases hospital or a repurposed multidisciplinary hospital for providing care to patients with COVID-19, where, if necessary, a specialized surgical team can be called. In the event of a life-threatening situation and the impossibility of transportation, surgical care is provided on site, in compliance with all sanitary and epidemiological measures. In the postoperative period, the patient is placed in an isolated box or a boxed department, from where he is transferred to an infectious or

a repurposed multidisciplinary hospital to provide care to patients with COVID-19 after the patient's condition has stabilized.

Emergency surgical care for patients without COVID-19

The patient is transported by an ambulance team to a multidisciplinary clinic designated to provide emergency surgical care to patients without COVID-19, with the ability to isolate patients suspected of having community-acquired pneumonia or acute respiratory viral infection.

The patient is urgently given material for laboratory testing for COVID-19, if there are indications, X-ray or CT of the chest is performed (using appropriate safety measures for both the patient and the medical staff). The timing of the test and its results cannot affect the decision on patient routing. In the absence of clinical signs of ARVI, pneumonia, emergency surgical care is provided in a specialized surgical hospital (having Meltzer boxes or an infectious diseases

department for patients with community-acquired pneumonia). Medical care is provided by medical personnel who do not have contact with patients with COVID-19, if the patient has been in quarantine for more than 14 days or has 2 negative tests for COVID-19.

If community-acquired pneumonia, acute respiratory viral infection or a positive test for COVID-19 is detected , the patient is transferred for emergency surgical care to a multidisciplinary infectious diseases hospital or a repurposed multidisciplinary hospital for providing care to patients with COVID-19, where, if necessary, a specialized surgical team can be called.

In case of a life-threatening situation and impossibility of transportation, assistance is provided on site in compliance with all sanitary and epidemiological measures. In the postoperative period, the patient is placed in an isolated box or a boxed department, from where he is transferred to an infectious or repurposed multidisciplinary hospital to provide assistance to patients with COVID-19 after stabilization of the condition.

Precautions to ensure the safety of medical personnel when providing emergency surgical care

The generation of tiny liquid particles (aerosols) during medical procedures may pose a risk to healthcare workers due to the possible presence of SARS-CoV-2 in the aerosol. The following procedures are considered to have the potential to generate aerosols containing COVID-19:

- Upper gastrointestinal endoscopy, which involves open aspiration of the contents of the upper respiratory tract;
- Surgical operations using high-speed devices (saws, etc.);
- Some emergency dental procedures (eg, high-speed drilling);
- Non-invasive ventilation, such as bilevel positive airway pressure ventilation and continuous positive airway pressure ventilation, high-frequency oscillation ventilation;
- Stimulation of expectoration;
- High flow nasal oxygenation.

For patients with suspected or confirmed COVID-19, any of these potentially infectious aerosol-generating procedures should only be performed if clearly necessary.

It is recommended that ventilation in both laminar flow and conventional ventilated rooms be fully enabled during surgical procedures, especially if the patient is infected with SARS-CoV-2. Maximum ventilation in the operating room will protect medical personnel from infection with the virus. Air exhausted from the operating room into the adjacent hospital area will be highly diluted and should not be considered as a possible infectious agent.

Prevention of formation and release of tiny liquid particles (aerosols) during surgical interventions

Intraoperative release of surgical smoke may contain tiny virus particles. In order to reduce the release of surgical smoke, it is recommended to reduce the power of electrocoagulation as much as possible. A number of studies have shown that corynebacteria, papillomavirus and HIV are found in surgical smoke. It is known that the concentration of smoke during laparoscopic surgery

is significantly higher than during open interventions. Reducing pneumoperitoneum at the end of the operation can significantly reduce the risk of spreading tiny liquid particles, especially during abrupt removal of trocars and/or during replacement of laparoscopic instruments. From a technical point of view, intelligent flow systems should be used that allow maintaining intra-abdominal pressure at an extremely low level and evacuating smoke. Classic aspiration
systems, on the contrary, are at higher risk of transmitting the smallest particles of SARS-CoV-2.

It is preferable to minimize various manipulations of the intestine, as well as its opening during surgical procedures that do not involve it. This is especially important when performing laparoscopic manipulations to reduce the risk of viral diffusion in the $CO_{2\ \text{insufflation mode}}$.

To date, there have been no cases of transmission of the disease through urine, however, drainage of the bladder and/or ureter during surgery should be carried out with caution, especially in the presence of pneumoperitoneum.

Protection of medical personnel in operating rooms

The entire surgical team (including surgeons, anesthesiologists, and nurses) should use additional PPE when performing surgical interventions on patients with coronavirus infection. Protective glasses, FFP2/3 mask (including a mask with a protective visor), and protective clothing (coveralls) are essential items in case of any invasive surgery performed during the COVID-19 emergency. Surgeons should minimize any contact with the patient's body fluids. Thorough cleaning and disinfection of all equipment, including laparoscopic, endoscopic stands, and surgical consoles is also necessary.

10. RULES FOR FORMULATION OF DIAGNOSIS, CODING ACCORDING TO ICD-10 AND RECORDING OF PATIENTS WITH COVID-19 IN AN INFORMATION RESOURCE

To ensure reliable statistical recording in the presence of coronavirus infection in a patient, or suspicion of it, the final clinical, pathological and forensic diagnoses must be formulated in accordance with the rules of ICD-10.

In morbidity statistics, at the end of an episode of medical care, from several diseases present in a patient, all other things being equal, only one disease should be selected as the main one, which accounted for the largest share of the resources used (volume 2, p. 107).

Coding of statistical information in the presence of a suspicion or established diagnosis of COVID-19 is carried out in accordance with the following procedure:

U07.1 – Coronavirus infection COVID-19, virus identified
(confirmed by laboratory testing regardless of the severity of clinical signs or symptoms)

U07.2 – Coronavirus infection COVID-19, virus not identified (COVID-19 is diagnosed clinically or epidemiologically, but laboratory tests are inconclusive or unavailable)
Z03.8 – Observation for suspected coronavirus infection **Z22.8** – Carrier of the causative agent of coronavirus infection **Z20.8** – Contact with a patient with coronavirus infection

Z11.5 – Screening examination for the detection of coronavirus infection
B34.2 – Unspecified coronavirus infection (except COVID-19)
B33.8 – Specified coronavirus infection (except COVID-19)
Z29.0 – Insulation
U08.9 – Personal history of COVID-19
U09.9 – Post- COVID-19 status
U11.9 – Need for immunization against COVID-19
U12.9 – COVID-19 vaccine causing adverse reaction

In the presence of pneumonia caused by COVID-19, the categories **J12-J18** are used as additional codes. In case of fatal outcomes, the categories of class XXI **(Z00-Z99)** of ICD-10 are not used.

Primary medical documentation (Patient's coupon for receiving medical care in an outpatient setting - form No. 025-1/u; Statistical card of a patient discharged from hospital - form No. 066/u) is filled in according to the established procedure. Additional codes are entered manually in the upper right corner.

Examples of diagnosis formulation and coding of COVID-19 according to ICD-10:

Example 1.

Underlying disease: Coronavirus infection COVID-19, virus identified **(U07.1)**

Underlying disease: Type 2 diabetes mellitus with renal complications **(E11.2)** **Complications:** Bilateral polysegmental viral pneumonia.

Example 2. (only for stage clinical diagnosis).

Underlying disease: Contact with a patient with coronavirus infection **(Z20.8).**

Example 3.

Underlying disease: Coronavirus infection COVID-19, virus identified **(U07.1)**

Complications: Bilateral pneumonia. Sepsis.

Associated diseases: HIV- related disease , with tuberculosis and Kaposi's sarcoma **(B22.7)**

Example 4.

Underlying disease: Coronavirus infection COVID-19, virus not identified **(U07.2)**

Complications: Bilateral polysegmental viral pneumonia caused by SARS-CoV-2.

Associated diseases: Malignant neoplasm of the middle third of the body of the stomach, cT3N0M0 stage IIb (tubular adenocarcinoma G1) **(C16.2)**

The coding and selection of the underlying cause of death depends on the correct formulation of the final clinical, pathological or forensic diagnosis.

Different countries have different approaches to formulating a pathological diagnosis, choosing and recording the causes of death from COVID-19, which probably explains the

significant differences in mortality statistics. At the same time, international rules for coding COVID-19 in death certificates are presented in the WHO recommendations. Preliminary and final pathological diagnoses are formulated in accordance with the order of the Ministry of Health of Russia dated 06.06.2013 No. 354 "On the procedure for conducting pathological autopsies" and the clinical recommendations of the Russian Society of Pathologists "Formulation of a pathological diagnosis for some infectious and parasitic diseases" RPSA.1 (2016), with the final one - after completion of the histological examination and receipt of laboratory test results.

The rules and examples of formulating a diagnosis and coding the causes of death in the presence of a new coronavirus infection COVID-19 in a patient are set out in the “Methodological recommendations for coding and selecting the main condition in morbidity statistics and the underlying cause in mortality statistics associated with COVID-19” (in the current version).

In order to collect information about patients with a new coronavirus infection, as well as persons with pneumonia, including those undergoing outpatient treatment, an information system has been developed (hereinafter referred to as the information resource), which is located at: https://covid.egisz.rosminzdrav.ru/ .

In accordance with the Decree of the Government of the Russian Federation of 31.03.2020 No. 373 "On approval of temporary rules for recording information in order to prevent the spread of a new coronavirus infection (COVID-19)", it is necessary to ensure that medical organizations provide information on individuals diagnosed with a new coronavirus infection (COVID-19) and individuals with signs of pneumonia. Information is sent in accordance with the procedure set out in the instructions for entering information into the information resource, located at http://portal.egisz.rosminzdrav.ru/materials/3557 , within the established timeframes:

- Within 2 hours from the moment of diagnosis of a new coronavirus infection (COVID-19) or hospitalization of a patient with signs of pneumonia;
- Within 2 hours from the moment of receiving the results of laboratory tests.

When entering information about a patient, you must specify:

а) Date of onset of clinical symptoms;

б) Diagnosis (ICD-10 code is indicated);

в) Date of diagnosis;

г) Presence of concomitant (comorbid) diseases:

- Chronic diseases of the bronchopulmonary system;
- Chronic diseases of the cardiovascular system;
- Chronic diseases of the endocrine system;
- Oncological diseases;
- HIV infections;
- tuberculosis;
- other diseases.

д) Presence of pregnancy;

е) Vaccination details (influenza and pneumococcal infection (if any);

It is necessary to ensure that medical information about the patient is updated on a daily basis.

а) Information about the treatment being carried out:

- Antiviral treatment;
- Respiratory support (artificial ventilation, ECMO);

з) Blood oxygen saturation level;

и) Severity of the disease.

In case of death of the patient

а) The “Final clinical diagnosis” section is filled in within 24 hours:

- Underlying disease;
- Comorbid, competing, combined and background diseases;
- Complications (of the underlying disease, comorbid diseases) – if any;
- Concomitant diseases (if any).

б) Within 24 hours from the moment of the autopsy, the section “preliminary pathological (forensic) diagnosis” is filled out based on the results of the first stage of the pathological or forensic examination:

- Underlying disease;
- Comorbid, competing, combined and background diseases;
- Complications (of the underlying disease, comorbid diseases) – if any;
- Concomitant diseases (if any).
- A scanned copy of the first part of the autopsy report or an extract from the results of the external and internal forensic medical examination, containing pathological changes that formed the basis for the forensic medical diagnosis.

в) "Medical Death Certificate":

Part I

Line "a" Disease or condition directly leading to death;

Line "b" Pathological condition that led to the occurrence of the above cause (if any);

Line "c" or "b", if the latter was not filled in - Underlying cause of death;

Line "g" External cause in case of injuries and poisoning (if any);

Part II. Other important conditions that contributed to death, but were not related to the disease or pathological condition (if any);

г) After completion of the pathological or forensic examination, the section “final pathological (forensic) diagnosis” is filled in.

- A scanned copy of the medical death certificate.
- Underlying disease;
- Comorbid, competing, combined and background diseases;
- Complications (of the underlying disease, comorbid diseases) – if any;
- Concomitant diseases (if any).
- A scanned copy of the second part of the pathological examination protocol or an extract

from the results of additional laboratory tests in the case of a forensic medical examination;

д) When making changes to the first (preliminary or final) “Medical Death Certificate” (if necessary in the case of issuing a duplicate certificate to replace the preliminary or final one), attach a scanned copy of the duplicate medical death certificate.

To gain access to the information resource, you must send an application for access using the form provided in the instructions to the email address egisz@rt-eu.ru .

Sources Used

1. Abramowicz JS, Basseal J. WFUMB Position Statement : How to Safely Conduct ultrasound examination and disinfection of ultrasound equipment in the context of COVID-19 // Ultrasound and functional diagnostics. 2020. No. 1. P. 12–23. Doi: 10.24835/1607-0771-2020-1-12-23. Published ahead of print. Access mode: // http://www.rasudm.org/files/WFUMB-Position-Statement-COVID.pdf , free. Caption from the screen. 04/16/2020.
2. Ai T. et al. Correlation of Chest CT and RT-PCR Testing in Coronavirus Disease 2019 (COVID-19) in China: A Report of 1014 Cases [published online ahead of print, 2020 Feb 26] // Radiology. 2020. 200642. Doi: 10.1148/radiol.2020200642.
3. Al-Tawfiq JA, Memish ZA Update on therapeutic options for Middle East Respiratory Syndrome Coronavirus (MERS-CoV) // Expert review of anti-infective therapy. 2017. 15. No. 3. pp. 269–275.
4. Alserehi H. et al. Impact of Middle East respiratory syndrome coronavirus (MERS-CoV) on pregnancy and perinatal outcome // BMC Infect Dis. 2016. No. 16, p. 105
5. Assiri A. et al. Middle East respiratory syndrome coronavirus infection during pregnancy: a report of 5 cases from Saudi Arabia // Clin Infect Dis. 2016. No. 63. pp. 951-953
6. Baig AM et al .. Evidence of the COVID-19 Virus Targeting the CNS: Tissue Distribution, Host–Virus Interaction, and Proposed Neurotropic Mechanisms. ACS Chem. Neurosci. 2020. doi:10.1021/acschemneuro.0c00122.
7. Bassetti M. The Novel Chinese Coronavirus (2019-nCoV) Infections: challenges for fighting the storm https://doi.org/10.1111/eci.13209 URL: https://onlinelibrary.wiley.com/doi/abs/10.1111/eci.13209
8. Behzadi MA, Leyva-Grado VH Overview of Current Therapeutics and Novel Candidates Against Influenza, Respiratory Syncytial Virus, and Middle East Respiratory Syndrome Coronavirus Infections // Frontiers in microbiology. 2019. No. 10. p. 1327.
9. Beloncle F, Mercat A Approaches and Techniques to Avoid Development or Progression of Acute Respiratory Distress Syndrome Review Curr Opin Crit Care 2018 Feb;24(1):10-15. doi: 10.1097/MCC.00000000000000477.
10. Benefits, Open questions and Challenges of the use of Ultrasound in the COVID-19 pandemic era. The views of a panel of worldwide international experts [published online ahead of print, 2020 Apr 15] // Ultraschall Med. 2020;10.1055/a-1149-9872. Doi: 10.1055/a-1149-9872.
11. Bernheim A. et al. Chest CT Findings in Coronavirus Disease-19 (COVID-19): Relationship to Duration of Infection. Radiology. 2020:200463. doi:10.1148/radiol.2020200463.
12. Canada.ca. 2019 novel coronavirus: Symptoms and treatment The official website of the Government of Canada URL: https://www.canada.ca/en/public-health/services/diseases/2019-novel-coronavirusinfection/symptoms.html
13. Carmeli Y. et al. Health and economic outcomes of antibiotic resistance in Pseudomonas aeruginosa. Arch Intern Med. 1999 May 24;159(10):1127-32.
14. CDC. 2019 Novel Coronavirus URL: https://www.cdc.gov/coronavirus/2019-ncov/index.html
15. Chaolin H. et al. Clinical features of patients infected with 2019 novel coronavirus in Wuhan, China? Lancet 2020; 395: 497–506 Published Online January 24, 2020 https://doi.org/10.1016/ S0140-6736(20)30183-5
16. Chaomin Wu et al. Risk Factors Associated With Acute Respiratory Distress Syndrome and Death in Patients With Coronavirus Disease 2019 Pneumonia in Wuhan, China. JAMA Intern Med. Published online March 13, 2020. doi:10.1001/jamainternmed.2020.099
17. Chen N. et al. Epidemiological and clinical characteristics of 99 cases of 2019 novel coronavirus pneumonia in Wuhan, China: a descriptive study // Lancet. 2020. doi: 10.1016/S0140-6736(20)30211-7
18. China CDC. Diagnosis and treatment protocol for COVID-19 patients (trial version 7, revised)
19. Chong YP et al. Antiviral Treatment Guidelines for Middle East Respiratory Syndrome // Infection & chemotherapy 2015. 47. No. 3. pp. 212–222.
20. Chung M. et al. CT Imaging Features of 2019 Novel Coronavirus (2019-nCoV) // Radiology. 2020. V. 295. No. 1. P. 202–207. Doi: 10.1148/radiol.2020200230.
21. Cinatl J. et al. Treatment of SARS with human interferons // Lancet. 2003. 362. No. 9380. pp. 293–294.
22. Clinical management of severe acute respiratory infection when Middle East respiratory syndrome

coronavirus (MERS-CoV) infection is suspected: Interim Guidance. Updated 2 July 2015. WHO/MERS/Clinical/15.1

23. Colson, P., Rolain, J.M., Lagier, J.C., Brouqui, P., & Raoult, D. Chloroquine and hydroxychloroquine as available weapons to fight COVID-19 International Journal of Antimicrobial Agents 2020.
24. Commonwealth of Australia | Department of Health. Novel coronavirus (2019-nCoV) URL: https://www.health.gov.au/health-topics/novel-coronavirus-2019-ncov
25. Corman VM et al. Detection of 2019 novel coronavirus (2019-nCoV) by real-time RT-PCR //Eurosurveillance. 2020. (3)25. doi: 10.2807/1560-7917.ES
26. Coronavirus. URL :https://multimedia.scmp.com/widgets/china/wuhanvirus/?fbclid=IwAR2hDHzpZEh5Nj360i2O%201E S 78rXRFymAaFaUK6ZG4m0UTCV1xozulxX1jio COVID Care Protocol. Last Updated 05-11-2020 3:15 pm https://www.evms.edu/covid-19/covid_care_for_clinicians/#covidcare
27. Cortegiani, A., Ingoglia, G., Ippolito, M., Giarratano, A., & Einav, S. (2020). A systematic review on the efficacy and safety of chloroquine for the treatment of COVID-19. Journal of Critical Care .
28. Critical Care COVID-19 Management Protocol (updated 4-15-2020).
29. Dayer MR et al. Lopinavir; A Potent Drug against Coronavirus Infection: Insight from Molecular Docking Study // Arch Clin Infect Dis. 2017; 12(4):e13823. doi: 10.5812/archcid.13823
30. Devaux CA, Rolain JM, Colson P, Raoult D. New insights on the antiviral effects of chloroquine against coronavirus: what to expect for COVID-19? Int J Antimicrob Agents. 2020 Mar 12:105938. doi: 10.1016/j.ijantimicag.2020.105938.
31. Dyall J. et al. Middle East Respiratory Syndrome and Severe Acute Respiratory Syndrome: Current Therapeutic Options and Potential Targets for Novel Therapies // Drugs. 2017. 77. No. 18. P. 1935–1966.
32. European Commission. Novel coronavirus 2019-nCoV URL: https://ec.europa.eu/health/coronavirus_en
33. Fan HH. et al. Repurposing of clinically approved drugs for treatment of coronavirus disease 2019 in a 2019-novel coronavirus (2019-nCoV) related coronavirus model. Chin Med J (Engl). 2020 Mar 6. doi: 10.1097/CM9.0000000000000797
34. Fang Y. et al. Sensitivity of Chest CT for COVID-19: Comparison to RT-PCR [published online ahead of print, 2020 Feb 19] // Radiology. 2020. 200432. Doi: 10.1148/radiol.2020200432.
35. FDA. Novel coronavirus (2019-nCoV) URL: https://www.fda.gov/emergency-preparedness-andresponse/mcm-issues/novel-coronavirus-2019-ncov
36. Federal Ministry of Health. Current information on the coronavirus URL: https://www.bundesgesundheitsministerium.de/en/en/press/2020/coronavirus.html
37. Franquet T. Imaging of pulmonary viral pneumonia // Radiology. 2011. V. 260. No. 1. P. 18–39. Doi: 10.1148/radiol.11092149.
38. Gao, J., Tian, Z., & Yang, X. Breakthrough: Chloroquine phosphate has shown apparent efficacy in the treatment of COVID-19 associated pneumonia in clinical studies. BioScience Trends 2020.
39. Gautret F., Lagier JC., Parola P. et al. Hydroxychloroquine and azithromycin as a treatment of COVID19: results of an open label non-randomized clinical trial. International Journal of Antimicrobial Agents. In Press 17 March 2020 –DOI : 10.1016/j.ijantimicag.2020.105949
40. Gorbalenya AEet al. Severe acute respiratory syndrome-related coronavirus: The species and its viruses – a statement of the Coronavirus Study Group, 2020. doi: https://doi.org/10.1101/2020.02.07.937862
41. Halyabar O. et al. Calm in the midst of cytokine storm: a collaborative approach to the diagnosis and treatment of hemophagocytic lymphohistiocytosis and macrophage activation syndrome, Pediatric Rheumatology, https://ped-rheum.biomedcentral.com/articles/10.1186/s12969-019-0309-6
42. Hart BJ et al. Interferon-β and mycophenolic acid are potent inhibitors of Middle East respiratory syndrome coronavirus in cell-based assays // The Journal of general virology. 2014. 95. Pt 3. pp. 571–577.
43. Henderson LA et al. On the Alert for Cytokine Storm: Immunopathology in COVID-19 Arthritis & Rheumatology Vol. 0, No. 0, Month 2020, pp 1–5 DOI 10.1002/art.41285
44. https://grls.rosminzdrav.ru/Grls_View_v2.aspx?routingGuid=4a3c02bc-a256-48f2-90a8-14b48b7d197c&t=
45. https://www.vmeda.org/wp-content/uploads/2020/03/koronavirus-metod-rekomendaczii.pdf
46. Huang C. et al. Clinical features of patients infected with 2019 novel coronavirus in Wuhan, China // Lancet. 2020 doi: 10.1016/S0140-6736(20)30183-5. [Epub ahead of print]
47. Infectious Diseases Society of America Guidelines on the Treatment and Management of Patients with COVID-19 . Published by IDSA, https://www.idsociety.org/practice-guideline/covid-19-guideline-treatment-and-management/ , [COVID-19 Guideline, Part 1: Treatment and Management]
48. Inui S. et al. Chest CT Findings in Cases from the Cruise Ship “Diamond Princess” with Coronavirus Disease 2019 (COVID-19). Radiology: Cardiothoracic Imaging. 2020;2:e200110. doi:10.1148/ryct.2020200110.
49. Jeong SY et al. MERS-CoV Infection in a Pregnant Woman in Korea. J Korean Med Sci. 2017 Oct;32(10):1717-1720. doi: 10.3346/jkms.2017.32.10.1717.
50. Jesú V. et al. Rationale for Prolonged Corticosteroid Treatment in the Acute Respiratory Distress Syndrome Caused by Coronavirus Disease 2019. critical Care Explorations: April 2020 - Volume 2 - Issue 4 -

p e0111 doi: 10.1097/CCE.0000000000000111
51. Ji W. et al. Homologous recombination within the spike glycoprotein of the newly identified coronavirus may boost cross-species transmission from snake to human // Journal of Medical Virology. – 2020.
52. Junqiang L. et al. CT Imaging of the 2019 Novel Coronavirus (2019-nCoV) Pneumonia https://doi.org/10.1148/radiol.2020200236 URL: https://pubs.rsna.org/doi/10.1148/radiol.2020200236
53. Kligerman SJ, Franks TJ, Galvin JR From the radiologic pathology archives: organization and fibrosis as a response to lung injury in diffuse alveolar damage, organizing pneumonia, and acute fibrinous and organizing pneumonia // Radiographics. 2013. V. 33. No. 7. P. 1951–1975. Doi: 10.1148/rg.337130057.
54. Kumar B. et al. A Personalized Diagnostic and Treatment Approach for Macrophage Activation Syndrome and Secondary Hemophagocytic Lymphohistiocytosis in Adults. Journal of Clinical Immunology, volume 37, pages 638–643(2017), https://link.springer.com/article/10.1007/s10875-017- 0439-x
55. Le Chang et al. Coronavirus Disease 2019: Coronaviruses and Blood Safety. Transfusion Medicine Reviews 2020. doi:10.1016/j.tmrv.2020.02.003.
56. Lee Kyung Soo. Pneumonia Associated with 2019 Novel Coronavirus: Can Computed Tomographic Findings Help Predict the Prognosis of the Disease? Korean Journal of Radiology(2020), 21(3):257
57. Li Q et al. Early Transmission Dynamics in Wuhan, China, of Novel Coronavirus-Infected Pneumonia N Engl J Med. 2020 Jan 29. doi: 10.1056/NEJMoa2001316
58. Li X et al. Potential of large 'first generation' human-to-human transmission of 2019-nCoV. J Med Virol. 2020 Jan 30. doi: 10.1002/jmv.25693. [Epub ahead of print]
59. Lu H. Drug treatment options for the 2019-new coronavirus (2019-nCoV). Biosci Trends. 2020 Jan 28. doi: 10.5582/bst.2020.01020. [Epub ahead of print]
60. Lu W., Zhang S., Chen B. et al. A Clinical Study of Noninvasive Assessment of Lung Lesions in Patients with Coronavirus Disease-19 (COVID-19) by Bedside Ultrasound [published online ahead of print, 2020 Apr 15] // Ultraschall Med. 2020;10.1055/a-1154-8795. Doi: 10.1055/a-1154-8795.
61. Ludvigsson JF. Systematic review of COVID-19 in children show milder cases and a better prognosis than adults https://doi.org/10.1111/apa.15270
62. Lupia T., Scabini S., Pinna SM, Di Perri G., De Rosa FG, Corcione S. 2019 novel coronavirus (2019- nCoV) outbreak: A new challenge, Journal of Global Antimicrobial Resistance 21 (2020) 22–27
63. Mandell LA et al. Infectious Diseases Society of America/American Thoracic Society consensus guidelines on the management of community-acquired pneumonia in adults //Clinical infectious diseases. – 2007. – T. 44. – No. Supplement_2. – pp. S27-S72.
64. Mao L. et al. Neurological Manifestations of Hospitalized Patients with COVID-19 in Wuhan, China: A retrospective case series study; 2020. doi: https://doi.org/10.1101/2020.02.22.20026500
65. Maude SL et al. Managing Cytokine Release Syndrome Associated With Novel T Cell-Engaging Therapies Cancer J. 2014 Mar-Apr; 20(2): 119–122. doi: 10.1097/PPO.0000000000000035
66. Mehta P, et al. COVID-19: consider cytokine storm syndromes and immunosuppression. Lancet. 2020 Mar 28;395(10229):1033-1034. doi: 10.1016/S0140-6736(20)30628-0. Epub 2020 Mar 16.
67. Ministère des Solidarités et de la Santé Coronavirus: questions-réponses URL: https://solidaritessante.gouv.fr/soins-et-maladies/maladies/maladies-infectieuses/coronavirus/coronavirus-questionsreponses
68. Mo Y., Fisher DA review of treatment modalities for Middle East Respiratory Syndrome // The Journal of antimicrobial chemotherapy. 2016. 71. No. 12. pp. 3340–3350.
69. Momattin H. et al. Therapeutic options for Middle East respiratory syndrome coronavirus (MERS-CoV) - possible lessons from a systematic review of SARS-CoV therapy. Int J Infect Dis. 2013 Oct;17(10):e7928
70. National Health Commission of the People's Republic of China. URL: http://en.nhc.gov.cn
71. Netland J. Severe Acute Respiratory Syndrome Coronavirus Infection Causes Neuronal Death in the Absence of Encephalitis in Mice Transgenic for Human ACE2. J Virol. 2008;82:7264–75. doi:10.1128/JVI.00737-08.
72. NHS. Coronavirus (2019-nCoV) URL: https://www.nhs.uk/conditions/wuhan-novel-coronavirus/
73. Omrani AS et al. Ribavirin and interferon alfa-2a for severe Middle East respiratory syndrome coronavirus infection: a retrospective cohort study //The Lancet Infectious Diseases. 2014. T. 14. No. 11. pp. 1090-1095.
74. Outbreak of acute respiratory syndrome associated with a novel coronavirus, China: first local transmission in the EU/EEA - third update URL: https://www.ecdc.europa.eu/sites/default/files/documents/novel-coronavirus-risk-assessment-china-31january-2020_0.pdf
75. Pan F. et al. Time Course of Lung Changes On Chest CT During Recovery From 2019 Novel Coronavirus (COVID-19) Pneumonia [published online ahead of print, 2020 Feb 13] // Radiology. 2020. 200370. Doi: 10.1148/radiol.2020200370.
76. Park MH et al. Emergency cesarean section in an epidemic of the Middle East respiratory syndrome: a case report Korean J Anesthesiol, 69 (2016), pp. 287-291, doi: 10.4097/kjae.2016.69.3.287
77. Paul La Rosee et al. Recommendations for the management of hemophagocytic lymphohistiocytosis in adults. Blood (2019) 133(23):2465–2477. https://doi.org/10.1182/blood.2018894618
78. Phan LT et al. Importation and Human-to-Human Transmission of a Novel Coronavirus in Vietnam //New England Journal of Medicine. – 2020.

79. Phylogeny of SARS-like betacoronaviruses including novel coronavirus (nCoV). URL: https://nextstrain.org/groups/blab/sars-like-cov
80. Pogue J., Kaye K., Veve M., et al. "Real World" treatment of multi-drug resistant (MDR) or extensively-drug resistant (XDR) P. aeruginosa infections with ceftolozane/tazobactam (C/T) versus a polymyxin or aminoglycoside (Poly/AG) based regimen: a multicenter comparative effectiveness study. Proceedings of the IDweek 2018, October 3-7, San Francisco, USA. Poster/Abstract 2406.
81. Public Health England. Investigation and initial clinical management of possible cases of novel coronavirus (2019-nCoV) infection URL: https://www.gov.uk/government/publications/wuhan-novelcoronavirus-initial-investigation-of-possible-cases/investigation-and-initial-clinical-management-ofpossible-cases-of-wuhan-novel-coronavirus-wn-cov-infection
82. Revel M.-P., Parkar A.P., Prosch H. et al. COVID-19 patients and the Radiology department – advice from the European Society of Radiology (ESR) and the European Society of Thoracic Imaging (ESTI) // Eur. Radio. Accepted on April 2, 2020.
83. Richardson P, Griffin I, Tucker C, Smith D, Oechsle O, Phelan A, Stebbing J. Baricitinib as potential treatment for 2019-nCoV acute respiratory disease. Lancet. 2020 Feb 15;395(10223):e30-e31. doi: 10.1016/S0140-6736(20)30304-4.
84. Rodriguez-Morales A. et al. Clinical, laboratory and imaging features of COVID-19: A systematic review and meta-analysis. Travel Medicine and Infectious Disease, Volume 34, March–April 2020, 101623 https://doi.org/10.1016/j.tmaid.2020.101623
85. Royal Pharmaceutical Society of Great Britain Trading as Royal Pharmaceutical Society. Wuhan novel coronavirus URL: https://www.rpharms.com/resources/pharmacy-guides/wuhan-novel-coronavirus
86. Rubin GD, Ryerson CJ, Haramati LB et al. The Role of Chest Imaging in Patient Management during the COVID-19 Pandemic: A Multinational Consensus Statement from the Fleischner Society [published online ahead of print, 2020 Apr 7] // Radiology. 2020;201365. Doi: 10.1148/radiol.2020201365.
87. Seif F, Aazami H, Khoshmirsafa M, Kamali M, Mohsenzadegan M, Pornour M, Mansouri D. JAK Inhibition as a New Treatment Strategy for Patients with COVID-19. Int Arch Allergy Immunol. 2020 May 11:1-9. doi: 10.1159/000508247.
88. Shannon L. Maude et al. Chimeric Antigen Receptor T Cells for Sustained Remissions in Leukemia. N Engl J Med 2014;371:1507-17. DOI: 10.1056/NEJMoa1407222
89. Simone S. et al. Acute myocarditis presenting as a reverse Tako-Tsubo syndrome in a patient with SARS-CoV-2 respiratory infection. European Heart Journal, Volume 41, Issue 19, 14 May 2020, Pages 1861– 1862, https://doi.org/10.1093/eurheartj/ehaa286
90. Soldati G., Smargiassi A., Inchingolo R. et al. Proposal for International Standardization of the Use of Lung Ultrasound for Patients With COVID-19: A Simple, Quantitative, Reproducible Method [published online ahead of print, 2020 Mar 30] // J. Ultrasound Med. 2020;10.1002/jum.15285. Doi: 10.1002/jum.15285.
91. Spinelli FR, Conti F, Gadina M. HiJAKing SARS-CoV-2? The potential role of JAK inhibitors in the management of COVID-19. Sci Immunol. 2020 May 8;5(47). piieabc5367. doi: 10.1126/sciimmunol.abc5367.
92. Tang N, Bai H, Chen X, Gong J, Li D, Sun Z. Anticoagulant treatment is associated with decreased mortality in severe coronavirus disease 2019 patients with coagulopathy. J Thromb Haemost. 2020 Mar 27. doi: 10.1111/jth.14817.
93. The Centers for Disease Control and Prevention (CDC). Interim guidance for healthcare professionals on human infections with 2019 novel coronavirus (2019-nCoV). URL: https://www.cdc.gov/coronavirus/2019-nCoV/hcp/index.html
94. The State Council The People's Republic Of China URL: http://english.www.gov.cn/
95. Touret, F., de Lamballerie, X. Of chloroquine and COVID-19. Antiviral Research, 104762.
96. Upchurch CP et al. Community-acquired pneumonia visualized on CT scans but not chest radiographs: pathogens, severity, and clinical outcomes //Chest. – 2018. – T. 153. – No. 3. – pp. 601-610.
97. Villar J, et al.; Dexamethasone treatment for the acute respiratory distress syndrome: a multicentre, randomized controlled trial. Lancet Respir Med. 2020 Feb 7.
98. Wang L C-reactive Protein Levels in the Early Stage of COVID-19 Med Mal Infect. 2020 Mar 31;S0399-077X(20)30086-X. doi: 10.1016/j.medmal.2020.03.007. Online ahead of print
99. Wang Z. et al. Clinical characteristics and therapeutic procedure for four cases with 2019 novel coronavirus pneumonia receiving combined Chinese and Western medicine treatment. Biosci Trends 2020. doi:10.5582/bst.2020.01030.
100. World Health Organization. COVID-19 Clinical management: living guidance. 25 January 2021. https://www.who.int/publications/i/item/WHO-2019-nCoV-clinical-2021-1
101. World Health Organization. Infection prevention and control guidance for long-term care facilities in the context of COVID-19: interim guidance, 21 March 2020. World Health Organization; 2020.
102. World Health Organization. Managing Ethical Issues in Infectious Disease Outbreaks. Publication date: 2016. URL: https://www.who.int/ethics/publications/infectious-disease-outbreaks/en/
103. World Health Organization. WHO Blood Regulators Network (BRN) Position Paper on Use of Convalescent

Plasma, Serum or Immune Globulin Concentrates as an Element in Response to an Emerging Virus* September 2017
https://www.who.int/bloodproducts/brn/2017_BRN_PositionPaper_ConvalescentPlasma.pdf
104. Wu P. et al. Real-time tentative assessment of the epidemiological characteristics of novel coronavirus infections in Wuhan, China, as at 22 January 2020 //Eurosurveillance. 2020. T. 25. No. 3. doi: 10.2807/1560-7917.ES.2020.25.3.2000044
105. Xiao A., et al J Clin Pharm. 2016; 56(1): 56-66; Luzelena Caro, David P. Nicolau, Jan J. De Waele et al. Lung penetration, bronchopulmonary pharmacokinetic/ pharmacodynamic profile and safety of 3 g of ceftolozane/tazobactam administered to ventilated, critically ill patients with pneumonia. Journal of Antimicrobial Chemotherapy, dkaa049, https://doi.org/10.1093/jac/dkaa049 Published: 24 March 2020
106. Xu L, Liu J, Lu M, Yang D, Zheng X. Liver injury during highly pathogenic human coronavirus infections. Liver Int. 2020 May;40(5):998-1004. doi: 10.1111/liv.14435. Epub 2020 Mar 30. Review. PubMed PMID: 32170806
107. Yuan M. et al. Association of radiological findings with mortality of patients infected with 2019 novel coronavirus in Wuhan, China. PLoS ONE. 2020;15:e0230548. doi:10.1371/journal.pone.0230548.
108. Zhang J. et al. Therapeutic and triage strategies for 2019 novel coronavirus disease in fever clinics. Lancet Respir Med 2020. doi:10.1016/S2213-2600(20)30071-0.
109. Zhang L, Liu Y. Potential Interventions for Novel Coronavirus in China: J Med Virol 2020. doi:10.1002/jmv.25707.
110. Zhang W, Zhao Y, Zhang F, Wang Q, Li T, Liu Z, Wang J, Qin Y, Zhang X, Yan X, Zeng X, Zhang S. The use of anti-inflammatory drugs in the treatment of people with severe coronavirus disease 2019 (COVID-19): The experience of clinical immunologists from China.
111. Zhang W. et al. The use of anti-inflammatory drugs in the treatment of people with severe coronavirus disease 2019 (COVID-19): The Perspectives of clinical immunologists from China. Clin Immunol. 2020 Mar 25;214:108393. doi: 10.1016/j.clim.2020.108393. [Epub ahead of print]
112. Zumla A. et al. Coronaviruses - drug discovery and therapeutic options // Nature reviews. Drug discovery. 2016. 15. No. 5. pp. 327–347.
113. World Health Organization. Interim guidance on the rational use of personal protective equipment against coronavirus disease (COVID-19) and considerations for situations of acute shortages. 6 April 2020. URL: https://apps.who.int/iris/bitstream/handle/10665/331695/WHO-2019-nCov-IPC_PPE_use-2020.3- rus.pdf
114. World Health Organization. Clinical guidelines for the management of severe acute respiratory infections when MERS-CoV infection is suspected. Interim recommendations. Publication date: July 2015. URL: https://www.who.int/csr/disease/coronavirus_infections/case-management-ipc/ru/
115. Kuzmenkov A.Yu., Trushin I.V., Avramenko A.A., Eidelshtein M.V., Dekhnich A.V., Kozlov R.S. AMRmap: Internet platform for monitoring antibiotic resistance. Clinical microbiology and antimicrobial chemotherapy. 2017;19(2):84-90.
116. Levshin N.Yu., Baranov A.A., Arshinov A.V. Second-generation low-molecular-weight heparin: efficacy, safety, and motivation for priority use in clinical practice. Difficult patient. 2014. - No. 6. P. 7-14
117. Radiation diagnostics of coronavirus disease (COVID-19): organization, methodology, interpretation of results: preprint No. CDT - 2020 - I. Version 2 of 04/17/2020 / compiled by S. P. Morozov, D. N. Protsenko, S. V. Smetanina [et al.] // Series "Best practices in radiation and instrumental diagnostics". - Issue. 65. - M.: State Budgetary Healthcare Institution "Research and Clinical Center for Radiology and T, Department of Health of the City of Moscow", 2020. - 78 p.
118. Materials of the analysis of teleconsultations of the NMIC FPI of the Ministry of Health of Russia.
119. Mitkov V.V., et al. RASUDM Consensus Statement on Ultrasound Examination of the Lungs in the Context of COVID-19 (version 1) // Ultrasound and Functional Diagnostics. 2020. No. 1. pp. 24–45. Doi: 10.24835/1607-0771-2020-1-24-45. Published ahead of print. Access mode: // http://www.rasudm.org/files/RASUDM-Consensus-Statement-COVID.pdf , free. Title. from the screen. 04/16/2020.
120. Portal of continuous medical and pharmaceutical education of the Ministry of Health of Russia URL: https://nmfo -vo.edu.rosminzdrav.ru/#/user-account/view-iom/e8b1f2ca-6be5-9125-4a1e
121. Federal Service for Surveillance on Consumer Rights Protection and Human Wellbeing. Methodological recommendations MR 3.1.0140-18 "Non-specific prevention of influenza and other acute respiratory infections".
122. Khristenko E.A. et al. CT patterns in COVID-19 associated pneumonia – standardization of study descriptions based on the Fleischner Society glossary // REJR. 2020. Vol. 10. No. 1. Pp. 16–26.
123. Sinitsyn V.E., Tyurin I.E., Mitkov V.V. Temporary consensus guidelines of the Russian Society of Roentgenologists and Radiologists (ROSR) and the Russian Association of Specialists in Ultrasound Diagnostics in Medicine (RASUDM) "Methods of radiation diagnostics of pneumonia in the new coronavirus infection COVID-19" (version 2). Bulletin of Roentgenology and Radiology. 2020; 101 (2): 72–89. https://doi.org/10.20862/0042-4676-2020-101-2-
124. Order of the Ministry of Health of the Russian Federation of December 29, 2012 No. 1705n "On the Procedure for Organizing Medical Rehabilitation".

125. Julie K. Silver: Prehabilitation could save lives in a pandemic March 19, 2020
126. Robert Simpson et al. Rehabilitation Following Critical Illness in People With COVID-19 Infection Am J Phys Med Rehabil2020 Apr 10. doi: 10.1097/PHM.0000000000001443.Online ahead of print
127. Thomas P et al. Physiotherapy management for COVID-19 in the acute hospital setting: clinical practice recommendations, Journal of Physiotherapy (2020), doi: https://doi.org/10.1016/j.jphys.2020.03.011 .
128. Kress, JP and JB Hall, ICU-acquired weakness and recovery from critical illness. N Engl J Med, 2014. 370(17): p. 1626-35.
129. Belkin A.A. et al. Rehabilitation in intensive care. Clinical guidelines, 2017, 56 p.
130. David C et al. Postacute Care Preparedness for COVID-19Thinking Ahead Author Affiliations Article Information JAMA. Published online March 25, 2020. doi:10.1001/jama.2020.4686
131. Michele Vitacca et al. Joint statement on the role of respiratory rehabilitation in the COVID-19 crisis: the Italian position paper Version - March 08, 2020. Graphics and publishing AIPO Ricerche Ed. - Milano edizioni@aiporicerche.it
132. Verticalization of patients during rehabilitation. Clinical recommendations. Moscow, 2014, 63 p.
133. Medrinal C et al. Comparison of exercise intensity during four early rehabilitation techniques in sedated and ventilated patients in ICU: A randomized cross-over trial. Crit Care. 2018; 22(1):110.
134. Karatzanos E et al. Electrical muscle stimulation: An effective form of exercise and early mobilization to preserve muscle strength in critically ill patients. Crit Care Res Pract. 2012; 2012: 432752.
135. Fossat G et al. Effect of in-bed leg cycling and electrical stimulation of the quadriceps on global muscle strength in critically ill adults: A Randomized Clinical Trial. JAMA. 2018; 320(4):368–378.
136. Henk J Stam et al. Covid-19 and Post Intensive Care Syndrome: A Call for Action. J Rehabil Med 2020 Apr 15;52(4):jrm00044 doi: 10.2340/16501977-2677.
137. Zabolotskikh I.B. et al. Anesthesiology and resuscitation care for patients with the new coronavirus infection COVID-19. Guidelines of the All-Russian public organization "Federation of Anesthesiologists and Resuscitators". Bulletin of Intensive Therapy named after A.I. Saltanov. 2020; S1:9–120. https://doi.org/10.21320/1818-474X-2020-S1-9-120
138. Bein T et al. S2e guideline: positioning and early mobilization in prophylaxis or therapy of pulmonary disorders Revision. Anaesthesist. 2015;64(September):1-26. doi:10.1007/s00101-015-0071-1.
139. Taito S et al. Early mobilization of mechanically ventilated patients in the intensive care unit. J Intensive Care. 2016;4(1):50. doi:10.1186/s40560-016-0179-7.
140. Eggmann S et al. Physiological effects and safety of an early, combined endurance and resistance training in mechanically ventilated, critically ill patients. Physiotherapy. 2015;101:e344-e345. doi:10.1016/j.physio.2015.03.553
141. Olenskaya T.L. et al. Rehabilitation in pulmonology. Study guide. – Vitebsk, 2016. – pp. 90-104.
142. Yang Feng et al. Pulmonary rehabilitation guidelines in the principle of 4S for patients infected with 2019 novel coronavirus (2019-nCoV). Cite as Chin J TubercRespir Dis. 2020: 43 (03): 180-182. DOI: 10.3760/cma.j.issn.1001-0939.2020.03.007.
143. Robert Simpson et al. Rehabilitation Following Critical Illness in People With COVID-19 Infection Am J Phys Med Rehabil2020 Apr 10. doi: 10.1097/PHM.0000000000001443.Online ahead of print
144. Spruit MA et al. An official american thoracic society/european respiratory society statement: key concepts and advances in pulmonary rehabilitation. Am J RespirCrit Care Med. 2013;188(8):e13–64.
145. Nasonov EL et al.; on behalf of the Presidium of the All-Russian Public Organization "Association of Rheumatologists of Russia". Draft recommendations of the All-Russian Public Organization "Association of Rheumatologists of Russia". Coronavirus disease 2019 (COVID-19) and immunoinflammatory (autoimmune) rheumatic diseases. Available at: https://rheumatolog.ru
146. Schulze-Koops H, et al. Preliminary recommendations of the German Society of Rheumatology (DGRh eV) for the management of patients with inflammatory rheumatic diseases during the SARS-CoV-2/Covid-19 pandemic. Ann Rheum Dis. 2020 Apr 28. pii: annrheumdis-2020-217628. doi: 10.1136/annrheumdis-2020-217628
147. Ceribelli A, et al. Recommendations for coronavirus infection in rheumatic diseases treated with biological therapy. J Autoimmun. May 2020 ; 109:102442. doi: 10.1016/j.jaut.2020.102442
148. Mikuls TR, et al. American College of Rheumatology guidance for the management of adult patients with rheumatic disease during the COVID-19 pandemic. Arthritis Rheumatol doi: https://onlinelibrary.wiley.com/doi/abs/10.1002/art.41301 .
149. COVID-19 rapid guideline: rheumatological autoimmune, inflammatory and metabolic bone diseases. NICE guideline, published 3 April 2020. www.nice.org./uk/guidancr/ng167
150. Kimberly Showalter, et al. Advice on Treating Rheumatic Diseases From a COVID-19 Epicenter - Medscape - May 11, 2020.

151. COVID-19: guidance for rheumatologists. British Society of Rheumatology. http://www.rheumatology.org.uk
152. Kuzkov V.V., Kirov M.Yu. Invasive monitoring of hemodynamics in intensive care and anesthesiology. Arkhangelsk, 2015. - 392 p.
153. M. R. Mehra, et al. Hydroxychloroquine or chloroquine with or without a macrolide for treatment of COVID-19: a multinational registry analysis, Lancet, 2020
154. Favipiravir versus Arbidol for COVID-19: A Randomized Clinical Trial. https://doi.org/10.1101/2020.03.17.20037432
155. Furuta Y, et al. : Favipiravir (T-705), a broad spectrum inhibitor of viral RNA polymerase. Proc Jpn Acad Ser B Phys Biol Sci. 2017;93(7):449-463. doi: 10.2183/pjab.93.027.
156. Q. Cai, et al. Experimental Treatment with Favipiravir for COVID-19: An Open-Label Control Study, Engineering (2020), doi: https://doi.org/10.1016/j.eng.2020.03.007
157. Pathological anatomy of COVID-19: Atlas / Zairatyants O. V., Samsonova M. V., Mikhaleva L. M., Chernyaev A. L., Mishnev O. D., Krupnov N. M., Kalinin D. V. Under the general editorship of O. V. Zairatyants. - Moscow, State Budgetary Institution "Research Institute of Health Protection of the Russian Federation, Moscow Department of Health", 2020. - 140 p., ill.
158. M. Ackermann, S. Verleden, M. Kuehnel, et al. Pulmonary Vascular Endothelialitis, Thrombosis, and Angiogenesis in Covid-19 DOI: 10.1056/NEJMoa2015432
159. S. E. Fox, A. Akmatbekov, J. L. Harbert, et al. Pulmonary and cardiac pathology in African American patients with COVID-19: an autopsy series from New Orleans. Lancet Respir Med 2020 doi:10.1016/S2213-2600(20)30244-7
160. P. Masi, G. Hekimian, M. Lejeune, et al. Systemic Inflammatory Response Syndrome is a Major Contributor to COVID-19-Associated Coagulopathy: Insights from a Prospective Single-Center Cohort Study. DOI: 10.1161/CIRCULATIONAHA.120.048925
161. Tang N., Li D., Wang X., Sun Z. Abnormal coagulation parameters are associated with poor prognosis in patients with novel coronavirus pneumonia. J Thromb Haemost 2020; 18:844–47.
162. Thachil J., Wada H., Gando S., et al. ISTH interim guidance on recognition and management of coagulopathy in COVID-19. J Thromb Haemost 2020; DOI:10.1111/jth.14810.
163. Spyropoulos AC, Levy JH, Ageno W, et al. Scientific and Standardization Committee Communication: Clinical Guidance on the Diagnosis, Prevention and Treatment of Venous Thromboembolism in Hospitalized Patients with COVID-19. J Thromb Haemost 2020; DOI: 10.1111/jth.14929.
164. Moores LK, Tritschler T, Brosnahan S, et al. Prevention, Diagnosis, and Treatment of VTE in Patients With COVID-19. CHEST Guideline and Expert Panel Report. Chest 2020; DOI: 10.1016/j.chest.2020.05.559.
165. COVID-19 Treatment Guidelines Panel. Coronavirus Disease 2019 (COVID-19) Treatment Guidelines. National Institutes of Health. Available at https://www.covid19treatmentguidelines.nih.gov/
166. Vashukova M.A., Tsinzerling V.A., Semenova N.Yu., Lugovskaya N.A., Narkevich T.A., Sukhanova Yu.V. Is perinatal COVID 19 possible: first results. Journal of Infectology.2020;3 (12):51- 55.
167. Donduray E.A., Isankina L.N., Afanasyeva O.I., Titeva A.V., Vishnevskaya T.V., Kondratyev V.A. et al. Characteristics of COVID-19 in children: first experience in a St. Petersburg hospital. Journal of Infectology.2020;3 (12):56-63.
168. Lobzin Yu.V., Cherkashina I.V., Samoilova I.G. Medical rehabilitation of children who have had COVID -19. Journal of Infectology.2020;3 (12):64-74.
169. Pshenisov K.V., Aleksandrovich Yu.S., Kaziakhmedov V.A., Kostik M.M., Kondrashov I.A. Novel coronavirus infection in children with concomitant diseases: there is always a chance for recovery (clinical cases) Journal of Infectology. 2020; 3 (12):80-89.
170. Uskov A.N., Lobzin Yu.V., Rychkova S.V., Babachenko I.V., Fedorov V.V., Ulukhanova L.U., Pochinyaeva L.M. The course of a new coronavirus infection in children: some aspects of monitoring and analysis of mortality. Journal of Infectology. 2020; 3 (12):12-21.
171. T.A. Ruzhentsova, P.V. Chuklyaev, D.A. Khavkina, A.A. et al. Possibilities of etiotropic therapy of coronavirus infection caused by SARS-CoV-2 in outpatients. Medical Opponent, No. 1 (9), 2020: pp. 48-58.
172. Beigel JH et al. ACTT-1 Study Group Members. Remdesivir for the Treatment of Covid-19 - Final Report. N Engl J Med. 2020 Oct 8: NEJMoa2007764. doi: 10.1056/NEJMoa2007764.
173. ESMO guidelines Cancer patients' management during the COVID-19 pandemic. https://www.esmo.org/guidelines/cancer-patient-management-during-the-covid-19-pandemic
174. Kuderer NM Chouein TK, Shah DP et al. Clinical impact of COVID-19 and patients with cancer (CCC19): A COHORT STUDY. Lncet 2020; S0140-6736(20)31187-9. Doi; 10.1016.S0140-6736(20)311197-9.

175. Poortmans HM, Gardoso MJ Cancer and COVID-19: what do we really know? www.thelancet.com.2020. https: //doi.org/10.1016/S0140-6736(20)31240-X.
176. Practical recommendations of the Russian Society of Clinical Oncology for the provision of oncological care in the context of the COVID-19 pandemic. https://rosoncoweb.ru/standarts/COVID- 19/
177. Peculiarities of management of oncohematological patients in the context of the COVID-19 pandemic / Edited by I.V. Poddubnaya. Moscow: Ekon-Inform, 2020.
178. E.A. Boryakh, O.L. Kochneva, E.N. Misyurina et al. Management of oncohematological patients with a new coronavirus infection: the experience of City Clinical Hospital No. 52. Modern oncology. 2020. Vol. 22. No. 2, pp. 28-32.
179. Kaprin A.D., Gameeva E.V., Polyakov A.A. et al. Impact of the COVID-19 pandemic on oncology practice. Siberian Journal of Oncology. 2020;19(3):5-22. https://doi.org/10.21294/1814-4861-2020-19-3-5-22 .
180. Bomshteyn N.G., Bolotov Yu.V., Kim I.A., D.V. Trukhin D.V. Efficiency of sorbed probiotics in the complex treatment of the new coronavirus infection COVID-19 in a hospital setting. Medicine of extreme situations. 2020; 4 (22):31–35. DOI: 10.4 183/mes.2020.021
181. Zakharchenko S.M., et al. Adverse drug reactions from the gastrointestinal tract and antibiotic-associated diarrhea during the use of antibiotics in outpatient practice: prevention and treatment. Clinical microbiology and antimicrobial chemotherapy, 2019; 3: 196-206.
182. Guarner F., ea World Gastroenterology Organization Global Guidelines. Probiotics and prebiotics. February 2017. Available at: http://www.worldgastroenterology.org/guidelines/global-guidelines/probiotics-and-prebiotics/probiotics-and-prebiotics-english.
183. Szajewska H., ea Probiotics for the Prevention of Antibiotic-Associated Diarrhea in Children. J Pediatr Gastroenterol Nutr. 2016; 62(3): 495-506.
184. Shen NT, ea Timely Use of Probiotics in Hospitalized Adults Prevents Clostridium difficile Infection: A Systematic Review With MetaRegression Analysis. Gastroenterology. 2017; 152(8): 1889-1900. doi: 10.1053/j.gastro.2017.02.003.
185. Zeltyn-Abramov E.M. et al. Stress-induced cardiomyopathy in a patient with fulminant COVID-19 early after kidney allotransplantation (Case study and brief literature review). Clinical Nephrology. 2020; (2): 10 15 DOI: https://dx.doi.org/10.18565/nephrology.2020.2.10-15 .
186. Frolova N.F. et al. Novel coronavirus infection COVID-19 in a patient after kidney allotransplantation. Clinical Nephrology. 2020; 2:16-20 DOI: https://dx.doi.org/10.18565/nephrology.2020.2.16-20 .
187. Zeltyn-Abramov E.M. et al. COVID19 - associated cardiac lesions in patients on program hemodialysis (Series of clinical observations and brief review of the literature). Nephrology and Dialysis. 2020; 22:9-20 DOI: 10.28996/2618-9801-2020-Special_Issue-21-32.
188. Bautin A.E., Avdeev S.N., Seyliev A.A., Shvechkova M.V., Merzhoeva Z.M., Trushenko N.V., Semenov A.P., Lapshin K.B., Rosenberg O.A. Inhalation therapy with surfactant in complex treatment severe form of COVID-19 pneumonia. Tuberculosis and Lung Diseases 2020; 98(No. 9): 6-12. http://doi.org/10.21292/2075-1230-2020-98-9-6-12 .
189. Averyanov A.V., Klypa T.V., Balionis O.I., Bychinin M.V., Chernyak A.V., Troitsky A.V. Inhalation surfactant in high-flow oxygen therapy in patients with COVID-19: results of a retrospective analysis. Remedium 2020; (7-8): doi: 10.21518/1561-5936-2020-7-8-84 88.
190. Piva S, DiBlasi RM, Slee AE, Jobe AH, Roccaro AM, Filippini M, Latronico N, Bertoni M, Marshall JC, Portman MA. Surfactant therapy for COVID-19 related ARDS: a retrospective case–control pilot study. Respir Res 2021; 22: 20. https://doi.org/10.1186/s12931-020-01603-w .
191. Busani S, Dall'Ara L, Tonelli R, et al. Surfactant replacement might help recovery of low-compliance lung in severe COVID-19 pneumonia. Ther Adv Respir Dis 2020, 14: 1–6 DOI: 10.1177/1753466620951043.
192. Heching M, Lev S, Shitenberg D, Dicker D, Kramer MR, Surfactant for Treatment of ARDS in COVID- 19 Patient. Chest 2021: doi: https://doi.org/10.1016/j.chest.2021.01.028 .
193. Zeltyn-Abramov E.M. et al. Risk factors for poor prognosis in patients on program hemodialysis with COVID-19. Focus on cardiovascular comorbidity (Single center experience). Nephrology and Dialysis. 2020. 22:21-32. DOI: 10.28996/2618-9801-2020-Special_Issue-21-32.
194. Stolyarevich E.S., Frolova N.F., Artyukhina L.Yu., Varyasin V.V. Kidney damage in COVID-19: clinical and morphological manifestations of renal pathology in patients who died from COVID-19. Nephrology and Dialysis. 2020; 22: 46-55. DOI: 10.28996/2618-9801-2020-Special_Issue- 46-55
195. Frolova N.F. et al. Eculizumab in the treatment of ANCA-associated vasculitis complicated by COVID-19. Clinical observations. Nephrology and Dialysis. 2020; 22:33-45 DOI: 10.28996/ 2618-9801-2020-Special_Issue-33-45

196. Gauthier S.V. et al. Features of the clinical course of coronavirus infection COVID-19 in recipients of the heart, kidney, liver: the first results of the national multicenter observational study "ROCCOR-recipient". Bulletin of Transplantology and Artificial Organs. 2020; 22(3):8-17. https://doi.org/10.15825/1995-1191-2020-3-8-17
197. Tuccori M, Ferraro S, Convertino I, Cappello E, Valdiserra G, Blandizzi C, Maggi F, Focosi D. Anti-SARS-CoV-2 neutralizing monoclonal antibodies: clinical pipeline. MAbs. 2020 Jan-Dec;12(1):1854149.
198. Copin R, et al. In vitro and in vivo preclinical studies predict REGEN-COV protection against the emergence of viral escape in humans. bioRxiv 2021.03.10.434834.
199. Fact sheet for health care providers emergency use authorization (EUA) of casirivimab and imdevimab. https://www.fda.gov/media/143892/download
200. Ramakrishnan S. et al. Inhaled budesonide in the treatment of early COVID-19 (STOIC): a phase 2, open label, randomized controlled trial. Lancet Respir Med. 2021 Apr 9:S2213-2600(21)00160-0.
201. Ly-Mee Yu et al. Inhaled budesonide for COVID-19 in people at higher risk of adverse outcomes in the community: interim analyzes from the PRINCIPLE trial. 2021 https://www.medrxiv.org/content/10.1101/2021.04.10.21254672v1
202. Parham Sadeghipour et al. Effect of Intermediate-Dose vs Standard-Dose Prophylactic Anticoagulation on Thrombotic Events, Extracorporeal Membrane Oxygenation Treatment, or Mortality Among Patients With COVID-19 Admitted to the Intensive Care Unit. The INSPIRATION Randomized Clinical Trial INSPIRATION Investigators *JAMA.* 2021;325(16):1620-1630. doi:10.1001/jama.2021.4152
203. Therapeutic Anticoagulation in Critically Ill Patients with Covid-19 – Preliminary Report The REMAP CAP, ACTIV-4a, and ATTACC Investigators doi:10.1101/2021.03.10.21252749

Appendix 1

A. Recommendations for the description of RG and CT data of the OGK

1. Radiography of the chest

Method of implementation

X-ray examination in the X-ray room is performed using a stationary (or mobile) X-ray machine in two standard projections: direct anterior and right lateral.

Radiography in the intensive care unit is performed using a mobile radiodiagnostic device in one standard projection: direct posterior when the patient is lying on his back or direct anterior when the patient is lying on his stomach. If necessary, a picture can be taken in the lateral projection with a horizontally directed beam of X-ray radiation (laterogram). The X-ray picture must contain standard markings and additional information (time of the X-ray in hours and minutes; patient's position at the time of the X-ray - on his back or on his stomach).

Other radiographic diagnostic techniques (fluoroscopy, linear tomography, etc.) are not used to evaluate patients with suspected/known COVID-19 pneumonia.

Protocol of the RG study

The protocol based on the results of the conducted WG is formed according to standard rules:

- Type of examination, projections, patient’s position at the time of RG.
- All medical devices and other objects visible in the images (endotracheal tube, catheters, drains, probes, artificial pacemakers, foreign bodies, etc.), their location and correct installation;
- Visible pathological changes.
- The presence of areas of lung tissue compaction, their location (peripheral, central, diffuse, increasing towards the diaphragm/apex). Local changes are related to individual lobes and/or segments. *Areas of lung tissue compaction in RG are usually not divided into ground glass and consolidation* .

- Shape of compacted areas: round, any other.
- Intensity of the shadow of consolidations in the lungs: low (usually corresponds to the "ground glass" symptom on CT), moderate intensity (usually corresponds to consolidation on CT), high (can be observed with total or subtotal lung damage) or a combination of these.
- Signs of pulmonary circulation disorder: increased (incl. redistribution of the pulmonary pattern to the upper lobes, expansion of the roots of the lungs, peribronchial cuffs, Kerley lines, expansion of the chambers of the heart, expansion of the vascular pedicle of the heart.
- Other signs of lung pathology: cavities, focal disseminations, local lobar and segmental compactions, etc.
- Pleural sinuses: signs of pleural effusion.
- Signs of pneumothorax and/or pneumomediastinum and/or pneumoperitoneum.

The conclusion must indicate the presence of pathological changes and their prevalence.

In the case of an initial examination, it is recommended to indicate the probability of the detected changes corresponding to COVID-19 pneumonia - **high/typical picture, average/indeterminate picture, low/atypical picture, normal** (to assess the degree of probability, you can use the table below for formulating CT conclusions). *For example, a probable radiographic picture of COVID-19 pneumonia; or a picture of increased pulmonary markings without characteristic radiographic signs of COVID-19 pneumonia; or a radiographic picture of pulmonary edema; or a radiographic picture of an abscess of the upper lobe of the right lung.*

During repeated studies, the dynamics of changes in the chest cavity must be indicated.

If necessary, additional studies are indicated that, in the opinion of the radiologist, can help in assessing the changes: CT, ultrasound, fibrobronchoscopy, etc.

2. Description of CT chest results

Method of implementation

The chest scan is performed according to a standard program established by the manufacturer, with the patient lying on his back, with his hands behind his head, and, if possible, with a calm, held breath.

When examining patients on artificial ventilation, breath holding will occur with a short cessation of respiratory movements. CT scanning of patients on artificial ventilation is possible only if technical conditions are met and the patient can be delivered to the office or on a mobile device.

Intravenous contrast is not required, but can be used if other pathological conditions are suspected, such as pulmonary embolism, as well as oncological diseases, etc.

The CT protocol is formed according to standard rules similar to those for X-ray examination. However, the terminology adopted for describing CT data is used.

In order to speed up the receipt of the most important CT chest results for doctors and to determine the tactics of patient management, it is possible to use preliminary standardized express protocol forms, and the conclusion itself is issued within the established timeframes.

A sample express form is provided below; it can be modified or corrected according to the needs of a specific medical organization.

Recommended Express Form
for Reporting Chest CT Scan Results in Patients with Suspected COVID Pneumonia

Date and time of the study		
Passport details		
Registration data		
Type of research	CT scan of the chest without intravenous contrast	
Another study		
**Changes in lung tissue (ground glass ± consolidation)**		
Left lung		Yes/No
Right lung		Yes/No
Approximate volume of detected lung changes (visually)	KT-1 Minimum	< 25% of volume
	KT-2 Medium	25 – 50% of volume
	CT-3 Significant	50 – 75% of the volume
	CT-4 Subtotal	
Other important findings (if any)		
**Fluid in the pleural cavity**		
On the right		Yes/No
Left		Yes/No
Probability of association of changes detected in the lungs with viral (in particular, COVID-19) pneumonia according to the ACR/RSNA/ESR-ESTI recommendations	Typical picture / High probability	
	An uncertain picture	
	Other diagnosis	
	Normal / No change	

3. General recommendations for reporting lung CT findings in patients with suspected COVID-19 pneumonia

For the correct interpretation of the data obtained, **before the description begins,** it is extremely important for the attending physician to obtain clinical information about the patient's medical history and condition (preferably remotely, without direct contact).

The CT scan protocol must indicate:

- Date of onset of disease symptoms on the day of CT scan (if known);

- Scanning technique, use of intravenous contrast;
- Medical devices and other objects in the scanning area (endotracheal tube, catheters, drains, probes, artificial pacemakers, foreign bodies, etc.), their location and correct installation;
- The presence of changes in the lungs such as “ground glass” opacity, consolidation, “cobblestone” symptoms, “air bronchography”, and reverse “halo”;
- Localization of changes in the lobes and segments of the lungs, as well as the predominant spatial distribution (peripheral, central, diffuse; predominantly posterior or anterior, upper or lower);
- The presence and approximate volume of fluid in the pleural cavity and/or pericardium;
- All other findings are indicated (according to the standard protocol for describing chest CT): findings in the lung tissue (foci, cavities, the “tree in buds” symptom, and others), the condition and course of the trachea and bronchi, the condition of individual groups of lymph nodes, the size and contours of the main vessels and chambers of the heart; the condition of the spine and other bones within the scanning area;
- A description of the dynamics is mandatory if there are data from previous CT scans;
- The **conclusion** provides a probabilistic assessment of the relationship between the identified changes and COVID-19 in accordance with international recommendations and an approximate volume of lung damage (see below).

It should always be remembered that the study can be performed on a patient with any pathology of the lungs, heart, mediastinum and other organs!

The principles of assessing lung tissue changes in COVID-19 based on chest CT data are presented in the table. The percentage of damage is assessed separately for each lung. The degree of changes is assessed for the lung with the greatest damage (regardless of the presence of postoperative changes).

Recommendations for the formulation of the CT report: the probability of association of the identified changes with COVID-19 pneumonia (RSNA/ACR/BSTI/ESR-ESTI recommendations)

**Signs of pathology in CT**	**Possible wording in the conclusion:**
**Typical picture**<br>• Numerous bilateral subpleural consolidation of lung tissue of the "ground glass" type,<br>o including with consolidation and/or with the "cobblestone" symptom;<br>• Numerous bilateral rounded areas of ground glass opacity deep within the lung tissue, including in combination with consolidation and/or cobblestone pavement;<br>• Areas of compaction of lung tissue in the form of combinations of "ground glass" and consolidation with the "reverse halo" symptom as signs of organizing pneumonia.	**COVID -19 pneumonia ,** given the clinical picture, there are typical CT signs of the disease.<br><br>Similar changes may occur in other viral pneumonias, as well as in connective tissue diseases, be associated with the toxic effects of drugs, or have a different etiology.
**Vague picture -** absence of a typical picture and<br>• Frosted glass areas predominantly root localization;<br>• Small areas of "frosted glass" without typical (peripheral) distribution, not rounded;<br>• One-sided "frosted glass" areas in within one share, with or without consolidation.	**Moderate (uncertain) probability of COVID -19 pneumonia**<br>The identified changes may be a manifestation of COVID-19 pneumonia, but they are nonspecific and may occur in other lung diseases (indicate which ones, if possible; for example, heart failure, bacterial pneumonia, etc.).<br><br>CT results should be interpreted with caution in patients with chronic concomitant diseases that increase the risk of changes in the chest cavity (coronary heart disease, cancer, kidney disease, etc.)
**Atypical picture -** absence of typical and indefinite picture and<br>• Consolidation of share (segment);<br>• Foci (including the "tree in kidneys");<br>• Volumetric formations;<br>• Cavities in the lungs and in areas consolidation;<br>• Uniform thickening of the interlobular partitions with fluid in the pleural cavities (picture of pulmonary edema);<br>• Subpleural reticular (net) changes;	**Alternative diagnosis**<br>The changes identified are not characteristic of COVID-19 pneumonia. The possibility of other diseases and pathological conditions should be considered (specify which ones, if possible; for example, tuberculosis, lung cancer, bacterial pneumonia, etc.).
• Lymphadenopathy without changes in the lungs.	

**Normal picture**	No signs of pneumonia or other pathological changes*

*It should be borne in mind that in the initial stages of the disease (1-5 days) CT results may be negative. A normal CT picture does not exclude COVID-19 and is not a limitation in conducting immunological (PCR) tests

4. Evaluation of the severity of changes in the lungs using CT

Assessment of the severity (volume, area, extent) of changes in the lungs in patients with suspected/known COVID-19 pneumonia can be carried out in several ways:

- By visual assessment;
- By using semi-quantitative scales proposed by a number of authors;
- Based on computer programs for assessing lung density and creating lung parenchyma density maps.

In conditions of a large flow of patients, for a rapid assessment of changes in the lungs after RG and CT, the so-called "empirical" visual scale is recommended. It is based on a visual assessment of the approximate volume of compacted lung tissue in the lung with the greatest damage.

Adapted "empirical" visual scale[XVIII]

Based on a visual assessment of the approximate volume of compacted lung tissue in the lung with the greatest damage:

1. Absence of characteristic manifestations (CT-0);
2. Minimum volume/extent <25% of lung volume (CT-1);
3. Average volume/prevalence 25 – 50% of lung volume (CT-2);
4. Significant volume/prevalence 50 – 75% of lung volume (CT-3);
5. Subtotal volume/extent >75% of lung volume (CT-4) .

In the first months of the pandemic, semi-quantitative scales were used to assess the severity of lesions with separate calculation of the volume and type of lesions for lobes and segments of the lungs, followed by summing up the results. With the increasing workload on medical organizations, the use of semi-quantitative scales is difficult and ineffective in terms of labor productivity. A visual assessment was proposed based on determining the approximate total volume of compacted lung tissue in the lung with the greatest lesion. The adapted scale was validated by proving the presence of a statistically significant trend in the directional change in the proportion of deceased patients among various CT0-CT4 categories [XIX].

Computer-assisted diagnostics

A number of workstations for modern CT and digital X-ray machines are equipped with computer programs that are designed for automatic or semi-automatic selection and measurement of the volume of "ground glass" and consolidation zones. The use of such programs allows for a more objective assessment of the volume of altered lung tissue than the "empirical" method, especially when observing dynamically. When using such computer programs, the radiologist must monitor the correctness of the selection of pathological areas in the lungs and decide whether the data obtained with their help are suitable for clinical use or not.

In general, visualization data can only be interpreted in the context of the clinical picture and the results of laboratory and instrumental parameters. Radiography and CT play an important, but not decisive role in assessing the severity of the disease and the prognosis of its development.

[1] Original scale - Inui S, Fujikawa A, Jitsu M et al. Chest CT Findings in Cases from the Cruise Ship Diamond Princess with Coronavirus Disease (COVID-19). Radiol Cardiothorac Imaging. 2020 Mar 17;2(2):e200110. doi: 10.1148/ryct.2020200110.
XIXRadiation diagnostics of coronavirus disease (COVID-19): organization, methodology, interpretation of results: guidelines / compiled by S. P. Morozov, D. N. Protsenko, S. V. Smetanina [et al.] // Series "Best practices in radiation and instrumental diagnostics". - Issue 93. -M .: State Budgetary Healthcare Institution "Research and Clinical Center for Diagnostics and Toxicology of the Moscow Department of Health", 2020. - 102 p.

These limitations are associated, among other things, with the lack of characteristics of lung tissue damage (ground glass and/or consolidation) and other manifestations of pathology (pleural effusion, pulmonary edema, etc.). Therefore, **the volume of lung damage in X-ray and CT may not have a direct correlation with the clinical severity of the disease** .

5. Evaluation of the dynamics of changes in the lungs in COVID-19 pneumonia

The main patterns of development of COVID-19 pneumonia are associated with the transformation of "ground glass" areas and consolidation in the lung tissue. In this regard, CT is the preferred method for assessing the dynamics. However, RG can also be used for this purpose if the changes are visible on the images and it is possible to assess their dynamics. Radiography using a mobile device is also the main method for monitoring patients in the intensive care unit.

Evaluation of dynamics involves the use of one method. Comparison of changes according to data from different methods and techniques (for example, RG and CT) is not correct.

All X-ray examinations are performed only for clinical indications.

The frequency of dynamic X-ray and CT studies is determined by the attending physician (ICU physician).

Table 1. Dynamics of changes in the lungs according to X-ray and CT data

Dynamics of the process	Signs
**Initial manifestations in the first days of the disease**	**Typical picture**<br>• Subpleural areas of ground glass opacities<br>• with or without consolidation, with or without thickening of the septa (cobblestone symptom);<br>• Areas of compaction of the "frosted glass" type, rounded in shape peribronchial location, with or without consolidation, with or without thickening of the septa (the "cobblestone" symptom);<br>• Combination of frosted glass areas and consolidation with "reverse halo" symptom and other signs organizing pneumonia;<br>• The location of the changes is bilateral, predominantly peripheral.
**Positive dynamics of changes (stabilization)**	• Transformation of "frosted glass" areas into seals consolidation type (increase in density of altered areas of lung tissue) without visible increase in volume (extent) of lung damage;<br>• Formation of a picture of organizing pneumonia;<br>• Reduction in the size of compacted areas of lung tissue.
**Negative dynamics of changes (progression)**	**Accumulation of changes** :<br>• Increasing the size (length, volume) of the existing areas of compaction of the "frosted glass" type;<br>• The appearance of new areas of "frosted glass";<br>• Merging individual areas of "frosted glass" into larger ones compaction up to subtotal lung damage;<br>• The severity of the "ground glass" areas remains significantly prevails over consolidation.<br>**The appearance of new signs** of other pathological processes:<br>• Left ventricular failure (hydrostatic cardiogenic pulmonary edema, bilateral pleural effusion),<br>• Respiratory distress syndrome (pulmonary edema);<br>• Bacterial pneumonia (new local areas) consolidation, pleural effusion);<br>• Lung abscess and multiple septic emboli;<br>• Pneumothorax and pneumomediastinum;<br>• Thromboembolism of small branches of the pulmonary artery (including decreased saturation against the background of a stable radiological picture - CT of the chest with contrast may be required);<br>• Other.
**Picture of ARDS**	**Typically characterized by:**<br>• Bilateral subtotal compaction of lung tissue by type consolidation and "frosted glass";<br>• Located in the middle and upper parts of the lungs;<br>• Swelling of the basal segments;<br>• The gradient of seals depending on the patient's position (on back, on the stomach);<br>• Air bronchography symptom;<br>• Increase in lesion volume by 50% in 24–48 hours against the background of respiratory disorders;

	• Fluid in the pleural cavities (hydrothorax).<br>**Usually not characteristic** (in the absence of circulatory failure):<br>• Kerley lines, peribronchial couplings;<br>• Enlargement of the left chambers of the heart, vascular pedicle of the heart.
**Permission**	• Reducing the size of consolidation areas and "matte" glass" (pictures of organizing pneumonia);<br>• The duration of the changes in the lungs can significantly exceed the time of clinical manifestations infections;<br>• The presence of residual compactions in the lung tissue does not affect duration of therapy for an infectious disease and is not an indication for its continuation in the absence of clinical manifestations of an acute inflammatory process.<br>• New "frosted glass" zones of no more than 25% are permitted transverse size of the hemithorax.

B. Illustrations of the probabilistic classification of lung changes in COVID-19 pneumonia based on CT (RG) data

All detected changes should be interpreted taking into account the epidemiological situation (COVID-19 epidemic) and clinical and laboratory data. A normal and/or almost normal picture in CT, and especially RG, can be observed in patients with COVID-19 at the initial stage of the disease (1-5 days of the disease, before the development of pneumonia) or in asymptomatic carriers of the virus, who can be a source of infection for other people.

B.1 CHEST RADIOGRAPHY

Typical picture. High probability of COVID pneumonia

1. Множественные периферические затемнения в нижних долях легких, на их фоне имеются округлые тени

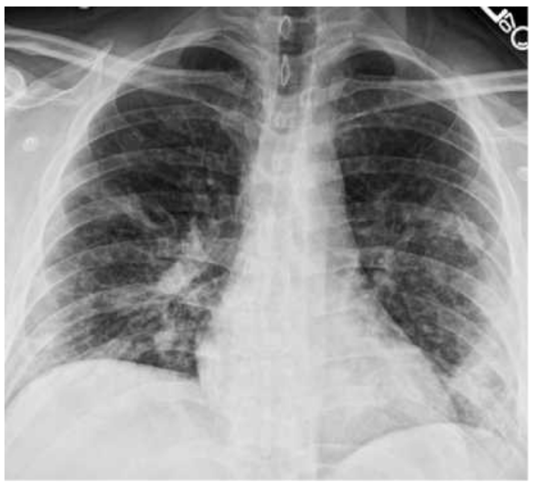

2. Multiple peripheral rounded opacities in the lower lobes of the lungs, merging with each other

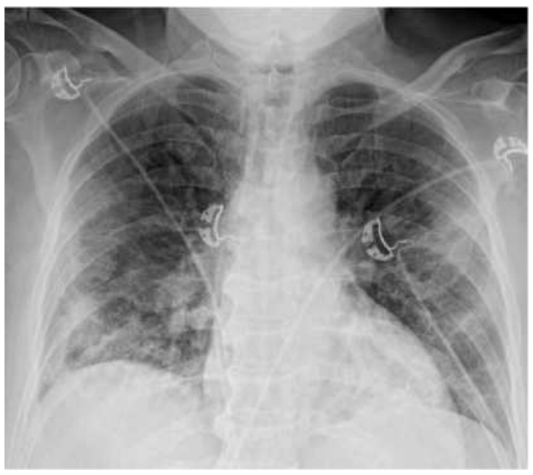

An uncertain picture.
Average Probability of COVID-19 Pneumonia

Диффузные затемнения в легочных полях, равномерно распределенные по легким без преимущественной периферической локализации

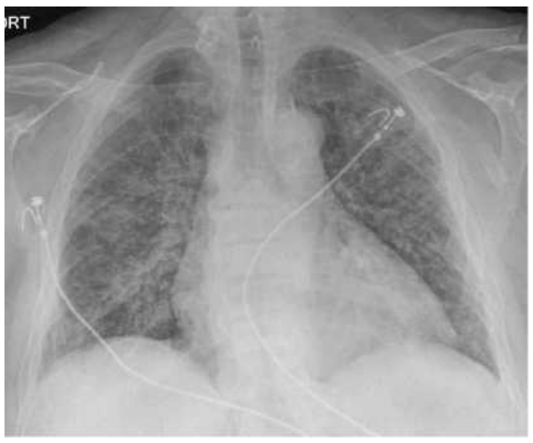

An atypical picture.
Low probability of COVID-19 pneumonia

Increased pulmonary pattern on both sides, increased density of bronchial walls

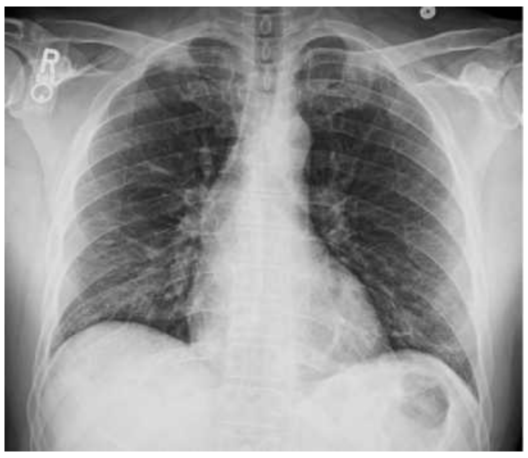

Source: D. Geffen, UCLA Health

B.2 COMPUTER TOMOGRAPHY OF THE CHEST

Typical picture. High probability of COVID-19 pneumonia

Numerous bilateral subpleural pulmonary consolidations with a ground-glass appearance

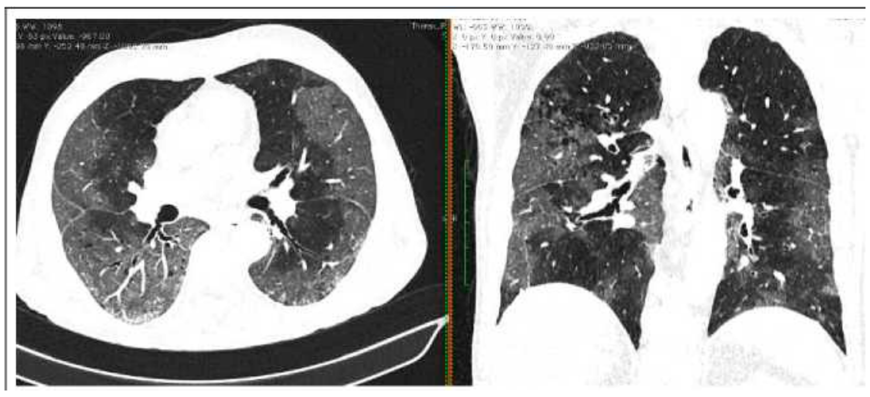

Areas of compaction of the "ground glass" type, rounded in shape, peripheral and peribronchial location, with consolidation

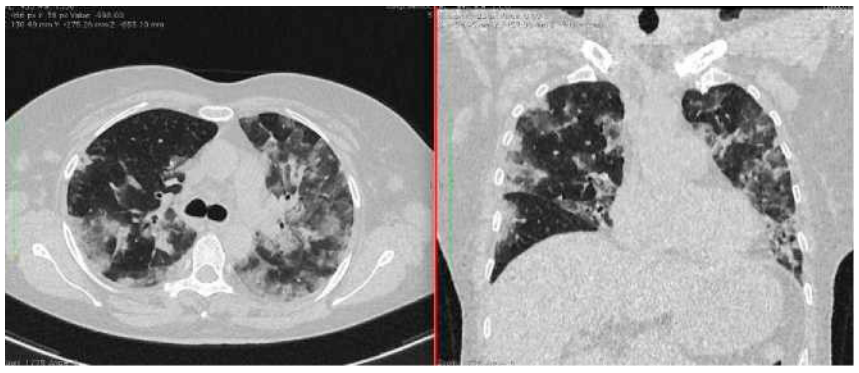

Multiple rounded areas of ground glass opacity in a peripheral and peribronchial location

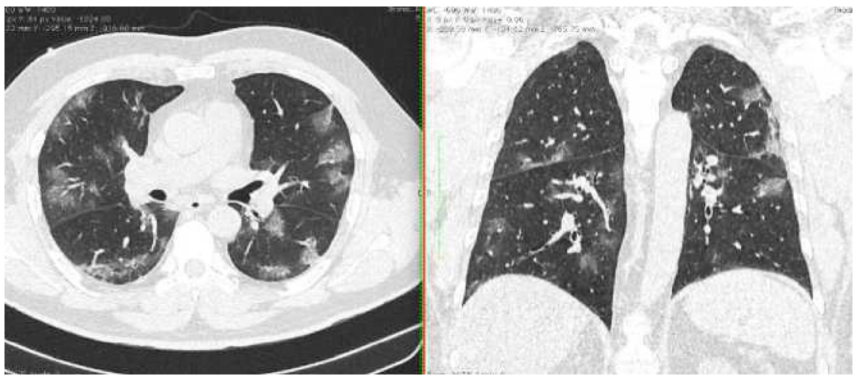

Bilateral, predominantly peripheral, subpleural consolidations of the lung tissue of the "ground glass" type with thickening of the septa (the "cobblestone pavement" symptom)

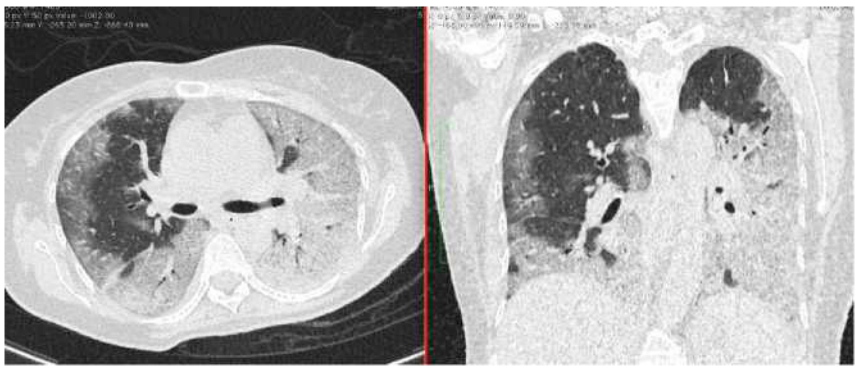

Uncertain picture. Average probability of COVID-19 pneumonia

Small areas of "frosted glass" without typical (peripheral) distribution, chaotic located inside the lungs

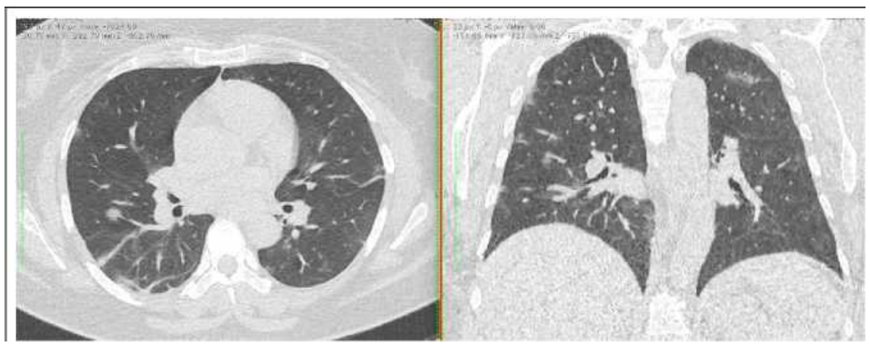

Single small areas of ground glass, without peripheral localization

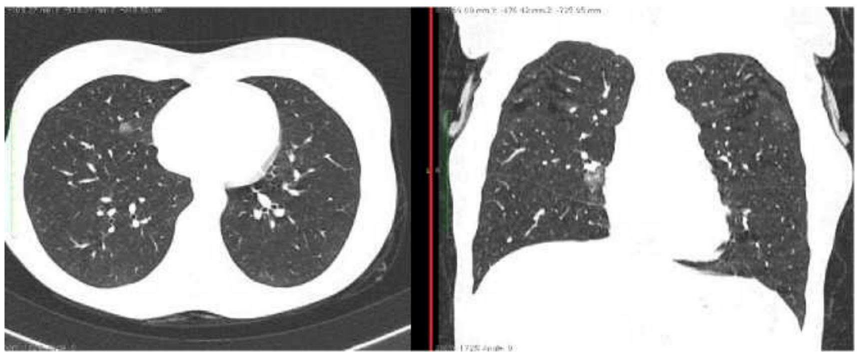

Atypical picture. Low probability of COVID-19 pneumonia

"Tree in buds" symptom with peribronchial infiltration in one lobe (bronchopneumonia, bronchiolitis)

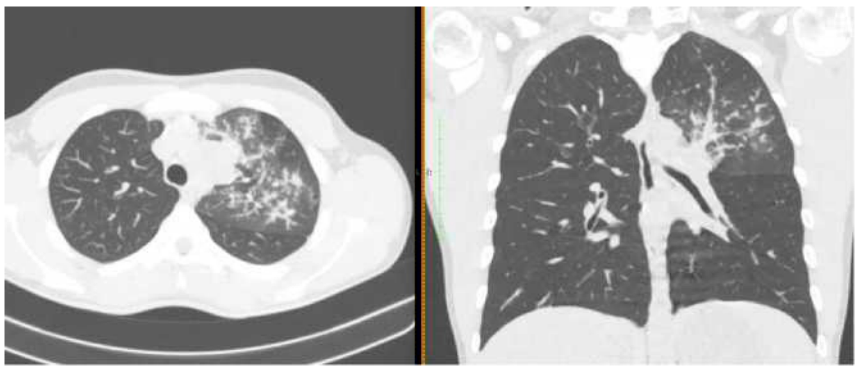

Severe infiltration and consolidation within one lobe (picture of lobar bacterial pneumonia)

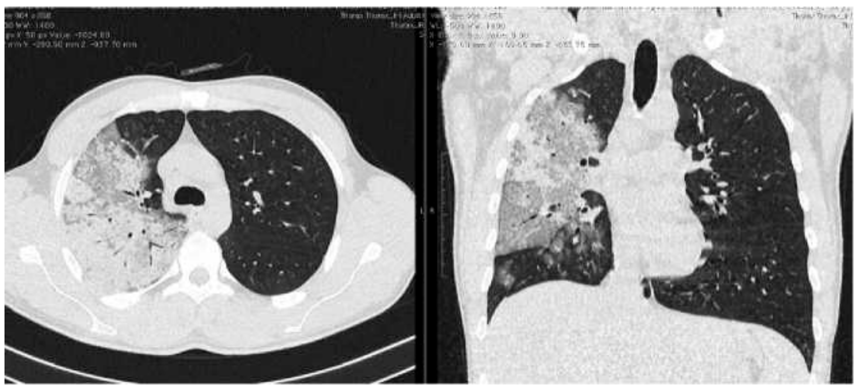

Symmetrical central foci of increased density of the “butterfly wing” type, hydrothorax – CT picture of alveolar pulmonary edema

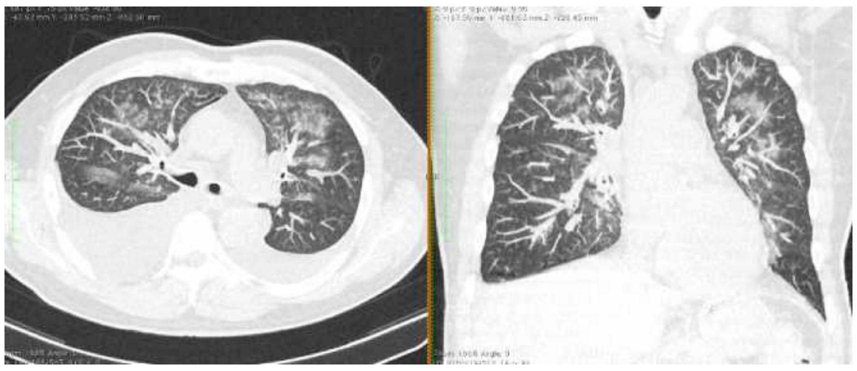

B.3 MAIN CT SYMPTOMS OF LUNG PATHOLOGY IN VIRAL LUNG INJURIES

Frosted glass

An area of partially air-filled lung tissue, against the background of which vessels, bronchial lumens and their walls are visible.
It is detected significantly more accurately by CT than by X-ray.

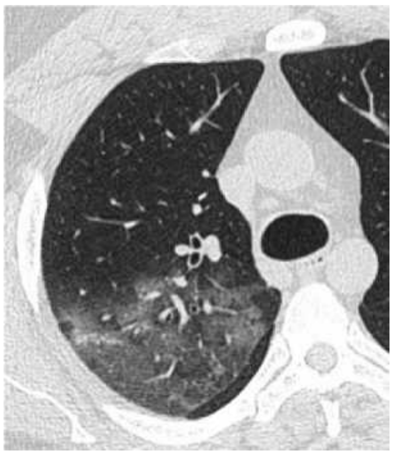

Consolidation

An area of airless lung tissue with visible air spaces of the bronchi and air cavities (e.g. emphysema). The vessels and walls of the bronchi in the compacted area are not visible.
It is detected equally accurately by X-ray and CT.

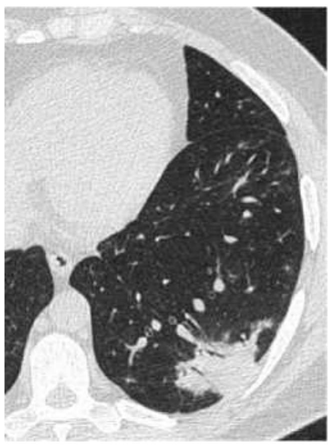

Reticular changes

Fine lines of pathologically altered pulmonary interstitium forming a network.
CT symptom. In RG it is designated as a reticular (cellular) deformation of the pulmonary pattern.

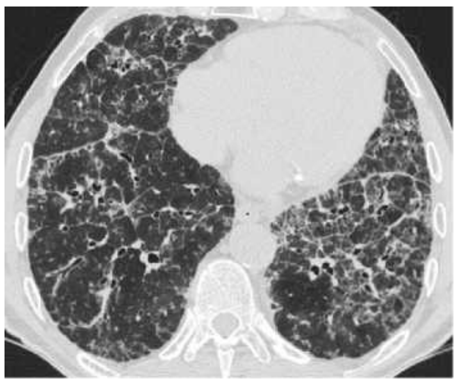

The "cobblestone" symptom

Synonym: patchwork quilt symptom. Image of reticular changes against the background of ground glass opacity. CT symptom.

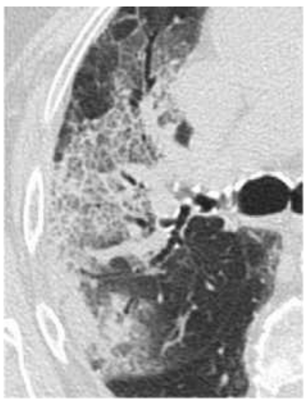

<u>Peribronchovascular distribution</u>

The location of altered areas of lung tissue along the bronchi and vessels of the lung.
It is detected equally by X-ray and CT.

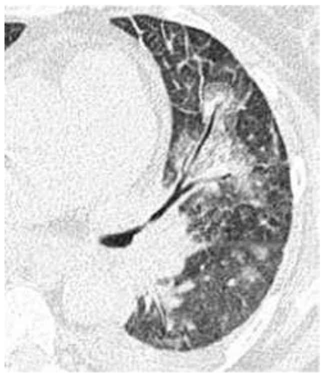

Cortical (subpleural, peripheral) **distribution**

The location of altered areas of lung tissue along the visceral pleura (costal, diaphragmatic, mediastinal, interlobar).
It is detected equally by X-ray and CT.

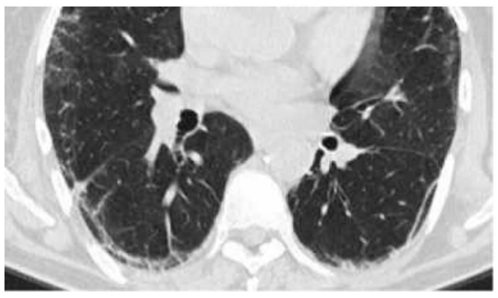

Perilobular changes

Compaction of lung tissue along the interlobular septa, one of the important signs of organizing pneumonia. CT symptom.

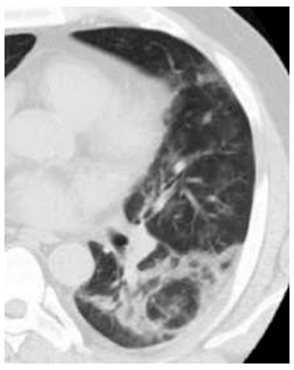

Root (central) **distribution**

The location of altered areas of lung tissue in the region of the root of the lung.
It is detected equally by X-ray and CT.

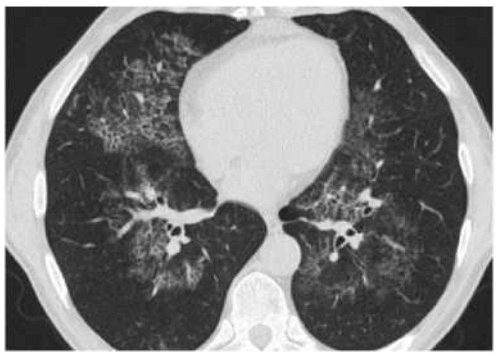

Air bronchography symptom

Visibility of air-filled bronchi in compacted lung tissue.
Indicates the preservation of bronchial patency. CT symptom.
Synonym: rim symptom.

Симптом ореола

The "ground glass" zone around the consolidation zone or the area of destruction (necrosis). Usually has a ring-shaped form.
CT symptom.

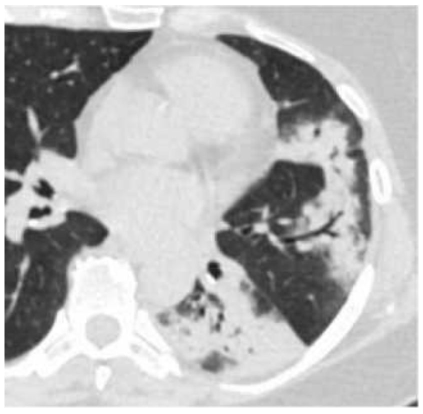

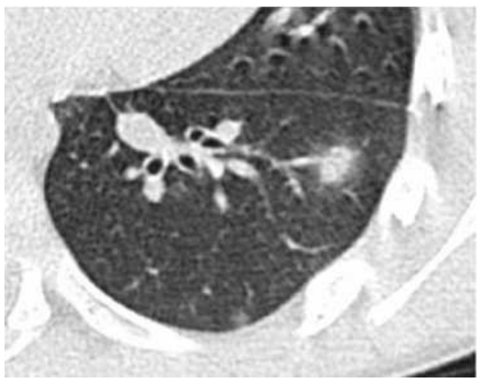

Reverse halo symptom

Synonym: inverse rim symptom, "atoll" symptom.
Consolidation zone around the "ground glass" area. Can be of any shape and size.
Characteristic sign of organizing pneumonia.
CT symptom.

Cavity in the lung or

consolidation area

A closed pathological space in the lung with thick (>2-3 mm) walls, surrounded by air-filled lung tissue. The cavity contains gas, liquid, and necrotic masses).
Usually observed in bacterial infections and neoplasms.
It is more accurately detected by CT, especially if the size is small.

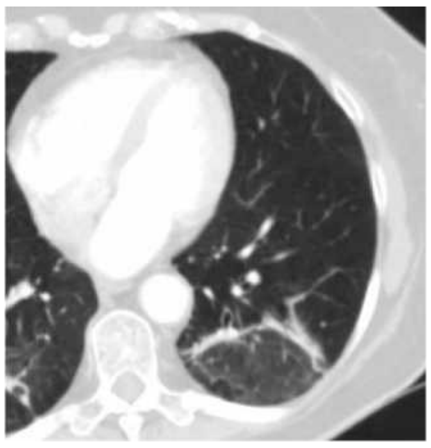

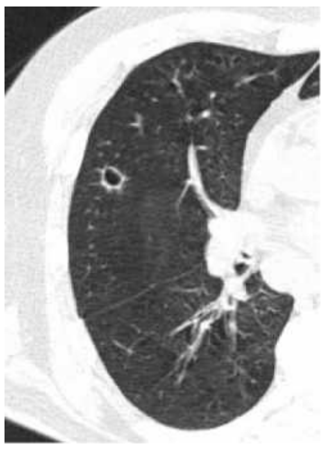

Cyst in the lung

A closed pathological space in the lung with thin (< 2 mm) walls, filled with gas or liquid.
CT symptom.

Focus(es) in the lungs

A compaction in the lung tissue up to 10 mm in size. They can be single, isolated (up to 6) and multiple (dissemination). More precisely, it is detected by CT (The term “nodule” is a synonym, but is not recommended for use).

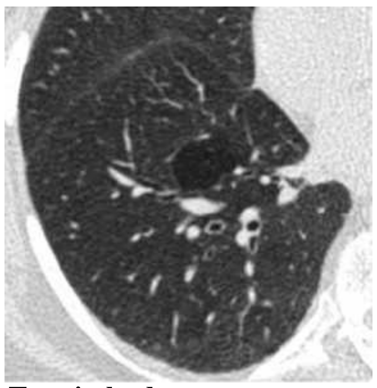

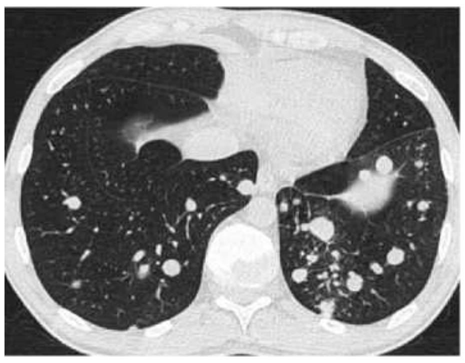

Tree in buds symptom

V- and Y-shaped pathological structures in the lung up to 1 cm in size, representing small bronchi and bronchioles filled with pathological contents and dilated. An important sign of bronchogenic infection of the lower respiratory tract.
CT symptom.

Picture of organizing pneumonia

Variable. Usually a combination of areas of ground glass opacity and consolidation with a reverse rim sign and a typical peribronchovascular and/or subpleural distribution.
A set of CT symptoms.

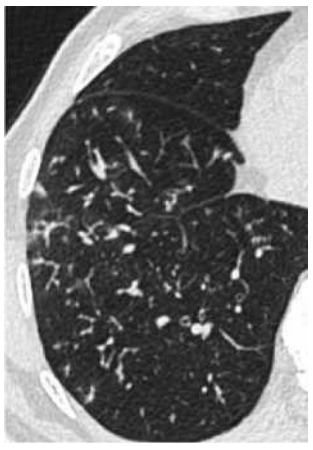

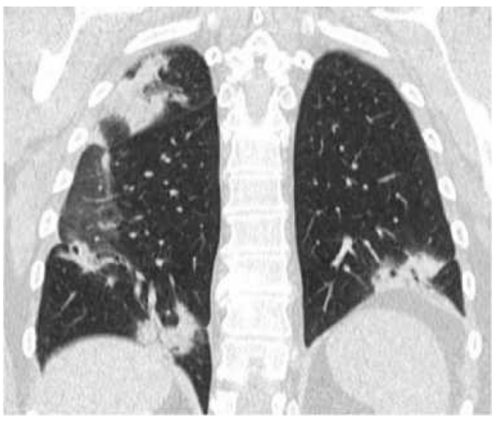

B. Recommendations for ultrasound in the context of the COVID-19 pandemic

1) Ultrasound of the lungs in patients with suspected or confirmed COVID-19 can be performed on any ultrasound machines, subject to mandatory compliance with all safety rules for personnel and disinfection of premises and equipment. It is preferable to allocate one or more ultrasound machines for working with patients with suspected or confirmed COVID-19. Depending on the contingent of subjects and the technical equipment of the institution, convex (preferably for adults), linear (preferably for newborns and young children), sector phased and microconvex sensors can be used.

2) The use of standardized lung ultrasound technology is recommended.

3) To simplify the interpretation of data and facilitate dynamic monitoring, it is

recommended to evaluate ultrasound changes in the lungs by gradation.

4) **There is insufficient scientific evidence to support the use of lung ultrasound in triage of patients with suspected or confirmed COVID-19.**

5) **A lung ultrasound cannot diagnose COVID-19.**

6) **A lung ultrasound cannot rule out a diagnosis of COVID-19.**

7) **There is insufficient scientific data to assess the severity of lung damage using ultrasound. The results of ultrasound of the lungs should be compared with the clinical and laboratory picture and CT data. They have no independent significance in COVID-19 .**

8) Lung ultrasound cannot replace chest X-ray and chest CT *in diagnosing pneumonia* in patients with suspected or confirmed COVID-19, but with an increase in the flow of patients, it can be included in a rational algorithm for diagnosing pneumonia for certain groups of patients (for example, pregnant women, newborns), ***provided that there are trained medical personnel*** .

9) Lung ultrasound cannot replace chest X-ray and chest CT *in the dynamic monitoring of patients with COVID-19 pneumonia* , but with an increase in the flow of patients, it can be included in a rational algorithm for dynamic monitoring, ***provided that there is (1) initial information about the true volume and cause of lung damage and (2) trained medical personnel*** .

10) It is necessary to comply with the safety rules for personnel and disinfection of premises and equipment, regardless of the reason for performing an ultrasound scan on patients with a suspected or confirmed diagnosis of COVID-19.

11) If there are clinical indications, ultrasound diagnostics can be used to assess the condition of other anatomical areas and individual organs in order to identify pathological changes and assess their dynamics, however, the principle of reasonable sufficiency should be a priority. Ultrasound examinations are mandatory for emergency indications. Examinations that are not necessary should be postponed or cancelled in order to minimize the possibility of healthy patients coming into contact with patients at risk for COVID-19 in a healthcare facility. If it is impossible to cancel scheduled ultrasound examinations, in order to reduce the risk of COVID-19 infection , it is important to: (1) adhere to the appointment schedule while maintaining the required time for each patient; (2) increase the intervals between patients to avoid crowding of waiting patients; (3) ensure a distance of at least 2 m between waiting chairs.

More detailed information on ultrasound examination of the lungs and the safety of ultrasound examinations in the context of COVID-19 is available on the website *www .rasudm .org* in the following documents:

1. Abramowicz JS, Basseal J. WFUMB Position Statement : How to Safely Perform Ultrasound and Disinfect Ultrasound Equipment in the Context of COVID-19 // Ultrasound and Functional Diagnostics. 2020. No. 1. pp. 12–23. Doi: 10.24835/1607-0771-2020-1-12 23. Published ahead of print. Access mode: // http://www.rasudm.org/files/WFUMB-Position-Statement-COVID.pdf , free. Title. from screen. 16.04.2020.

2. Mitkov V.V., Safonov D.V., Mitkova M.D., Alekhine M.N., Katrich A.N., Kabin Yu.V., Vetsheva N.N., Khudorozhkova E.D., Lakhin R.E., Kadrev A.V., Doroshenko D.A., Grenkova T.A. Consensus statement of RASUDM on ultrasound examination of the lungs in the context of the COVID-19 pandemic (version 2) // Ultrasound and functional diagnostics. 2020. No. 1. P. 46–77. Doi: 10.24835/1607-0771-2020-1 46-77. Published ahead of print. Access mode: // http://www.rasudm.org/files/RASUDM-Consensus-Statement-COVID-2.pdf , free. Title. from the screen. 04/30/2020.

Appendix 2

Laboratory monitoring of patients with COVID-19 or suspected of having COVID-19 depending on the severity of the condition

Mild to moderate course Outpatient treatment	Moderate severity Hospitalization	Severe course of intensive care
Clinical blood test	Clinical blood test no later than 12 hours after admission x 1 time in 2-3 days	Clinical blood test no later than 3 hours after admission, daily and as indicated
Biochemical studies as indicated	Biochemical tests (mandatory list*) x 1 time in 2-3 days	Mandatory biochemical tests*, electrolytes, albumin, lactate – daily and as indicated
	Coagulogram (APTT, prothrombin time, fibrinogen) x 1 time in 2-3 days<br>D-dimer as indicated	Hemostasis monitoring: D-dimer upon admission to the intensive care unit, then as indicated.<br>Coagulogram (APTT, prothrombin time, fibrinogen) upon admission, then as indicated, but not less than once every 2-3 days
	CRP no later than 24 hours after admission, then at least 2 times a week; procalcitonin and ferritin as indicated;<br>IL-6 if available	CRP at least once every 2 days, procalcitonin as indicated in case of suspected bacterial infection and sepsis;<br>ferritin, troponin upon admission and over time as indicated;<br>IL-6, NT-proBNP/BNP, T and B lymphocytes when available

* List of mandatory biochemical tests: urea, creatinine, glucose, alanine aminotransferase, aspartate aminotransferase, bilirubin, lactate dehydrogenase

Instructions for diagnostics of COVID-19 using nucleic acid amplification methods

General Provisions

In accordance with the order of the Ministry of Health of the Russian Federation No. 198n and METHODOLOGICAL recommendations MP3.1.0169-20 " LABORATORY diagnostics of COVID-19", approved by the Chief Sanitary Doctor of the Russian Federation on March 30, 2020, diagnostics of the new coronavirus infection is carried out in all laboratories of the Russian Federation, regardless of their organizational and legal form (hereinafter referred to as the Laboratory), that have a sanitary and epidemiological conclusion on the possibility of carrying out work with pathogens of human infectious diseases of III - IV pathogenicity and conditions for research using MAAT.

To detect the SARS-CoV-2 pathogen , NAAT (without accumulation of the pathogen) are used, using test systems registered in the established manner in the territory of the Russian Federation, in accordance with the instructions for their use.

Etiological diagnostics of COVID-19 is carried out using NAAT: polymerase chain reaction (PCR) and isothermal amplification (LAMP) .

The main type of biomaterial for laboratory testing for SARS-CoV-2 RNA is the material obtained by taking a swab from the nasopharynx (from both nasal passages) and oropharynx. Swabs from the mucous membrane of the nasopharynx and oropharynx are collected in one test tube for a higher concentration of the virus. In case of signs of a lower respiratory tract disease, in the event of a negative result in smears from the mucous membrane of the nasopharynx and oropharynx, sputum (if available) or bronchial washings obtained during fibrobronchoscopy (bronchoalveolar lavage), (endo)tracheal, nasopharyngeal aspirate are additionally examined. In patients on mechanical ventilation, it is recommended to obtain and examine an aspirate of the tracheal contents in order to detect SARS-CoV-2 . Biopsy or autopsy material of the lungs, whole blood, serum, and feces can be used as additional material for the study.

Collection, storage and transportation of diagnostic material

Collection of diagnostic material, its packaging, labeling and transportation are carried out in accordance with the requirements and rules for working with materials potentially infected with pathogens of pathogenicity group II , their storage and transportation in accordance with MU 1.3.2569-09 "Organization of the work of laboratories using MAAT when working with material containing microorganisms of pathogenicity groups I - IV " and Methodological recommendations of Rospotrebnadzor MP 3.1.0169-20 "Laboratory diagnostics of COVID-19".

The healthcare worker who collects, labels and packages the diagnostic material must be instructed in sanitary and epidemiological requirements and biological safety rules when working with patients potentially infected with pathogenicity group II microorganisms . Biological material (nasopharyngeal, oropharyngeal swabs, feces) can be collected independently by the patient according to the instructions. The healthcare worker must be provided with PPE: FFP2 respirators or their equivalent, or a pneumatic helmet providing a higher level of protection; glasses to protect the eyes or a protective

screen; anti-plague suit, disposable latex (rubber) gloves; waterproof apron.

A swab from the nasopharynx or oropharynx (pharynx) is taken with a sterile swab, which after taking the material is placed in a sterile plastic tube with a transport medium (taking into account the

recommendations of the manufacturer of the reagent kits used). To increase the concentration of the virus, nasopharyngeal and oropharyngeal swabs should be placed in one tube. The temperature during transportation should be +2° - +8 °C. The storage time of samples before testing should not exceed 5 days at +2° - +8 °C, it can be longer at -20 °C or -70 °C.

Sputum is collected in a disposable sterile plastic container with a capacity of 30-50 ml, tightly closed with a screw cap. The diameter of the container neck should be at least 30 mm. It is necessary to make sure that the collected material is sputum (secretions of the lower respiratory tract). Samples can be transported at a temperature of +2° - +8 °C. The storage time of samples before testing should not exceed 48 hours days at +2° - +8 °C. When stored in a transport medium containing antifungal and antibacterial drugs, more - at -20 °C or -70 °C.

Endotracheal aspirate, nasopharyngeal aspirate or nasal wash is collected in a sterile disposable container. Samples can be transported at a temperature of +2° to +8 °C. The storage time of samples before testing should not exceed 48 hours at +2° to +8 °C, more - at -20 °C or -70 °C.

Bronchoalveolar lavage is collected in a sterile disposable container. Samples can be transported at a temperature of +2°-+8 °C. The storage time of samples before testing should not exceed 48 hours at +2°-+8 °C, more - at -20 °C or -70 °C.

Biopsy or autopsy tissues, including lungs, are placed in disposable containers with a physiological solution containing antifungal and antibacterial drugs (collected in a sterile disposable container). Samples can be transported at a temperature of +2° to +8 °C. The storage time of samples before testing should not exceed 24 hours at +2° to +8 °C, more - at -20 °C or -70 °C.

To identify samples, containers/tubes are labelled at the collection point using self-adhesive labels containing information that ensures unambiguous identification of the sample and its appropriateness to the intended destination.

Transport of hermetically sealed containers with samples to the laboratory is carried out in special containers/boxes. Referrals and other documentation on paper media are transferred in a separate plastic bag.

If it is necessary to send samples to a laboratory of another medical institution, the requirements for sending infectious materials of pathogenicity group II are met (SP 1.2.036-95 "Procedure for recording, storing, transferring and transporting microorganisms of pathogenicity groups I-IV ").

The test tubes/containers with samples together with the lid are sealed with various plasticizers (paraffin, parafilm, etc.); the container is labeled. Each patient's samples are placed in an individual sealed package with an absorbent material and additionally packed in a common sealed package.

Two or more samples from the same patient may be packaged in one plastic bag. It is prohibited to package clinical samples from different people in the same package.

The package with containers is placed in a hermetically sealed container for transporting biological materials. The container is placed in a foam thermocontainer with cooling thermoelements. The transport container is sealed and marked. It is advisable to place a disposable indicator in the container to monitor compliance with the temperature from +2° to +8 °C.

Accompanying documents are placed in individual packaging separately from the biological material and are securely attached to the outside of the container.

Referral for research

A referral for laboratory testing is issued electronically (through the remote electronic registration system, or in the form of an electronic order in the MIS program by a clinician), or on paper.

A referral for laboratory testing must contain:

- personal data of the patient, ensuring his/her unique identification;
- name of the department (organization) that sent the biomaterial;
- preliminary diagnosis of the disease: "pneumonia" or "ARI" or “testing of contacts for SARS-CoV-2”;
- indication of the type of diagnostic material;
- date and time of the laboratory test appointment;
- date and time of material collection;
- the last name, first name, patronymic (if any) and position of the doctor or other authorized representative who ordered the laboratory test.
- the last name, first name, patronymic (if any) of the medical worker who collected the biomaterial.

When sending diagnostic materials for examination to the laboratory of another medical organization, in addition to the information listed above, the name of the medical organization to which the diagnostic material is being sent must be indicated.

Samples from patients with respiratory symptoms who have arrived from countries with reported cases of COVID-19 or who belong to risk groups must be marked with “Cito”. These samples must be sent to the laboratory and examined as a matter of priority.

The transfer of diagnostic samples from patients suspected of having COVID-19 is carried out with the provision of referrals and the execution of an Acceptance and Transfer Act, which must contain:

- name of the medical institution/department/unit that sent the samples for testing;
- name of the medical institution/department/unit that accepted the samples for testing;
- date of sample transfer;
- the last name, first name, patronymic and signature of the employee who submitted the samples;
- last name, first name, patronymic and signature of the employee who accepted the samples;
- list of samples to be transferred (with the designation of samples sent for “Cito” testing) and their quantity.

The act is drawn up in two copies, one for the sending organization, the other for the organization that accepted the samples.

Timeframe for completing the study

The time for submitting a conclusion on the results of the study upon receipt of negative, doubtful or positive results should not exceed 48 hours from the moment the biological material sample is received by the laboratory, except in cases of sample rejection. When prescribing the "Cito" study, the result should be provided within several hours, depending on the reagent kits used.

Requirements for premises and equipment of laboratories

Laboratory tests to detect the COVID-19 pathogen, classified as pathogenicity group II , must be carried out in compliance with the sanitary and epidemiological rules SP 1.3.3118-13 “Safety of

work with microorganisms
I-II pathogenicity (hazard) groups”, as well as when using molecular genetic methods (without accumulation of the pathogen) in laboratories that have a sanitary and epidemiological conclusion on the possibility of working with microorganisms of pathogenicity group III (clause 2.1.6. SP 1.3.3118-13 “Safety of working with microorganisms of pathogenicity (hazard) groups I-II ”) in accordance with the requirements of SP 1.3.2518-09 (1.3.2322-08) “Safety of working with microorganisms of pathogenicity (hazard) groups III-IV and causative agents of parasitic diseases”.

Requirements for the premises and equipment for specific laboratory (etiological) diagnostics of COVID-19 comply with the above sanitary rules and the rules set out in MU 1.3.2569-09 "Organization of work of laboratories using NAAT when working with material containing microorganisms of pathogenicity groups I-IV ". To prevent cross-contamination of samples with amplified products, work areas for isolating RNA and conducting NAAT with reverse transcription and recording its results when using the hybridization-fluorescence detection method should be located in separate rooms, remote from each other.

The laboratory must have equipment sufficient to perform NAAT for the diagnosis of COVID-19, taking into account the methods used and the volume of work.

The laboratory must have PPE (disposable protective suit type IV , masks, respirators, safety glasses, disposable latex (rubber) gloves, etc.) in sufficient quantities in accordance with SP 1.3.3118-13, including the required number of sets for each work area, the ability to change PPE during the day, a stock of PPE that ensures uninterrupted supply of personnel.

Laboratory stages of diagnostics

At the stage of receiving, sorting and registering the material, the laboratory must reject samples for which the information in the referral does not match the data on the label or in the Transfer Certificate, the terms and rules of transportation are violated, the containers are not hermetically sealed. The laboratory is obliged to inform the medical institution/department or the doctor who sent the samples about the rejection of the samples and the reason for it.

To conduct NAAT, laboratories use test systems/reagents registered in the Russian Federation as medical devices. Laboratory specialists must be proficient in NAAT.

If a positive result for COVID-2019 is received , it is necessary to immediately inform the territorial bodies of Rospotrebnadzor in the constituent entities of the Russian Federation and send biological material to federal budgetary healthcare institutions - hygiene and epidemiology centers in the constituent entities of the Russian Federation.

Disinfection, waste management

Disinfection measures are carried out in the laboratory premises taking into account zoning [XX]. In the premises of working zone 1 (reception and analysis of material, its marking, primary processing and disinfection), routine cleaning is carried out daily using disinfectants. In work zones 2 (isolation and purification of pathogen RNA from samples prepared in work zone 1), 3 (preparation of reaction mixtures, conducting a reverse transcription reaction, amplification of nucleic acids and recording the results using the hybridization fluorescence detection method) or 2-3, it is recommended to carry out daily: treatment of rooms with ultraviolet radiation before starting and after finishing work, treatment of the corresponding microbiological safety boxes and PCR boxes with ultraviolet radiation after finishing each

[XX]MR 3.1.0229-21. Recommendations for the organization of anti-epidemic measures in medical organizations providing medical care to patients with a new coronavirus infection (COVID-19)

stage of research (isolation of nucleic acids and amplification), treatment of the working surfaces of microbiological safety boxes and PCR boxes after finishing work with chlorine-active disinfectants (chloramine, disinfectants based on sodium dichloroisocyanuric acid, disinfectants based on

trichloroisocyanuric acid (DP-2T)) with the removal of residual disinfectants from the surfaces of microbiological safety boxes and PCR boxes, followed by treatment with ultraviolet radiation for 30 minutes before starting work, current wet cleaning of floors after completion of work with disinfectants approved for use.

During the research, waste of classes A, B, C and G is generated (SanPiN 2.1.7.2790-10 “Sanitary and Epidemiological Requirements for Handling Medical Waste”).

All used disposable materials and other waste are treated with disinfectants and then disposed of in accordance with SanPiN 2.1.7.2527-09 (2.1.7.728-99) “Rules for the collection, storage and disposal of waste from medical and preventive institutions” and MU 287-113 “Methodological guidelines for disinfection, pre-sterilization cleaning and sterilization of medical devices”.

Instructions for conducting diagnostics of COVID-19 using immunochemical methods

General Provisions

Immunochemical methods are used to detect SARS-CoV-2 antigens and antibodies to the virus and complement the complex of clinical, instrumental and laboratory methods for diagnosing COVID-19 with new information of clinical and epidemiological significance. Immunochemical methods include enzyme-linked immunosorbent assay (ELISA), immunochemiluminescence (ICL) and immunochromatography. The effectiveness of immunochemical methods depends on the stage of the infectious process, the state of the patient's immune system, as well as on the characteristics of the diagnostic kits used.

Immunochemical studies for the diagnosis of COVID-19 can be carried out in all laboratories of the Russian Federation, regardless of their organizational and legal form, that have a sanitary and epidemiological conclusion on the possibility of carrying out work with pathogens of human infectious diseases of III - IV pathogenicity and conditions for research using immunochemical methods. To conduct the study, diagnostic reagent kits (test systems) registered in the established manner on the territory of the Russian Federation must be used in accordance with the instructions for their use. The list of registered reagent kits is presented in State register of medical devices .

For the purpose of diagnosing COVID-19 using an indirect method, it is recommended to conduct separate testing for IgM/IgA and IgG class antibodies, as well as monitoring the appearance of antibodies over time (detection of seroconversion) - retesting in unclear cases after 5-7 days.

To minimize false positive results, it is recommended to introduce an algorithm for sequential testing of patients who have initially positive results for IgM/IgA or IgG antibodies using another test. For this purpose, it is necessary to use a test system with maximum sensitivity and specificity, as well as with the simultaneous detection of antibodies of classes A, M, G, which will act as a reference (verifying) test system. As such a test system, a test system for detecting the receptor-binding domain of the surface glycoprotein S of SARS-CoV-2 can be used .

Testing for antibodies to the SARS-Cov-2 virus is recommended for use:

- as an additional method for diagnosing acute infection (taking into account the seronegative period) or when it is impossible to study smears using NAAT, including during hospitalization due to somatic pathology;
- to establish the fact of a previous infection;
- to select potential donors of immune plasma.

Immunochemical methods for diagnosing COVID-19

SARS-CoV-2 antigens are detected using immunochromatographic methods (ELISA, ICL, or immunochromatography). Rapid tests for antigen detection (using immunochromatography) provide results in 10-30 minutes. The diagnostic sensitivity and specificity of rapid tests for SARS-CoV-2 antigen may be lower than those of NAAT-based tests.

SARS-CoV-2 antigen test results can be expected at the same time during the disease development as detection of viral RNA: 2 days before and 5-7 days after the onset of symptoms. A positive test result can be considered as a confirmation of the diagnosis of COVID-19, a negative result does not allow to exclude COVID-19.

Immunochromatographic tests can be used for rapid diagnostics of COVID-19 in situations where molecular genetic testing (NAAT) is not possible, including in remote and sparsely populated regions. It should be borne in mind that the SARS-CoV-2 antigen test has a lower positive predictive

value in cases of low disease prevalence.

Antibodies to SARS-CoV-2 are detected using ELISA, ICL or immunochromatography. The developed ELISA and ICL methods allow obtaining qualitative, semi-quantitative (positivity rate) and quantitative results. To determine the levels of immunoglobulins to SARS-CoV-2 , it is necessary to use reagent kits for the quantitative determination of antibodies, and the results of the studies should be presented using conventional units of measurement BAU/ml (binding antibody units).

Immunochromatographic methods are rapid diagnostic tests, considered as "bedside" tests (result time is 10-30 minutes). Immunochromatographic tests are qualitative tests and allow to determine the presence of antibodies to SARS-CoV-2 (IgM, IgG, total antibodies).

By detecting individual classes of antibodies to SARS-CoV-2 (IgA/IgM, IgG), it is possible to determine various phases of the infectious process:

- seronegative phase - antibodies may not be detected in the initial sample, but are detected in samples taken several days later;
- active phase - when determining a diagnostically significant level of IgA and/or IgM in one sample or a significant increase in the level of IgG in paired sera taken at an interval of 2-4 weeks;
- convalescence phase - the concentration of IgA and IgM, as a rule, decreases significantly (titer decreases by 2-4 times) during recovery, with IgG remaining 2 weeks after the course of treatment and later;
- past infection - persistence of IgG without an increase in its level in paired sera and the absence of IgA and IgM.

Determination of IgG to SARS-CoV-2 4 weeks after vaccination allows confirmation of the presence of post-vaccination immunity. The duration of the period of detectable presence of antibodies has not been determined, but is currently considered to be a period of at least 3 months.

Collection, storage and transportation of biological material samples

Preanalytical stage. The biomaterial for conducting immunochemical studies can be capillary blood, serum, plasma, whole venous blood (depending on the type of testing and according to the manufacturer's instructions).

Blood sampling is carried out in accordance with GOST R 53079.4-2008 Clinical laboratory technologies. Quality assurance of clinical laboratory studies. Part 4. Rules for conducting the preanalytical stage.

Blood tubes are transported in thermal containers labeled "blood samples for laboratory testing". The temperature in the thermal container must be maintained at +4 to +8 °C. Blood samples from individuals diagnosed with COVID-19 are placed in an additional secondary container, then in a thermal container labeled "samples with infected material".

Blood samples must be delivered to the laboratory as soon as possible. The laboratory research log records the time of delivery of samples to the laboratory.

Storage of whole blood samples is permitted at a temperature of 20-25°C for no more than 2 hours, at a temperature of 2-8°C - no more than 6 hours from the moment of collection of the biomaterial.

The shelf life of serum samples at a temperature of 2-8°C is no more than 4 days. Serum samples are stored frozen for 5 days to 1 year at a temperature no higher than minus 18°C. Long-term storage of samples (more than a year) is carried out at a temperature no higher than minus 40°C.

The analytical stage is carried out in accordance with the instructions for the reagent kit used for the study.

Post-analytical stage. The results of immunochemical studies are assessed in accordance with the instructions of the manufacturer of the medical devices used (test systems/reagent kits and equipment). Recommendations for interpreting the results of the studies are given in the table.

Interpretation of results of studies using nucleic acid amplification and immunochemical methods

SARS-CoV-2 Research Results				Interpretation*
RNA	Anti gene	IgM/ IgA	IgG	
-	-	-	-	Absence of current and previous COVID-19 infection
+	+	-	-	Acute phase of infection. Seronegative period. The result may precede the appearance
+	+	+	-	Acute phase of infection, Beginning of development of immune response
+	+	+	+	Acute phase of infection, pronounced immune response to COVID-19 infection
-	-	+	+	Late phase of the disease or recovery, pronounced immune response.
-	-	-	+	Previous COVID-19 infection or recovery. Immunity to SARS-CoV-2 has been developed.

* - the results of total antibody studies are interpreted in accordance with the type of antibody included in the definition

The results of immunochemical studies should be interpreted taking into account the anamnesis and clinical data, as well as the results of other laboratory and instrumental studies. It is also necessary to take into account the probability of obtaining false-positive and false-negative results.

False positive results may appear in the presence of so-called "cross-reacting" antibodies in the patient's blood, similar in their immunochemical properties to specific antibodies (other coronaviruses, rheumatoid factor IgM). False negative results may be obtained when examining biological samples taken at the seronegative stage of infection, or when using reagent kits (test systems) with a low sensitivity level. False negative results may be obtained when examining patients with reduced immunity.

False positive and false negative results may also appear when the rules for conducting laboratory tests are violated at all stages.

Disinfection and waste handling during COVID-19 diagnostics using immunochemical methods are carried out in the same way as the requirements for disinfection and waste handling using nucleic acid amplification methods (Appendix 3-1).

Algorithm for etiological diagnostics in patients with suspected COVID-19

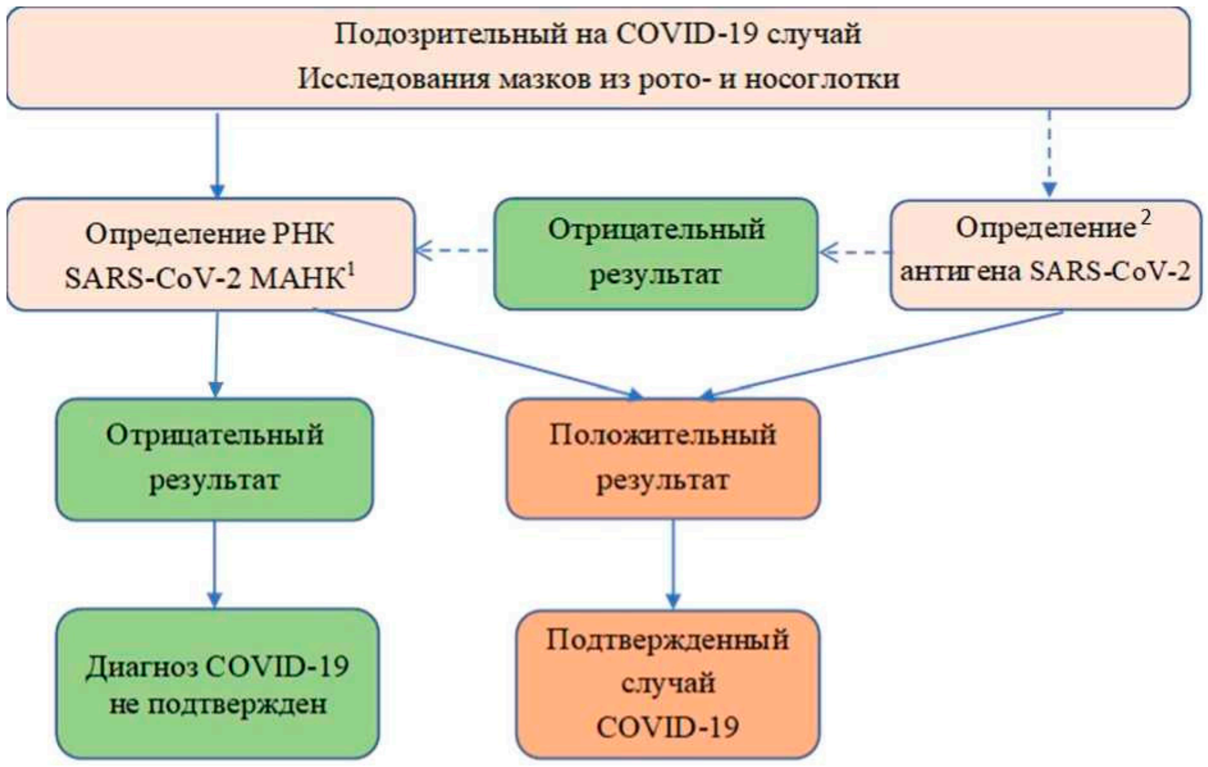

[1] NAAT – nucleic acid amplification tests (section 4; appendix 3.1.)
[2] SARS-CoV-2 antigen by immunochromatographic or other methods immunochemical methods (section 4; appendix 3.2.)

Appendix 3-4 Search for medical devices (test systems/reagent kits) registered in the Russian Federation for detecting SARS-CoV-2 and antibodies to it

Information on medical devices registered in the Russian Federation for detecting immunoglobulins to SARS-CoV-2 is posted in the State Register of Medical Devices and Organizations (Individual Entrepreneurs) Engaged in the Production and Manufacturing of Medical Devices (hereinafter referred to as the State Register), published on the official website of Roszdravnadzor www.roszdravnadzor.ru in the section "Electronic services" https://roszdravnadzor.gov.ru/services/misearch/

To search for a medical product, you can use the search bar of the “Advanced Search” service.

The "Advanced Search" line allows you to navigate by:

- registration certificate number;

- date of the registration certificate;
- name and country of manufacturer;
- type of nomenclature classification of a medical device, etc.

When using the search bar of the "Advanced Search" service, you need to set the necessary search criteria and click the "Display results" button.

Types of Nomenclature Classification of Medical Devices that are Diagnostic Reagent Kits for Detecting SARS-CoV-2 RNA

Item No.	Type of Nomenclature Classification	Name of the type of Nomenclature Classification of Medical Devices
1.	142160	SARS Coronavirus nucleic acid IVD, kit, nucleic acid analysis
2.	142190	SARS Coronavirus nucleic acid IVD, calibrator
3.	142220	SARS Coronavirus Nucleic Acid IDV Control Material
4.	142240	SARS Coronavirus nucleic acid IVD, reagent

Types of Nomenclature Classification of Medical Devices Representing are diagnostic reagent kits for the detection of immunoglobulins to SARS-CoV-2

Item No.	Type of Nomenclature Classification	Name of the type of Nomenclature Classification of Medical Devices
1.	142020	SARS Coronavirus IgA/IgG/IgM antibodies , control material
2.	142030	SARS Coronavirus Immunoglobulin G (IgG) antibodies IVD, calibrator
3.	142070	SARS Coronavirus Immunoglobulin G (IgG) antibodies IVD, control material
4.	142090	SARS Coronavirus antibodies class immunoglobulin G (IgG) IVD, reagent
5.	142060	SARS Coronavirus antibodies of classes immunoglobulin A (IgA)/IgG/IgM IVD, reagent
6.	142100	SARS Coronavirus immunoglobulin G (IgG)/IgM class antibodies IVD, kit, enzyme-linked immunosorbent assay (ELISA)
7.	142120	SARS Coronavirus Immunoglobulin G (IgG)/IgM IFA, Kit, Immunofluorescence Assay
8.	142130	SARS Coronavirus immunoglobulin M (IgM) class antibodies IVD, kit, enzyme immunoassay
9.	142150	SARS Coronavirus antibodies class immunoglobulin M (IgM) IVD, kit, immunofluorescence analysis
10.	142180	SARS Coronavirus immunoglobulin M (IgM) antibodies IVD, calibrator
11.	142200	SARS Coronavirus immunoglobulin G (IgG)/IgM antibodies IVD, reagent
12.	142210	SARS Coronavirus Immunoglobulin M (IgM) class antibodies IVD, control material
13.	142230	SARS Coronavirus antibodies class immunoglobulin M (IgM) IVD, reagent
14.	142250	SARS Coronavirus antibodies immunoglobulin A (IgA)/IgG/IgM IVD, kit, immunochromatographic assay, rapid assay
15.	142170	SARS Coronavirus Immunoglobulin G (IgG)/IgM IDV antibodies, control material

16.	142290	SARS Coronavirus antibodies immunoglobulin A (IgA)/IgG/IgM classes IVD, calibrator
17.	142260	SARS Coronavirus immunoglobulin G (IgG) class antibodies IVD, kit, enzyme-linked immunosorbent assay (ELISA)
18.	142300	SARS Coronavirus Immunoglobulin G (IgG) Antibodies IVD Kit Immunofluorescence Assay

Types of Nomenclature Classification of Medical Devices Representing

rapid tests for the detection of immunoglobulins to SARS-CoV-2

Item No.	Type of Nomenclature Classification	Name of the type of Nomenclature Classification of Medical Devices
1	142250	SARS Coronavirus antibodies immunoglobulin A (IgA)/IgG/IgM IVD, kit, immunochromatographic assay, rapid assay

Types of Nomenclature Classification of Medical Devices Representing

are diagnostic reagent kits for detection of SARS-CoV-2 antigen

Item No.	Type of Nomenclature Classification	Name of the type of Nomenclature Classification of Medical Devices
1.	142010	SARS Coronavirus Antigens IVD, Kit, Immunochromatographic Analysis, Rapid Analysis
2.	142050	SARS Coronavirus Antigens IVD, Calibrator
3.	142080	SARS Coronavirus Antigens IVD, Control Material
4.	142110	SARS Coronavirus Antigens IVD, Reagent
5.	142280	SARS Coronavirus Antigens IVD, Kit, Enzyme Immunoassay (ELISA)

Appendix 4

Drug interactions of antithrombotic and anticoagulant drugs for the treatment of patients with COVID-19

Preparation	Favipiravir	Remdesivir	Tocilizumab, sarilumab	Baricitinib	Dexamethasone
Acenocoumarol	↔	↔	↓	↔	↑
Apixaban	↔	↔	↓	↔	↓
Acetylsalicylic acid	↔	↔	↔	↔	
Clopidogrel	↔	↔	↓	↔	
Dabigatran	↔	↔	↔	↔	↓
Dipyridamole	↔	↔	↔	↔	
Enoxaparin	↔	↔	↔	↔	
Fondaparinux	↔	↔	↔	↔	
NFG	↔	↔	↔	↔	
Prasugrel	↔	↔	↓	↔	
Rivaroxaban	↔	↔	↓	↔	↓
Streptokinase	↔	↔	↔	↔	
Ticagrelor	↔	↔	↓	↔	
Warfarin	↔	↔	↓	↔	↑
↑	Increases exposure to antithrombotic drug				
↓	Reduces exposure to antithrombotic drug				
←→	Does not affect the exposure of the antithrombotic drug				
	The drugs should not be prescribed simultaneously.				
	The drugs may potentially interact and dose adjustments and monitoring may be required.				
	The drugs interact weakly.				
	The drugs do not interact				

Appendix 5

List of possible drugs to be prescribed for the treatment of COVID-19 in adults

Drug (INN)	Mechanism of action	Release forms	Assignment schemes	Contraindications, special instructions, side effects
**Favipiravir**	Inhibits RNA-dependent RNA polymerase of the SARS-CoV-2 virus	Pills	For weight <75 kg: 1600 mg 2 times a day on the 1st day and then 600 mg 2 times a day on days 2-10.<br><br>For those weighing 75 kg or more: 1800 mg 2 times a day on the 1st day, then 800 mg 2 times a day from the 2nd to the 10th day.	• Hypersensitivity to favipiravir;<br>• Severe liver failure;<br>• SCF < 30 ml/min;<br>• Pregnancy or planning a pregnancy while taking the drug and for 7 days after its completion (women and men should use the most effective methods of contraception during sexual intercourse, for example, a condom with spermicide);<br>• Breastfeeding period;<br>• Children under 18 years of age.<br>With caution:<br>In patients with a history of gout and hyperuricemia, in elderly patients, in patients with mild to moderate hepatic impairment, in patients with moderate renal impairment<br>(SCF < 60 ml/min and ≥ 30 ml/min).<br>Can be used both in outpatient and inpatient settings.
**Remdesivir**	The active metabolite acts as an adenosine triphosphate (ATP) analogue and competes with the natural ATP substrate for incorporation into nascent RNA chains by the SARS-CoV-2 RNA-dependent RNA polymerase.	Lyophilisate	The lyophilisate is diluted in 0.9% sodium chloride solution (to a total volume of 100 or 250 ml) and administered intravenously over 30-120 minutes.<br>Day 1: 200 mg intravenously once.<br>From the 2nd day: 100 mg intravenously 1 time per day. The total course is no more than 10 days.	Contraindicated:<br>• children (under 18 years old);<br>• patients with SCF < 30 ml/min;<br>• with ALT ≥ 5 ULN;<br>• during pregnancy and breastfeeding.<br><br>It is prohibited to administer intramuscularly.

**Human immunoglobulin against COVID-19**	The antiviral effect is due to virus binding specific antibodies to SARS-CoV-2 class IgG	Solution for infusions	Intravenously by drip at a dose of 4 ml/kg of body weight. The initial rate of administration is from 0.01 to 0.02 ml/kg of body weight per minute for 30 minutes. If the drug is well tolerated, the rate of administration can be gradually increased to a maximum of 0.12 ml/kg of body weight per minute.	• Hypersensitivity to human immunoglobulin, especially in rare cases of deficiency of immunoglobulin class A (IgA) in the blood and the presence of antibodies against IgA;<br>• Hypersensitivity to the components of the drug;<br>• History of allergic reactions to human blood products;<br>• Age under 18 years and over 60 years (due to lack of data on efficacy and safety);<br>• Pregnancy and breastfeeding period;<br>• Autoimmune diseases.
**IFN -α**	It has local immunomodulatory, anti-inflammatory and antiviral effects.	Intranasal forms: spray, drops, solution, lyophilisate for solution preparation, gel or ointment	In accordance with the instructions for use of the drug	Pregnant women are prescribed only recombinant IFN-α2b
**Umifenovir**	Refers to fusion inhibitors, interacts with hemagglutinin virus and prevents the fusion of the lipid membrane of the virus and cell membranes.	Capsules	200 mg 4 times a day for 5-7 days	Contraindicated during pregnancy.

Preemptive Anti-inflammatory Therapy for COVID-19 in Adults

Drug (INN)	Mechanism of action	Release forms	Assignment schemes	Contraindications
**Baricitinib**		Pills	4 mg 1 time per day for 7-14 days	
**Tofacitinib**	Selective inhibitors of Janus kinases (JAK1 and JAK2).	Pills	10 mg 2 times a day for 7-14 days	different from COVID-19<br>• Lymphopenia $<0.5*10^{9}/l$,<br>• Neutropenia $<1*10^{9}/l$,<br>• Hemoglobin <8 g/dl,<br>• Creatinine clearance <30 ml/min,<br>• Severe liver failure/if drug-induced liver injury is suspected,<br>• Active hepatitis B and/or C,<br>• Active tuberculosis,<br>• History of pulmonary embolism/pulmonary embolism<br>With caution: age over 75 years, taking COX-2 inhibitors
**Netakimab**	Recombinant humanized monoclonal antibodies inhibiting interleukin-17A (IL-17A)	Solution for subcutaneous administration	120 mg in the form of two subcutaneous injections of 1 ml (60 mg) of the drug each.	• Hypersensitivity to netakimab, as well as to any of the excipients of the drug.<br>• Clinically significant infectious diseases in the acute phase, including tuberculosis.<br>• Children and adolescents up to 18 years of age.<br>Pregnancy, breastfeeding.

<table>
<tr>
<td>Olokizumab</td>
<td>Humanized monoclonal antibodies of the immunoglobulin G4/kappa isotype, developed as an antagonist of IL-6</td>
<td>Solution for subcutaneous administration</td>
<td>64 mg (one vial 160 mg/ml 0.4 ml) subcutaneously or intravenously. If the effect is insufficient, repeat the intravenous administration after 24 hours. In total, administer no more than 256 mg.

128 mg (two vials of 160 mg/ml 0.4 ml) are diluted in 100 ml of 0.9% NaCl solution and administered intravenously by drip over 60 minutes. If the effect is insufficient, repeat the administration after 12 hours.
Do not administer more than 256 mg in total.

256 mg (four vials of 160 mg/ml 0.4 ml) are diluted in 100 ml of 0.9% NaCl solution and administered intravenously by drip once over 60 minutes.</td>
<td rowspan="3">• Sepsis confirmed by pathogens other than COVID-19;
• Hypersensitivity to any component of the drug;
• Viral hepatitis B;
• Comorbidities associated with poor prognosis;
• Immunosuppressive therapy in organ transplantation;
• Neutropenia is $<0.5*10^{9}$/l;
• Increase in AST or ALT activity more than 5 times;
• Thrombocytopenia $<50*10^{9}$/l.

Not recommended during pregnancy and lactation.</td>
</tr>
<tr>
<td>Levilimab</td>
<td rowspan="2">Monoclonal antibodies that inhibit IL-6 receptors.</td>
<td>Solution for subcutaneous administration</td>
<td>324 mg (two pre-filled syringes of 162 mg/0.9 ml) subcutaneously or intravenously once. If the effect is insufficient, repeat the administration after 24 hours.

648 mg (four pre-filled syringes of 162 mg/0.9 ml) are diluted in 100 ml of 0.9% NaCl solution and administered intravenously by drip over 60 minutes in combination with GCS.</td>
</tr>
<tr>
<td>Tocilizumab</td>
<td>Concentrate for preparation of solution for infusions</td>
<td>4-8 mg/kg/administration in combination with GCS. 400 mg is diluted in 100 ml of 0.9% NaCl solution, administered intravenously by drip over 60 minutes.
Do not administer more than 800 mg.
If the effect is insufficient, repeat the administration after 12 hours*.</td>
</tr>
</table>

**Sarilumab**		Solution in a syringe pen	200 mg or 400 mg in combination with GCS.<br>A pre-filled syringe-pen at a dosage of 200 mg (1 or 2 syringes depending on the dose) is diluted in 100 ml of 0.9% NaCl solution, administered intravenously by drip over 60 minutes.<br>If the effect is insufficient, repeat the administration after 12 hours.	
**Canakinumab**	Monoclonal antibodies that inhibit IL-1β receptors.	Lyophilisate	Canakinumab 4-8 mg/kg IV in combination with GCS. Prescribed when tocilizumab or sarilumab cannot be used or is ineffective.<br>150 mg of lyophilisate is dissolved in 1 ml of water for injection. The prepared concentrate is introduced into a vial with 250 ml of 5% glucose solution. Do not shake.<br>Dose of canakinumab (volume of concentrate for solution preparation (150 mg/ml) 300 mg – 2 ml<br>150 mg – 1 ml	
**Anakinra**	Interleukin inhibitors, a key proinflammatory cytokine	Solution for subcutaneous administration	200 – 400 mg/day, intravenously by drip for 10 days	
**Methylprednisolone**	Belong to glucocorticosteroids, have immunoinflammatory, immunosuppressive, anti-shock pharmacological action. Affect all phases of inflammation.	Solution	In moderate cases:<br>- without IL-6 receptor antagonists or IL-6 blockers - at a dose of 125 mg/administration intravenously every 12 hours. OR<br>- 250 mg/intravenous administration once daily.<br>In moderate cases with respiratory failure, in combination with IL-6 or IL-6 receptor blockers - at a dose of 60 mg/administration/intravenously every 8 hours.<br>In severe cases:<br>- without IL-6 receptor antagonists or IL-6 or IL-1β blockers - at a dose of 125 mg/administration intravenously every 6-8 hours. OR	**Use with caution in:**<br>• Diabetes mellitus,<br>• Hypertension,<br>• Gastric ulcer and duodenal ulcer,<br>• Obesity,<br>• Signs of an active bacterial infection,<br>• Thrombotic disorders.

			OR<br>- 250 mg/intravenous administration once.<br>In severe cases:<br>- in combination with IL-6 receptor antagonists or IL-6 or IL-1b blockers - at a dose of 60 mg/administration/intravenously every 6-8 hours.<br>The maximum dose of Methylprednisolone is used for 3-4 days.<br>The dose of methylprednisolone is reduced by 20-25 % per administration every 1-2 days, then by 50% every 1-2 days until complete withdrawal.<br>Dose reduction begins under the condition of stable (within 3-4 days) relief of fever, a decrease in the level of CRP and the level of serum ferritin by at least 15%.	
		Pills	6-12 mg - once in the morning, after meals, 12 hours before the start of reducing the dose of methylprednisolone for intravenous administration, for 7 days, from the 8th day, gradually reducing the dose by 2 mg per day.	
**Dexamethasone**		Solution	In moderate cases:<br>- without IL-6 receptor antagonists or IL-6 blockers - at a dose of 16-20 mg/day intravenously, depending on the severity of the patient's condition, in 2 administrations.<br>In moderate cases with respiratory failure:<br>- in combination with IL-6 receptor antagonists or IL-6 blockers - at a dose of 16-24 mg/day intravenously, depending on the severity of the patient's condition, in 2 administrations.<br>In severe cases:<br>- in combination with IL-6 receptor antagonists or IL-6 or IL-1b blockers	

			- at a dose of 16-24 mg/day intravenously, depending on the severity of the patient's condition, in 2 administrations.<br>- without IL-6 receptor blockers or IL-6 or IL-1b blockers - at a dose of 24 mg/day/intravenously in 2 administrations. The maximum dose of dexamethasone is used for 3-4 days. The dose of Dexamethasone is reduced by 20-25% per administration every 1-2 days, then by 50% every 1-2 days until complete cancellation.<br>Dose reduction begins under the condition of stable (within 3-4 days) relief of fever, a decrease in the level of CRP and the level of serum ferritin by at least 15%.	
**Hydrocortisone**		Solution	Intravenous (bolus) administration at a dose of 50-100 mg, followed by slow intravenous administration over an hour at a dose of 200 mg per day only in the event of adrenal insufficiency.	
**Budesonide**	Refers to glucocorticosteroids, has anti-inflammatory action. Affects all phases of inflammation.	Dosed powder for inhalation	800 mcg 2 times a day until recovery, but not more than 14 days******	• Children under 6 years of age;<br>• Hypersensitivity to budesonide.<br><br>With caution: (more careful monitoring of patients is required) the drug should be prescribed to patients with active pulmonary tuberculosis, fungal, bacterial infections of the respiratory system, and liver cirrhosis.<br>When prescribing, the possible manifestation of the systemic action of GCS should be taken into account.

* - Additional administration of IL-6 inhibitors at the same dose after 12 hours in the absence or insufficient clinical effect (fever was not relieved)

******- For patients with bronchial asthma, the dosage of budesonide should be increased to 1600 mcg per day.

Anticoagulants for the treatment of COVID-19 in adults

Preparation	Preventive dose	Intermediate dose [2]	Therapeutic dose
**Anticoagulants for parenteral administration**			
**Unfractionated heparin**			
Unfractionated heparin	Subcutaneously 5000 IU 2-3 times/day	Subcutaneously 7500 IU 2-3 times/day	IV infusion is optimally controlled by anti-Xa activity (APTT may increase in COVID-19, so it may be unreliable). The initial dose for venous thromboembolic complications is an intravenous bolus of 80 U/kg (maximum 5000 U) and infusion at an initial rate of 18 U/kg/h.
**Low molecular weight heparins**			
Dalteparin sodium [1]	Subcutaneously 5000 anti-Xa IU 1 time/day.	Subcutaneously 5000 anti-Xa IU 2 times/day	Subcutaneously 100 anti-Xa IU/kg 2 times/day
Nadroparin calcium [1]	Subcutaneously 3800 anti-Xa IU (0.4 ml) 1 time/day for body weight <70 kg or 5700 anti-Xa IU (0.6 ml) 1 time/day for body weight >70 kg	Subcutaneously 5700 anti-Xa IU (0.6 ml) 2 times/day	Subcutaneously 86 anti-Xa IU/kg 2 times/day
Enoxaparin sodium [1]	Subcutaneously 4000 anti-Xa IU (40 mg) 1 time/day	Subcutaneously 4000 anti-Xa IU (40 mg) 2 times/day; possibly increased to 50 IU (0.5 mg)/kg 2 times/day	Subcutaneously 100 anti-Xa IU (1 mg)/kg 2 times/day, with creatinine clearance of 15-30 ml/min 100 anti-Xa IU (1 mg)/kg 1 time/day
Parnaparin sodium [1]	Subcutaneously 0.3 ml (3200 anti-Xa IU) or 0.4 mg (4250 anti-Xa IU) 1 time/day	Subcutaneously 0.3 ml (3200 anti-Xa IU) 2 times/day	Subcutaneously 0.6 ml (6400 anti-Xa IU) 2 times/day
Bemiparin sodium [1]	Subcutaneously 2500 anti-Xa IU 1 time/day	Subcutaneously 3500 anti-Xa IU 1 time/day	
**Synthetic anticoagulants**			

Fondaparinux sodium [1]	Subcutaneously 2.5 mg 1 time/day. With creatinine clearance of 20-50 ml/min - 1.5 mg 1 time/day		Treatment of DVT/PE: 5 mg once daily for body weight up to 50 kg; 7.5 mg once daily for body weight 50-100 kg; 10 mg once daily for body weight over 100 kg

Oral anticoagulants [3]			
Rivaroxaban	10 mg 1 time/day		Treatment of DVT/PE: 15 mg 2 times a day for 21 days, then 20 mg 1 time a day for at least 3 months
Apixaban	2.5 mg 2 times a day		Treatment of DVT/PE: 10 mg 2 times/day for 7 days, then 5 mg 2 times/day for at least 3 months
Dabigatran etexilate [4]	110 mg 2 times/day; 75 mg 2 times/day in patients with creatinine clearance of 30-49 ml/min		Treatment of DVT/PE: after at least 5 days of administration of therapeutic doses of parenteral anticoagulants 150 mg 2 times a day for at least 3 months

Notes:

1 — contraindicated in cases of severe renal failure (see instructions for the drugs);

2 — there is no single definition of intermediate doses of anticoagulants;

3 - in the absence of anticoagulants for parenteral administration;

4 — the efficacy of dabigatran etexilate in the prevention of DVT/PE has been studied only in major orthopedic interventions.

Routine monitoring of anti-Xa activity in the blood is not required when anticoagulants are administered subcutaneously. However, if possible, it is advisable to use it to select a dose in patients with very low or high body weight, severe renal impairment, high risk of bleeding, and during pregnancy. Target values for prophylactic use are 0.2-0.6 anti-Xa U/ml, for therapeutic doses 0.6-1.0 anti-Xa U/ml. When using LMWH, blood for determining anti-Xa activity is taken 4-6 hours after administration of the drug (optimally after 3-4 injections), with subcutaneous administration of intermediate doses of UFH - in the middle between injections, with intravenous infusion of UFH - 6 hours after each change in dose.

Приложение 7-3

Algorithm for the use of anticoagulants for the treatment of COVID-19 in adult patients in a hospital setting

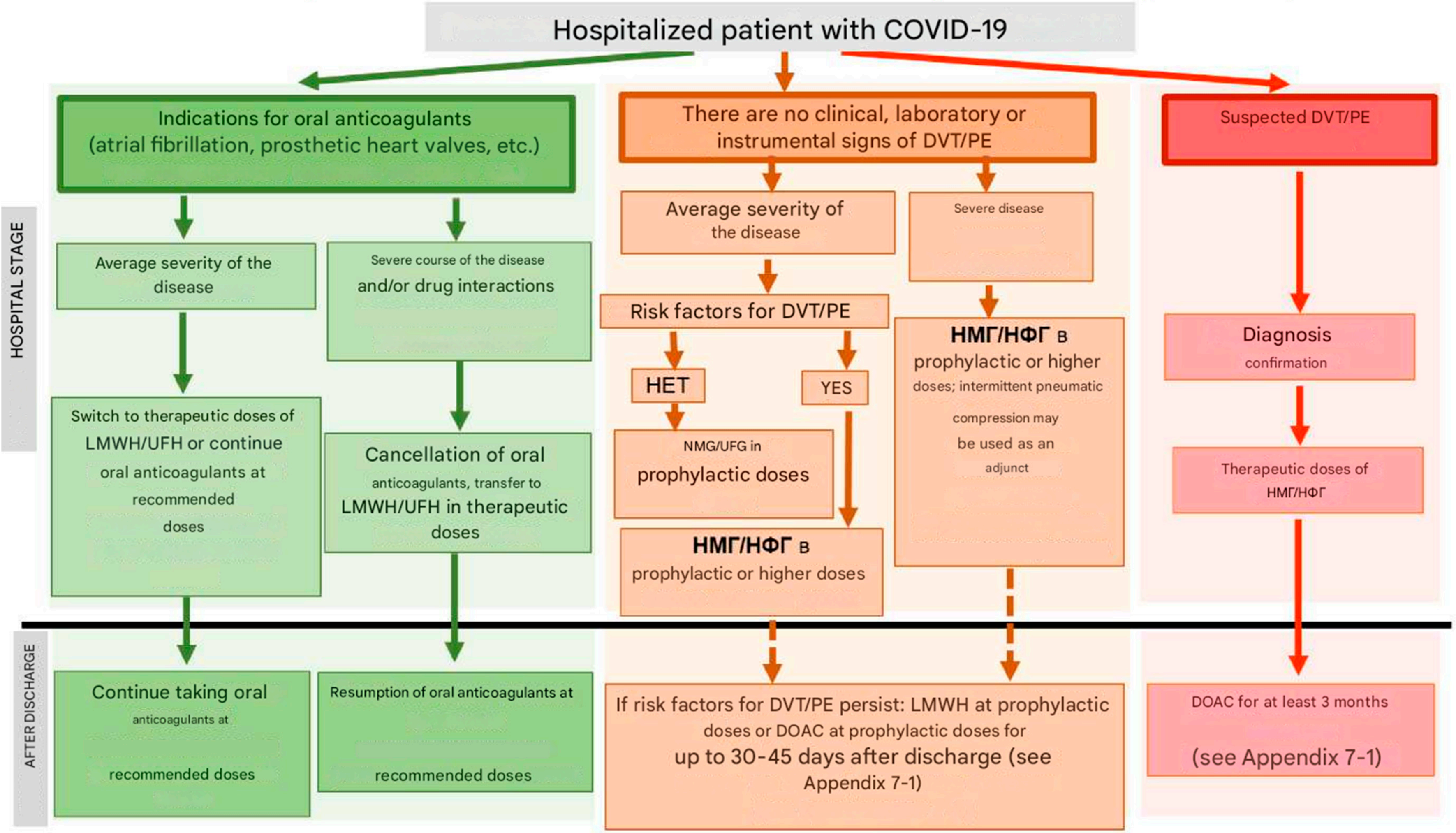

Algorithm for the use of anticoagulants for the treatment of COVID-19 in adult patients in an outpatient setting

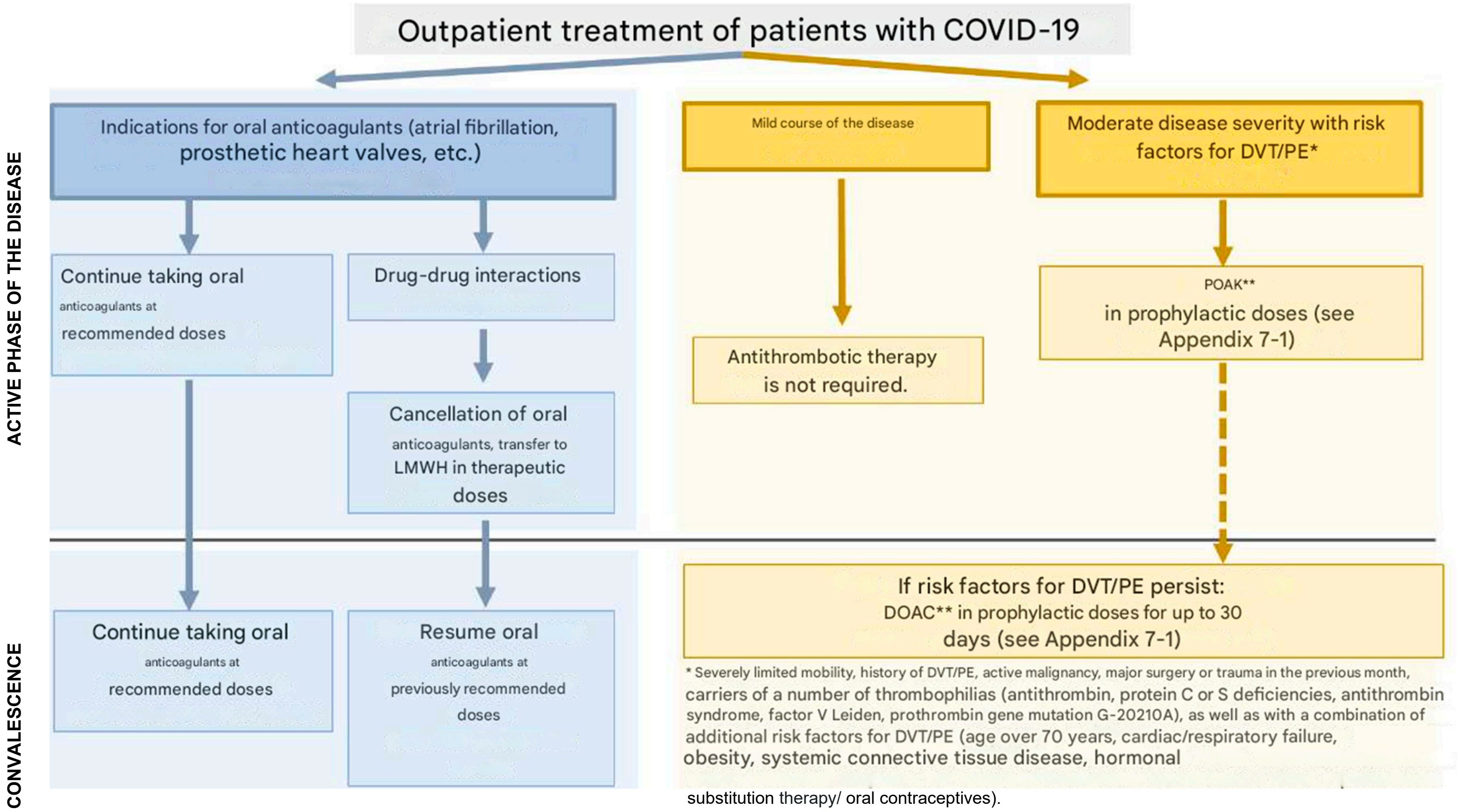

* Severely limited mobility, history of DVT/PE, active malignancy, major surgery or trauma in the previous month, carriers of a number of thrombophilias (antithrombin, protein C or S deficiencies, antithrombin syndrome, factor V Leiden, prothrombin gene mutation G-20210A), as well as with a combination of additional risk factors for DVT/PE (age over 70 years, cardiac/respiratory failure, obesity, systemic connective tissue disease, hormonal substitution therapy/ oral contraceptives).

** In the absence of LMWH.

Appendix 8-1

Recommended treatment regimens in outpatient settings

	No.	Preparation	Dosage regimen
**Mild flow**			
**Scheme 1**[1,4]	1[3]	Favipiravir[5]	For patients weighing <75 kg:<br>1600 mg 2 times a day on the 1st day and then 600 mg 2 times a day from days 2 to 10.<br>For patients weighing 75 kg or more:<br>1800 mg 2 times a day on the 1st day, then 800 mg 2 times a day from the 2nd to the 10th day.
	2	IFN-α, intranasal forms[5]	In accordance with the instructions for use of the drug (spray, drops, solution, lyophilisate for solution preparation, gel or ointment)
	3	Paracetamol	1-2 tablets (500-1000 mg) 2-3 times a day, no more than 4 g per day (at body temperature > 38.0 ºC)
**Scheme 2**[1,4]	1[3]	Umifenovir[5]	200 mg 4 times a day for 5-7 days
	2	IFN-α, intranasal forms[5]	In accordance with the instructions for use of the drug (spray, drops, solution, lyophilisate for solution preparation, gel or ointment)
	3	Paracetamol	1-2 tablets (500-1000 mg) 2-3 times a day, no more than 4 g per day (at body temperature > 38.0 ºC)
**Scheme 3**[2,4]	1[3]	Favipiravir[5]	For patients weighing <75 kg:<br>1600 mg 2 times a day on the 1st day and then 600 mg 2 times a day from days 2 to 10.<br>For patients weighing 75 kg or more:<br>1800 mg 2 times a day on the 1st day, then 800 mg 2 times a day from the 2nd to the 10th day.
	2	IFN-α, intranasal forms[5]	In accordance with the instructions for use of the drug (spray, drops, solution, lyophilisate for solution preparation, gel or ointment)
	3	Budesonide (dosed inhalation powder)	800 mcg 2 times a day until recovery, but not more than 14 days
	4	Paracetamol	1-2 tablets (500-1000 mg) 2-3 times a day, no more than 4 g per day (at body temperature > 38.0 ºC)
**Scheme 4**[2,4]	1[3]	Umifenovir[5]	200 mg 4 times a day for 5-7 days
	2	IFN-α, intranasal forms[5]	In accordance with the instructions for use of the drug (spray, drops, solution, lyophilisate for solution preparation, gel or ointment)

	3	Budesonide (dosed inhalation powder)	800 mcg 2 times a day until recovery, but not more than 14 days
	4	Paracetamol	1-2 tablets (500-1000 mg) 2-3 times a day, no more than 4 g per day (at body temperature > 38.0 ºC)
**Moderately severe course**			
**Scheme 1**	1	Favipiravir	For patients weighing <75 kg:<br>1600 mg 2 times a day on the 1st day and then 600 mg 2 times a day from the 2nd to the 10th day.<br>For patients weighing 75 kg or more:<br>1800 mg 2 times a day on the 1st day, then 800 mg 2 times a day from the 2nd to the 10th day.
	2	IFN-α, intranasal forms	In accordance with the instructions for use of the drug (spray, drops, solution, **lyophilisate** for solution preparation, gel or ointment)
	3	Paracetamol	1-2 tablets (500-1000 mg) 2-3 times a day, no more than 4 g per day (at body temperature > 38.0 ºC)
	4	Rivaroxaban	10 mg 1 time per day for up to 30 days
		or	
		Apixaban	2.5 mg 2 times a day for up to 30 days
		or	
		Dabigatran etexilate [6]	110 mg 2 times a day;<br>75 mg 2 times a day in patients with creatinine clearance of 30-49 ml/min for up to 30 days

1 - the regimen is prescribed when the body temperature rises to <38 ° C for less than 3 days;
2 - the regimen is prescribed when the body temperature increases to <38 ° C for more than 3 days;
3 — if it is possible to organize treatment in a day hospital, consider alternative etiotropic treatment with virus-neutralizing antibodies or human immunoglobulin against COVID-19 for patients with a high comorbidity index or pregnant women;
4 — it is recommended to prescribe an oral anticoagulant in the presence of risk factors for thrombosis (patients with severely limited mobility, a history of DVT/PE, active malignancy, major surgery or trauma in the previous month, carriers of a number of thrombophilias (antithrombin deficiency, protein C or S, antiphospholipid syndrome, factor V Leiden, prothrombin gene mutation G-20210A), as well as in the presence of a combination of additional risk factors for DVT/PE: cardiac/respiratory failure, obesity, systemic connective tissue disease, hormone replacement therapy, oral contraceptive use);
5 – during the first 7 days of illness or with a positive laboratory test result for SARS-CoV-2 RNA NAAT
6 — the efficacy of dabigatran etexilate in the prevention of DVT/PE has only been studied in major orthopedic interventions.

Antibacterial therapy for 3-7 days is prescribed in accordance with section 5.4 only in the presence of convincing signs of bacterial infection, including leukocytosis > $12x10^9$/l (in the absence of previous use of glucocorticosteroids), band shift ≥ 10%, the appearance of purulent sputum, an increase in the level of procalcitonin (with a procalcitonin level ≤0.1 ng / ml, the probability of a bacterial infection is low, with a level ≥0.5 ng / ml, the probability of a bacterial infection is high)

Recommended treatment regimens in a hospital setting

	No.	Preparation	Dosage regimen
**Mild course (patients with a high comorbidity index)**			
Scheme 1	1	Favipiravir [3]	For patients weighing < 75 kg:<br>1600 mg 2 times a day on the 1st day and then 600 mg 2 times a **day from** the 2nd to the 10th day.<br>For patients weighing 75 kg or more:<br>1800 mg 2 times a day on the 1st day, then 800 mg 2 times a day from the 2nd to the 10th day .
		**or**	
		Remdesivir [3]	Day 1: 200 mg (in 0.9% sodium chloride solution) once intravenously.<br>From the 2nd day: 100 mg intravenously 1 time per day.<br>The general course is no more than 10 days.
		**or**	
		Human immunoglobulin against COVID-19 [3]	1 ml per kg of body weight once intravenously by drip without dilution
	2	Baricitinib	4 mg 2 times a day for 7-14 days
		**or**	
		Tofacitinib	10 mg 2 times a day for 7-14 days
		**or**	
		Netakimab	120 mg subcutaneously once in the form of two subcutaneous injections of 1 ml (60 mg) of the drug each
		**or**	
		Levilimab	324 mg (two pre-filled syringes of 162 mg/0.9 ml) subcutaneously once. If the effect is insufficient, repeat the subcutaneous/intravenous administration after 24 hours
		**or**	
		Olokizumab	64 mg (1 bottle 160 mg/ml, 0.4 ml) subcutaneously.<br>If the effect is insufficient, repeat the intravenous administration after 24 hours.<br>Do not administer more than 256 mg in total.
	3	Anticoagulant drug for parenteral administration in prophylactic dose, see Appendix 7-1	

Moderately severe course			
**Scheme 1**	1	Favipiravir [3]	For patients weighing <75 kg:<br>1600 mg 2 times a day on the 1st day and then 600 mg 2 times a day from the 2nd to the 10th day.<br>For patients weighing 75 kg or more:<br>1800 mg 2 times a day on the 1st day, then 800 mg 2 times a day from the 2nd to the 10th day.
		**or**	
		Remdesivir	Day 1: 200 mg (in 0.9% sodium chloride solution) intravenously once. From the 2nd day: 100 mg intravenously once a day.<br>The general course is no more than 10 days.
		**or**	
		Human immunoglobulin against COVID-19 [3]	1 ml per kg of body weight once intravenously by drip without dilution
	2	Levilimab	324 mg (two pre-filled syringes of 162 mg/0.9 ml) intravenously once, diluted in 100 ml of 0.9% NaCl solution, administered intravenously by drip over 60 minutes). If the effect is insufficient, repeat the administration after 12 hours.
		**or**	
		Olokizumab	64 – 128 mg (1 or 2 vials of 160 mg/ml, 0.4 ml) diluted in 100 ml of 0.9% NaCl solution, administered intravenously by drip over 60 minutes)<br>If the effect is insufficient, repeat the administration after 12 hours.<br>Do not administer more than 256 mg in total.
		**or**	
		Tocilizumab	4 mg/kg body weight intravenously
		**or**	
		Sarilumab	200 mg diluted in 100 ml of 0.9% NaCl solution, administered intravenously, if the effect is insufficient, repeat the administration after 12 hours.
	3	Methylprednisolone	60 mg/administration/intravenously every 8 hours. The maximum dose is used for 3-4 days. The dose of GCS is reduced by 20-25% per administration every 1-2 days, then by 50% every 1-2 days until complete withdrawal.
		**or**	
		Dexamethasone [4]	16-24 mg/day intravenously depending on the severity of the patient's condition in 2 doses. The maximum dose is used for 3-4 days. The dose of GCS is reduced by 20-25% per dose every 1-2 days, then by 50% every 1-2 days until complete withdrawal;
	**4**	Anticoagulant drug for parenteral administration, see Appendix 7-1	

**Scheme 2**	1	Favipiravir [3]	For patients weighing <75 kg:<br>1600 mg 2 times a day on the 1st day and then 600 mg 2 times a day from the 2nd to the 10th day.<br>For patients weighing 75 kg or more:<br>1800 mg 2 times a day on the 1st day, then 800 mg 2 times a day from the 2nd to the 10th day.
		**or**	
		Remdesivir	Day 1: 200 mg (in 0.9% sodium chloride solution) once intravenously.<br>From the 2nd day: 100 mg intravenously 1 time per day.<br>The general course is no more than 10 days.
		**or**	
		Human immunoglobulin against COVID-19 [3]	1 ml per kg of body weight once intravenously by drip without dilution
	2	Methylprednisolone	125 mg/administration/intravenously every 12 hours. The maximum dose is used for 3-4 days. The dose of GCS is reduced by 20-25% per administration every 1-2 days, then by 50% every 1-2 days until complete withdrawal.<br>**or**<br>250 mg/administration/IV once a day for 3 days, with a gradual reduction in dose by 20-25% per administration every 1-2 days for 3-4 days, then by 50% every 1-2 days until complete discontinuation.
		**or**	
		Dexamethasone	16-20 mg/day intravenously depending on the severity of the patient's condition in 2 doses. The maximum dose is used for 3-4 days. The dose of GCS is reduced by 20-25% per dose every 1-2 days, then by 50% every 1-2 days until complete withdrawal.
	3	In case of fever (t > 38.0 °C) for more than 3 days, antibacterial and antimycotic therapy is prescribed according to indications in accordance with section 5.4.	
	4	Anticoagulant drug for parenteral administration, see Appendix 7-1	

Severe and extremely severe course (pneumonia with respiratory failure, ARDS)			
**Scheme 1**	1	Remdesivir	Day 1: 200 mg (in 0.9% sodium chloride solution) once intravenously. From the 2nd day: 100 mg intravenously 1 time per day.<br>The general course is no more than 10 days.
		**or**	
		Human immunoglobulin against COVID-19 [3]	1 ml per kg of body weight once intravenously by drip without dilution
	2	Tocilizumab	4-8 mg/kg body weight intravenously<br>400 mg is diluted in 100 ml of 0.9% NaCl solution and administered intravenously by drip.<br>Do not administer more than 800 mg.<br>If the effect is insufficient (febrile fever is not relieved), repeat the administration after 12 hours.
		**or**	
		Sarilumab	400 mg (pre-filled 200 mg syringe pen) diluted in 100 ml of 0.9% NaCl solution, administered intravenously, if the effect is insufficient, repeat the administration after 12 hours.
	3	Methylprednisolone	60 mg/administration/intravenously every 6-8 hours. The maximum dose is used for 3-4 days. The dose of GCS is reduced by 20-25% per administration every 1-2 days, then by 50% every 1-2 days until complete withdrawal.
		**or**	
		Dexamethasone	16-24 mg/day intravenously depending on the severity of the patient's condition in 2 doses. The maximum dose is used for 3-4 days. The dose of GCS is reduced by 20-25% per dose every 1-2 days, then by 50% every 1-2 days until complete withdrawal.
	4	Anticoagulant drug for parenteral administration, see Appendix 7-1	
	5	In case of fever (t > 38.0 °C) for more than 3 days, antibacterial and antimycotic therapy is prescribed according to indications in accordance with section 5.4.	
**Scheme 2**	1	Remdesivir	Day 1: 200 mg (in 0.9% sodium chloride solution) once intravenously. From the 2nd day: 100 mg intravenously 1 time per day.<br>The general course is no more than 10 days.
		**or**	
		Human immunoglobulin against COVID-19 [3]	1 ml per kg of body weight once intravenously by drip without dilution
	2	Canakinumab	4-8 mg per kg of body weight. 150 mg in 1 ml of water for injection, then injected into a vial with 250 ml of 5% glucose solution.

		**or**	

		Anakinra	200 – 400 mg/day, intravenously by drip for 10 days
	3	Methylprednisolone	60 mg/administration/intravenously every 6-8 hours. The maximum dose is used for 3-4 days. The dose of GCS is reduced by 20-25% per administration every 1-2 days, then by 50% every 1-2 days until complete withdrawal.
		**or**	
		Dexamethasone	16-24 mg/day intravenously depending on the severity of the patient's condition in 2 doses. The maximum dose is used for 3-4 days. The dose of GCS is reduced by 20-25% per dose every 1-2 days, then by 50% every 1-2 days until complete withdrawal.
	4	Anticoagulant drug for parenteral administration, see Appendix 7-1	
	5	In case of fever (t > 38.0 °C) for more than 3 days, antibacterial and antimycotic therapy is prescribed according to indications in accordance with section 5.4.	
**Scheme 3** [1]	1	Remdesivir	Day 1: 200 mg (in 0.9% sodium chloride solution) once intravenously. From the 2nd day: 100 mg intravenously 1 time per day.<br>The general course is no more than 10 days.
		**or**	
		Human immunoglobulin against COVID-19 [3]	1 ml per kg of body weight once intravenously by drip without dilution
	2	Methylprednisolone	125 mg/administration/intravenously every 6-8 hours. The maximum dose is used for 3-4 days. The dose of GCS is reduced by 20-25% per administration every 1-2 days, then by 50% every 1-2 days until complete withdrawal.<br>**or**<br>250 mg/administration/IV once a day for 3-4 days, with a gradual reduction in the dose by 20-25% per administration every 1-2 days, then by 50% every 1-2 days until complete discontinuation.
		**or**	
		Dexamethasone	24 mg/day intravenously depending on the severity of the patient's condition in 2 doses. The maximum dose is used for 3-4 days. The dose of GCS is reduced by 20-25% per dose every 1-2 days, then by 50% every 1-2 days until complete withdrawal.
	3	In case of fever (t > 38.0 °C) for more than 3 days, antibacterial and antimycotic therapy is prescribed according to indications in accordance with section 5.4.	
	4	Anticoagulant drug for parenteral administration, see Appendix 7-1	

**Scheme 4**	1	Remdesivir	Day 1: 200 mg (in 0.9% sodium chloride solution) once intravenously.<br>From the 2nd day: 100 mg intravenously 1 time per day.<br>The general course is no more than 10 days.
		**or**	
		Human immunoglobulin against COVID-19 [3]	1 ml per kg of body weight once intravenously by drip without dilution
	2	Methylprednisolone	60 mg/administration/intravenously every 8 hours, the maximum dose is used for 3-4 days. The dose of GCS is reduced by 20-25% per administration every 1-2 days, then by 50% every 1-2 days until complete withdrawal.
		**or**	
		Dexamethasone	16-24 mg/day intravenously depending on the severity of the patient's condition in 2 doses. The maximum dose is used for 3-4 days. The dose of GCS is reduced by 20-25% per dose every 1-2 days, then by 50% every 1-2 days until complete withdrawal.
	3	Levilimab	162 mg×4 once intravenously by drip over 60 minutes, diluted in 100 ml of 0.9% NaCl solution. If the effect is insufficient, repeat the administration after 12 hours.
		**or**	
		Olokizumab	256 mg intravenously (4 vials of 160 mg/ml, 0.4 ml).<br>256 mg is diluted in 100 ml of 0.9% NaCl solution, administered intravenously by drip over 60 minutes, once
	4	Anticoagulant drug for parenteral administration, see Appendix 7-1	
	5	In case of fever (t > 38.0 °C) for more than 3 days, antibacterial and antimycotic therapy is prescribed according to indications in accordance with section 5.4.	

1 – if there are contraindications to the use and/or the possibility of using genetically engineered biological drugs
2 - if there are contraindications to the use of glucocorticosteroids
3 - patients with a high risk of severe disease (age over 65 years, presence of concomitant diseases (diabetes mellitus, obesity, chronic cardiovascular diseases) during the first 7 days of illness or with a positive laboratory test result for SARS-CoV-2 RNA NAAT
4 - in case of respiratory failure

Recommended regimens for drug prevention of COVID-19

Group	Recommended scheme*
Healthy individuals and individuals at risk (over 60 years of age or with underlying chronic diseases)	**IFN-a (intranasal forms) ****<br>in accordance with the instructions for use of the drug (spray, drops, solution, lyophilisate for solution preparation, gel or ointment)<br><br>OR<br><br>**Umifenovir** 200 mg 2 times a week for 3 weeks
Post-exposure prophylaxis in individuals with single contact with a confirmed case of COVID-19, including healthcare workers	**IFN-a (intranasal forms) ****<br>in accordance with the instructions for use of the drug (spray, drops, solution, lyophilisate for solution preparation, gel or ointment)<br>+<br>**Umifenovir** 200 mg once a day for 10-14 days

* If necessary, preventive courses are repeated

** Pregnant women are prescribed only recombinant IFN-α2b

Instructions
for compliance with infection safety measures
for mobile ambulance teams

1. Medical workers of the mobile ambulance team (MHP) responding to a patient with suspected COVID-19 put on PPE immediately before leaving. Personal protective equipment is changed after each patient.
2. The driver of the mobile ambulance team is also provided with PPE.
3. During the medical evacuation of a patient suspected of having COVID-19 , air disinfection in the ambulance vehicle is provided by closed-type bactericidal irradiators (recirculators) and (or) other devices for disinfecting air and (or) surfaces. All transported persons are provided with a medical mask.
4. In case of contamination of the passenger compartment with biological material from a patient suspected of having COVID-19, the contaminated areas are immediately disinfected.
5. The driver and medical workers of the mobile ambulance teams are required to disinfect shoes and hand PPE in designated areas after transferring the patient to a medical organization providing medical care in inpatient settings, specially created for this contingent of patients (hereinafter referred to as a special medical organization).
6. After completion of the medical evacuation of a patient with suspected COVID-19 to a special medical organization, the ambulance and items used during the medical evacuation are disinfected by disinfectors on the territory of the special medical organization on a specially equipped site with a drain and a pit.
7. If it is impossible to disinfect the interior of an ambulance vehicle using disinfectors on the premises of a special medical organization, disinfection is carried out by the driver and medical workers of the ambulance team.

8. All surfaces in the ambulance vehicle, including the surfaces of medical devices, are disinfected.
9. Surfaces are treated by wiping with a cloth soaked in a disinfectant solution or by spraying with a disinfectant solution.
10. After exposure, the disinfectant solution is washed off with clean water, wiped with a dry cloth, and then aired until the smell of the disinfectant disappears.
11. PPE used in providing medical care and cleaning rags are collected in bags and thrown into special containers for class B waste on the territory of a special medical organization.
12. After disinfection has been carried out in the ambulance vehicle interior, when the ambulance team returns to the ambulance station (substation, department), the air and surfaces of the ambulance vehicle interior are disinfected using open-type ultraviolet bactericidal irradiators, providing a UV dose of at least 25 mJ/cm2 and a total bactericidal radiation flux of at least 100 W for 10 minutes.
13. After completing a call, the driver and medical workers of the ambulance team are required to undergo sanitary treatment, which includes wiping exposed areas of the body with a skin antiseptic.

Algorithm of actions of medical workers providing medical care in outpatient settings, including at home, to patients with acute respiratory viral infections

	Typical cases	Tactics of management
1.	**Contact**<br><br>There was contact with a patient diagnosed with COVID-19.<br>There are no symptoms of acute respiratory viral infection.	• Registration of a sick leave certificate for 14 days;<br>• Home isolation for 14 days;<br>• If symptoms of acute respiratory viral infection or other diseases appear, the patient calls a doctor to the home;<br>• Taking a swab from the nasopharynx and oropharynx on the day of the initial examination by a doctor (as soon as possible); if clinical symptoms of acute respiratory viral infection appear, immediately.
2.	**Mild acute respiratory viral infection** (except for mild acute respiratory viral infections in patients from the risk group).<br><br>The presence of 2 criteria:<br>• SpO2 ≥ 95% (mandatory criterion) ;<br>• T <38 °C;<br>• Respiratory rate ≤ 22.	• Collection of a swab from the nasopharynx and oropharynx on the day of treatment to conduct a laboratory test for COVID-19 with an initial examination by a doctor;<br>• Registration of a sick leave certificate for at least for 14 days.<br>• Home isolation for 14 days;<br>• Daily audio monitoring of the condition, in case if a deterioration in the patient's condition is detected, a doctor should visit the patient at home or call a specialized ambulance team;<br>• Discharge upon recovery without conducting laboratory testing for COVID-19;<br>• If you receive a positive test result for COVID-19 - patient management tactics are determined in accordance with Appendix No. 8 to the order of the Ministry of Health of the Russian Federation dated March 19, 2020 No. 198n (as amended on December 4, 2020 No. [illegible]
3.	**Mild acute respiratory viral infection in a patient belonging to a risk group***	Hospitalization by a specialized mobile ambulance team.

4.	**Moderate or severe acute respiratory viral infection**<br><br>The presence of 2 criteria:<br>• SpO2 < 95% (mandatory criterion);<br>• T ≥ 38 °C;<br>• Respiratory rate > 22.	• Hospitalization by a mobile ambulance team (treatment of patients with moderate acute respiratory viral infections at home is permitted if conditions are available) in a medical organization providing medical care in inpatient settings, determined for this contingent of patients, excluding the possibility of hospitalization in therapeutic, pulmonology departments and anesthesiology and resuscitation departments of other medical organizations.<br>• If the patient refuses hospitalization:<br>- collection of a swab from the nasopharynx and oropharynx on the day of application for laboratory testing for COVID-19 with an initial examination by a doctor;<br>- issuance of a sick leave certificate for at least 14 days;<br>- home isolation for 14 days;<br>- daily audio monitoring of the patient's condition; if a deterioration in the patient's condition is detected, a doctor will visit the patient at home or call an ambulance team to the home;<br>- discharge upon recovery without laboratory testing for COVID-19;<br>- if a positive test result for COVID-19 is received , the patient management tactics are determined in accordance with Appendix No. 8 to the order of the Ministry of Health of the Russian Federation dated March 19, 2020 No. 198n (as amended on December 4, 2020 No. 1288n)

*Persons over 65 years of age; persons with chronic diseases of the bronchopulmonary, cardiovascular, endocrine systems; systemic connective tissue diseases; chronic kidney disease; oncological diseases; immunodeficiencies; motor neuron diseases; liver cirrhosis; chronic inflammatory bowel diseases.

List of temporary guidelines for physicians

1) Features of clinical manifestations and treatment of the disease caused by the new coronavirus infection (COVID-19) in children
2) Organization of medical care for pregnant women, women in labor, and women in labor and newborns with the new coronavirus infection COVID-19
3) Medical rehabilitation for new coronavirus infection (COVID-19)
4) Drug therapy of acute respiratory viral infections (ARVI) in outpatient settings practice during the COVID-19 epidemic
5) Methodological recommendations for the organization of preventive medical examinations and medical check-ups in the context of the continuing risks of the spread of the new coronavirus infection (COVID-19)
6) Guidelines for coding and choosing the main state in statistics incidence and underlying cause in COVID-19 related mortality statistics
7) Procedure for vaccinating the adult population against COVID-19
8) Clinical guidelines of the Federation of Anesthesiologists and Resuscitators “Application non-invasive ventilation"
9) Clinical guidelines of the Federation of Anesthesiologists and Resuscitators "Diagnostics and intensive care of acute respiratory distress syndrome"
10) Guidelines of the Federation of Anesthesiologists and Resuscitators on Anesthesiological and -Resuscitation Management of Patients with COVID-19
11) Recommendations for psychological support of the activities of heads of medical organizations and their structural divisions in the context of providing medical care to patients with COVID-19
12) Recommendations for the prevention of psychological distress in health workers during the COVID-19 pandemic
13) Recommendations for healthcare workers on coping with stress during the COVID-19 pandemic

LIST OF ABBREVIATIONS USED

ACE - angiotensin-converting enzyme
ATP - adenosine triphosphate
APTT - activated partial thromboplastin time
DMARD - basic anti-inflammatory drug
i/v - intravenously
ULN - upper limit of normal
WHO - World Health Organization
GIBP - genetically engineered biological preparation
GCS - glucocorticosteroids
HLH - hemophagocytic lymphohistocytosis
DN - respiratory failure
GIT - gastrointestinal tract
ALV - artificial lung ventilation
IIRZ - immune-mediated inflammatory rheumatic diseases
IFN - interferon
PRC - People's Republic of China
CT - computed tomography
NAAT - nucleic acid amplification method
IU - International Units of Measurement
INR - International Normalized Ratio
MP - methylprednisolone
NIVL - non-invasive ventilation
NSAID - non-steroidal anti-inflammatory drug
LMWH - low molecular weight heparin
UFH - unfractionated heparin
OC - chest organs
ARF - acute respiratory failure
ARI - acute respiratory viral infection
ARI - acute respiratory infection
ARDS - acute respiratory distress syndrome
ORIT - intensive care unit
DOAC - oral anticoagulants
PCR - polymerase chain reaction
PEP - oral enteral nutrition
RNA - ribonucleic acid
SAM - macrophage activation syndrome
FFP - fresh frozen donor plasma
PPE - personal protective equipment
RPE - personal respiratory protective equipment
GFR - glomerular filtration rate
SMP – emergency medical care
ESR – erythrocyte sedimentation rate

CRP – C-reactive protein
SS – septic shock
DVT – deep vein thrombosis
TIB – transport insulating box
SARI - severe acute respiratory infection
SARS – severe acute respiratory syndrome
PE – pulmonary embolism
UVBI – ultraviolet germicidal radiation
TNF-α – tumor necrosis factor alpha
CKD – chronic kidney disease
COPD - Chronic Obstructive Pulmonary Disease
ECG – electrocardiography
ECMO – extracorporeal membrane oxygenation
EP – enteral nutrition
COVID-19 is an infection caused by the new coronavirus SARS-CoV-2
CPAP – continuous positive air pressure ventilation
FiO_2 – concentration of oxygen in the breathing mixture
IgM – class M immunoglobulins
IgG – immunoglobulins of class G
IgA – immunoglobulin class A
MDR – multiple drug resistant strains
MERS – Middle East Respiratory Syndrome
MERS-CoV – the coronavirus that caused the Middle East respiratory syndrome outbreak
MRSA – methicillin-resistant Staphylococcus aureus
NT-proBNP – brain natriuretic peptide
PaCO2 – partial pressure of carbon dioxide in the blood
PaO2 – partial pressure of oxygen in the blood
PEEP – continuous positive airway pressure (Positive End Expiratory Pressure)
PvO_2 – oxygen tension in venous blood
SARS (Severe Acute Respiratory Syndrome)
SARS-CoV is the coronavirus that caused the outbreak of severe acute respiratory syndrome
SARS-CoV-2 is a new coronavirus that caused an outbreak of infection in 2019-2020.
SOFA – SOFA (**Sequential** Organ Failure Assessment) scale for organ failure assessment
failure, risk of mortality and sepsis
SpO2 – the level of oxygen saturation in the blood (saturation)
T – body temperature
Vt – tidal volume (ml)/body weight (kg) of the patient
XDR – strains with extreme resistance

TEAM OF AUTHORS

Avdeev Sergey Nikolaevich - Chief Pulmonologist, Head of the Pulmonology Department of the Federal State Autonomous Educational Institution of Higher Education I.M. Sechenov First Moscow State Medical University of the Ministry of Health of the Russian Federation (Sechenov University), Deputy Director of the Federal State Budgetary Institution "Research Institute of Pulmonology of the Federal Medical and Biological Agency"

Adamyan Leila Vladimirovna - chief freelance specialist in gynecology, deputy director for scientific work of the Federal State Budgetary Institution "National Medical Research Center for Obstetrics, Gynecology and Perinatology named after Academician V.I. Kulakov" of the Ministry of Health of the Russian Federation

Alekseeva Ekaterina Iosifovna - chief freelance pediatric rheumatologist, head of the rheumatology department of the Federal State Autonomous Institution "National Medical Research Center of Children's Health" of the Ministry of Health of Russia, director of the Clinical Institute of Children's Health named after N.F. Filatov of the Federal State Autonomous Educational Institution of Higher Education I.M. Sechenov First Moscow State Medical University of the Ministry of Health of Russia (Sechenov University)

Bagnenko Sergey Fedorovich - Rector of the Federal State Budgetary Educational Institution of Higher Education "First Saint Petersburg State Medical University named after Academician I.P. Pavlov" of the Ministry of Health of the Russian Federation, Chief Specialist in Emergency Medical Care of the Ministry of Health of the Russian Federation

Baranov Aleksandr Aleksandrovich – chief freelance specialist pediatrician, scientific director of the Federal State Autonomous Scientific Institution “National Medical Research Center for Children’s Health” of the Ministry of Health of Russia

Baranova Natalya Nikolaevna - Chief Physician of the Center for Medical Evacuation and Emergency Medical Care of the Federal State Budgetary Institution "All-Russian Center for Disaster Medicine "Zashchita"" of the Federal Medical and Biological Agency of Russia

Belevsky Andrey Stanislavovich - Head of the Department of Pulmonology of the Federal State Autonomous Educational Institution of Higher Education "Russian National Research Medical University named after N.I. Pirogov" of the Ministry of Health of the Russian Federation, Chief Pulmonologist of the Moscow Department of Health

Belkin Andrey Avgustovich - Chief Specialist in Medical Rehabilitation of the Ministry of Health of the Russian Federation in the Ural Federal District, Professor of the Department of Physical and Rehabilitation Medicine of the Federal State Budgetary Educational Institution of Higher Education "Ural State Medical University" of the Ministry of Health of the Russian Federation, Director of the Clinical Institute of the Brain LLC

Beloborodov Vladimir Borisovich - Head of the Department of Infectious Diseases of the Federal State Budgetary Educational Institution of Additional Professional Education "Russian Medical Academy of Continuous Professional Education" of the Ministry of Health of Russia

Bodrova Rezeda Akhmetovna - Head of the Department of Rehabilitation and Sports Medicine of the Kazan State Medical Academy, a branch of the Federal State Budgetary Educational Institution of Additional Professional Education "Russian Medical Academy of Postgraduate Education" of the Ministry of Health of the Russian Federation

Builova Tatyana Valentinovna - chief freelance specialist in medical rehabilitation of the Ministry of Health of Russia in the Volga Federal District, director of the Institute of Rehabilitation 221

and human health of the Federal State Autonomous Educational Institution of Higher Education "National Research Nizhny Novgorod State University named after N.I. Lobachevsky"

Boyko Elena Alekseevna - Head of the Department of the Coordination and Analytical Center for Ensuring Chemical and Biological Safety of the Federal State Budgetary Institution "Center for Strategic Planning and Management of Medical and Biological Health Risks" of the Federal Medical and Biological Agency of Russia

Briko Nikolay Ivanovich - Head of the Department of Epidemiology and Evidence-Based Medicine of

the Federal State Autonomous Educational Institution of Higher Education I.M. Sechenov First Moscow State Medical University of the Ministry of Health of the Russian Federation (Sechenov University)

Brusina Elena Borisovna - Chief Epidemiologist of the Kemerovo Region, Head of the Department of Epidemiology, Infectious Diseases and Dermatovenereology of the Federal State Budgetary Educational Institution of Higher Education "Kemerovo State Medical University" of the Ministry of Health of Russia

Tatyana Vladimirovna Vavilova – Chief Specialist in Clinical Laboratory Diagnostics of the Russian Ministry of Health, Head of the Department of Laboratory Medicine and Genetics of the Institute of Medical Education of the Federal State Budgetary Institution "V.A. Almazov National Medical Research Center" of the Russian Ministry of Health

Vaysman David Shunevich - Chief Researcher of the Department of Public Health and Demography of the Federal State Budgetary Institution "Central Research Institute for Health Organization and Informatization" of the Ministry of Health of the Russian Federation, Doctor of Medical Sciences

Vasilyeva Elena Yuryevna - Chief Physician of the State Budgetary Healthcare Institution of Moscow "City Clinical Hospital named after I.V. Davydovsky of the Moscow City Healthcare Department", Chief Cardiologist of the City of Moscow

Vasilyeva Irina Anatolyevna - Chief freelance specialist phthisiologist of the Ministry of Health of Russia, Director of the Federal State Budgetary Institution "National Medical Research Center for Phthisiopulmonology and Infectious Diseases" of the Ministry of Health of Russia

Vasilyeva Natalia Vsevolodovna - Chief Specialist in Clinical Microbiology and Antimicrobial Resistance of the Ministry of Health of the Russian Federation for the Northwestern Federal District, Director of the Research Institute of Medical Mycology named after P.N. Kashkin, Head of the Department of Medical Microbiology of the Federal State Budgetary Educational Institution of Higher Education "North-West State Medical University named after I.I. Mechnikov" of the Ministry of Health of the Russian Federation

Veselova Elena Igorevna - research fellow of the Department of Infectious Pathology of the Federal State Budgetary Institution "National Medical Research Center for Phthisiopulmonology and Infectious Diseases" of the Ministry of Health of the Russian Federation

Vishneva Elena Aleksandrovna - Deputy Head of Science at the Research Institute of Pediatrics and Health Protection of the Federal State Budgetary Institution of Healthcare of the Central Clinical Hospital of the Russian Academy of Sciences

Volchkova Elena Vasilievna - Head of the Department of Infectious Diseases of the Federal State Autonomous Educational Institution of Higher Education I.M. Sechenov First Moscow State Medical University of the Ministry of Health of the Russian Federation (Sechenov University)

Volchenkov Grigory Vasilievich - Chief Physician of the State Budgetary Healthcare Institution of the Vladimir Region, Center for Specialized Phthisiopulmonological Care

Gaponova Tatyana Vladimirovna - Chief freelance specialist transfusiologist of the Ministry of Health of Russia, Deputy Director General for transfusiology of the Federal State Budgetary Institution "National Medical Research Center of Hematology" of the Ministry of Health of Russia

Godkov Mikhail Andreevich – Head of the Laboratory Diagnostics Department of the State Budgetary Healthcare Institution "N.V. Sklifosovsky Research Institute of Emergency Care" of the Moscow City Healthcare Department

Goncharov Sergey Fedorovich - Chief Specialist in Disaster Medicine of the Ministry of Health of Russia, Director of the Federal State Budgetary Institution "All-Russian Center for Disaster Medicine "Zashchita"" FMBA of Russia

Gautier Sergey Vladimirovich – Chief Transplantologist of the Ministry of Health of the Russian Federation, Director of the Federal State Budgetary Institution "NMITs TIO named after Academician V.I. Shumakov" of the Ministry of Health of the Russian Federation, Head of the Department of Transplantology and Artificial Organs of the First Moscow State Medical University named after I.M. Sechenov

Grechko Andrey Vyacheslavovich - Director of the Federal State Budgetary Scientific Institution

"Federal Scientific and Clinical Center for Resuscitation and Rehabilitation"

***Danilenko Darya Mikhailovna* –** Deputy Director for Research of the Federal State Budgetary Institution “A.A. Smorodintsev Research Institute of Influenza” of the Ministry of Health of the Russian Federation

Dmitriev Aleksandr Sergeevich - infectious disease specialist at the Federal State Budgetary Institution "National Medical Research Center for Phthisiopulmonology and Infectious Diseases" of the Ministry of Health of Russia, chief freelance specialist in infectious diseases of the Ministry of Health of Russia in the Volga Federal District

Dolgushina Natalia Vitalievna - Deputy Director - Head of the Department of Organization of Scientific Activities of the Federal State Budgetary Institution "National Medical Research Center for Obstetrics, Gynecology and Perinatology named after Academician V.I. Kulakov" of the Ministry of Health of Russia, Chief Specialist of the Ministry of Health of Russia on Women's Reproductive Health

***Oksana Mikhailovna Drapkina* –** chief freelance specialist in therapy and general medical prevention, director of the Federal State Institution “National Medical Research Center for Preventive Medicine” of the Ministry of Health of Russia

Dyagtyarev Dmitry Nikolaevich - Deputy Director for Research of the Federal State Budgetary Institution "National Medical Research Center for Obstetrics, Gynecology and Perinatology named after Academician V.I. Kulakov" of the Ministry of Health of the Russian Federation

Zhukova Olga Valentinovna - chief freelance specialist in dermatovenereology and cosmetology of the Ministry of Health of Russia in the Central Federal District, chief physician of the State Budgetary Healthcare Institution "Moscow Scientific and Practical Center for Dermatovenereology and Cosmetology of the Moscow City Health Department", acting head of the department of skin and venereal diseases of the Federal State Autonomous Educational Institution of Higher Education "Peoples' Friendship University of Russia" of the Ministry of Education and Science of Russia

***Zhuravel Sergey Vladimirovich* –** Head of the Scientific Department of Anesthesiology of the State Budgetary Healthcare Institution “N.V. Sklifosovsky Research Institute of Emergency Care” of the Moscow City Healthcare Department

***Zabozlaev Fedor Georgievich* -** professor of the department of clinical laboratory diagnostics and pathological anatomy of the Federal State Budgetary Educational Institution Academy of Postgraduate Education of the Federal Scientific and Clinical Center of the Federal Medical and Biological Agency of Russia, head of the pathological anatomical department of the Federal Scientific and Clinical Center of the Federal Medical and Biological Agency of Russia, vice-president of the Russian Society of Pathologists.

Igor Borisovich Zabolotskikh - Head of the Department of Anesthesiology, Resuscitation and Transfusiology of the Federal State Budgetary Educational Institution of Higher Education "Kuban State Medical University" of the Ministry of Health of the Russian Federation

***Zagrebneva Alena Igorevna* -** Associate Professor of the Department of General Therapy of the Faculty of Advanced Medical Studies of the Federal State Autonomous Educational Institution of Higher Education "Russian National Research Medical University named after N.I. Pirogov" of the Ministry of Health of the Russian Federation, Chief Rheumatologist of the Moscow Department of Health

***Zairatyants Oleg Vadimovich* -** Chief Freelance Pathologist of the Moscow City Health Department, Chief Freelance Pathologist-Expert of Roszdravnadzor for the Central Federal District, Head of the Department of Pathological Anatomy of the Federal State Budgetary Educational Institution of Higher Education "A.I. Evdokimov Moscow State Medical University" of the Ministry of Health of Russia, Vice President of the Russian Society of Pathologists, Chairman of the Moscow Society of Pathologists

Zaitsev Andrey Alekseevich - Chief Pulmonologist of the Ministry of Defense of the Russian Federation, Chief Pulmonologist of the Federal State Budgetary Institution "Main Military Clinical Hospital named after Academician N.N. Burdenko" of the Ministry of Defense of the Russian Federation

Zyuzya Yulia Rashidovna - pathologist of the Federal State Budgetary Institution "National Medical Research Center for Phthisiopulmonology and Infectious Diseases" of the Ministry of Health of the Russian

Federation

Ivanov Dmitry Olegovich - chief freelance specialist neonatologist of the Ministry of Health of Russia, rector of the Federal State Budgetary Institution of Higher Education St. Petersburg State Pediatric Medical University

Ivanov Sergey Anatolyevich - Director of the A.F. Tsyb MRRC - branch of the Federal State Budgetary Institution "NMIC Radiology" of the Ministry of Health of Russia, chief freelance oncologist of the Ministry of Health of Russia for the Central Federal District

Ivanova Galina Evgenievna - Chief Specialist in Medical Rehabilitation of the Ministry of Health of the Russian Federation, Head of the Department of Medical Rehabilitation of the Faculty of Continuing Professional Education of the Federal State Autonomous Educational Institution of Higher Education "Russian National Research Medical University named after N.I. Pirogov" of the Ministry of Health of the Russian Federation, Head of the Department of Medical Rehabilitation of the Federal State Budgetary Institution FCMS FMBA of Russia

Vladimir Trofimovich Ivashkin - Chief Gastroenterologist of the Russian Ministry of Health, Head of the Department of Propaedeutics of Internal Diseases, Gastroenterology and Hepatology of the N.V. Sklifosovsky Institute of Clinical Medicine, Director of the Clinic of Propaedeutics of Internal Diseases, Gastroenterology and Hepatology named after V.Kh. Vasilenko of the Federal State Autonomous Educational Institution of Higher Education I.M. Sechenov First Moscow State Medical University of the Russian Ministry of Health (Sechenov University)

Isaeva Irina Vladimirovna – Deputy Chief of Staff of the All-Russian Disaster Medicine Service of the Federal State Budgetary Institution “All-Russian Center for Disaster Medicine “Zashchita”” of the Federal Medical and Biological Agency of Russia

Kaminsky Grigory Dmitrievich - Head of the Department of Infectious Pathology of the Federal State Budgetary Institution "National Medical Research Center for Phthisiopulmonology and Infectious Diseases" of the Ministry of Health of the Russian Federation

Kaprin Andrey Dmitrievich - General Director of the Federal State Budgetary Institution "National Medical Research Center of Radiology" of the Ministry of Health of Russia, Chief Freelance Oncologist of the Ministry of Health of Russia for the Central, Volga and North Caucasian Federal Districts

Karpov Oleg Eduardovich - General Director of the Federal State Budgetary Institution "National Medical and Surgical Center named after N.I. Pirogov" of the Ministry of Health of the Russian Federation

Kachanova Natalia Aleksandrovna - Head of the Department of Organizational and Methodological Work of the Center for Medical Evacuation and Emergency Medical Care of the Federal State Budgetary Institution "All-Russian Center for Disaster Medicine "Zashchita"" of the Federal Medical and Biological Agency of Russia

Kirov Mikhail Yuryevich - Head of the Department of Anesthesiology and Resuscitation of the Federal State Budgetary Educational Institution of Higher Education "Northern State Medical University" of the Ministry of Health of the Russian Federation

Klimov Vladimir Anatolyevich - Head of the Medical Care Organization Service of the Federal State Budgetary Institution "National Medical Research Center for Obstetrics, Gynecology and Perinatology named after Academician V.I. Kulakov" of the Ministry of Health of the Russian Federation

Klimko Nikolay Nikolaevich - Head of the Department of Clinical Mycology, Allergology and Immunology of the Federal State Budgetary Educational Institution of Higher Education "North-West State Medical University named after I.I. Mechnikov" of the Ministry of Health of the Russian Federation

Klyuev Oleg Igorevich - Head of the Department of Anesthesiology and Resuscitation with Resuscitation and Intensive Care Units of the Federal State Budgetary Institution "National Medical Research Center for Phthisiopulmonology and Infectious Diseases" of the Ministry of Health of the Russian Federation

Olga Vasilievna Kovalishena - Chief Epidemiologist of the Volga Federal District, Head of the Department of Epidemiology, Microbiology and Evidence-Based Medicine of the Federal State Budgetary Educational Institution of Higher Education "Volga Research Medical University" of the Ministry of Health of the Russian Federation

Kozlov Roman Sergeevich - Rector of the Federal State Budgetary Educational Institution of Higher Education "Smolensk State Medical University", Chief Specialist in Clinical Microbiology and Antimicrobial Resistance of the Ministry of Health of the Russian Federation

Vladimir Viktorovich Krylov – Chief Neurosurgeon of the Ministry of Health, Director of the University Clinic of the A. I. Evdokimov Moscow State Medical University, Head of the Department of Neurosurgery and Neuroreanimation of the A. I. Evdokimov Moscow State Medical University, Chief Researcher of the Department of Neurosurgery of the State Budgetary Healthcare Institution "N. V. Sklifosovsky Research Institute of Emergency Care" of the Moscow City Health Department

Kuzovlev Artem Nikolaevich - Deputy Director-Head of the Research Institute of General Resuscitation named after V.A. Negovskiy of the Federal State Budgetary Scientific Institution "Federal Scientific and Clinical Center for Resuscitation and Rehabilitation"

Lebedinsky Konstantin Mikhailovich - Head of the Department of Anesthesiology and Resuscitation named after V.L. Vanevsky of the Federal State Budgetary Educational Institution of Higher Education "North-West State Medical University named after I.I. Mechnikov" of the Ministry of Health of the Russian Federation

Lioznov Dmitry Anatolyevich - Director of the Federal State Budgetary Institution "A.A. Smorodintsev Research Institute of Influenza" of the Ministry of Health of the Russian Federation, 225

Chief Specialist in Infectious Diseases of the Russian Ministry of Health in the Northwestern Federal District

Lobzin Yuri Vladimirovich - Chief Specialist of the Ministry of Health of Russia and the Federal Medical and Biological Agency of Russia for Infectious Diseases in Children, President of the Federal State Budgetary Institution "Children's Scientific and Clinical Center for Infectious Diseases of the Federal Medical and Biological Agency"

Lomakin Nikita Valerievich - Head of the Emergency Cardiology Department of the Federal State Budgetary Institution "Central Clinical Hospital with a Polyclinic" of the Presidential Executive Office of the

Russian Federation, Chief Cardiologist of the Presidential Executive Office of the Russian Federation.

Galina Viktorovna Lukina **—** Head of the Rheumatology Research Department, Head of the Moscow City Rheumatology Center of the State Budgetary Healthcare Institution “Moscow Clinical Research Center named after A.S. Loginov” of the Moscow City Healthcare Department, Leading Researcher of the Laboratory for the Study of Comorbid Infections and Monitoring the Safety of Drug Therapy of the Federal State Budgetary Scientific Institution “V.A. Nasonova Research Institute of Rheumatology”

Lysenko Maryana Anatolyevna **-** Chief Physician of the State Budgetary Healthcare Institution "City Clinical Hospital No. 52 of the Moscow City Healthcare Department"

Mazus Aleksey Izrailevich - chief freelance specialist in the problems of diagnostics and treatment of HIV infection of the Ministry of Health of Russia and the Department of Health of the city of Moscow, head of the Moscow city center for the prevention and control of AIDS of the Department of Health of the city of Moscow

Maleev Viktor Vasilyevich **–** Advisor to the Director for Scientific Work of the Federal Budgetary Scientific Institution Central Research Institute of Epidemiology of the Federal Service for Surveillance on Consumer Rights Protection and Human Wellbeing

Malinnikova Elena Yuryevna **-** Head of the Department of Virology of the Federal State Budgetary Educational Institution of Additional Professional Education "Russian Medical Academy of Continuous Professional Education" of the Ministry of Health of Russia

Mamonova Nina Alekseevna **–** researcher at the Laboratory of Genetic Technologies and Translational Research of the Federal State Budgetary Institution “National Medical Research Center for Phthisiopulmonology and Infectious Diseases” of the Ministry of Health of the Russian Federation

Melnikova Elena Valentinovna **-** chief freelance specialist in medical rehabilitation of the Ministry of Health of Russia in the Northwestern Federal District, deputy chief physician - head of the regional vascular center of the St. Petersburg state budgetary health care institution "City Hospital No. 26", professor of the department of physical methods of treatment and sports medicine of the faculty of postgraduate education of the federal state budgetary educational institution of higher education "First St. Petersburg State Medical University named after Academician I.P. Pavlov"

Mitkov Vladimir Vyacheslavovich - Head of the Department of Ultrasound Diagnostics of the Federal State Budgetary Educational Institution of Continuous Professional Education "Russian Medical Academy of Continuous Professional Education" of the Ministry of Health of Russia

Mishina Irina Evgenievna **-** Vice-Rector for Academic Affairs, Head of the Department of Therapy of the Federal State Budgetary Institution of Healthcare "Ivanovo State Medical Academy" of the Ministry of Health of Russia

Mokrysheva Natalia Georgievna **-** Director of the Federal State Budgetary Institution "National Medical Research Center of Endocrinology" of the Ministry of Health of Russia

Molchanov Igor Vladimirovich **-** Chief Specialist of the Ministry of Health of Russia in Anesthesiology and Resuscitation, Head of the Department of Anesthesiology and Resuscitation of the Federal State Budgetary Educational Institution of Additional Professional Education "Russian Medical Academy of Continuous Professional Education" of the Ministry of Health of Russia

Morozov Dmitry Anatolyevich **-** Head of the Department of Pediatric Surgery and Urology-Andrology named after Professor L.P. Alexandrov of the Federal State Autonomous Educational Institution of Higher Education I.M. Sechenov First Moscow State Medical University of the Ministry of Health of the Russian Federation (Sechenov University)

Morozov Sergey Pavlovich - Chief Specialist in Radiation and Instrumental Diagnostics of the Ministry of Health of Russia for the Central Federal District of the Russian Federation, Director of the State Budgetary Healthcare Institution "Scientific and Practical Clinical Center for Diagnostics and Telemedicine Technologies of the Moscow Department of Healthcare"

Nasonov Evgeny Lvovich **-** Chief Freelance Specialist Rheumatologist, Scientific Director of the Federal State Budgetary Scientific Institution "V.A. Nasonova Research Institute of Rheumatology" of the Ministry of

Health of the Russian Federation

Nikitin Igor Gennadievich - Head of the Department of Hospital Therapy No. 2 of the Medical Faculty of the Federal State Autonomous Educational Institution of Higher Education "Russian National Research Medical University named after N.I. Pirogov", Director of the Federal State Autonomous Institution "Medical - Rehabilitation Center" of the Ministry of Health of Russia

Nikiforov Vladimir Vladimirovich - Head of the Department of Infectious Diseases and Epidemiology of the Federal State Autonomous Educational Institution of Higher Education "N.I. Pirogov Russian National Research Medical University" of the Ministry of Health of the Russian Federation

Nikolaeva Anastasia Vladimirovna - Chief Physician of the Federal State Budgetary Institution "National Medical Research Center for Obstetrics, Gynecology and Perinatology named after Academician V.I. Kulakov" of the Ministry of Health of the Russian Federation

Omelyanovsky Vitaly Vladimirovich - General Director of the Federal State Budgetary Institution "Center for Expertise and Quality Control of Medical Care" of the Ministry of Health of Russia

Elizaveta Pavlovna Panchenko – Head of the Department of Clinical Problems of Atherothrombosis, A.L. Myasnikov Institute of Cardiology, Federal State Budgetary Institution "National Medical Research Center of Cardiology" of the Ministry of Health of Russia

Panova Anna Evgenievna - Head of the Laboratory Diagnostics Department of the Federal State Budgetary Institution "National Medical Research Center for Phthisiopulmonology and Infectious Diseases" of the Ministry of Health of the Russian Federation

Pyregov Aleksey Viktorovich - Head of the Department of Anesthesiology and Resuscitation of the Federal State Budgetary Institution "National Medical Research Center of Obstetrics, Gynecology and Perinatology named after Academician V.I. Kulakov of the Ministry of Health of the Russian Federation

Petrova Marina Vladimirovna - Deputy Director for Scientific and Clinical Activities of the Federal State Budgetary Scientific Institution "Federal Scientific and Clinical Center for Resuscitation and Rehabilitation"

Petrov Vladimir Aleksandrovich - Head of the Scientific and Educational Department of the A.F. Tsyb MRRC - branch of the Federal State Budgetary Institution "NMITs of Radiology" of the Ministry of Health of the Russian Federation, Head of the Department of Infectious Diseases, Public Health and Healthcare of the Obninsk Institute of Atomic Energy - branch of the Federal State Autonomous Educational Institution of Higher Education "National Research Nuclear University MEPhI"

Pimenov Nikolay Nikolaevich - Head of the Laboratory of Epidemiology of Infectious Diseases of the Federal State Budgetary Institution "National Medical Research Center for Phthisiopulmonology and Infectious Diseases" of the Ministry of Health of the Russian Federation

Ploskireva Antonina Aleksandrovna - Deputy Director for Clinical Work of the Federal Budgetary Scientific Institution Central Research Institute of Epidemiology of the Federal Service for Surveillance on Consumer Rights Protection and Human Wellbeing

Poluektova Elena Aleksandrovna - Professor of the Department of Propaedeutics of Internal Medicine, Gastroenterology and Hepatology of the N.V. Sklifosovsky Institute of Clinical Medicine of the Federal State Autonomous Educational Institution of Higher Education I.M. Sechenov First Moscow State Medical University of the Ministry of Health of the Russian Federation (Sechenov University)

Potekaev Nikolay Nikolaevich - chief freelance specialist in dermatovenereology and cosmetology of the Ministry of Health of the Russian Federation and the Moscow Department of Health, director of the State Budgetary Healthcare Institution "Moscow Scientific and Practical Center for Dermatovenereology and Cosmetology of the Moscow Department of Health", head of the Department of Skin Diseases and Cosmetology of the Faculty of Continuing Professional Education of the Federal State Autonomous Educational Institution of Higher Education "N.I. Pirogov Russian National Research Medical University" of the Ministry of Health of the Russian Federation

Protsenko Denis Nikolaevich - Chief Specialist in Anesthesiology and Resuscitation of the Moscow City

Health Department, Chief Physician of the State Budgetary Healthcare Institution City Clinical Hospital No. 40 of the Moscow Health Department, Associate Professor of the Department of Anesthesiology and Resuscitation of the Federal State Autonomous Educational Institution of Higher Education "N.I. Pirogov Russian National Research Medical University" of the Ministry of Health of the Russian Federation

Pushkar Dmitry Yuryevich - Chief Urologist of the Ministry of Health of Russia, Chief Urologist of the Moscow City Health Department, Head of the Urology Department of the Moscow State University of Medicine and Dentistry named after A.I. Evdokimov

Pshenichnaya Natalya Yuryevna - Deputy Director for Clinical and Analytical Work of the Federal Budgetary Scientific Institution Central Research Institute of Epidemiology of the Federal Service for Surveillance on Consumer Rights Protection and Human Wellbeing

Revishvili Amiran Shotaevich - Chief Surgeon of the Ministry of Health of Russia, Director of the Federal State Budgetary Institution "National Medical Research Center of Surgery named after A.V. Vishnevsky" of the Ministry of Health of Russia

Rodin Alexander Anatolyevich - Head of the Department of Radiation Diagnostics of the Federal State Budgetary Institution "National Medical Research Center for Phthisiopulmonology and Infectious Diseases" of the Ministry of Health of the Russian Federation

Romanov Vladimir Vasilievich - Deputy Head of the Federal Medical and Biological Agency

Roshal Leonid Mikhailovich - President of the State Budgetary Healthcare Institution of Moscow "Research Institute of Emergency Children's Surgery and Traumatology" of the Moscow Department of Health; President of the National Medical Chamber.

Russkikh Anastasia Evgenievna - research fellow of the Department of Differential Diagnostics and Treatment of Tuberculosis and Combined Infections of the Federal State Budgetary Institution "National Medical Research Center for Phthisiopulmonology and Infectious Diseases" of the Ministry of Health of the Russian Federation

Savchenko Valery Grigorievich - Chief freelance hematologist of the Ministry of Health of Russia, General Director of the Federal State Budgetary Institution "National Medical Research Center of Hematology" of the Ministry of Health of Russia

Samoylova Anastasia Gennadyevna - First Deputy Director of the Federal State Budgetary Institution "National Medical Research Center for Phthisiopulmonology and Infectious Diseases" of the Ministry of Health of the Russian Federation

Sinitsyn Valentin Evgenievich - Head of the Course of Radiation Diagnostics and Radiation Therapy of the Faculty of Fundamental Medicine of the Moscow State University named after M.V. Lomonosov, Professor of the Department of Roentgenology and Radiology of the Federal State Budgetary Educational Institution of Additional Professional Education "Russian Medical Academy of Continuous Professional Education" of the Ministry of Health of the Russian Federation

Vladimir Ivanovich Starodubov - Chief Freelance Specialist in Medical Statistics of the Russian Ministry of Health, Scientific Director of the Federal State Budgetary Institution "Central Research Institute for Health Organization and Informatization", Academician-Secretary of the Department of Medical Sciences of the Russian Academy of Sciences

Stepanenko Sergey Mikhailovich - chief freelance pediatric specialist anesthesiologist-resuscitator, professor of the Department of Pediatric Surgery of the Pediatric Faculty of the Federal State Autonomous Educational Institution of Higher Education "Russian National Research Medical University named after N.I. Pirogov" of the Ministry of Health of the Russian Federation

Suranova Tatyana Grigoryevna - Deputy Head of the Department for the Organization of Medical Protection of the Population from Extreme Factors of the Headquarters of the All-Russian Center for Disaster Medicine "Zashchita" of the Federal Medical and Biological Agency of Russia

Su.Khorukikh Olga Aleksandrovna - Head of the Department of Medical Support for Standardization of the Federal State Budgetary Institution "Center for Expertise and Quality Control of Medical Care" of the

Ministry of Health of Russia

Sychev Dmitry Alekseevich - Head of the Department of Clinical Pharmacology and Therapy named after Academician B.E. Votchal, Federal State Budgetary Educational Institution of Higher Professional Education "Russian Medical Academy of Continuous Professional Education" of the Ministry of Health of the Russian Federation

Tkacheva Olga Nikolaevna - Chief freelance geriatrician of the Ministry of Health of Russia, director of the separate structural unit "Russian Gerontological Scientific Clinical Center" of the Federal State Autonomous Educational Institution of Higher Education "Russian National Research Medical University named after N.I. Pirogov" of the Ministry of Health of Russia

Irina Nikolaevna Tragira – Head of the Center for Infectious Diseases of the Federal State Budgetary Institution “National Medical Research Center for Phthisiopulmonology and Infectious Diseases” of the Ministry of Health of Russia

Tyurin Igor Evgenievich - chief freelance specialist in radiation and instrumental diagnostics, head of the department of radiology and radiology of the Federal State Budgetary Educational Institution of Additional Professional Education "Russian Medical Academy of Continuous Professional Education" of the Ministry of Health of Russia

Urtikov Aleksandr Valerievich - research fellow of the laboratory of epidemiology of infectious diseases of the Federal State Budgetary Institution "National Medical Research Center for Phthisiopulmonology and Infectious Diseases" of the Ministry of Health of the Russian Federation

Uskov Aleksandr Nikolaevich - Director of the Federal State Budgetary Institution "Children's Scientific and Clinical Center for Infectious Diseases of the Federal Medical and Biological Agency"

Falaleeva Natalia Aleksandrovna - Head of the Department of Drug Treatment of Malignant Neoplasms of the A.F. Tsyb Medical Research Center - branch of the Federal State Budgetary Institution "NMITs of Radiology" of the Ministry of Health of the Russian Federation

Fomicheva Anastasia Aleksandrovna - epidemiologist at the Center for Infectious Diseases, junior researcher at the Laboratory of Epidemiology of Infectious Diseases of the Federal State Budgetary Institution "National Medical Research Center for Phthisiopulmonology and Infectious Diseases" of the Ministry of Health of Russia

Fomina Darya Sergeevna - Chief Allergist-Immunologist of the Moscow City Health Department, Head of the Allergology and Immunology Center of the Moscow City State Budgetary Healthcare Institution "City Clinical Hospital No. 52 of the Moscow City Health Department"

Frank Georgy Avraamovich - Chief Pathologist of the Ministry of Health of Russia, Head of the Department of Pathological Anatomy of the Federal State Budgetary Educational Institution of Additional Professional Education "Russian Medical Academy of Continuous Professional Education" of the Ministry of Health of Russia

Khailova Zhanna Vladimirovna - Deputy Director for Organizational and Methodological Work of the A.F. Tsyb MRRC - branch of the Federal State Budgetary Institution "NMITS Radiology" of the Ministry of Health of Russia, Head of the Center for Coordination of the Activities of Regional Institutions in the Field of Oncology and Radiology of the Federal State Budgetary Institution "NMITS Radiology" of the Ministry of Health of Russia

Tsarenko Sergey Vasilevich - Deputy Chief Physician for Anesthesiology and Resuscitation of the State Budgetary Healthcare Institution of the City of Moscow "City Clinical Hospital No. 52 of the Moscow City Healthcare Department"

Tsinzerling Vsevolod Aleksandrovich - Head of the Research Institute of Pathomorphology of the Federal State Budgetary Institution "V.A. Almazov National Medical Research Center" of the Ministry of Health of the Russian Federation

Tsykunov Mikhail Borisovich - Head of the Department of Medical Rehabilitation of the Federal State Budgetary Institution "National Medical Research Center of Traumatology and Orthopedics named after N.N. Priorov" of the Ministry of Health of the Russian Federation, Professor of the Department of Medical

Rehabilitation of the Faculty of Continuing Professional Education of the Federal State Autonomous Educational Institution of Higher Education "Russian National Research Medical University named after N.I. Pirogov" of the Ministry of Health of Russia

Vladimir Borisovich Chentsov – Head of the Department of Resuscitation and Intensive Care of the State Budgetary Healthcare Institution of the City of Moscow "Infectious Diseases Clinical Hospital No. 2 of the Moscow City Healthcare Department"

Vladimir Petrovich Chulanov – Chief Specialist in Infectious Diseases of the Russian Ministry of Health, Deputy Director for Research and Innovative Development of the Federal State Budgetary Institution “National Medical Research Center for Phthisiopulmonology and Infectious Diseases” of the Russian Ministry of Health

Shelygin Yuri Anatolyevich - Chief Proctologist of the Ministry of Health of the Russian Federation, Chief Proctologist of the Moscow City Health Department, Director of the A. N. Ryzhikh State Scientific Center of Proctology of the Ministry of Health of the Russian Federation

Shestakova Marina Vladimirovna - Deputy Director of the Federal State Budgetary Institution "National Medical Research Center of Endocrinology" of the Ministry of Health of Russia, Director of the Institute of Diabetes of the Federal State Budgetary Institution "National Medical Research Center of Endocrinology" of the Ministry of Health of Russia

Shipulin German Aleksandrovich - Deputy Director of the Federal State Budgetary Institution "Center for Strategic Planning and Management of Medical and Biological Health Risks" of the Federal Medical and Biological Agency of Russia

Shlemskaya Valeria Vadimovna – Deputy Director of the Federal State Budgetary Institution “All-Russian Center for Disaster Medicine “Zashchita”” FMBA of Russia

Shmakov Roman Georgievich - Chief Specialist of the Ministry of Health of the Russian Federation in Obstetrics, Director of the Institute of Obstetrics of the Federal State Budgetary Institution "National Medical Research Center for Obstetrics, Gynecology and Perinatology named after Academician V.I. Kulakov" of the Ministry of Health of the Russian Federation

Shulgina Marina Vladimirovna - Advisor to the Director of Science of the Federal State Budgetary Institution "National Medical Research Center for Phthisiopulmonology and Infectious Diseases" of the Ministry of Health of the Russian Federation

Igor Semenovich Yavelov – Head of the Department of Fundamental and Clinical Problems of Thrombosis in Non-Infectious Diseases, Federal State Budgetary Institution “National Medical Research Center for Therapy and Preventive Medicine” of the Ministry of Health of the Russian Federation

Yaroshetskiy Andrey Igorevich - Professor of the Department of Pulmonology of the Federal State Autonomous Educational Institution of Higher Education "First Moscow State Medical University named after I.M. Sechenov" (Sechenov University) of the Ministry of Health of the Russian Federation, Head of the Department of Anesthesiology and Resuscitation of the Research Institute of Clinical Surgery of the Federal State Autonomous Educational Institution of Higher Education "Russian National Research Medical University named after N.I. Pirogov" of the Ministry of Health of the Russian Federation, Chairman of the Committee of the Federation of Anesthesiologists and Resuscitators for Respiratory and Metabolic Support

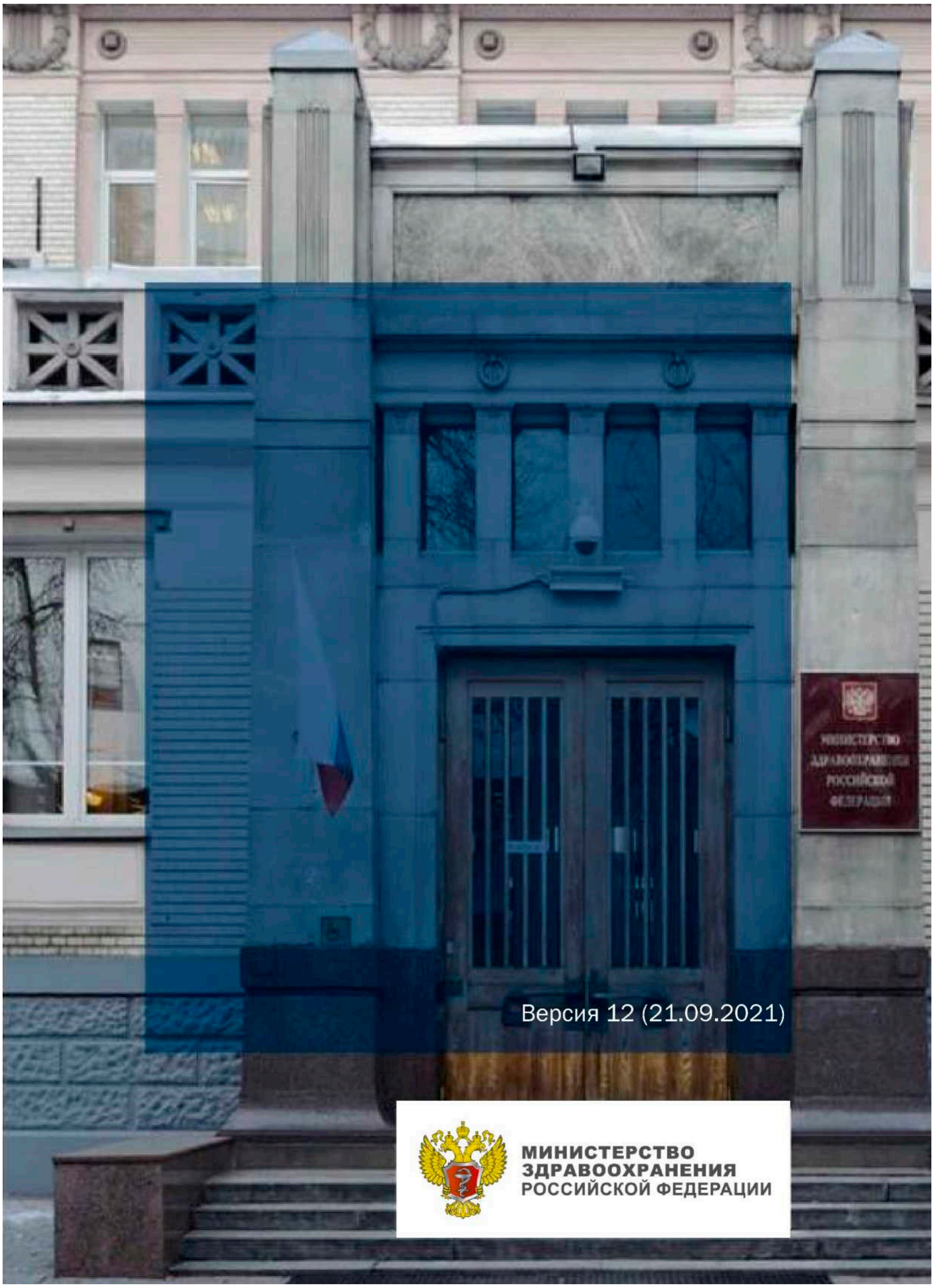
Версия 12 (21.09.2021)
МИНИСТЕРСТВО
ЗДРАВООХРАНЕНИЯ
РОССИЙСКОЙ ФЕДЕРАЦИИ
