# Supplementary material for: Severely Ill COVID-19 Patients May Exhibit Hypercoagulability Despite Escalated Anticoagulation
Source: J Clin Med. 2025 Mar 14;14(6):1966. doi: 10.3390/jcm14061966 (PMC11943368; doi:10.3390/jcm14061966)
Supplement: Supplementary file 1 [file jcm-14-01966-s001.zip › File S2. Supplement Tables and Figures.pdf]

Supplementary material

Severely Ill COVID-19 Patients May Exhibit Hypercoagulability Despite Escalated Anticoagulation

Soslan Shakhidzhanov [1,2,*], Anna Filippova [1,2], Elizaveta Bovt [1,2], Andrew Gubkin [3], Gennady Sukhikh [4], Sergey Tsarenko [5], Ilya Spiridonov [2], Denis Protsenko [6], Dmitriy Zateyshchikov [7], Elena Vasilieva [8], Anna Kalinskaya [8], Oleg Dukhin [8], Galina Novichkova [1], Sergey Karamzin [2], Ilya Serebriyskiy [2], Elena Lipets [2], Daria Kopnenkova [6], Daria Morozova [1,2], Evgeniya Melnikova [2], Alexander Rumyantsev [1] and Fazoil Ataullakhanov [1,2,*] for the COVITRO study group

[1] Dmitriy Rogachev National Medical Research Center of Pediatric Hematology, Oncology, and Immunology, 117997 Moscow, Russia; ae.zadorozhnaya@physics.msu.ru (A.F.); elizaveta.bovt@dgoi.ru (E.B.); novichkova.galina@dgoi.ru (G.N.); morozova.daria@ctppcp.ru (D.M.); alexander.rumyantsev@dgoi.ru (A.R.)

[2] Center for Theoretical Problems of Physicochemical Pharmacology, 109029 Moscow, Russia; spiridonov.ilya@ctppcp.ru(I.Spiridonov); karamzin.sergey@ctppcp.ru (S.K.); serebriyskiy.ilya@ctppcp.ru (I.Serebriyskiy); lipets.elena@ctppcp.ru(E.L.); melnikova.evgeniya@ctppcp.ru (E.M.)

[3] Central Clinical Hospital No. 2 Named After N.A.Semashko "RZD-Medicine", 121359 Moscow, Russia; gubkinav@gmail.com

[4] National Medical Research Center for Obstetrics, Gynecology and Perinatology Named after Academician V.I.Kulakov, 117997 Moscow, Russia; g_sukhikh@oparina4.ru

[5] City Clinical Hospital No. 52 of Moscow Health care Department, 123182 Moscow, Russia; s9637501492@yandex.ru

[6] Moscow Multiprofile Clinical Center "Kommunarka" of Moscow Healthcare Department, 142770 Moscow, Russia; drprotsenko@me.com (D.P.); primadaria95@gmail.ru (D.K.)

[7] City Clinical Hospital No. 51 of Moscow Health care Department, 121309 Moscow, Russia; dz@bk.ru

[8] City Clinical Hospital No. 23 of Moscow Health care Department, 109004 Moscow, Russia; vasilievahelena@gmail.com (E.V.); kalinskaya.anna@gmail.com (A.K.); dukhinoa@zdrav.mos.ru (O.D.)

* Correspondence: soslan.shakhidzhanov@dgoi.ru (S.S.); ataullakhanov.fazly@ctppcp.ru (F.A.); Tel.: +1-215-746-8177 (F.A.)

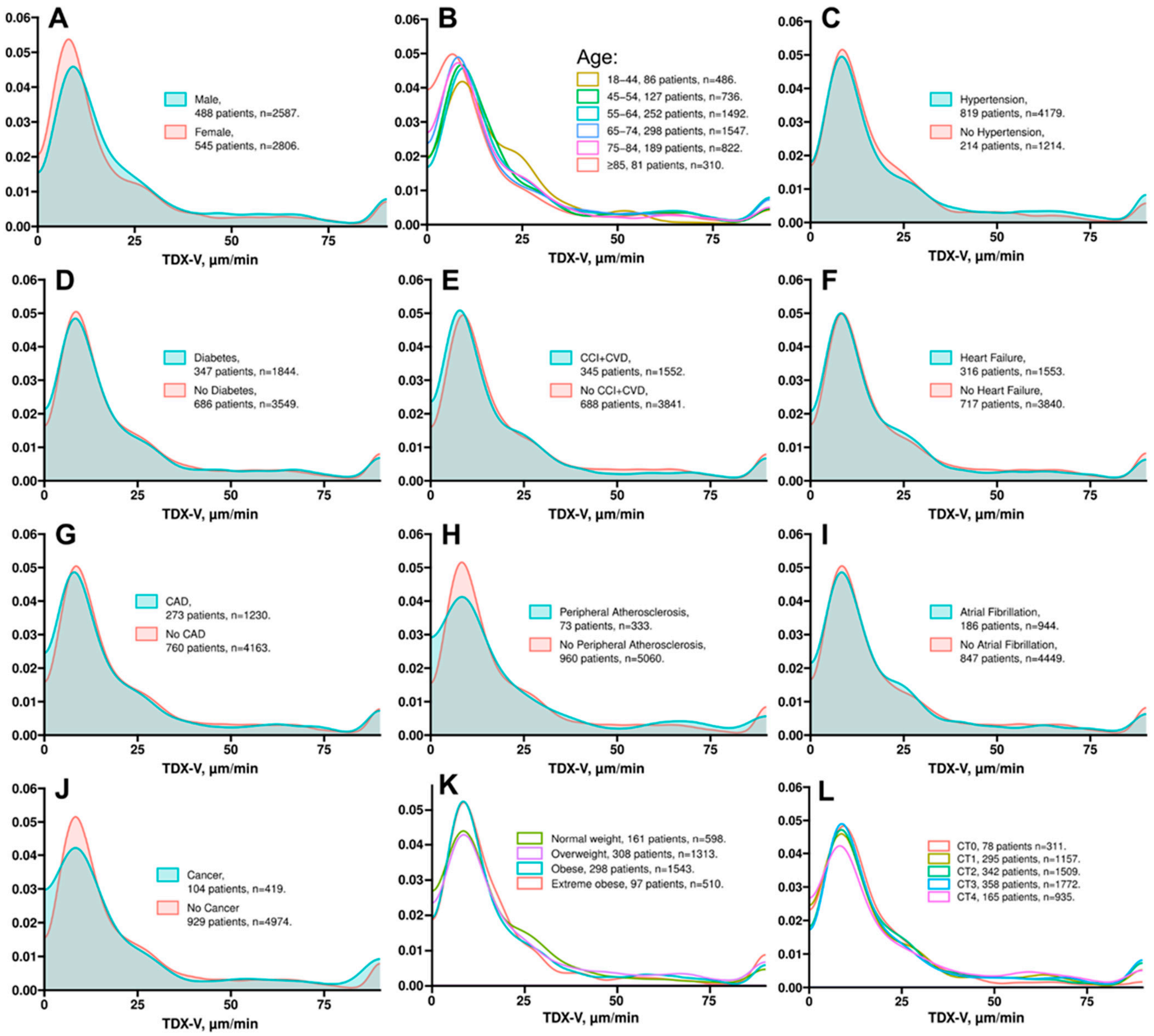

Figure S1. TDX-V distributions in severely ill patients receiving therapeutic doses of heparins with adjustment to sex, age, and comorbidities. Distributions of patients with specific risk factors are shown in blue, distributions of patients without them are shown in red. The red and blue distributions were almost similar. A number of patients in each group and a total number of measurements are shown in the legends. (A) – Sex. (B) – Age. (C) – Hypertension. (D) – Diabetes mellitus. (E) – Chronic cerebral ischemia + Cerebrovascular disease. (F) – Heart failure. (G) – Coronary artery disease. (H) – Peripheral atherosclerosis. (I) – Atrial fibrillation. (J) – Cancer. (K) – Patient's body mass index, normal weight: 18.6—24.9; overweight: 25—29.9; obese: 30—39.9; extreme obese: ≥ 40. A sample size of patients with underweight was too small. (L) – CT score at admission.

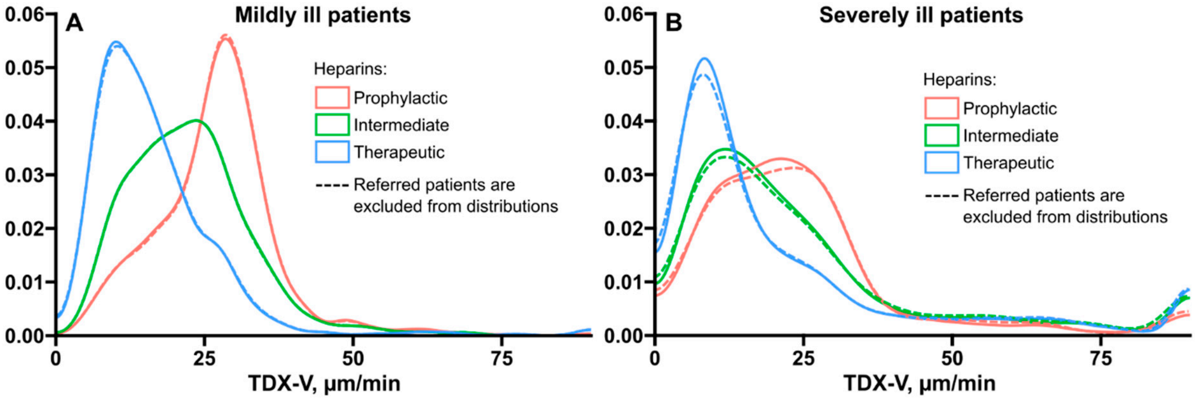

Figure S2. **TDX-V distributions with and without patients referred form other clinics. (A)** - distributions of mildly ill patients receiving different doses of heparins (solid lines) and the same distributions when patients referred from other hospitals are excluded (dashed lines). Sample sizes of the distributions of all patients: prophylactic n=1063, intermediate n=1539, therapeutic n=594. Sample sizes of the distributions when patients referred from other hospitals are excluded: prophylactic n=1046, intermediate n=1529, therapeutic n=573. **(B)** - distributions of severely ill patients receiving different doses of heparins (solid lines) and the same distributions when patients with adjusted doses of heparins are excluded (dashed lines). Sample sizes of the distributions of all patients: prophylactic n=427, intermediate n=1722, therapeutic n=5393. Sample sizes of the distributions when patients referred from other hospitals are excluded: prophylactic n=328, intermediate n=1431, therapeutic n=4850.

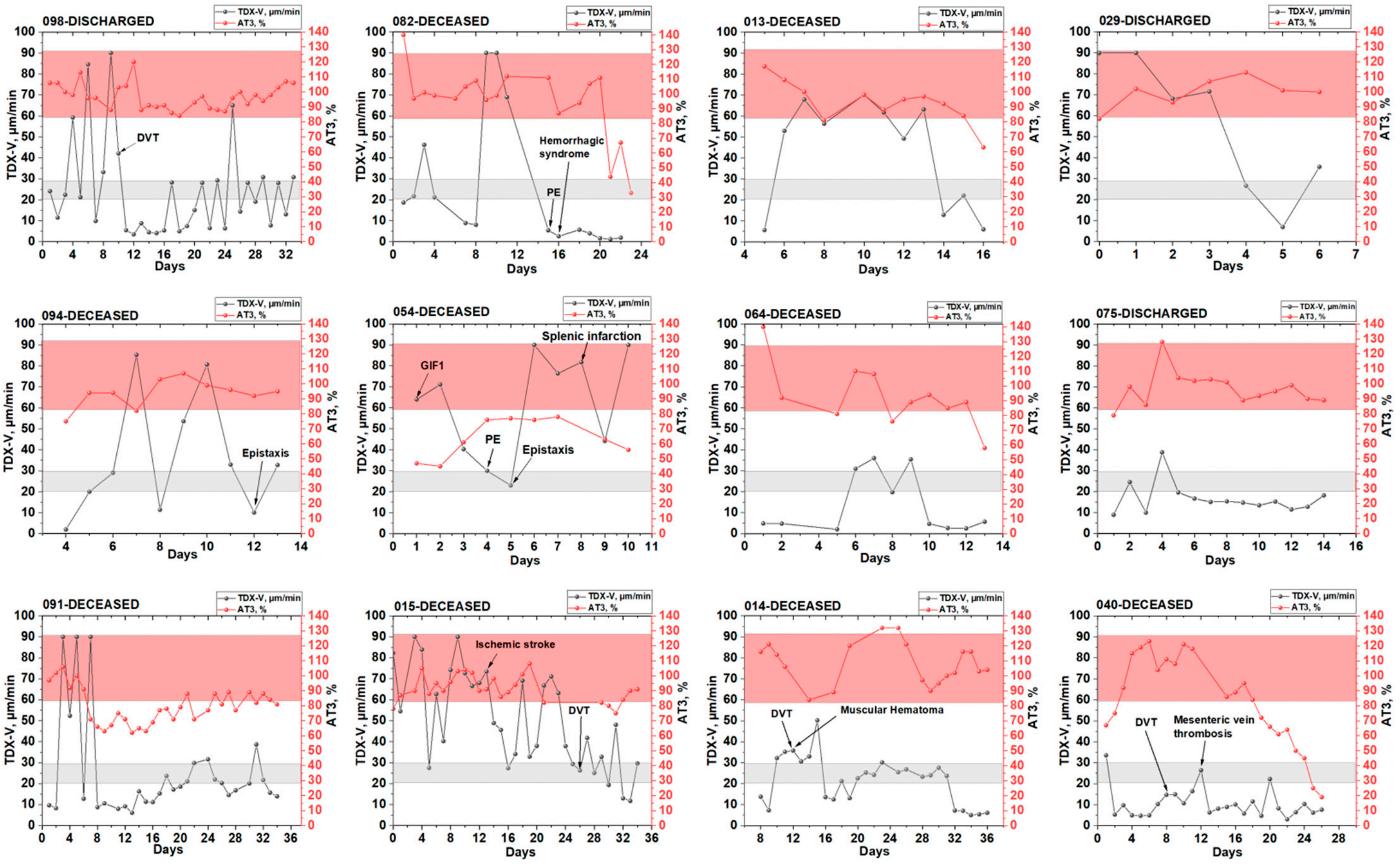

Figure S3. examples of TDX-V and AT3 level time-courses. TDX-V time-courses are shown in *black*. The TDX-V reference range (20-29 μm/min) is shown with grey areas, with its axis on the left-hand side. The AT3 time-courses are shown in *red*. The AT3 level reference range (84-126%) is shown with red areas, with its axis on the right-hand side. Arrows indicate the moments when complications were confirmed. Large fluctuations of both AT3 and TDX-V are notable, but AT3 generally remains within the reference range when TDX-V is high. **Patient 054** is an exception; he had low AT3, which resulted in several thrombotic complications. He was also hospitalised with gastrointestinal bleeding. **Patient 040** experienced thrombotic complications during plasma hypocoagulability. **Patient 014** developed muscular hematoma during ECMO. GIF - gastrointestinal bleeding Forrest 1, DVT - deep vein thrombosis, PE - pulmonary embolism.

Table S1. Cox regressions for different time to event models in severely ill patients.

Model	N	Variable	Base model HR (95% CI), p-val	Model adjusted for age, sex and BMI HR (95% CI), p-val
Death, D-dimer ≥5000 ng/ml	534	Intense hypercoagulability (IH) [#)]	1.27 (1.12-1.44), ***	1.26 (1.10-1.43), ***
		Age	-	1.40 (1.24-1.58), ***
		Male	-	0.99 (0.79-1.25), ns
		BMI	-	0.99 (0.87-1.13), ns
Thrombosis, D-dimer ≥5000 ng/ml	444	Intense hypercoagulability (IH) [#)]	1.91 (1.54-2.35), ***	1.90 (1.54-2.34), ***
		Age	-	0.99 (0.82-1.21), ns
		Male	-	0.88 (0.59-1.31), ns
		BMI	-	0.79 (0.63-0.99), *
Death, APTT ≤25.1 sec	641	Intense hypercoagulability (IH) [#)]	0.98 (0.82-1.18), ns	1.04 (0.87-1.25), ns
		Age	-	1.44 (1.29-1.62), ***
		Male	-	0.96 (0.78-1.18), ns
		BMI	-	1.07 (0.96-1.20), ns
Thrombosis, APTT ≤25.1 sec	523	Intense hypercoagulability (IH) [#)]	1.15 (0.87-1.52), ns	1.15 (0.87-1.52), ns
		Age	-	0.99 (0.83-1.20), ns
		Male	-	0.89 (0.62-1.28), ns
		BMI	-	0.87 (0.72-1.06), ns

[#)]The regression variable IH reflected how long high D-dimer values or shortened APTT times were experienced by a patient. IH = 0 if the patient had such values for <25% of the time up to the event (death or thrombosis). IH = 1 if they were observed for ≥25% and IH = 2 if they were observed for ≥50% of the time up to the event. HR – hazard ratio, CI – confidence interval, BMI – body mass index.

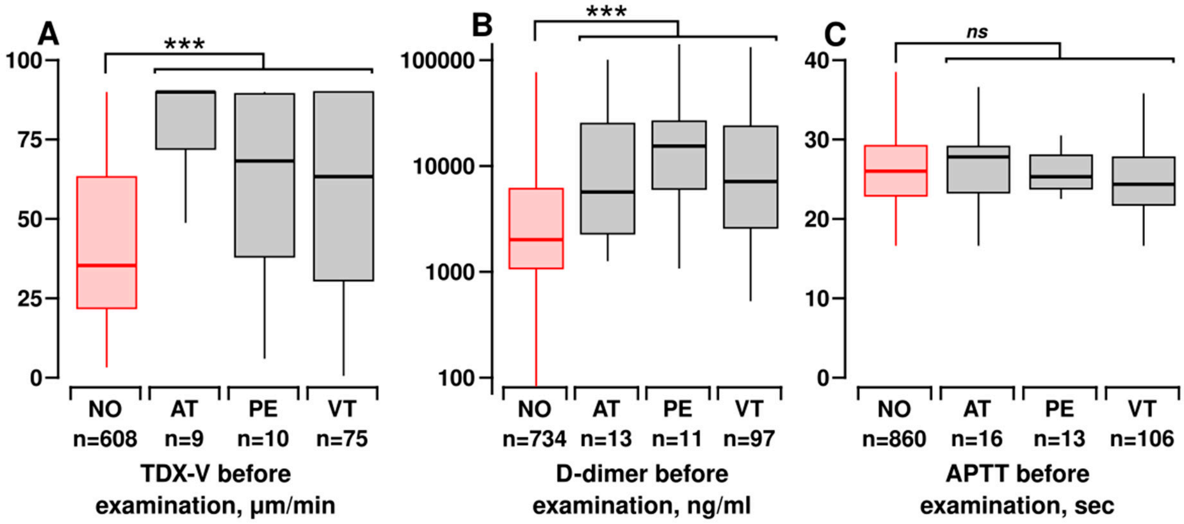

Figure S4. APTT, D-dimer and TDX-V values shortly before the confirmation of a new thrombosis. The patients had instrumental examinations of the arterial and vein patency of lower and upper limbs at least every eight days, and computer tomography angiography in the case of symptoms of pulmonary or arterial embolism. APTT, D-dimer and TDX-V values were obtained prior to instrumental examination which confirmed thrombosis (AT - arterial thrombosis, PE - pulmonary embolism, VT - venous thrombosis) or did not confirm it (NO - no new thrombosis). Statistical significance was assessed using the Mann-Whitney U test. **(A)** - APTT values prior to confirmation of a new thrombosis did not differ from control. **(B)** - D-dimer values were higher prior to confirmation of a new thrombosis. **(C)** - TDX-V were higher prior to confirmation of a new thrombosis.

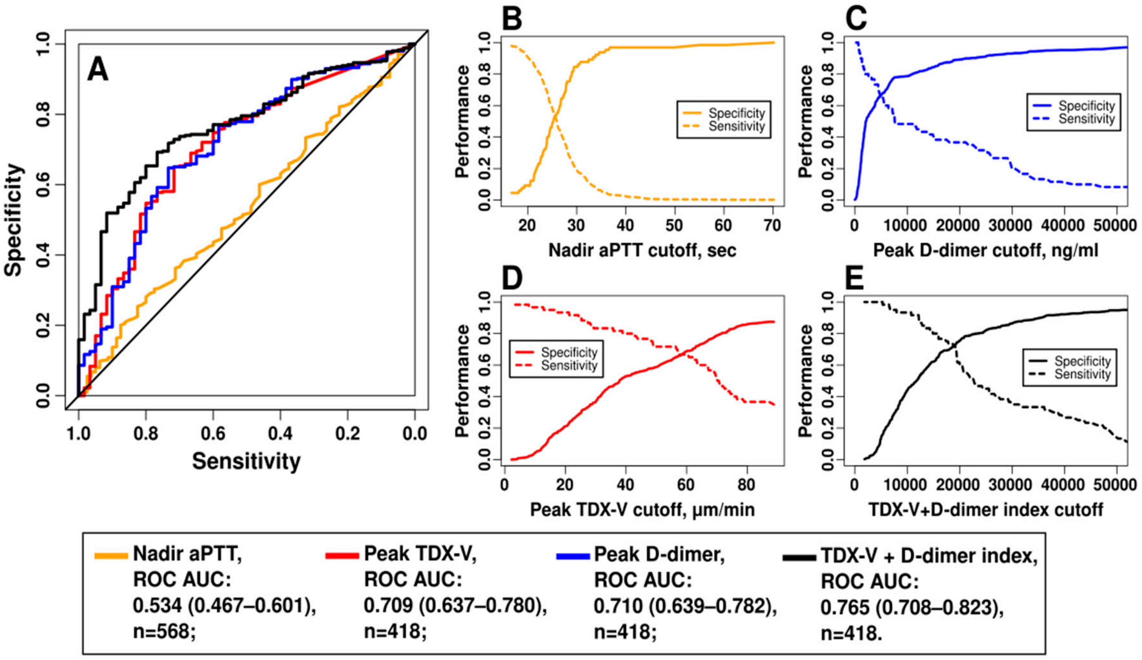

Figure S5. usage of various coagulation indices to predict thrombotic complications. Nadir APTT (*yellow*), peak D-dimer level (*blue*), peak TDX-V (*red*), and a combined TDX-V and D-dimer index (*black*) are shown. **(A)** - Error curves for various markers, with the areas under the curves; their values with the 95% confidence intervals and sample sizes are shown at the bottom of the figure. **(B-E)** - sensitivity and specificity of indices for different threshold levels. **(B)** - nadir APTT, **(C)** - peak D-dimer, **(D)** - peak TDX-V, **(E)** - combined TDX-V and D-dimer index[1)]. The combined index had the largest area under the error curve.

[1)] The value was calculated as $200*z_1+z_2$, where z_1 is the peak TDX-V, and z_2 is the peak D-dimer level.

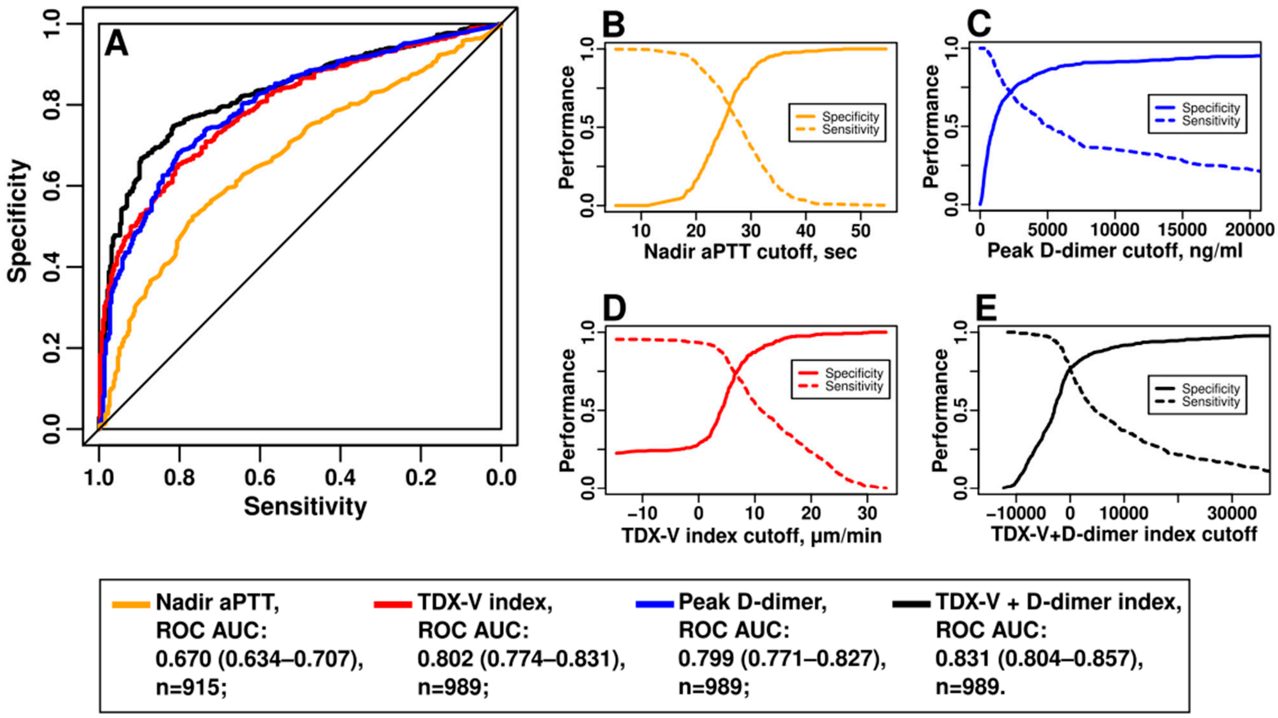

Figure S6. usage of various coagulation indices to predict lethal outcomes. Nadir APTT (*yellow*), peak D-dimer level (*blue*), a Thrombodynamics index calculated from the minimum and maximum TDX-V values during hospitalization[2)] (*red*), and a combined index from Thrombodynamics and D-dimer level[3)] (*black*) are shown. **(A)** - error curves for various markers, with the areas under the curves; their values with the 95% confidence interval and sample sizes shown at the bottom of the figure. **(B-E)** - sensitivity and specificity of indices for different threshold levels. **(B)** - nadir APTT, **(C)** - peak D-dimer level, **(D)** - the Thrombodynamics index[2)], **(E)** - the combined index from Thrombodynamics and D-dimer level[3)]. The combined index from Thrombodynamics and D-dimer level had the largest area under the error curve.

[2)] First, we identified the minimum and maximum TDX-V values obtained during a patient's hospitalisation. Second, the smaller value between TDX-V_{min} and (75 - TDX-V_{max}) was chosen as the patient's index. For example, if a patient's minimal TDX-V is 10 µm/min and maximal TDX-V is 70 µm/min, then 10 is greater than (75 - 70) = 5. Therefore, the index for this patient is 5. This index considers the risks of lethal outcomes due to both hypocoagulability and hypercoagulability.

[3)] The combined index of D-dimer level and Thrombodynamics was calculated as y_1 - 385*y_2. Here, y_1 is the patient's peak level of D-dimer and y_2 is the TDX-V index calculated in [2)].
