# Supplementary material for: Severely Ill COVID-19 Patients May Exhibit Hypercoagulability Despite Escalated Anticoagulation
Source: J Clin Med. 2025 Mar 14;14(6):1966. doi: 10.3390/jcm14061966 (PMC11943368; doi:10.3390/jcm14061966)
Supplement: Supplementary file 1 [file jcm-14-01966-s001.zip › File S3. Thrombodynamics revised.pdf]

Severely Ill COVID-19 Patients Experience Hypercoagulability Despite Receiving Escalated Anticoagulation

DESCRIPTION OF THROMBODYNAMICS AND ITS CLINICAL APPLICATION

Coagulation factors and inhibitors are evenly dissolved in blood plasma; however, clot formation is a spatially inhomogeneous process (79). The process begins at the site of vascular injury, on the surface of cells expressing tissue factor (TF). Accordingly, the first active coagulation factors appear on the surface of TF-expressing cells. The gradual diffusion of activated coagulation factors leads to the assembly of complexes of the inner tenase (fIXa-fVIIIa) and prothrombinase (fXa-fVa) on the membranes of activated platelets or microvesicles located away from the TF surface. Thus, clot formation spreads from the site of vascular damage. At this stage, the rates of the enzymatic reactions and the rates of diffusion are both important.

Thrombodynamics is a clinical laboratory assay which considers spatial heterogeneity of plasma clotting and imitates plasma coagulation *in vivo*. It received approval for clinical use in Russia and several Central Asian countries.

Procedure
Thrombodynamics assay is conducted using a specialized laboratory diagnostic system "Thrombodynamics Analyzer T2-F" (LLC HemaCore, Moscow, Russia)[1] and kits (LLC HemaCore, Moscow, Russia) consisting of corn trypsin inhibitor, calcium acetate and a plastic insert with relipidated recombinant TF (Instrumentation Laboratory, Bedford, USA) immobilized on its ends. LLC HemaCore provides the information about the density of immobilized TF: 113±26 pmoles/m^2; ACTICHROME® TF activity assay is used. Thrombodynamics utilizes platelet-free blood plasma, prepared through two consecutive steps of centrifugation and supernatant collection (41). Corn trypsin inhibitor is added to plasma and it is recalcified immediately before the test. Prepared plasma samples are placed into the channels of a special measuring cuvette (**Fig. T1A**). Next, the insert is loaded into a cuvette and its ends come into contact with plasma. It initiates coagulation process and a fibrin clot begins to grow away from the ends of the insert. Its growth is recorded for 30 minutes with a digital camera in the dark field mode (registration of the light scattering pattern). The resulting series of images reveals changes in the size, shape, and density of the fibrin clot over time (**Fig. T1B and T1C**).

[1] https://hemacore.com/en/

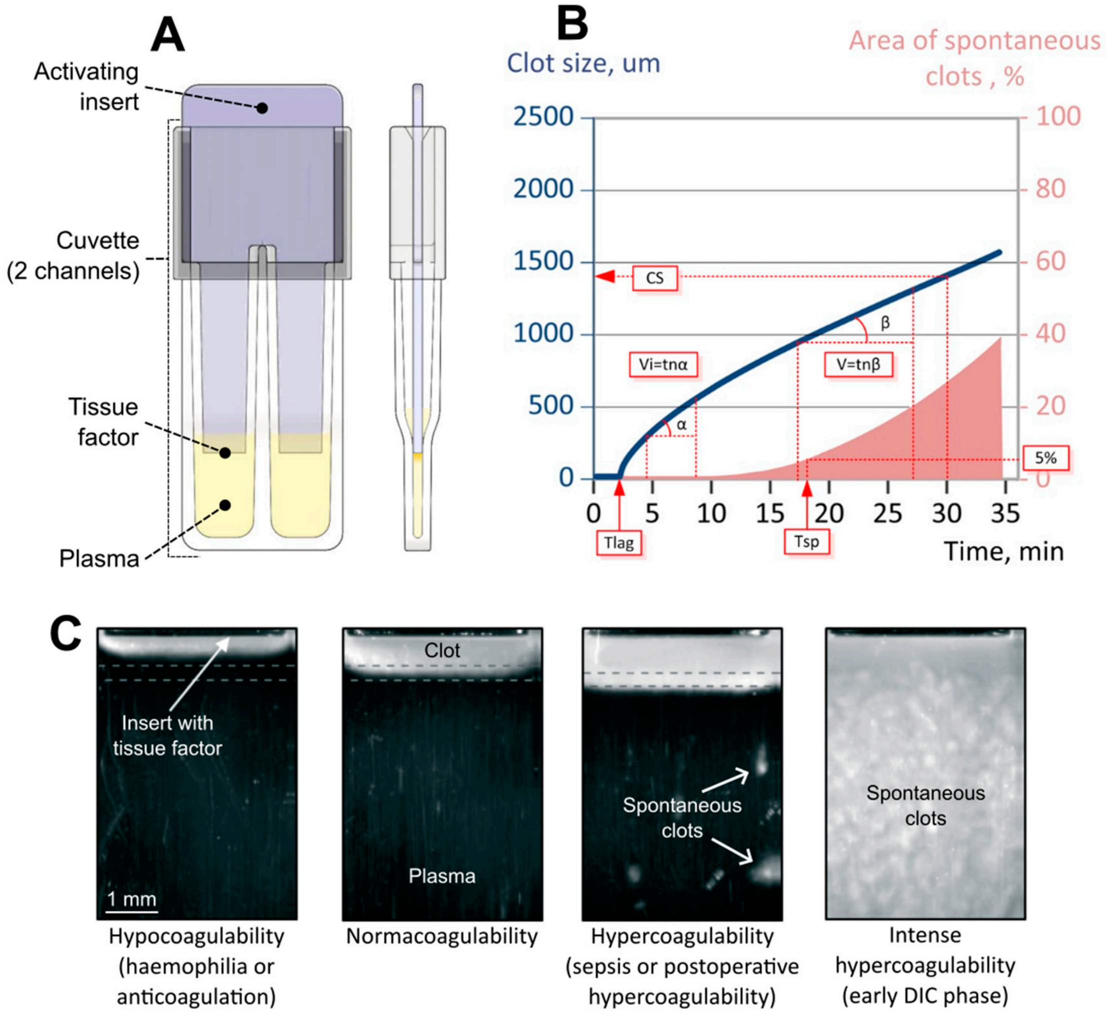

Figure T1. **Principles of Thrombodynamics. (A)** - Measuring cuvette with activator insert: tissue factor (TF), which initiates clotting upon contact with plasma, is immobilized on the ends of the plastic activating insert. Measurements are carried out simultaneously in both channels of a cuvette. **(B)** - Graphs of clot size (blue line) and spontaneous plasma coagulation area (red) as a function of time. **(C)** - Photographs of fibrin clot in Thrombodynamics for various states of coagulation. The photographs were taken 30 minutes after the start of the assay.

In addition to primary clot growing from the activating insert, spontaneous coagulation may be observed within the plasma volume which does not contact with the primary clot. This phenomenon can be seen in patients with intense hypercoagulability, such as in the initial phase of disseminated intravascular coagulation (DIC) syndrome, and is attributed to the presence of the patient's own procoagulant components in the plasma sample - microvesicles, active coagulation factors, and traces of tissue factor (80).

Thrombodynamics Parameters

The Thrombodynamics software utilizes the captured images to generate a clot size versus time plot (**Fig. T1B**) and calculates numerical parameters that characterize the spatial dynamics of the fibrin clot growth and spontaneous coagulation.

- Lag-time (Tlag) - the delay before clot formation after plasma contacts the activating insert. It can be used to monitor anticoagulant therapy with vitamin K antagonists (VKAs) since lag-time increases during such therapy (81). Additionally, lag-time is sensitive to the effects of direct oral anticoagulants (DOACs): factor Xa inhibitors (rivaroxaban) and prothrombin inhibitors (dabigatran etexilate). Lag-time also increases with severe deficiencies in factors of the extrinsic and common pathways (82).
- Clot growth rate (V or TDX-V) - the average clot growth rate calculated within the interval of 15-25 minutes after beginning of the clot growth. If the parameter V cannot be calculated within the specified interval due to the intensive formation of spontaneous clots, it is calculated over the 5-minute interval preceding the onset of spontaneous clot formation. It is the main parameter of the assay and is highly sensitive to changes in hemostasis of various origins, from hemophilia to hypercoagulable states (e.g., in the postoperative period (83), during disseminated intravascular coagulation (42), enterectomy (84)). TDX-V decreases during therapy with heparins and VKAs (81), and in cases of severe deficiencies in factors of the intrinsic and common pathways (82).
- Time to spontaneous clot formation (Tsp) in plasma volume - a moment when the volume outside the growing clot becomes filled with spontaneous clots covering 5% of the volume. This indicates a high prothrombotic potential of the tested plasma. The presence of activated coagulation factors (e.g., factors IXa and XIa), tissue factor, and an increased concentration of procoagulant microvesicles in the blood leads to the formation of spontaneous clots (80). A shortened Tsp indicates an increase in prothrombotic activity.
- Initial clot growth rate (Vi) - the average rate of clot growth calculated within the interval of 2-6 minutes after beginning of the clot growth.
- Fibrin clot size (CS) - the size of the clot by the end of the assay. It is an integral characteristic reflecting the combined activity of individual components of the coagulation system.
- Clot density (D) - an optical measure equal to the intensity of light scattering by the fibrin clot. It allows detection of abnormalities in fibrinogen concentration or functional activity: D decreases proportionally with fibrinogen deficiency. Increasing fibrinogen concentration is accompanied by a moderate increase in D (85, 86).

A distinctive feature of Thrombodynamics is the ability to visually observe the process of spatial clot growth. The recorded video of clot growth provides clear information about the state of the coagulation system. The manufacturer provides reference ranges of normal Thrombodynamics parameters (**Tab. T1**), which were obtained from plasma samples of healthy volunteers (41).

Table T1. Reference ranges of the Thrombodynamics parameters.

Parameter	Reference range
Lag-time (Tlag)	0.6-1.5 min
Clot growth rate (V or TDX-V)	20-29 μm/min
Time to spontaneous clot formation (Tsp)	>30 min
Initial clot growth rate (Vi)	38-56 μm/min
Clot size (CS)	800-1200 μm
Clot density (D)	15000 - 32000 AU

Clinical Application of Thrombodynamics

More than 200 scientific articles have been published; most of them focus on evaluating the clinical informativeness of the method. This section summarizes the results of the clinical application of Thrombodynamics.

Patients having high risk of developing thrombotic complications

A large number of studies allow us to confidently state that Thrombodynamics adequately reflects the hemostatic status in patient groups characterized by an increased risk of thrombotic complications. In such patients, Thrombodynamics demonstrates the presence of hypercoagulability disorders, primarily manifested by increased TDX-V and spontaneous clot formation. Thrombodynamics detected hypercoagulability in patients with the following conditions:

- after surgical intervention (87-89),
- in oncological patients (90, 91),
- in patients with autoimmune diseases (92, 93),
- in severe infectious diseases (42, 94, 95),
- in patients with cardiovascular diseases (96, 97),
- in cases of hemolytic anemias (98),
- in cases of cerebrovascular disorders (99).

Moreover, these changes have a progressive nature: hypercoagulability worsens in patients having more severe course of disease and comorbidities.

Prediction of thrombotic complications

Studies examining the potential of Thrombodynamics in predicting thrombosis can be divided into observational and intervention cohort studies. Observational studies show that patients with registered thromboses have significantly higher TDX-V, than patients without thrombosis (84, 100, 101). Details of the studies are provided in **Table T2**. Clinical cases show that an increase in clotting precedes the development of thrombosis (42, 102-104).

It is worth noting that in the group of patients with idiopathic deep vein thrombosis of the lower extremities (105), Thrombodynamics shows only a slight increase in clotting during the post-thrombotic period (patients sought assistance one week after the onset of thrombosis). This is likely due to the absence of systemic hypercoagulability in these patients and the lability of the hemostatic system, which quickly normalizes after thrombus formation.

Monitoring of anticoagulation therapy

It has been shown that Thrombodynamics is sensitive to all major anticoagulants: unfractionated (UFH) and low molecular weight (LMWH) heparins, vitamin K antagonists (VKA), and direct oral anticoagulants (DOACs). It is valuable that the effects of various medications on clot growth in the assay are not uniform (**Fig. T2**):

- Heparins in prophylactic and therapeutic doses have almost no effect on lag-time but significantly (1.5-5 times) reduce TDX-V (41, 42).
- Warfarin therapy leads to approximately a two-fold prolongation of lag-time compared to normal and a slight (20-30% reduction compared to normal) decrease in TDX-V (89, 106, 107).
- Preliminary data indicate that DOACs (rivaroxaban and dabigatran etexilate) also prolong lag-time and reduce clot growth rate, but the effect is less pronounced compared to heparins and warfarin (81).

Other studies focus on evaluating the effectiveness of thrombosis prevention using Thrombodynamics. It has been shown that a significant proportion of patients (from 20% to 40%) remain in normal or hypercoagulability states despite receiving anticoagulant prophylaxis (88, 108, 109). Therefore, the usage of Thrombodynamics holds promise for identifying high-risk groups for thrombosis and for tailoring anticoagulant prophylaxis to these patients. These studies have demonstrated that Thrombodynamics can be used for personalized anticoagulant therapy, effectively reducing the risks of thrombosis and other adverse outcomes (110-112), as detailed in **Table T2**. Additionally, Thrombodynamics detects the excessive doses of anticoagulation, corroborated by observed bleeding events (81, 113).

Table T2. Results of clinical usage of Thrombodynamics to assess the risk of thrombotic complications and their prevention.

Patients	Outcome	% of patients with adverse outcomes	Conclusion	Ref.
**Observational cohort studies**				
80 patients with colorectal cancer after surgery having high risk of thrombosis according to the Caprini score and receiving standard thromboprophylaxis (elastic compression + 40 mg enoxaparin once daily).	The presence/absence of deep vein thrombosis in the early postoperative period (symptomatic or asymptomatic - assessed by ultrasound on the 8th day after surgery).	26.3% (21 patient)	Thrombodynamics enhances the predictive ability of the Caprini score (AUC increases from 0.839 ± 0.045 to 0.924 ± 0.029) in patients undergoing extensive surgical interventions regarding deep vein thrombosis.	84
59 patients with prostate cancer after surgery having high risk of thrombosis according to the Caprini score and receiving standard thromboprophylaxis (elastic compression + 0.3 or 0.6 mL (for weight exceeding 70 kg) of nadroparin once daily).	The presence/absence of deep vein thrombosis in the early postoperative period (symptomatic or asymptomatic - assessed by ultrasound on days 3 and 10-14 after surgery).	5.1% (3 patients)	TDX-V exceeding 43 μm/min before the initial administration or within 3 hours after injection of nadroparin calcium was associated with an increased risk of developing VTE (two-sided Fisher's exact test, $p = 0.0172$).	100
80 patients admitted to the hospital for non-cardiac surgical procedures.<br>Before surgery, LMWH therapy was given to 34.6% (n = 9) of patients. In the postoperative period, VTE prophylaxis with LMWH was in 58.8% (n = 46) of patients.	The presence/absence of deep vein thrombosis in the early postoperative period.	2.5% (2 patients)	The initial growth rate of the clot in patients with thrombosis was significantly higher compared to other patients during the first postoperative day: 61.0 ± 1.83 vs. 52.1 ± 12.2 μm/min (t-test, $p = 0.004$).	83
153 COVID-19 patients observed in a hospital setting. All patients received therapeutic LMWH (n = 108, except for ECMO patients who received UFH (n = 15). Thrombodynamics, thromboelastography, and standard clotting assays were performed.	76% of patients had hypercoagulability on admission. Anticoagulation resulted in hypocoagulability in 76.8% of patients and normal coagulability in 14.7% of patients.	8.5% of patients had hypercoagulability despite anticoagulation.	Patients with COVID-19 have hypercoagulability on admission, anticoagulation resolved hypercoagulability in most patients. Correlations between TEG R and K and TDX-V, Vi, and CS were observed.	72
**Interventional cohort studies**				

Patients	Outcome	% of patients with adverse outcomes	Conclusion	Ref.
245 patients with SARS-CoV-2-associated pneumonia: Group I - 117 patients received prophylactic dose of LMWH (enoxaparin 40 mg once daily subcutaneously), Group II - 128 patients received prophylactic dose of anticoagulant (enoxaparin) adjusted based on Thrombodynamics results (could be increased to 80 mg twice daily subcutaneously).	The presence/absence of positive dynamics in clinical symptoms (improvement in patient's condition, reduction in lung tissue damage area, normalization of laboratory parameters, including decrease in D-dimer levels at discharge) and lethal outcomes.	No positive dynamics in clinical symptoms: 13.7% (14 patients) in Group I, 4.1% (5 patients) in Group II. Mortality outcome: 6.4% (7 patients) in Group I, 0% in Group II.	Positive dynamics was identified 1.8 times more frequently in patients of Group II compared to Group I ($p < 0.05$). By the end of the study, the number of deaths in Group I exceeded those in Group II by 3.3 times ($p < 0.01$). No haemorrhagic complications were registered in either group.	110
A total of 106 patients with severe COVID-19 who were admitted to the ICU and had no prior history of COVID-19 treatment were included in the study. After inclusion in the protocol, all patients were divided into 2 groups: Group A - 49 patients received dosage of LMWH adjusted according body weight. Group B - 57 patients received dosage of LMWH adjusted based on Thrombodynamics results.	The presence/absence of thrombosis, pulmonary embolism, stroke and clinically significant bleeding (BARC types 2-5).	Thrombosis: The incidence of thrombosis was 3 times lower in Group B, where anticoagulation was performed under TD control, compared to Group A without TD control (7% vs 23%). Bleeding: There were no clinically significant bleeding episodes in Group B, and there were 3 episodes in Group A: neck hematoma in one patient, gluteal hematoma with hip hematoma in the second patient and inguinal hematoma with hip hematoma in the third. The number of outcomes is not enough for statistical analysis.	The use of Thrombodynamics to monitor LMWH prophylaxis in patients with severe COVID-19 significantly reduces the frequency of VTE. ROC analysis showed that the Thrombodynamics parameter mean clot growth rate V at the 3-day interval (<V3>) had a high predictive power for thrombosis (area under the ROC curve AUC=0.83, Figure 9). The cutoff value for <V3> was 25 μm/min (sensitivity 80%, specificity 80% for thrombosis detection). The relative risk of thrombosis if the mean clot growth rate <V3> exceeded the threshold value of 25 μm/min was 14.3 (p=0.0005, 95% confidence interval 3.2-63.7).	126

Patients	Outcome	% of patients with adverse outcomes	Conclusion	Ref.
61 patients who underwent TIPS (Transjugular Intrahepatic Portosystemic Shunt) without embolization of gastroesophageal veins: Group I (control) - 31 patients received medication according to standard treatment for liver cirrhosis patients, Group II - 30 patients received anticoagulant therapy based on Thrombodynamics results.	The presence/absence of shunt thrombosis (assessed by the presence/absence of flow through the shunt), follow-up period ranged from 6 to 36 months.	16.1% (5 patients) in Group I, 0% in Group II.	The use of Thrombodynamics to monitor LMWH prophylaxis in patients with liver cirrhosis undergoing transjugular intrahepatic portosystemic shunt surgery significantly reduces the frequency of VTE in the postoperative period (exact two-sided Fisher's test <0.001).	111
Patients having high risk of thrombosis due to malignant neoplasms of the gastrointestinal tract, undergoing surgical treatment: Group I - 77 patients received prophylactic dose of LMWH (enoxaparin 40 mg once daily subcutaneously), Group II - 64 patients received prophylactic dose of anticoagulant (enoxaparin) adjusted based on Thrombodynamics results (could be increased to 60 mg once daily subcutaneously).	The presence/absence of proximal thrombosis and pulmonary embolism on days 7-10 after surgery.	14.3% (11 patients) in Group I 4.7% (3 patients) in Group II.	The use of thrombodynamics to monitor LMWH prophylaxis in oncology patients having high risk of thrombosis significantly reduces the frequency of VTE in the postoperative period (Fisher's exact test <0.05).	101

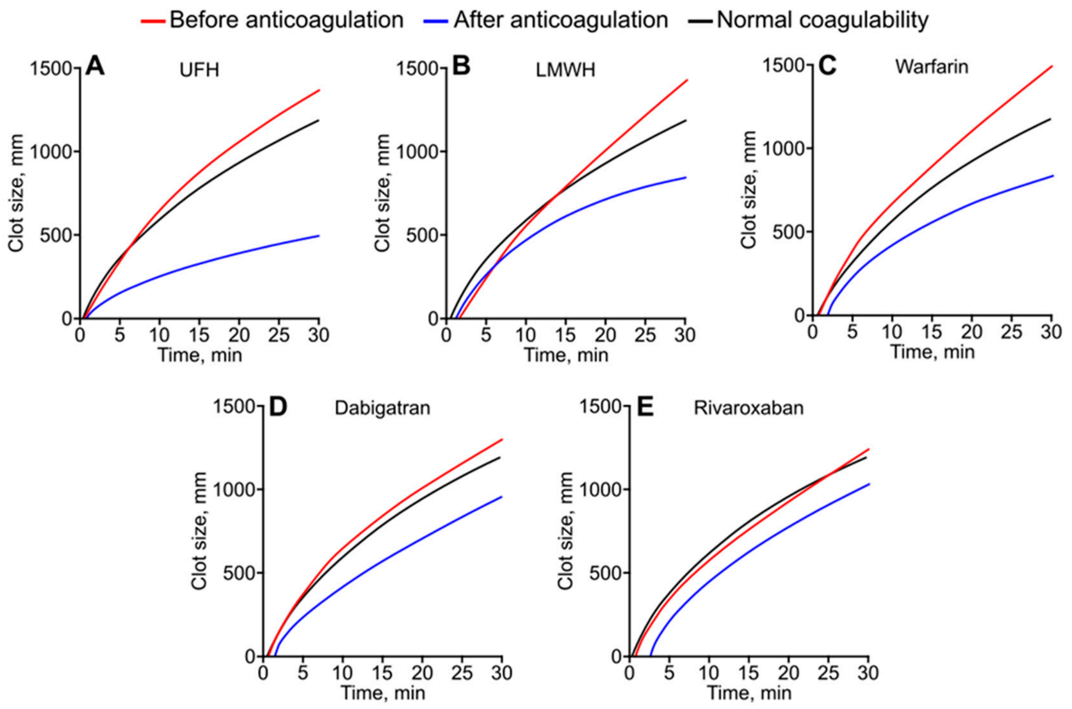

Figure T2. **Sensitivity of Thrombodynamics to different types of anticoagulants.** Fibrin clot growth curves before (red curve) and after (blue curve) anticoagulation. The black curves represent clot growth in plasma of a healthy donor. **(A)** - before and 2 hours after an injection of 10000 IU of UFH; **(B)** - before and 4 hours after an injection of 3000 IU of LMWH; **(C)** - before and one week after starting warfarin therapy at a dose of 5 mg/day; **(D)** - before and 2 hours after taking 110 mg of dabigatran etexilate; **(E)** - before and 2 hours after taking 15 mg of rivaroxaban.

Coagulopathies

Clinical studies show Thrombodynamics sensitivity to severe coagulation factor deficiencies. Examination of patients with severe hemophilia A showed a decrease in TDX-V to 14 μm/min, failing to reach the lower limit of normal (20 μm/min) in any patient (114). Patients having sepsis or septic shock exhibited a decline in TDX-V over several days leading to a hypocoagulability state just before death (likely due to disseminated intravascular coagulation and total depletion of clotting factors) (42). In such cases, Thrombodynamics parameters can serve as prognostic indicators of mortality. Additionally, a sharp decrease in TDX-V, associated with fatal outcomes, has been recorded in severe infectious diseases (94).

Obstetrics

Pregnancy, childbirth, and the early postpartum period are times of increased risk for thrombosis development, with several associated pathologies linked to a very high risk of thrombotic complications (115). In addition, infertility and miscarriage in some cases have causes related to hypercoagulability and, consequently, disturbances in placental blood flow.

Thrombodynamics adequately reflects physiological enhancement of coagulation during normal pregnancy (116, 117). Women with a history of miscarriage exhibit hypercoagulability disorders according to Thrombodynamics (118), and IVF programs are less effective in patients with Thrombodynamics-detected hypercoagulability before the protocol initiation (119). Hemostasis in women after cesarean section shows a state of hypercoagulability according to Thrombodynamics, which is not resolved even with prophylactic doses of LMWH in half of the patients, indicating the need for individualized anticoagulant dosing and hemostasis monitoring (83). Clinical cases demonstrate that using Thrombodynamics to monitor anticoagulation allows achieving pregnancy and successfully completing childbirth (120, 121).

Pediatrics and neonatology

Thrombodynamics in children from one to 18 years has shown no significant differences in coagulation compared to adults (85, 122). On the other hand, healthy full-term newborns in the first three days of life demonstrate enhanced coagulation: spontaneous clot formation (123). This phenomenon is likely related to the deficiency of coagulation inhibitors observed in newborns.

Research on hemostasis in preterm infants has shown even greater enhancement of coagulation compared to full-term infants (123). Newborns with necrotizing enterocolitis demonstrate pronounced hypercoagulation compared to newborns of the same gestational age without inflammation (124). Additionally, a clinical case has been described where Thrombodynamics facilitated successful selection of anticoagulant therapy for a newborn with congenital heart defect undergoing treatment for right atrial thrombosis (125).

SUPPLEMENTAL REFERENCES

79. Panteleev MA, Dashkevich NM, Ataullakhanov FI. Hemostasis and thrombosis beyond biochemistry: roles of geometry, flow and diffusion. Thromb Res. 2015; 136(4):699-711.
80. Lipets E, Vlasova O, Urnova E, et al. Circulating contact-pathway-activating microparticles together with factors IXa and XIa induce spontaneous clotting in plasma of hematology and cardiologic patients. PLoS One. 2014; 9(1):e87692
81. Koltsova EM, Kuprash AD, Dashkevich NM, et al. Determination of fibrin clot growth and spatial thrombin propagation in the presence of different types of phospholipid surfaces. Platelets. 2021; 32(8):1031-1037.
82. Kuprash AD, Shibeko AM, Vijay R, et al. Sensitivity and Robustness of Spatially Dependent Thrombin Generation and Fibrin Clot Propagation. Biophys J. 2018; 115(12):2461-2473.
83. Koltsova EM, Balandina AN, Grischuk KI, et al. The laboratory control of anticoagulant thromboprophylaxis during the early postpartum period after cesarean delivery. J Perinat Med. 2018; 46(3):251-260.
84. Lobastov K, Dementieva G, Soshitova N, et al. Utilization of the Caprini score in conjunction with thrombodynamic testing reduces the number of unpredicted postoperative venous thromboembolism events in patients with colorectal cancer. J Vasc Surg Venous Lymphat Disord. 2020;8(1):31-41.
85. Seregina EA, Kumskova MA, Gracheva MA, Poletaev AV, Kopilov KG, Ataullakhanov FI. The Global Hemostatic Thrombodynamics Assay in Healthy Children. Thromb Haemost Res. 2021; 5(1):1055.
86. Ovanesov MV, Ananyeva NM, Panteleev MA, Ataullakhanov FI, Saenko EL. Initiation and propagation of coagulation from tissue factor-bearing cell monolayers to plasma: initiator cells do not regulate spatial growth rate. J Thromb Haemost. 2005; 3(2):321-331.
87. Ivanov P, Tsvyatkovska T, Konova E, Komsa-Penkova R. Inherited thrombophilia and IVF failure: the impact of coagulation disorders on implantation process. Am J Reprod Immunol. 2012; 68(3):189-198.
88. Popov AA, Budykina TS, Loginova EA, Stotskaya TV. Efficiency of preventing thromboembolic events with nadroparin calcium in morbidly obese and normal weight patients during laparoscopic hysterectomy. Russian Bulletin of Obstetrician-Gynecologist. 2018; 18(1):51-58. (**In Russ. with Eng. abstract, link**)
89. Vagin IV, Karipidi GK, Barishev AG, et al. Using the thrombodynamics test in the integrated perioperative monitoring of the blood coagulation system in surgical patients. Kuban Scientific Medical Bulletin. 2018; 25(6):44-49. (**In Russ. with Eng. abstract, link**)
90. Gracheva MA, Urnova ES, Sinauridze EI, et al. Thromboelastography, thrombin generation test and thrombodynamics reveal hypercoagulability in patients with multiple myeloma. Leuk Lymphoma. 2015; 56(12):3418-3425.
91. Ignatyev SV, Zotina EN, Fokina ES, et al. Procoagulantive state in patients with non-hodgkin's lymphomas. Tromboz, Gemostaz i Reologiya. 2018; 3:35-40. (**In Russ. with Eng. abstract, link**)

92. Peshkova AD, Evdokimova TA, Sibgatullin TB, Ataullakhanov FI, Litvinov RI. Changes in the parameters of thrombodynamics and blood clot contraction in patients with rheumatoid arthritis. Rheumatology Science and Practice. 2020; 58(3):294-303. (**In Russ. with Eng. abstract, link**)
93. Seregina EA, Tsvetaeva NV, Nikulina OF, et al. Eculizumab effect on the hemostatic state in patients with paroxysmal nocturnal hemoglobinuria. Blood Cells Mol Dis. 2015; 54(2):144-150.
94. Evtugina NG, Sannikova SS, Peshkova AD, et al. Peculiarities of blood coagulation disorders in patients with COVID-19. Terapevticheskii Arkhiv. 2021; 93(11):1255-1263. (**In Russ. with Eng. abstract, link**)
95. Koltsova EM, Sorokina MA, Pisaryuk AS, et al. Hypercoagulation detected by routine and global laboratory hemostasis assays in patients with infective endocarditis. PLoS One. 2021; 16(12):e0261429.
96. 57. Shlyk IF. Informativeness of the thrombodynamic method in assessing the state of hemostasis in patients with coronary heart disease. Medical Herald of the South of Russia. 2019; 10(2):48-54. (**In Russ. with Eng. abstract, link**)
97. Kuznik BI, Davydov SO, Guseva EC, Smolyakov YN, Stepanov AV, Tsybikov NN. The Role of Blood Cells in Formation of Hemocoagulation Shifts in Essential Hypertension. Pathological physiology and experimental Therapy. 2018; 62(4):84-92. (**In Russ. with Eng. abstract, link**)
98. Seregina EA, Poletaev AV, Bondar EV, Vuimo TA, Ataullakhanov FI, Smetanina NS. The hemostasis system in children with hereditary spherocytosis. Thromb Res. 2019; 176:11-17.
99. Tikhomirova OV, Kozhevnikova VV, Zybina NN, Startseva ON, Bobko OV. A role of insomnia in the development of silent cerebral infarctions. S.S. Korsakov Journal of Neurology and Psychiatry. 2018; 118(9-2):3-7. (**In Russ. with Eng. abstract, link**)
100. Suvorin PA, Khoronenko VE, Smirnova TY, Alexandrova EA. Correlation between thrombodynamics test levels and the incidence of venous thromboembolic complications in prostate cancer patients undergone radical prostatectomy. Retrospective study. Annals of Critical Care. 2021; 1:134-142. (**In Russ. with Eng. abstract, link**)
101. Pryakhin IS, Murashko SS, Berns SA, Pasechnik IN. Effect of concomitant diseases on the hemostasis system in the perioperative period of non-cardiac surgery. Lechaschi Vrach. 2021; 11:73-78. (**In Russ. with Eng. abstract, link**)
102. Seregina EA, Nikulina OF, Tsvetaeva NV, et al. Laboratory tests for coagulation system monitoring in a patient with β-thalassemia. Int J Hematol. 2014; 99(5):588-596.
103. Yatsenko AA, Petrenko TI, Filimonov PN. Cases of thrombotic events in fibrous-cavernous pulmonary tuberculosis patients. Tuberculosis and Lung Diseases. 2017; 95(8):35-40. (**In Russ. with Eng. abstract, link**)
104. Vuimo T, Belikov E, Litinskaya O, Davtyan K. Efficiency of Thrombodynamics for Analysis of Hemostasis in Case of Transitory Ischemic Attack after Radio-frequency Ablation in a Patient with Paroxysmal Atrial Fibrillation. American Journal of Medical Case Reports. 2015; 3(10):333-337.
105. Cherniakov A, Balandina A, Vardanyan D, Grigoriev A, Stupin V. Recent methods of monitoring the effectiveness of heparin treatment in patients with deep vein thrombosis of the lower limbs in hospital. Modern Science: actual problems of theory and practice. 2016; 3:69-77. (**In Russ. with Eng. abstract, link**)
106. Vardanyan DM, Cherniakov AV, Stupin VA, Grigoriev AS, Balandina AN. Monitoring the effectiveness of varfarin treatment in patients with deep vein thrombosis of the lower limbs in hospital. Modern problems of science and education. 2016; 5:60-66. (In Russ. with Eng. abstract, link)
107. Goncharova EI, Spiridonova EA, Balandina AN, et al. Diagnosis of Impairments in the Hemostatic System in the Use of Warfarin in Cardiac Surgical Patients. General Reanimatology. 2015; 11(4):51-59. (In Eng., link)
108. Shikhmetov AN, Lebedev NNi, Zadikyan AM, Risk assessment and prevention of venous thromboembolic complications in surgical patients in the outpatient clinic. Surgeon. 2019; 3(4):57-62. (In Russ. with Eng. abstract, link)
109. Bulanov AY, Bulanova EL, Simarova IB, et al. Hemostasis system in COVID-19 patients with heparin therapy. Russian Journal of Preventive Medicine. 2020; 23(6):85-93. (In Russ. with Eng. abstract, link)
110. Krylov A, Khorobrykh T, Petrovskaya A, Khmyrova S, Agadzhanov V, Khusainova N. Role of thrombodynamics global coagulation test in improving treatment results in patients with coronavirus infection at a covid-19 hospital. Georgian Med News. 2021; (313):72-79.

111.Sapronova NG, Svetova EV, Kantsurov RN, Eroshenko OL, Prostov II, Stagniev DV 2020. Estimation of long-term results of treatment of patients with liver cirrhosis after transmitted by the operation of a transjugular intrahepatic portosystemic shunt. Modern problems of science and education. 2020; 2:110. (**In Russ. with Eng. abstract, link**)
112.Katelnitskaya OV, Kit OI, Katelnitskiy II, Guskova NK, Avanesova KA. Individual approach to postoperative thromboprophylaxis in oncology. Tromboz, Gemostaz i Reologiya. 2018; 3:48-53. (**In Russ. with Eng. abstract, link**)
113.Soshitova NP, Lobastov KV, Dement'eva GI, Laberko LA, Rodoman GV. The Poor Effectiveness and Safety of the Standard Algorithm for the Prevention of Postoperative Venous Thromboembolism Attributable to the Individual Reaction of the Hemostatic System. Journal of Venous Disorders. 2015; 9(4):50-57. (**In Russ. with Eng. abstract, link**)
114.Tarandovskiy ID, Balandina AN, Kopylov KG, et al. Investigation of the phenotype heterogeneity in severe hemophilia A using thromboelastography, thrombin generation, and thrombodynamics. Thromb Res. 2013; 131(6):e274-e280.
115.Ataullakhanov FI, Koltsova EM, Balandina AN, Serebriyskiy II, Vuimo TA, Panteleev MA. Classic and Global Hemostasis Testing in Pregnancy and during Pregnancy Complications. Semin Thromb Hemost. 2016; 42(7):696-716.
116.Voroshilina ES, Ovsepyan RA, Plotko EE, Baskova OYu, Gerasimova OB, Budykina TS, Vuimo TA. The ranges of thrombodynamic assay parameters during physiological pregnancy. Russian Bulletin of Obstetrician-Gynecologist. 2016; 16(1):10-16. (**In Russ. with Eng. abstract, link**)
117.Momot AP, Molchanova IV, Batrak TA, et al. Reference values of hemostatic system parameters in normal pregnancy and after delivery. Russian Journal of Human Reproduction. 2015; 21(1):89-97. (**In Russ. with Eng. abstract, link**)
118.Peshkova AD, Safiullina SI, Evtugina NG, et al. Premorbid Hemostasis in Women with a History of Pregnancy Loss. Thromb Haemost. 2019; 119(12):1994-2004.
119.Balandina AN, Koltsova EM, Teterina TA, et al. An enhanced clot growth rate before in vitro fertilization decreases the probability of pregnancy. PLoS One. 2019; 14(5):e0216724.
120.Safiullina SI, Vuimo TA, Budykina TS, Ilizarova NA. Hormone replacement therapy in a patient with Shereshevsky—Turner syndrome and a history of congenital thrombophilia and estrogen-associated thrombosis. Russian Bulletin of Obstetrician-Gynecologist. 2018; 18(4):84-87. (**In Russ. with Eng. abstract, link**)
121.Safiullina SI, Vuimo TA, Ilizarova NA, Sabirova VL, Sigitova ON, Pyregov AV. Current possibilities of personalized thromboprophylaxis when implementing an assisted reproductive technology protocol and during pregnancy in women at high risk for venous thromboembolism. Akusherstvo i Ginekologiya/Obstetrics and Gynecology. 2018; 4:144-149. (**In Russ. with Eng. abstract, link**)
122.Razin MP, Ignatyev SV, Semakin AS. Coagulation disorders in children with destructive forms of appendicitis. Novosti Khirurgii. 2020; 28:664-670. (**In Russ. with Eng. abstract, link**)
123.Koltsova EM, Balashova EN, Ignatova AA, et al. Impaired platelet activity and hypercoagulation in healthy term and moderately preterm newborns during the early neonatal period. Pediatr Res. 2019; 85(1):63-71.
124.Cherkasova SV, Chugunova OL, Cherkasov SN, Kossova AA, Shumilov PV. Assessment of hemostasis in newborns with necrotizing enterocolitis. Kursk Scientific and Practical Bulletin "Man and His Health". 2019; (4):31-38. (**In Russ. with Eng. abstract, link**)
125.Morozova NY, Burov AA, Dorofeeva EI, et al. Thrombosis of the right atrium in a newborn: a favorable outcome. Pediatric Hematology/Oncology and Immunopathology. 2018; 17(1):87-92. (**In Russ. with Eng. abstract, link**)
126.Vuimo TS, Tsarenko SV, Filimonova EV, Seregina EA, Karamzin SS. Correction of Anticoagulant Therapy in Patients with Severe COVID-19 Virus Infection Using a Thrombodynamics Coagulation Assay. Clinical and Applied Thrombosis/Hemostasis. 2022; 28:1-10
